# Exploring Monomer‐Amino Acid Interactions in Mimicking Mips for PSA Detection—Using the Novel MBASM Approach

**DOI:** 10.1002/jcc.70139

**Published:** 2025-05-21

**Authors:** Lariel Chagas da Silva Neres, Johnatan Mucelini, Gabriel Augusto Pinheiro, Helen Luiza Brandão Silva Ambrósio, Albérico Borges Ferreira da Silva, Maria Del Pilar Taboada Sotomayor, Karla Furtado Andriani

**Affiliations:** ^1^ São Paulo State University (UNESP), Institute of Chemistry Araraquara São Paulo Brazil; ^2^ São Carlos Institute of Chemistry, University of São Paulo (USP) São Carlos São Paulo Brazil; ^3^ Institute of Science and Technology, Federal University of São Paulo (UNIFESP) São José dos Campos São Paulo Brazil; ^4^ Department of Exact Sciences State University of Santa Cruz (UESC) Ilhéus Bahia Brazil

**Keywords:** algorithm, DFT, MIP, molecular docking, PSA

## Abstract

Given the rising incidence of prostate cancer (PCa), there is an increasing demand for cost‐effective and reliable methods for early detection using the prostate‐specific antigen (PSA) biomarker. PCa remains a leading cause of mortality among individuals with prostates aged 55–80 years. Molecularly Imprinted Polymers (MIPs) represent a promising solution due to their selectivity, sensitivity, and stability for PSA detection. However, the synthesis of MIPs for protein targets presents significant challenges, particularly in the rational selection of functional monomers and cross‐linkers. This study introduces a theoretical framework to aid the development of MIPs by assisting in the selection of optimal reagents for PSA targeting. A novel algorithm, the Molecular Binding Algorithm for Surface Mapping (MBASM), was developed to efficiently generate amino acid‐monomer complexes. The integrated MBASM + DFT approach was validated through comparison with the GFN2‐xTB method and the Quantum Cluster Growth approach implemented in the CREST program. The results demonstrated strong agreement between the methods, establishing MBASM + DFT as a viable and innovative alternative tool for predicting interaction structures and energies. Through this strategy, promising monomers for PSA‐targeted MIP synthesis were identified, including itaconic acid, 4‐imidazole acrylic acid, and methacrylic acid, with 1,4‐divinylbenzene emerging as the most effective cross‐linker. This computational methodology provides a powerful and systematic approach for optimizing MIP synthesis aimed at selective PSA detection.

## Graphical abstract

This study presents the novel Molecular Binding Algorithm for Surface Mapping (MBASM) to identify optimal amino acid‐functional monomer complexes for enhancing molecularly imprinted polymers (MIPs) in PSA detection. By screening the combination of 20 amino acids and 15 functional monomers, we uncover promising complexes, establishing a basis for future MIP applications.

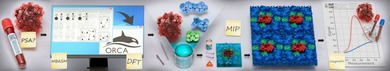

**Abbreviations**
DFT: density functional theory
MIP: molecularly imprinted polymer
PSA: prostate‐specific antigen

## 1 Introduction

Prostate cancer (PCa) represents a significant global health concern, with its incidence steadily increasing in recent years. It is a leading cause of mortality among individuals aged 55 to 80 years with a testicular reproductive system, accounting for approximately 10% of all cancer‐related deaths worldwide and ranked as the sixth deadliest cancer globally [1, 2]. The progression of PCa to metastatic stages, often due to delayed diagnosis, dramatically worsens its prognosis [3, 4]. Therefore, early detection and timely intervention are critical, as they are strongly associated with favorable outcomes, particularly when the disease remains confined to the prostate lobes. PCa is often asymptomatic in its early stages, with significant symptoms typically emerging only after metastasis to lymph nodes or bones [2]. This underscores the urgent need for reliable diagnostic tools capable of detecting PCa in its early stages to improve treatment success and patient outcomes.

In this context, prostate‐specific antigen (PSA) has become the most widely adopted biomarker for PCa, as it is the only known substance in the human body specific for this application [1, 3]. PSA is a serine protease commonly found in human serum that facilitates minimally invasive PCa screening [1, 4]. As a member of the kallikrein family of proteases, PSA functions as a glycoprotein involved in androgen regulation, with gene transcription upregulated by androgen receptor activity [5]. PSA exists in various isoforms, with isoelectric points ranging from 6.8 to 7.2 [3]. Clinically, the “gray zone” of PCa risk is associated with PSA concentrations between 4 and 10 ng ${\text{mL}}^{-1}$. PSA levels below 4 ng ${\text{mL}}^{-1}$ are generally considered negative, indicating a low probability of PCa [3]. However, studies have shown that approximately 25% of individuals with PSA levels between 2.5 and 4 ng ${\text{mL}}^{-1}$ were diagnosed with PCa, highlighting the limitations of current diagnostic thresholds [1, 6].

Nevertheless, while PSA is widely used as a biomarker for PCa, its specificity to prostate cancer is limited. PSA levels tend to increase with patient age and may also reflect non‐cancerous conditions, such as benign prostatic hyperplasia, or even other malignancies, such as breast cancer [4, 5, 7]. The most common method for detecting PSA levels in clinical practice is the enzyme‐linked immunosorbent assay (ELISA), which is highly sensitive and effective for quantifying PSA. However, ELISA is a complex and time‐consuming procedure. Furthermore, PSA instability during analysis can contribute to suboptimal results, increasing the risk of errors [1, 3]. Thus, ELISA assays are relatively expensive compared to alternative diagnostic methods. Despite these limitations, ELISA remains the gold standard for PSA detection and continues to play a central role in PCa diagnostics and monitoring [1, 3, 8].

In recent years, molecularly imprinted polymers (MIPs) have gained significant attention in academic research as a promising, cost‐effective, more stable, and easier‐to‐handle alternative to ELISA [9, 10]. MIPs offer several advantages over ELISA, including straightforward synthesis without the need for sophisticated equipment. Additionally, MIPs are reusable, leading to substantial cost savings in repeated analysis [11]. They are highly versatile, functioning effectively across a wide range of temperatures, pH levels, and in organic‐aqueous solutions. Furthermore, their high selectivity for target molecules minimizes the occurrence of false positives and false negatives [12, 13, 14]. Despite these advantages, MIPs are still under the development stage and require further validation before they can be reliably applied in clinical diagnostics [1, 15]. Nevertheless, their potential to address the limitation of current PSA detection methods makes them a compelling focus of research in the quest for more efficient and cost‐effective PCa diagnostics.

The synthesis of MIPs relies on five essential elements: The template molecule or analyte (TM), the functional monomer (FM), the cross‐linker or structural monomer (SM), the radical initiator, and the solvent. The characteristics and concentrations of these components are critical for achieving optimal polymer performance [16, 17]. However, MIP development for macromolecules such as proteins presents significant challenges, particularly with respect to achieving high selectivity [16, 18]. One notable challenge is the decrease in selectivity with increasing TM size, making PSA a difficult target for MIP design [16]. The wide variety of available FMs further complicates the selection process, as the chemical compatibility between the FM and analyte is crucial—acidic monomers interact more effectively with basic regions of the analyte and vice versa [19].

To overcome these complexities, the integration of computational approaches with experimental strategies offers a promising pathway for the design and optimization of selective PSA‐sensitive MIPs [16, 18]. This combined approach minimizes the need for extensive experimentation, thereby saving both time and resources. Currently, Density Functional Theory (DFT) and Molecular Docking are widely employed to evaluate binding affinities and interaction sites. These methods provide valuable insights into monomer‐template interactions, enabling predictions of molecular properties that guides the selection of functional monomers (FM), structural monomers (SM), and solvents, thereby improving MIP design and application efficiency [13, 16, 18, 20, 21, 22, 23, 24, 25].

Despite their advantages, DFT methods are computationally expensive, particularly when handling large datasets involving thousands of molecular structures. These constraints limit the systematic exploration of monomer‐analyte configurations and reduce research efficiency. Consequently, many studies rely on manually constructed interaction models, narrowing the configuration range to be explored and potentially overlooking optimal binding scenarios [16, 26, 27, 28, 29].

To overcome this barrier, deterministic algorithms—which operate exclusively on input data without stochastic variation—have emerged as efficient tools for automating complex (analyte + monomer) generation and improving the scalability and reliability of molecular simulations [30]. In this study, we introduce the Molecular Binding Algorithm by Surface Mapping (MBASM), a novel algorithm designed to automatically construct monomer‐analyte interaction complexes by evaluating molecular chemical environments. MBASM significantly enhances the efficiency of structure generation and enables broader sampling of potential configurations. When integrated with DFT, the algorithm yields results comparable to those obtained using semi‐empirical methods such as GFN2‐xTB + DFT. Future work will focus on refining the functionality of the algorithm and improving its performance in high‐throughput screening for MIP design.

## 2 Theoretical Approach and Computational Details

### 2.1 Introduction to MBASM in Molecular Simulation

The Molecular Binding Algorithm by Surface Mapping (MBASM) differentiates itself from other structure‐generation algorithms by implementing a unique protocol that integrates several internal and external tools to achieve its main goal, that is, constructing interacting complexes such as monomer‐analyte systems, van der Waals (vdW) complexes, adsorbed systems, and others. This protocol performs a sophisticated analysis of the chemical environment, followed by the identification and selection of representative interaction sites to ensure the accurate generation of physically meaningful complexes, with a primary focus on molecule–molecule interactions.

In MBASM, molecules are treated as rigid bodies, and atoms are represented as spheres with vdW radii or scaled fractions thereof. The algorithm maps the chemical environment on the molecular surface based on local atomic neighborhoods, allowing the systematic exploration of interaction modes between molecular pairs. This methodology enables the generation of hundreds of thousands of analyte–monomer complexes, which would be computationally infeasible to analyze without an effective data reduction strategy.

To address this challenge, MBASM incorporates the *k‐means* clustering algorithm—a widely used method for partitioning high‐dimensional datasets into representative clusters [31, 32, 33, 34, 35, 36]. By applying *k‐means*, the algorithm efficiently extracts representative structures from the large ensemble of generated complexes for subsequent quantum mechanical evaluation via DFT. For more details on *k‐means*, see the work of MacQueen [32].

#### 2.1.1 Surface Mapping

**FIGURE 1:**
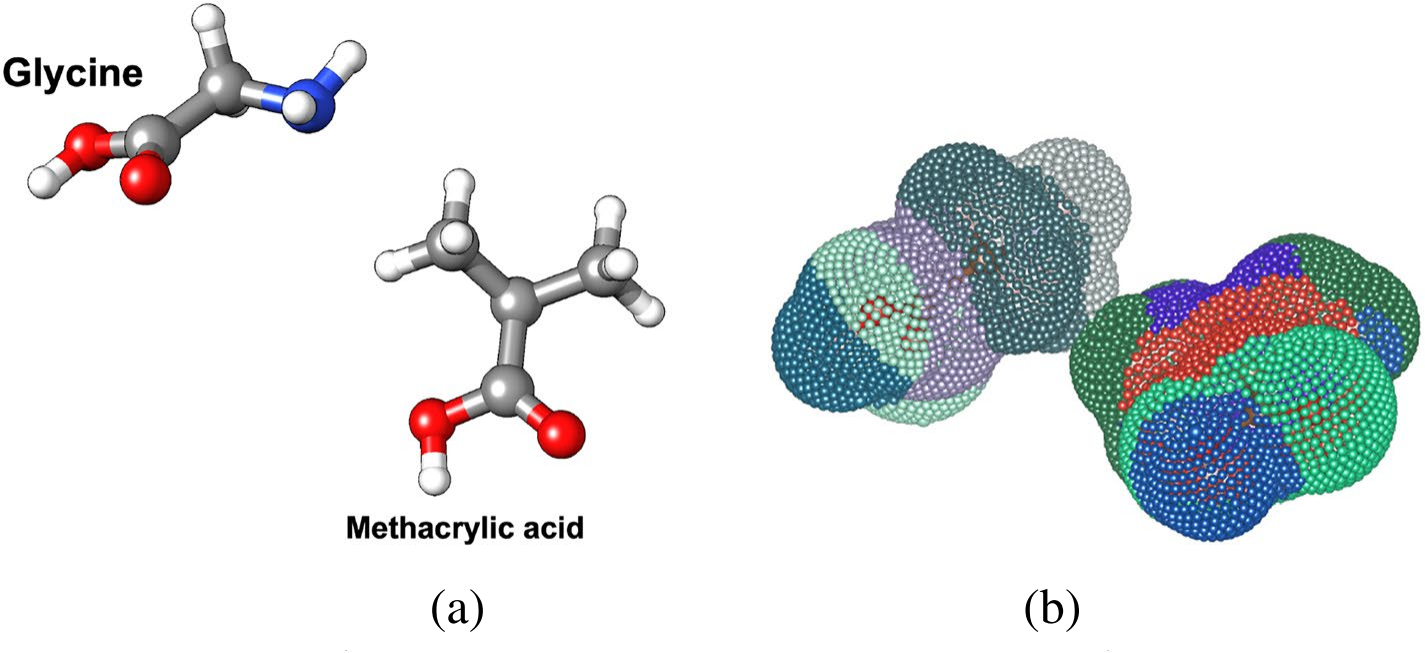
(a) Input structures, here showing a glycine molecule interacting with a methacrylic acid structure, and forming a van der Waals complex, (b) example of surface point mapping on structures. The number of points mapped is determined by user settings, showcasing the flexibility and precision of the algorithm in surface analysis.

The objective of this initial stage is to identify a set of *k* representative points on the molecular surface that capture the diversity of local chemical environments. This is accomplished through the following steps:

- **Surface Point Identification**: Firstly, the molecule surface is defined as the outermost surface formed by the union of vdW radii spheres around each atom. A large and predetermined number of surface points is then generated using the Deserno algorithm [37], ensuring uniform and comprehensive coverage of the molecular surface.
- **Feature Vector Calculation for Surface Points**: For each surface point, a feature vector is constructed to quantify the local chemical environment. The vector elements decay with the distance d from the point to each atom in the molecule, following the exponential function $e^{-d^{2/3}}$, which assigns greater weight to nearby atoms. These contributions are categorized and grouped based on the atomic species. Subsequently, *k‐means* clustering is applied to the feature vectors, grouping surface points into *k* clusters. The point closest to the centroid of each cluster is selected as the representative for that specific local chemical environment (see Figure 1).

#### 2.1.2 Binding Procedure Algorithm

The trial binding structures are generated by aligning two molecules at their representative interaction points on the molecular surfaces. To comprehensively explore potential binding geometries, multiple spatial alignments are evaluated. For each pair of representative points, one molecule undergoes systematic rotation to generate a full set of configurational complexes. A controlled rotational grid is employed to sample all feasible three‐dimensional orientations, ensuring thorough exploration of the interaction space [38, 39]. This approach produces a large dataset of trial configurations, which are then filtered and refined according to the following criteria:

- **Non‐overlapping Molecules:** Configurations in which atoms from the two molecules overlap are excluded. Overlap is defined as any pair of atoms from different molecules being separated by a distance less than the sum of their vdW radii, multiplied by a weighted parameter (σ). After testing several values for this σ parameter, 0.5 was identified as optimal for monomer‐analyte interactions.
- **Distinct Configurations:** Configurations must be sufficiently distinct to avoid redundancy in the dataset. This is enforced through a filtering process based on geometric feature vectors. Each binding configuration is described by a concatenated feature vector composed of three key spatial points, that is, the geometric centers of both molecules and the representative surface points used to align them. The Euclidean distance between the feature vector of the current configuration and those of previously accepted configurations is computed. If this distance exceeds a specified threshold (0.08 was determined to be optimal for this study), the configuration is retained in the final pool of selected structures.

#### 2.1.3 Representative Set Extraction Algorithm

**FIGURE 2:**
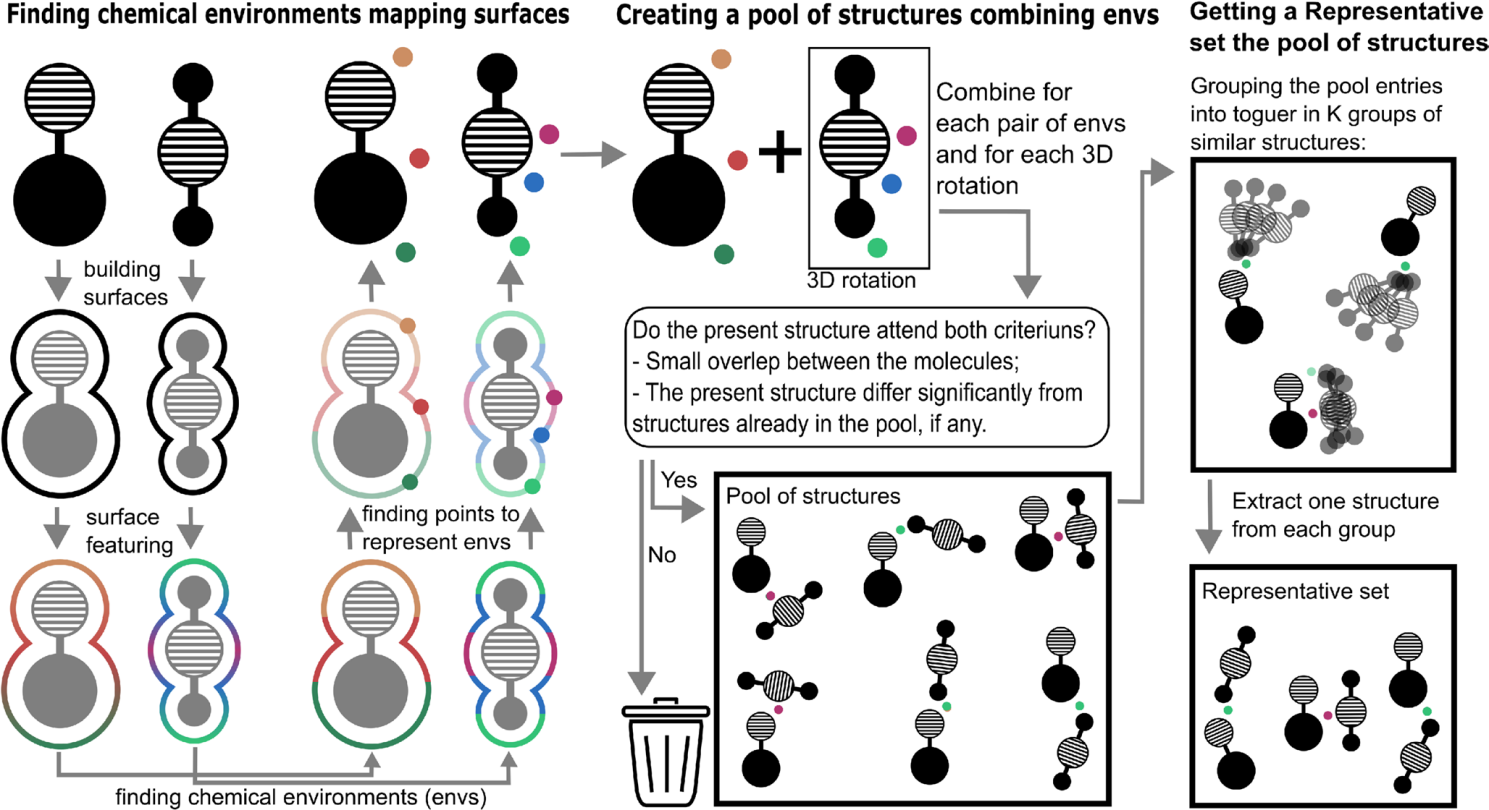
Schematic representation of the Molecular Binding Algorithm by Surface Mapping (MBASM), illustrating the workflow for identifying chemical environments, generating a pool of structures, and selecting a representative set for further analysis.

Finally, the selected structures from the filter pool are further clustered using the *k‐means*algorithm, resulting in a refined and smallest set of representative configurations. To visualize the clustering process, the t‐distributed Stochastic Neighbor Embedding (t‐SNE) algorithm is employed (Figure SM36). This dimensionality reduction technique, developed by Geoffrey Hinton and Laurens van der Maaten [40], enables intuitive visualization of high‐dimensional data in a lower‐dimensional space, facilitating the interpretation of structural diversity within the dataset. Figure 2 illustrates the complete workflow of the MBASM algorithm, detailing the key steps involved in identifying chemically relevant surface environments, generating a comprehensive pool of candidate complexes, and selecting a representative subset for further analysis.

Using the MBASM protocol, millions of chemical structures were generated and systematically filtered to identify representative monomer‐analyte configurations appropriate for DFT calculations. The initial stages of the protocol focused on evaluating a broad range of monomer‐amino acid complexes to identify those with the highest potential for effective molecular interactions. However, performing DFT calculations on such large datasets would be computationally expensive. To overcome this limitation, the pool of trial complexes was reduced to approximately 2400 representative monomer‐analyte configurations. These optimized structures were selected based on their chemical relevance and structural diversity, ensuring that the most promising candidates were retained for subsequent DFT analysis. The theoretical framework and corresponding results are presented and discussed in the following subsections.

### 2.2 Ab Initio DFT Calculations

All total energy calculations were performed using ab initio DFT methods, employing the B3LYP formulation for the exchange‐correlation functional [41]. Thus, to solve the Kohn‐Sham (KS) equations, the KS orbitals were expanded on Gaussian‐type basis functions as implemented in the ORCA software package [42, 43], using the triple‐zeta valence with polarization (TZVP) basis set quality [44]. For convergence criteria, we employed the Tight (default) ORCA settings, in which the electron density self‐consistency was achieved once the total energy was smaller than $1.0\times 10^{-8}$ Hartree, while the equilibrium geometries were obtained once the forces on all atoms were smaller than $1.0\times 10^{-5}$ Hartree/Bohr. These criteria ensured accurate, optimized geometries for all studied amino acid‐monomer complexes. Vibrational frequencies calculations were also carried out for all isolated amino acids (AA) and monomers at the same level of theory to verify them as local minima. This was confirmed by the absence of negative eigenvalues in the Hessian matrix (Figures SM1 to SM35). Thus, to account for solvent effects, the implicit Conductor‐like Polarizable Continuum Model (CPCM) was applied, using water as solvent [45].

### 2.3 Molecular Docking Simulations

**FIGURE 3:**
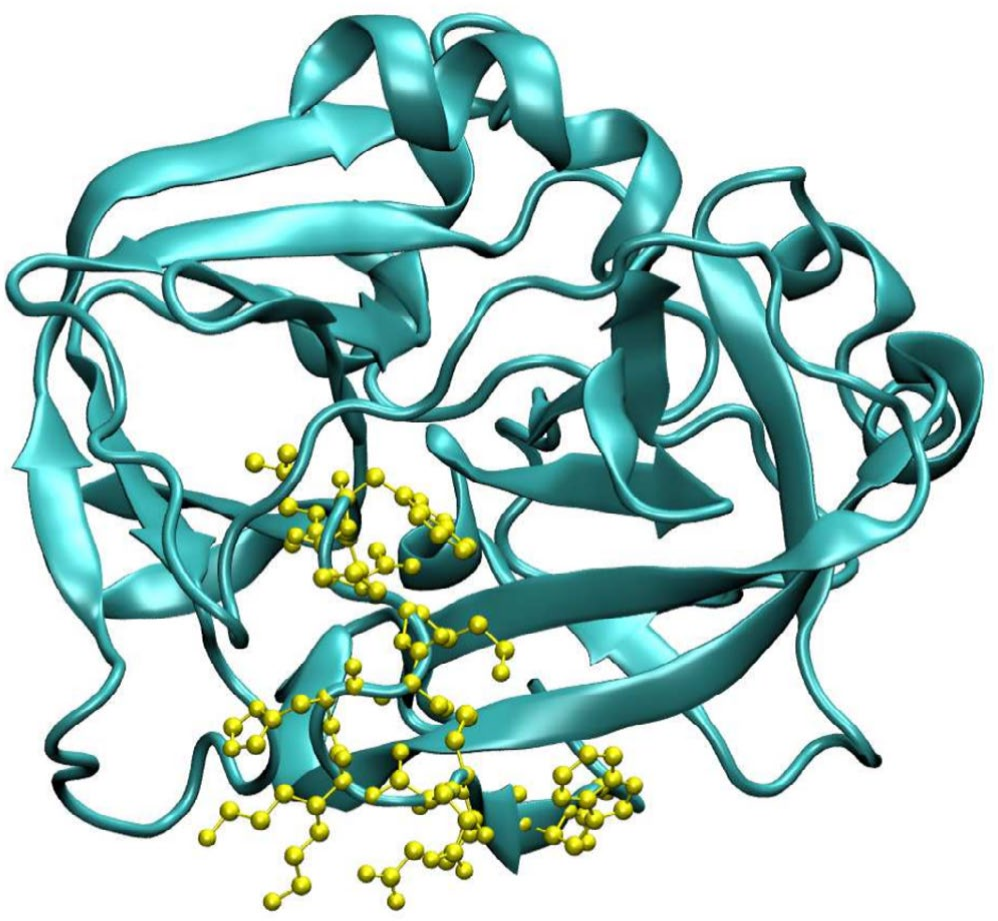
The amino acids highlighted in yellow form a large peptide identical to the one found in human PSA, with this region also containing several other identical AA. A detailed sequence comparison between HPK and human PSA is provided in Carvalho (2002) [49, 50].

Molecular docking using a flexible‐rigid ligand‐receptor model was performed with the AutoDock Vina v1.2.0 software [46] in conjunction with AutoDock Tools 1.5.6 [47]. The PSA protein structure was obtained from the PDB website [48], code: 1GVZ with a resolution of 1.42 Å [49] (see Figure 3). Although 1GVZ corresponds to horse PSA (HPK), it shares over 60% amino acid sequence similarity with human PSA, including several identical regions [49]. Homology analysis between HPK and human PSA was performed using BLAST analysis, based on FASTA sequence data [51, 52]. The analysis highlights key sequence similarities and differences, providing valuable insights into their structural and functional relationships (Figures SM44 and SM45). Furthermore, given the size and inherent structural flexibility of protein molecules, molecular imprinting is often limited to using surface‐exposed peptide fragments, known as epitopes. This strategy, known as Epitope Molecularly Imprinted Polymers (EMIPs), was introduced by Rachkov and Minoura in 2001 [53, 54]. In this approach, the amino acid sequence of the epitope used as a template is identical to a terminal segment of the target protein. EMIPs offer several advantages, including exceptionally high selectivity, lower cost, easy availability of template molecules, and high efficiency in template immobilization on the matrix surface [53, 54, 55]. Additionally, due to their simpler structure, epitopes are less sensitive to environmental conditions and can be used with organic solvents, thereby broadening the range of monomers available for MIP synthesis [55].

In addition to the aforementioned methodology, two crucial considerations were incorporated to ensure that the docking simulations focused on the most relevant binding regions of the PSA protein:

- **Active Site Selection:** The active site was chosen based on its sequence similarity to the human PSA binding site. Specifically, the selected region included a significant number of AA also present in the human PSA active site, ensuring that the predictive binding characteristics would be relevant for intended applications.
- **Surface Accessibility:** The selected binding region was required to be surface‐exposed. This consideration is particularly important because the functional monomer is expected to interact with the protein surface during polymerization. Therefore, ensuring surface accessibility promotes effective binding to the epitope and facilitates the formation of MIP.

Considering these criteria, molecular docking simulations were performed at physiological pH (7.4), setting the protonation states of both the ligand (monomer) and receptor (PSA). Docking was carried out using AutoDock Vina in a flexible‐rigid ligand‐receptor regime, employing default parameters with the exception of the exhaustiveness setting, which was increased to 10 to enable a more comprehensive exploration of binding conformations. The homologous regions of horse PSA (HPK) corresponding to human PSA active site were used to define the docking grid box (8.795×43.245×−5.193; Figures SM44 and SM45).

## 3 Results and Discussion

### 3.1 Evaluating MBASM Performance in Monomer‐Analyte Complexes

**FIGURE 4:**
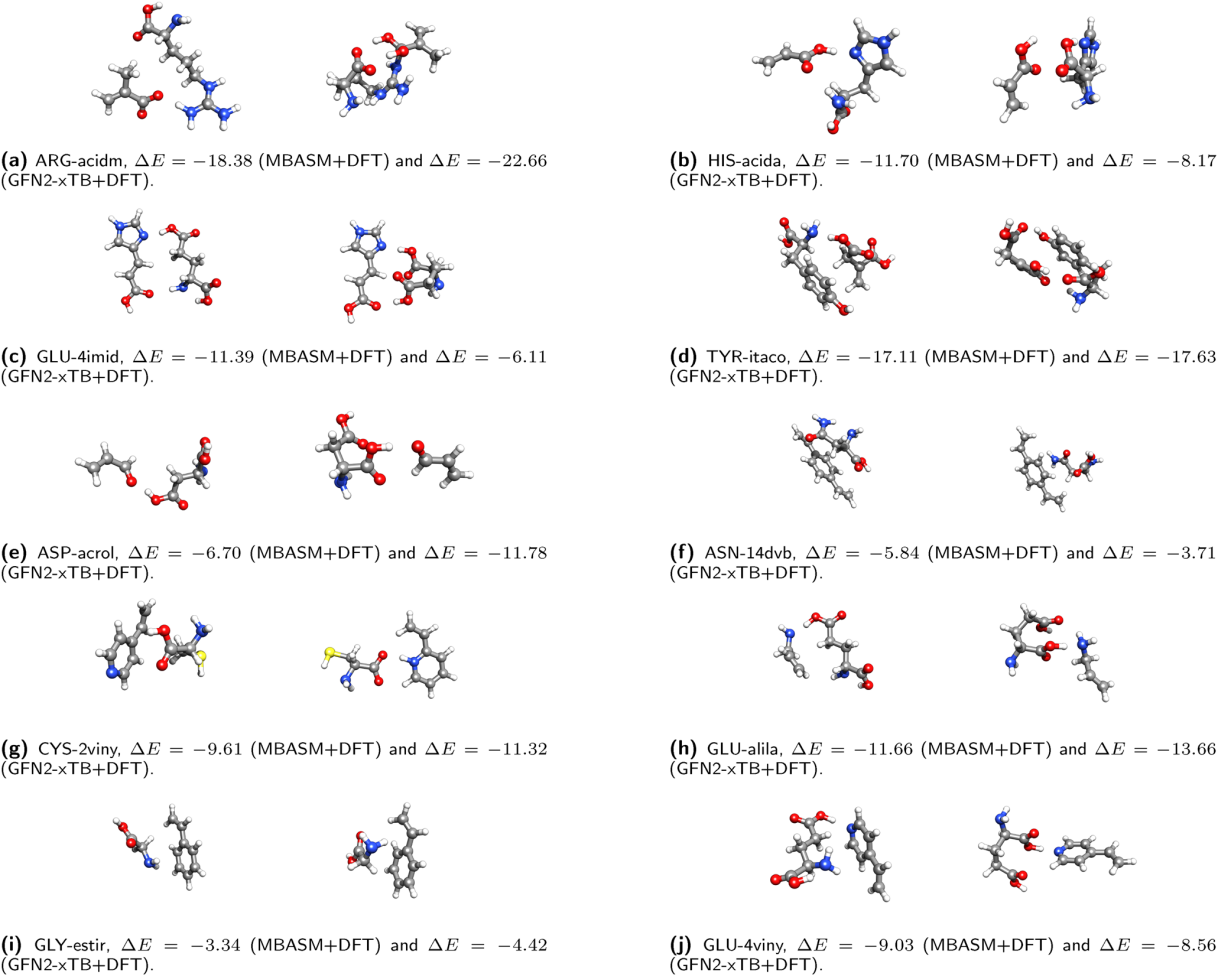
Comparison of optimized amino acid‐monomer complexes obtained using the MBASM+DFT and GFN2‐xTB+DFT approaches. The binding energy (ΔE) is reported in kcal mol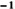. (a) ARG‐acidm, ΔE=−18.38 (MBASM+DFT) and ΔE=−22.66 (GFN2‐xTB+DFT), (b) HIS‐acida, ΔE=−11.70 (MBASM+DFT) and ΔE=−8.17 (GFN2‐xTB+DFT), (c) GLU‐4imid, ΔE=−11.39 (MBASM+DFT) and ΔE=−6.11 (GFN2‐xTB+DFT), (d) TYR‐itaco, ΔE=−17.11 (MBASM+DFT) and ΔE=−17.63 (GFN2‐xTB+DFT), (e) ASP‐acrol, ΔE=−6.70 (MBASM+DFT) and ΔE=−11.78 (GFN2‐xTB+DFT), (f) ASN‐14dvb, ΔE=−5.84 (MBASM+DFT) and ΔE=−3.71 (GFN2‐xTB+DFT), (g) CYS‐2viny, ΔE=−9.61 (MBASM+DFT) and ΔE=−11.32 (GFN2‐xTB+DFT), (h) GLU‐alila, ΔE=−11.66 (MBASM+DFT) and ΔE=−13.66 (GFN2‐xTB+DFT), (i) GLY‐estir, ΔE=−3.34 (MBASM+DFT) and ΔE=−4.42 (GFN2‐xTB+DFT), and (j) GLU‐4viny, ΔE=−9.03 (MBASM+DFT) and ΔE=−8.56 (GFN2‐xTB+DFT).

In this study, we assessed the effectiveness of the MBASM protocol combined with DFT calculations for generating and optimizing monomer‐analyte complexes. Its performance was benchmarked against a well‐established automated approach based on the semi‐empirical GFN2‐xTB method, combined with the Quantum Cluster Growth (QCG) strategy in the Conformer‐Rotamer Sampling Tool (CREST) program [56]. CREST was selected for its demonstrated efficiency in exploring low‐energy conformational spaces, particularly in systems dominated by interactions that are non‐covalent in nature [56]. Our comparative analysis began with a direct evaluation of the MBASM + DFT protocol relative to the GFN2‐xTB approach (Figure SM37. This was followed by an assessment of DFT‐optimized structures derived from GFN2‐xTB‐generated conformers (GFN2‐xTB followed by DFT optimization). Specifically, we compared the most stable structures generated by the CREST + GFN2‐xTB + DFT approach with those obtained using our MBASM + DFT protocol, as summarized in Figure 4.

The results demonstrate the comparative efficiency and accuracy of the MBASM + DFT protocol compared with the automated CREST + GFN2‐xTB + DFT approach in generating and optimizing monomer‐analyte complexes. For instance, our protocol consistently produced a broader and more chemically diverse range of initial complexes compared to the GFN2‐xTB method. This highlights the capability of MBASM to explore the configurational space more exhaustively, particularly for interactions sensitive to local chemical environments. Thus, the monomer‐analyte DFT‐optimized structures derived from the MBASM protocol were comparable in stability to those generated by the CREST + GFN2‐xTB approach. The calculated binding energies of the most stable structures from both methods showed only minor energetic differences within the margin of computational error. However, in some cases, the CREST + GFN2‐xTB + DFT approach produced slightly more favorable binding energy magnitudes, which are discussed in detail in subsequent sections.

Despite its exhaustive sampling of configurations, MBASM efficiently reduced the trial complexes pool to a manageable and representative subset for DFT optimization. In contrast, the CREST + GFN2‐xTB approach generated a much larger number of candidate structures, requiring significantly more computational resources for processing and post‐optimization. Furthermore, while the GFN2‐xTB approach is designed for rapid pre‐optimization, it occasionally converged to local minima that demanded extensive refinement during subsequent optimization using DFT. The MBASM protocol, by leveraging its chemically informed surface‐mapping algorithm, consistently generated high‐quality initial configurations, thereby minimizing the need for computationally expensive post‐processing.

The initial results suggest that the binding energies obtained using raw GFN2‐xTB exhibit significant energetic discrepancies when compared to those calculated with MBASM + DFT (Figure SM37). For instance, in the ARG‐acidm complex, MBASM + DFT predicted a binding energy of −18.38 kcal mol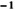, whereas GFN2‐xTB resulted a much lower energy of −29.27 kcal mol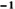. Therefore, these results highlight the potential limitations of semi‐empirical methods in accurately estimating binding energies, likely due to an oversimplified treatment of electronic interactions and inadequate representation of polarization effects.

**FIGURE 5:**
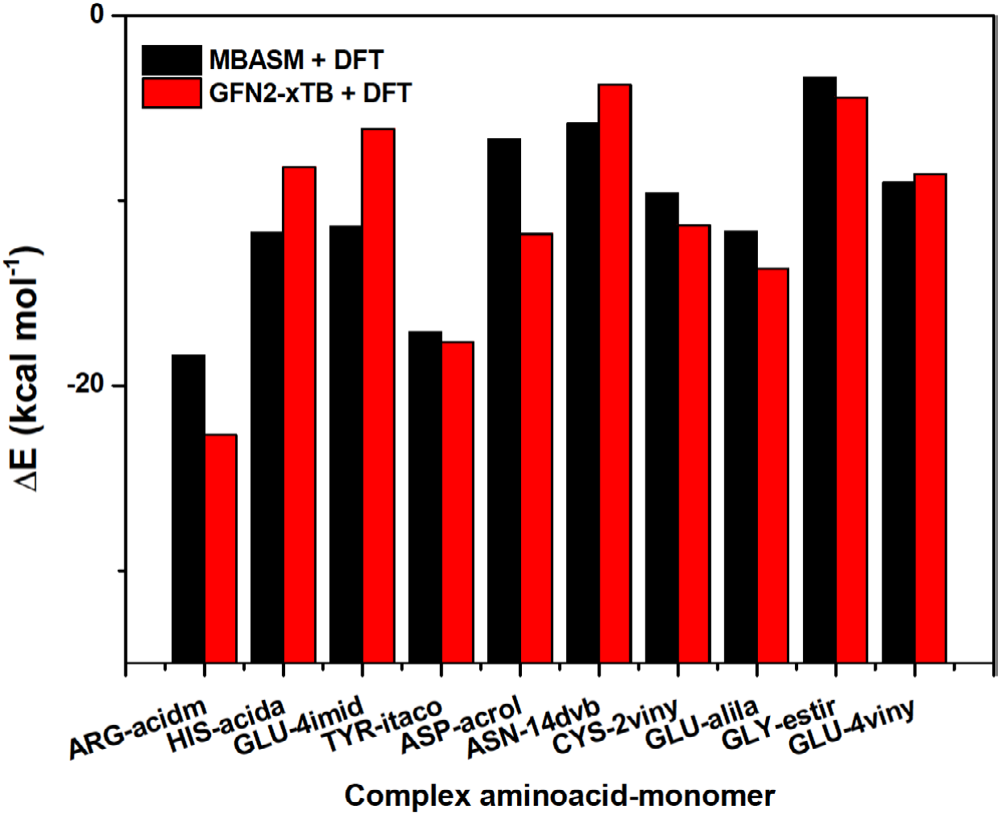
Binding energy relationship (ΔE in kcal mol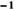) for the most relevant amino acid‐functional monomer complexes, comparing the MBASM+DFT methodology with CREST+GFN2‐xTB+DFT.

However, when the GFN2‐xTB structures were subsequently refined via DFT, the discrepancy was notably reduced. The DFT‐optimized GFN2‐xTB structure for the ARG‐acidm complex yielded a binding energy of −22.66 kcal mol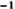 (see Figure 5). This enhanced stability is attributed to proton migration during the DFT optimization, which resulted in a more favorable electronic configuration. This behavior illustrates the utility of GFN2‐xTB in generating reasonable intermediate structures that can be improved through higher‐level post‐optimization. In contrast, the MBASM + DFT protocol inherently incorporated critical non‐covalent interactions—such as the formation of two hydrogen bonds—during its initial complex generation phase. This capability stems from MBASM's chemically informed surface‐mapping strategy, which enables the construction of highly relevant configurations prior to quantum mechanical refinement. As a result, MBASM + DFT achieved greater stabilization without the need for significant structural corrections, underscoring its effectiveness in capturing significant non‐covalent interactions directly, particularly when hydrogen bonding or other specific interactions play a crucial role in binding stability.

Consistent results were also observed for the TYR‐itaco and GLU‐4imid complexes. In the case of TYR‐itaco, the MBASM + DFT approach yielded a binding energy of −17.11 kcal mol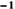, while the GFN2‐xTB + DFT method produced a value of −17.63 kcal mol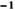. The close agreement between these values demonstrates that the MBASM protocol is capable of generating chemically meaningful initial structures that are well‐aligned with refined results obtained from other established methodologies. This further supports the utility of MBASM as a reliable and efficient alternative for the high‐throughput generation of monomer‐analyte complexes, offering flexibility in identifying low‐energy structures with high accuracy.

Ongoing and future development of the MBASM algorithm will focus on introducing new functions to enhance its accuracy and applicability. The results presented above highlight areas for further improvement, such as expanding the sampling of configurations to capture additional interactions, including multiple hydrogen bonds in specific cases (e.g., ASP‐acrol). Enhancing the chemical environment mapping or incorporating adaptive sampling methods could further improve the accuracy and breadth of generated complexes. Future versions of the MBASM code may include advanced clustering algorithms for improved structural selection, more accurate treatment of protonation states, and integration with machine learning models to dynamically predict interaction sites and optimize binding geometries. These enhancements are expected to increase the protocol's robustness and scalability across a wider range of molecular systems and applications.

### 3.2 Structural, Energetic, and Electronic Features of Monomer‐Analyte Complexes

#### 3.2.1 Energetic Analysis: Evaluation of the Binding Energy

Using DFT, the binding interactions between a diverse array of monomers and 20 AA were systematically evaluated. For all calculations involving isolated molecules and complexes, an implicit water model was employed to account for the biological nature of the system under investigation. In total, approximately 15 monomers were analyzed. The monomers included in this study are abbreviated as follows:

- *Functional monomers* and their abbreviations: 1‐allyl piperazine (*1ally*), 1‐vinylimidazole (*4imid*), 4‐vinylpyridine (*2hydr*), 2‐vinylpyridine (*1viny*), 4‐imidazoleacrylic acid (*4viny*), 2‐hydroxyethyl methacrylate (*acidm*), acrylamide (*2viny*), methacrylic acid (*acril*), acrolein (*acrol*), acrylic acid (*acida*), allylamine (*alila*), styrene (*estir*), itaconic acid (*itaco*).
- *Structural monomers* and their abbreviations: 1,4‐divinylbenzene (*14dvb*), N,N'‐methylenebisacrylamide (*bisac*).

**FIGURE 6:**
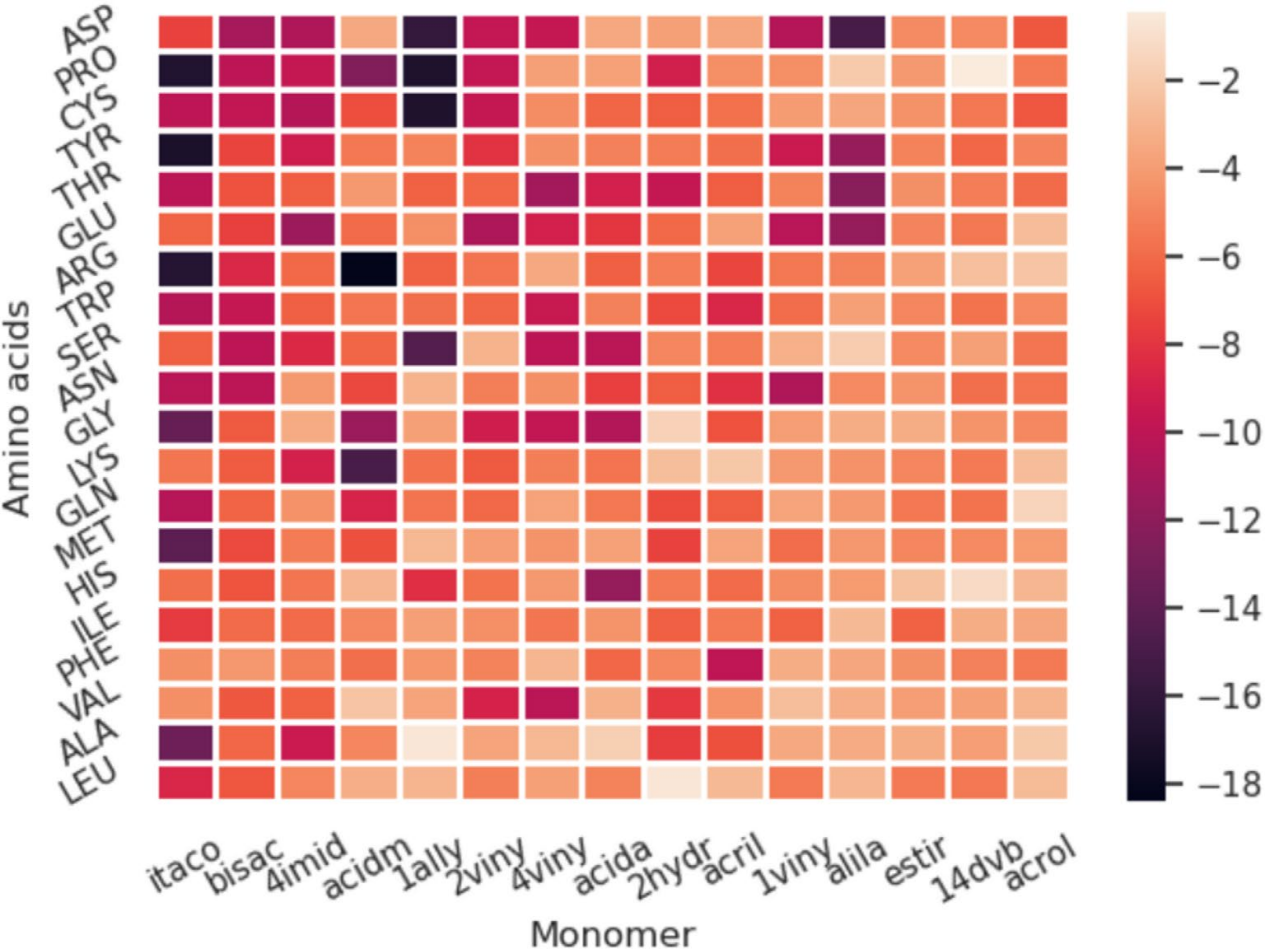
Heatmap of binding energy (Δ E in kcal mol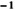) for all optimized configurations of amino acid‐monomer complexes obtained using the MBASM+DFT approach.

Therefore, the strength of the representative complexes (AA + monomers) was evaluated through binding energy analysis (ΔE) using the following formula:

(1) $$\Delta E=(E_{\text{amino acid}}^{DFT}+E_{\text{monomer}}^{DFT})-E_{\text{complex}}^{DFT}$$

where $E_{\text{amino acid}}^{DFT}$ and $E_{\text{monomer}}^{DFT}$ are the DFT total energy for the gas‐phase amino acid and monomer molecules, respectively, while $E_{\text{complex}}^{DFT}$ are the DFT total energy for the amino acid + monomer complex. Therefore, more favorable binding occurs with more negative values of ΔE. The table with all explicit DFT data is available in the SM file (Table SM1). Below, Figure 6 shows the relationship interaction of each FM with the respective AA, highlighting the difference in the binding energy in kcal mol
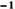


Firstly, the selected specific region of the HPK protein resembling the active site of the human PSA does not encompass all 20 AA. For this reason, the predominant AA in the selected region includes cysteine (CYS), glutamine (GLN), glycine (GLY), glutamate (GLU), lysine (LYS), tryptophan (TRP), valine (VAL), and isoleucine (ILE). Although the majority of the evaluated monomer‐AA complexes exhibit negative binding energies, it is crucial to determine which complex demonstrates the most favorable binding with all the AA present in this region. Accordingly, as shown in Figure 6, the FMs with the lowest ΔE energies are itaco, 4imid, and acidm, with acidm and acida being the most commonly employed FMs in MIP syntheses [57].

Among the eight AA in the selected binding region, GLY (−13.08 kcal mol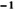) and TRP (−10.00 kcal mol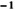) exhibited the strongest interactions with itaco, followed by CYS (−10.50 kcal mol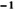) and GLN (−10.38 kcal mol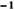). These results position itaco as a particularly promising FM candidate. For example, the itaco‐MET complex features a binding energy of −14.08 kcal mol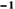 and forms two hydrogen bonds, suggesting substantial stability compared to other complexes (Figure SM38).

Additionally, another key mechanistic feature observed in these interactions is the proton transfer from the FM carboxyl group to the amine nitrogen of AA. This transfer enhances the binding strength by increasing the positive charge on nitrogen and the negative charge on oxygen, reinforcing electrostatic interactions. Although this proton transfer is likely reversible, the resulting complex remains stable through resonance stabilization of the carboxylate group. This reversibility is crucial for the eventual extraction of the template molecule from the polymer, facilitating the formation of selective recognition cavities in the polymer matrix.

The results also show that the interaction between the intermediate FM acida and GLY is significant, particularly in the active site region. For example, the most stable acida‐GLY complex features two hydrogen bonds, forming a well‐symmetric structure with an energy of −10.51 kcal mol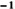 (Figure SM38). In contrast, the most stable itaco‐GLY complex has a single hydrogen bond but a lower energy of −13.64 kcal mol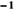 (Figure SM38). Another relevant complex is acida‐GLN, as GLN is also part of the relevant region in the protein. The most stable acida‐GLN complex, which has a moderate energy of −5.53 kcal mol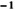, and is stabilized by a hydrogen bond (Figure SM38). Although this energy is less favorable, the intermediate FM acida remains a good candidate because the relatively weak interaction may facilitate the removal of the PSA template post‐polymerization, supporting acida's utility as an FM candidate.

The FM 4imid showed uniformly favorable interactions with the eight AAs in the PSA binding site region, making it a significant candidate for our analysis. Conversely, acidm exhibited particularly strong binding with ARG, yielding a binding energy of −18.38 kcal mol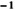 for complex number 4 (Figure SM38), the strongest interaction among all evaluated complexes between an FM and AA. Other FMs, including 1ally, 2viny, and acida, showed moderate to strong interactions, suggesting their suitability as intermediate candidates.

It is also noteworthy that 1ally displayed strong binding with AAs such as PRO and CYS, with binding energies of approximately −16.91 kcal mol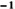 and −16.82 kcal mol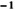, respectively; with SER (−14.44 kcal mol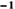); and HIS (−8.21 kcal mol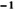). Among these, the selected binding site region of the protein. Therefore, while 1ally may not be the best candidate for this specific PSA‐targeted application, it remains a promising candidate for MIPs targeting other proteins that contain these AAs in their accessible regions. Since this study focuses on the interaction between FM and the AA, the dataset could also be applied for use in MIPs designed for various biological macromolecules.

When comparing the two structural monomers (14dvb and bisac), the preferred monomer choice for synthesis is the one with weaker interaction energies with the AAs. This minimizes competitive binding with the FM and helps preserve the structural integrity of the final polymer [28]. For example, in the 14dvb‐GLY complex, the interaction involves π‐orbital stacking from the aromatic ring, with a binding energy of −4.40 kcal mol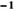 for the most stable configuration (complex 7). Conversely, the bisac‐GLY complex shows a stronger interaction with binding energy of −6.60 kcal mol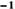 (complex 8), supported by a hydrogen bond (Figure SM38). These findings support the selection of 14dvb as the preferred structural monomer, as it is less likely to interfere with FM‐AA binding while still contributing to the rigidity of the imprinted polymer. Additional information is provided in Figures SM39 to SM41.

#### 3.2.2 Electronic and Structural Analysis: Evaluation of Frontier Orbitals and Geometric Parameters

The analysis of frontier molecular orbitals (FMOs), specifically the Highest Occupied Molecular Orbital (HOMO) and Lowest Unoccupied Molecular Orbital (LUMO), along with the corresponding HOMO‐LUMO gap, is presented herein. These parameters are crucial for evaluating the electronic properties and chemical reactivity of molecules. The HOMO reflects the electron‐donating ability of the molecule, whereas LUMO represents its electron‐accepting capacity. The HOMO‐LUMO energy gap is an important indicator of both kinetic stability and overall molecular reactivity [58, 59, 60].

For the acidm‐GLY complex, the calculated HOMO and LUMO energies were $E_{\text{HOMO‐acidm}}=-7.52\;\text{eV}$ and $E_{\text{LUMO‐acidm}}=-1.52\;\text{eV}$, respectively. The LUMO of acidm, dominated by hydroxyl contributions, facilitates hydrogen bonding with the HOMO of GLY, whose energy is $E_{\text{HOMO‐GLY}}=-6.94\;\text{eV}$. This orbital alignment contributes to the observed favorable binding energy of $-11.46\;\text{kcal}\;{\text{mol}}^{-1}$ (Figure SM42).

In contrast, the 4imid‐Gly complex exhibited a weaker binding energy of $-3.37\;\text{kcal}\;{\text{mol}}^{-1}$. The higher HOMO energy of 4imid ($-6.30\;\text{kcal}\;{\text{mol}}^{-1}$) compared to GLY ($-6.94\;\text{kcal}\;{\text{mol}}^{-1}$) indicates a reduced electron donation capacity, as shown in Figure SM42. According to FMO theory, a higher HOMO energy indicates increased electron donation potential. However, in this context, the smaller energy difference between the HOMO of 4imid and the LUMO of glycine results in reduced interaction strength, explaining the less favorable binding energy. These observations demonstrate the utility of FMO theory in rationalizing molecular interaction trends and predicting relative stability and reactivity.

Beyond molecular orbital analyses, structural changes were observed in isolated molecules upon complex formation. For example, in the isolated methacrylic acid molecule, the bond length of the carbonyl group (C=O) is 1.35 Å, while the hydroxyl O—H bond length measures 0.97 Å (Figure SM43). Additionally, the angle between the hydroxyl group and the carbonyl group of the carboxylic moiety is 108.9°. Upon interaction with GLY, forming the most stable complex, these structural parameters undergo significant changes. The C=O bond length reduces to 1.32 Å, while the O—H bond length increases to 1.05 Å. The internal angle between these groups expands to 111.93°. These distortions indicate electronic and spatial reorganization due to complexation.

Such distortions in bond lengths and angles can be attributed to electrostatic interactions and hydrogen bonding between the carboxyl group of methacrylic acid and the amino and carboxyl groups of glycine. These non‐covalent interactions contribute to the overall stabilization of the complex. In light of these observations, a more detailed analysis is required to further explore the bonding characteristics and electronic redistribution mechanisms responsible for stabilizing the complex. These aspects are explored in the following subsection.

#### 3.2.3 Topological Analysis: Evaluating the Nature of Chemical Interaction on Amino Acids‐Monomer Complexes

This section evaluates the nature of chemical interactions in amino acid‐functional monomer complexes using the Quantum Theory of Atoms in Molecules (QTAIM). Briefly, QTAIM enables the characterization of chemical bonding through the topological analysis of the electron density distribution, revealing insights into bond strength, nature (covalent and non‐covalent), and stability—key factors that are critical for understanding and enhancing MIP selectivity.

In QTAIM, critical points of the electron density are classified by their rank and signature. Relevant types may include nuclear critical points NCP(3, −3), bond critical points BCP(3, −1), ring critical points RCP(3, +1), and cage critical points CCP(3, +3) [61, 62, 63]. The analysis in this work focuses on BCPs, where electron density $\rho_{b}$ and its Laplacian $\nabla^{2}\rho_{b}$ serve as key descriptors of bonding interactions.

For hydrogen bonds, accepted values of $\rho_{b}$ between 0.002–0.034 a.u. and $\nabla^{2}\rho_{b}$ between 0.024–0.139 a.u. [61]. These values indicate closed‐shell interactions typical of hydrogen bonding and vdW interactions.

Additionally, the electronic energy density H(r) at the BCP is defined as follows:

(2) H(r)=G(r)+V(r)

where, *G*(r) is the kinetic energy density and *V*(r) is the potential energy density. A negative *H*(r) typically suggests partial covalent character, whereas a positive *H*(r) supports electrostatic (non‐covalent) interactions.

QTAIM classifies chemical interactions based on the values of the electron density topological properties, as follows: If $\nabla^{2}\rho (r)>0$ and H(r)>0, the interaction is considered weak and electrostatic; if $\nabla^{2}\rho (r)<0$ and H(r)<0, the interaction is considered strong and covalent bond; and if $\nabla^{2}\rho (r)>0$ and H(r)<0, the interaction is moderate with partial covalent characteristics [61, 64].

In addition to QTAIM, the Electron Localization Function (ELF) provides complementary insights into bonding characteristics. ELF values range from 0 to 1, where values near 0 indicate low localization, typically associated with weak interactions near zero, while values near 1 suggest high localization, which may be indicative of covalent bonding. Therefore, ELF analysis can also be used to evaluate the strength and nature of hydrogen bonds within a complex. All wavefunction analyses were performed using Multiwfn v.3.7. The Poincaré‐Hopf relationship was satisfied for all monomer‐analyte complexes, ensuring topological completeness of the QTAIM analyses.

The analyzed monomer‐analyte complexes exhibit hydrogen bonds of varying strengths, as summarized in Table 1. Insights derived from electronic structure descriptors, particularly the ELF and the QTAIM parameters, provide valuable information on the nature and stability of these bonding interactions. For instance, for weak interactions, as on 4imid‐GLU complex (17H‐26N, CP 43) exhibits H(r)=−0.001 a.u., ELF of 0.151, and bond length of 1.89 Å. Here, the significantly lower ELF value and the small negative energy density indicate a weak, electrostatically dominated interaction. This indicates a more diffuse electron density at the bond critical point (BCP), which is characteristic of the weaker, non‐covalent hydrogen bond.

**TABLE 1:** Topological, electronic and structural properties for the selected bond critical points (BCP) in the monomer (A)‐amino acid (B) complexes.

Complexes	A‐B	H(r)	ELF	$D_{\text{bond}}$	ρ(r)	$\nabla^{2}\rho (r)$
		(a.u.)	(‐)	(Ã…)	(a.u.)	(a.u.)
itaco‐gly	21H‐4N	−0.007	0.267	1.61	0.055	0.124
4imid‐glu	17H‐26N	−0.001	0.151	1.89	0.035	0.095
4imid‐glu	6H‐22O	−0.005	0.047	2.12	0.017	0.059
acidm‐gly	3H‐16O	0.001	0.027	2.91	0.010	0.038
acidm‐gly	22H‐4N	−0.006	0.258	1.56	0.052	0.119
acidm‐lys	36H‐1N	−0.007	0.269	1.71	0.055	0.125
acida‐gly	10H‐18O	−40.002	0.179	1.65	0.050	0.149
acida‐gly	19H‐8O	−0.001	0.171	1.67	0.046	0.143

*Note:* The presented properties are electronic energy density, H(r); electron localization function, ELF; bond distance, $D_{bond}$; electron density, ρ(r); and Laplacian of electron density, $\nabla^{2}\rho (r)$.

In contrast, moderate‐strength interactions are observed in complexes such as itaco‐GLY (21H‐4N, CP 44) with H(r)=0.007 a.u., ELF of 0.267, and a bond length of 1.61 Å. Here, the negative H(r) and the relatively elevated ELF suggest a stabilizing hydrogen bond, where the electron density distribution is more localized and partial covalent character at the BCP, reinforcing the interaction. Similarly, the acidm‐LYS complex (36H‐1N, CP 43) exhibits values of H(r)=−0.007 a.u., ELF of 0.269, and a bond length of 1.71 Å. These parameters also indicate a moderate hydrogen bond with appreciable covalent contributions, due to the more localized electron density. Similarly, the acida‐GLY complex (19H‐8O, CP 22) presents a weaker interaction, with *H*(r)=−0.001 a.u., ELF of 0.171, and a bond length of 1.67 Å. Although the lower ELF and H(r) values point to a less localized interaction than the previous cases, this interaction contributes moderately to the stabilization, through a combination of both electrostatic and minor covalent effects.

Overall, the QTAIM parameters and ELF analysis collectively reveal that the strength and nature of the hydrogen bonds formed in these monomer‐analyte complexes range from weak, predominantly electrostatically driven interactions, to moderate, stabilizing bonds with partial covalent character. These insights are detailed in Table 1, where the full set of calculated parameters for each complex is shown.

### 3.3 Evaluating PSA‐Monomers Interactions Through Molecular Docking

Although molecular dynamics (MD) simulations have been successfully employed in our previous studies involving MIPs [16], we opted for molecular docking in the present work to facilitate the rapid and systematic screening of 15 candidate FMs. While MD simulations provide detailed insights into molecular interactions, they are computationally expensive and time‐consuming. In contrast, molecular docking offers a computationally efficient approach [65], enabling fast estimation of interaction tendencies and making it ideally suited for the preliminary selection of promising monomers for further study.

Moreover, molecular docking shares conceptual similarities with the MIP imprinting process, as both are based on principles of molecular complementarity and pre‐organization. In docking, the ligand aligns with the receptor in a manner that maximizes binding affinity and interaction complementarity, closely resembling the “lock‐and‐key” model [66, 67, 68]. This mechanism parallels the MIP approach, wherein monomers are selected to complement the size, shape, and functional groups of the target molecule [67, 68]

In this study, molecular docking was performed using the FMs, that is, acida, itaco, 4imid, and acidm as flexible ligands, while the receptor active site region of the PSA protein is composed of the following eight AA: CYS, GLN, GLY, GLU, LYS, TRP, VAL, and ILE (Figures SM46 to SM56). Our previous DFT analyses have elucidated the stabilizing nature of FM‐AA interactions, where hydrogen bonds play a crucial role. However, docking results revealed that multi‐residue interactions could introduce additional destabilizing or stabilizing effects due to the formation of multiple hydrogen bonds as well as hydrophobic interactions.

For instance, in the case of acidm, hydrogen bonds were observed with ILE16 and GLY19, while hydrophobic interactions were predominant with ILE17, GLY18, and GLN156, as shown in Table SM2. For all eight AA included in the active site, the calculated binding free energy (ΔG) for acidm is approximately −3.00 kcal mol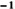, one of the most favorable values among all studied FMs. FM 4imid also formed stabilizing interactions with AA GLY18, GLY19, and GLN156, through hydrogen bonds, and hydrophobic interactions with ILE16, ILE17, and GLU21. However, acidm exhibited a slightly more favorable ΔG, DFT results (Figure 6) indicate that 4imid generally provides stronger and more consistent interactions with AAs, reinforcing its suitability as a promising FM.

**FIGURE 7:**
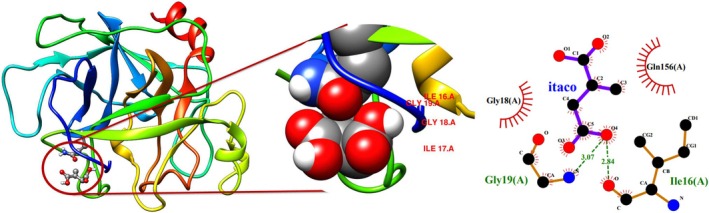
Depiction of the 3D and 2D molecular affinity of itaconic acid with the amino acids present in the strategic region of the PSA protein. The 3D structure includes a ribbon representation, illustrating the conformation of each protease peptide [69], while the 2D interactions were analyzed using LIGPLOT+ software [70]. Atom colors are as follows: Carbon (black), nitrogen (blue), and oxygen (red), with hydrogen atoms omitted for clarity. The binding free energy (ΔG) for itaconic acid is −3.40 kcal mol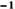, as detailed in Table SM2.

It is crucial to recognize that the overall binding strength of a given FM increases as additional favorable interactions accumulate. Specifically, FM itaco, identified in DFT analysis as the second‐strongest interaction, interacts with four AAs via docking, two of them by hydrogen bonding, while the other two through hydrophobic interactions, as shown in Figure 7. The hydrogen bonds are formed with GLY18, GLY19, and ILE16, and hydrophobic interactions occur with GLN156 and ILE17. The ΔG for itaco is −3.40 kcal mol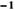, indicating a strong and stable interaction in this region. Hydrogen bonds lenghts typically range from 2.7 Å to 3.0 Å [71], with values around 3.0 Å being the most common in biological systems. The hydrogen bond lengths observed in the itaco complex fall within this expected range, further confirming the validity of the interaction model.

In the case of FM acida, docking revealed three hydrogen bonds with three different AA, that is, GLY18, GLY19, and ILE16, which significantly contribute to stabilizing the interaction. Additionally, hydrophobic interactions with ILE17 and GLN156 are observed. The ΔG of −2.50 kcal mol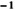 (Table SM2) supports its classification as good, but intermediate FM, which is consistent with the previous DFT prediction.

Finally, FM 1allyl, which slightly outperforms acida according to DFT analysis, exhibits a ΔG of −2.60 kcal mol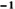 in molecular docking simulations. This close agreement between DFT and docking data enhances the reliability of the theoretical methodologies employed in this study to predict favorable FM‐AA interactions within the PSA active site.

## 4 Conclusion

In this study, a novel algorithm, the Molecular Binding Algorithm by Surface Mapping (MBASM), was developed to systematically generate amino acid‐monomer complexes, followed by structural optimization using Density Functional theory (DFT). This integrated MBASM + DFT protocol was designed to identify the optimal functional monomer for the synthesis of molecularly imprinted polymer (MIP) targeting prostate‐specific antigen (PSA). The MBASM + DFT protocol streamlined the selection process, reducing thousands of potential structures to a manageable set of 2400, which were then subjected to detailed DFT analysis.

Preliminary benchmarking demonstrated the effectiveness of MBASM compared to alternative approaches. However, ongoing development and refinement of the algorithm are planned to further improve its accuracy, scalability, and applicability to other complex molecular systems.

From the combined DFT and molecular docking analyses, promising functional monomers for MIP synthesis were identified, including itaconic acid, 4‐imidazole acrylic acid, and methacrylic acid. Additionally, 1,4‐divinylbenzene was identified as a superior structural monomer (cross‐linker) based on its favorable interaction profile and minimal interference with FM‐template interactions.

Furthermore, the most promising stable FM‐AA complexes were found to be stabilized by hydrogen bonding interactions, which range in strength from weak to moderate, as confirmed by QTAIM and ELF analyses. These non‐covalent interactions play a key role in molecular recognition and contribute to the selective imprinting behavior of MIPs.

Overall, the MBASM + DFT novel framework offers a powerful and scalable approach for the rational design of MIP systems. This refined methodology has the potential to accelerate the development of targeted MIPs for biorecognition and diagnostic applications.

## Conflicts of Interest

The authors declare no conflicts of interest.

## Supporting information


**Data S1.** Supporting Information.

## Data Availability

The data that support the findings of this study are available from the corresponding author upon reasonable request.

## References

[1] G. Ertürk , M. Hedström , M. A. Tümer , A. Denizli , and B. Mattiasson , “Real‐Time Prostate‐Specific Antigen Detection With Prostate‐Specific Antigen Imprinted Capacitive Biosensors,” Analytica Chimica Acta 891 (2015): 120. 26388370 10.1016/j.aca.2015.07.055

[2] C. Dejous and U. M. Krishnan , “Sensors for Diagnosis of Prostate Cancer: Looking Beyond the Prostate Specific Antigen,” Biosensors and Bioelectronics 173 (2021): 112790. 33190047 10.1016/j.bios.2020.112790

[3] Z. Yazdani , H. Yadegari , and H. Heli , “A Molecularly Imprinted Electrochemical Nanobiosensor for Prostate Specific Antigen Determination,” Analytical Biochemistry 566 (2019): 116. 30472220 10.1016/j.ab.2018.11.020

[4] S. M. Traynor , R. Pandey , R. Maclachlan , et al., “Review—Recent Advances in Electrochemical Detection of Prostate Specific Antigen (PSA) in Clinically‐Relevant Samples,” Journal of the Electrochemical Society 167 (2020): 037551.

[5] S. P. Balk , Y.‐J. Ko , and G. J. Bubley , “Biology of Prostate‐Specific Antigen,” Journal of Clinical Oncology 21 (2003): 383. 12525533 10.1200/JCO.2003.02.083

[6] R. J. Babaian , D. A. Johnston , W. Naccarato , A. Ayala , V. A. Bhadkamkar , and H. A. Fritsche , “The Incidence of Prostate Cancer in a Screening Population With a Serum Prostate Specific Antigen Between 2.5 and 4.0 Ng/mL: Relation to Biopsy Stragegy,” Journal of Urology 165 (2001): 757. 11176461

[7] X.‐H. Pham , E. Hahm , K.‐H. Huynh , B. S. Son , H.‐M. Kim , and B.‐H. Jun , “Sensitive Colorimetric Detection of Prostate Specific Antigen Using a Peroxidase‐Mimicking Anti‐PSA Antibody Coated Au Nanoparticle,” BioChip Journal 14 (2020): 158.

[8] A. Jedinak , K. R. Loughlin , and M. A. Moses , “Approaches to the Discovery of Non‐Invasive Urinary Biomarkers of Prostate Cancer,” Oncotarget 9 (2018): 3253432550. 10.18632/oncotarget.25946 PMC6126692 30197761

[9] E. Jara‐Cornejo , S. Khan , J. Vega‐Chacn , et al., “Biomimetic Material for Quantification of Methotrexate Using Sensor Based on Molecularly Imprinted Polypyrrole Film and Mwcnt/Gce,” Biomimetics 8 (2023): 77. 36810408 10.3390/biomimetics8010077 PMC9944472

[10] Z. Mazouz , M. Mokni , N. Fourati , et al., “Computational Approach and Electrochemical Measurements for Protein Detection With Mip‐Based Sensor,” Biosensors and Bioelectronics 151 (2020): 111978. 31999585 10.1016/j.bios.2019.111978

[11] S. Wang , L. Zhang , J. Zeng , et al., “Multi‐Templates Molecularly Imprinted Polymers for Simultaneous Recognition of Multiple Targets: From Academy to Application,” TrAC Trends in Analytical Chemistry 166 (2023): 117173.

[12] R. C. Costa , B. E. Nagay , J. E. L. Villa , et al., “Pathogenesis‐Guided Engineering: Ph‐Responsive Imprinted Polymer Co‐Delivering Folate for Inflammation‐Resolving as Immunotherapy in Implant‐Related Infections,” Advanced Functional Materials 34 (2024): 2406640.

[13] J. E. L. Villa , S. Khan , L. C. S. Neres , and M. D. P. T. Sotomayor , “Preparation of a Magnetic Molecularly Imprinted Polymer for Non‐Invasive Determination of Cortisol,” Journal of Polymer Research 28 (2021): 298.

[14] F. A. Trikka , K. Yoshimatsu , L. Ye , and D. A. Kyriakidis , “Molecularly Imprinted Polymers for Histamine Recognition in Aqueous Environment,” Amino Acids 43 (2012): 21132124. 10.1007/s00726-012-1297-8 22526245

[15] L. Abbasy , A. Mohammadzadeh , M. Hasanzadeh , and N. Razmi , “Development of a Reliable Bioanalytical Method Based on Prostate Specific Antigen Trapping on the Cavity of Molecular Imprinted Polymer Towards Sensing of PSA Using Binding Affinity of PSA‐MIP Receptor: A Novel Biosensor,” Journal of Pharmaceutical and Biomedical Analysis 188 (2020): 113447. 32623317 10.1016/j.jpba.2020.113447

[16] L. C. S. Neres , G. T. Feliciano , R. F. Dutra , and M. D. P. T. Sotomayor , “Development of a Selective Molecularly Imprinted Polymer for Troponin T Detection: A Theoretical‐Experimental Approach,” Materials Today Communications 30 (2022): 102996.

[17] R. Bujak , R. Gadzaa‐Kopciuch , A. Nowaczyk , et al., “Selective Determination of Cocaine and Its Metabolite Benzoylecgonine in Environmental Samples by Newly Developed Sorbent Materials,” Talanta 146 (2016): 401409. 10.1016/j.talanta.2015.08.066 26695282

[18] T. A. Sales , L. V. F. Ferreira , A. G. Nogueira , and T. C. Ramalho , “A Theoretical Protocol for the Rational Design of the Bioinspired Multifunctional Hybrid Material Mip@Cercosporin,” Journal of Molecular Modeling 29 (2023): 321. 37725158 10.1007/s00894-023-05653-x

[19] C. R. T. Tarley , M. D. P. T. Sotomayor , and L. T. Kubota , “Polímeros Biomiméticos em Química Analítica. Parte 1: Preparo e Aplicações de MIP (“Molecularly Imprinted Polymers”) em Técnicas de Extração e Separação,” Química Nova 28 (2005): 1076.

[20] E. Mohsenzadeh , V. Ratautaite , E. Brazys , et al., “Application of Computational Methods in the Design of Molecularly Imprinted Polymers (Review),” TrAC Trends in Analytical Chemistry 171 (2024): 117480.

[21] A. K. Singh and M. Singh , “Designing l‐Serine Targeted Molecularly Imprinted Polymer via Theoretical Investigation,” Journal of Theoretical and Computational Chemistry 15 (2016): 1650041.

[22] A. Nezhadali and M. Mojarrab , “Computational Design and Multivariate Optimization of an Electrochemical Metoprolol Sensor Based on Molecular Imprinting in Combination With Carbon Nanotubes,” Analytica Chimica Acta 924 (2016): 86. 27181648 10.1016/j.aca.2016.04.017

[23] J. J. BelBruno , “Molecularly Imprinted Polymers,” Chemical Reviews 119 (2018): 94. 30246529 10.1021/acs.chemrev.8b00171

[24] C. F. Silva , L. F. Menezes , A. C. Pereira , and C. S. Nascimento , “Molecularly Imprinted Polymer (MIP) for Thiamethoxam: A Theoretical and Experimental Study,” Journal of Molecular Structure 1231 (2021): 129980.

[25] Y. Sun , Y. Gu , and Y. Jiang , “Adsorption Behavior of a Tri‐Functionalized Imprinted Resin With High Selectivity for 5‐Sulfosalicylic Acid: Batch Experiments and DFT Calculation,” Journal of Hazardous Materials 412 (2021): 125271. 33548783 10.1016/j.jhazmat.2021.125271

[26] L. Xie , N. Xiao , L. Li , X. Xie , and Y. Li , “Theoretical Insight Into the Interaction Between Chloramphenicol and Functional Monomer (Methacrylic Acid) in Molecularly Imprinted Polymers,” International Journal of Molecular Sciences 21 (2020): 4139. 32532004 10.3390/ijms21114139 PMC7312358

[27] W. Dong , M. Yan , Z. Liu , G. Wu , and Y. Li , “Effects of Solvents on the Adsorption Selectivity of Molecularly Imprinted Polymers: Molecular Simulation and Experimental Validation,” Separation and Purification Technology 53 (2007): 183.

[28] D. Mukasa , M. Wang , J. Min , et al., “A Computationally Assisted Approach for Designing Wearable Biosensors Toward Non‐Invasive Personalized Molecular Analysis,” Advanced Materials 35 (2023): 2212161. 10.1002/adma.202212161 PMC10529901 37159949

[29] D. S. Sholl and J. A. Steckel , Density Functional Theory: A Practical Introduction (John Wiley & Sons, 2011).

[30] J. Barhen , V. Protopopescu , and D. Reister , “TRUST: A Deterministic Algorithm for Global Optimization,” Science 276 (1997): 1094.

[31] D. Sculley , Web‐Scale k‐Means Clustering, in Proceedings of the 19th International Conference on World Wide Web ‐ WWW 10 (ACM Press, 2010).

[32] J. MacQueen , “Classification and Analysis of Multivariate Observations, in 5th Berkeley Symp,” Mathematical Statistics Probability (1967): 281–297.

[33] G. Hamerly and C. Elkan , “Learning the k in k‐Means,” Advances in Neural Information Processing Systems 16 (2003).

[34] F. Orlando Morais , K. F. Andriani , and J. L. F. Da Silva , “Investigation of the Stability Mechanisms of Eight‐Atom Binary Metal Clusters Using DFT Calculations and k‐Means Clustering Algorithm,” Journal of Chemical Information and Modeling 61 (2021): 34113420. 10.1021/acs.jcim.1c00253 34161078

[35] K. F. Andriani , P. Felicio‐Sousa , F. O. Morais , and J. L. F. Da Silva , “Role of Quantum‐Size Effects in the Dehydrogenation of CH4 on 3d TMn Clusters: DFT Calculations Combined With Data Mining,” Catalysis Science & Technology 12 (2022): 916.

[36] R. Bühler , M. Schütz , K. F. Andriani , et al., “A Living Library Concept to Capture the Dynamics and Reactivity of Mixed‐Metal Clusters for Catalysis,” Nature Chemistry 17 (2025): 525. 10.1038/s41557-024-01726-3 PMC11964927 39849109

[37] Deserno , accessed March 2022, 30, https://www.cmu.edu/biolphys/deserno/pdf/sphere_equi.pdf.

[38] A. Yershova , S. Jain , S. M. LaValle , and J. C. Mitchell , “Generating Uniform Incremental Grids on So(3) Using the Hopf Fibration,” International Journal of Robotics Research 29 (2009): 801. 10.1177/0278364909352700 PMC2896220 20607113

[39] J. C. Mitchell , “Sampling Rotation Groups by Successive Orthogonal Images,” SIAM Journal on Scientific Computing 30 (2008): 525.

[40] L. Van der Maaten and G. Hinton , “Visualizing Data Using t‐SNE,” Journal of Machine Learning Research 9 (2008): 2579.

[41] C. Lee , W. Yang , and R. G. Parr , “Development of the Colle‐Salvetti Correlation‐Energy Formula Into a Functional of the Electron Density,” Physical Review B 37 (1988): 785. 10.1103/physrevb.37.785 9944570

[42] F. Neese , “Software Update: The Orca Program System, Version 4.0, Wiley Interdisciplinary Reviews: Computational Molecular,” Science 8 (2018): e1327.

[43] F. Neese , “Orca, an Ab Initio, DFT and Semiempirical Electronic Structure Package 3,” 2009.

[44] F. Weigend and R. Ahlrichs , “Balanced Basis Sets of Split Valence, Triple Zeta Valence and Quadruple Zeta Valence Quality for h to Rn: Design and Assessment of Accuracy,” Physical Chemistry Chemical Physics 7 (2005): 3297. 16240044 10.1039/b508541a

[45] Y. Takano and K. N. Houk , “Benchmarking the Conductor‐Like Polarizable Continuum Model (CPCM) for Aqueous Solvation Free Energies of Neutral and Ionic Organic Molecules,” Journal of Chemical Theory and Computation 1 (2004): 70. 10.1021/ct049977a 26641117

[46] O. Trott and A. J. Olson , “AutoDock Vina: Improving the Speed and Accuracy of Docking With a New Scoring Function, Efficient Optimization, and Multithreading,” Journal of Computational Chemistry 31 (2009): 455. 10.1002/jcc.21334 PMC3041641 19499576

[47] G. M. Morris , R. Huey , W. Lindstrom , et al., “AutoDock4 and AutoDockTools4: Automated Docking With Selective Receptor Flexibility,” Journal of Computational Chemistry 30 (2009): 2785. 19399780 10.1002/jcc.21256 PMC2760638

[48] H. M. Berman , T. Battistuz , T. N. Bhat , et al., “The Protein Data Bank,” Acta Crystallographica Section D: Biological Crystallography 58 (2002): 899. 12037327 10.1107/s0907444902003451

[49] A. L. Carvalho , L. Sanz , D. Barettino , A. Romero , J. J. Calvete , and M. J. Romão , “Crystal Structure of a Prostate Kallikrein Isolated From Stallion Seminal Plasma: A Homologue of Human PSA,” Journal of Molecular Biology 322 (2002): 325. 12217694 10.1016/s0022-2836(02)00705-2

[50] W. Humphrey , A. Dalke , and K. Schulten , “VMD – Visual Molecular Dynamics,” Journal of Molecular Graphics 14 (1996): 33. 8744570 10.1016/0263-7855(96)00018-5

[51] W. R. Pearson and D. J. Lipman , “Improved Tools for Biological Sequence Comparison,” in Proceedings of the National Academy of Sciences, vol. 85 (1988), 24442448. 10.1073/pnas.85.8.2444 PMC280013 3162770

[52] S. F. Altschul , W. Gish , W. Miller , E. W. Myers , and D. J. Lipman , “Basic Local Alignment Search Tool,” Journal of Molecular Biology 215 (1990): 403410. 10.1016/S0022-2836(05)80360-2 2231712

[53] X. Wang , G. Chen , P. Zhang , and Q. Jia , “Advances in Epitope Molecularly Imprinted Polymers for Protein Detection: A Review,” Analytical Methods 13 (2021): 16601671. 10.1039/d1ay00067e 33861232

[54] S. Dietl , H. Sobek , and B. Mizaikoff , “Epitope‐Imprinted Polymers for Biomacromolecules: Recent Strategies, Future Challenges and Selected Applications,” TrAC Trends in Analytical Chemistry 143 (2021): 116414.

[55] T. Khumsap , A. Corpuz , and L. T. Nguyen , “Epitope‐Imprinted Polymers: Applications in Protein Recognition and Separation,” RSC Advances 11 (2021): 1140311414. 10.1039/d0ra10742e PMC8695941 35423617

[56] P. Pracht , S. Grimme , C. Bannwarth , et al., “Crest ‐ A Program for the Exploration of Low‐Energy Molecular Chemical Space,” Journal of Chemical Physics 160 (2024): 114110. 38511658 10.1063/5.0197592

[57] K. Nishchaya , V. K. Rai , and H. Bansode , “Methacrylic Acid as a Potential Monomer for Molecular Imprinting: A Review of Recent Advances,” Results in Materials 18 (2023): 100379.

[58] A. Rahman , M. M. Hoque , M. A. K. Khan , M. G. Sarwar , and M. A. Halim , “Non‐Covalent Interactions Involving Halogenated Derivatives of Capecitabine and Thymidylate Synthase: A Computational Approach,” SpringerPlus 5 (2016): 146. 27026843 10.1186/s40064-016-1844-y PMC4764604

[59] S. Janani , H. Rajagopal , S. Muthu , S. Aayisha , and M. Raja , “Molecular Structure, Spectroscopic (Ft‐Ir, Ft‐Raman, Nmr), Homo‐Lumo, Chemical Reactivity, AIM, ELF, LOL and Molecular Docking Studies on 1‐Benzyl‐4‐(n‐Boc‐Amino)piperidine,” Journal of Molecular Structure 1230 (2021): 129657.

[60] P. Atkins , Shriver and Atkins' Inorganic Chemistry (Oxford University Press, 2010).

[61] F. Wang , H. Du , H. Liu , and X. Gong , “Hydrogenbonding Interactions and Properties of Energetic Nitroamino[1, 3, 5]Triazinebased Guanidinium Salts: Dftd and Qtaim Studies,” Chemistry an Asian Journal 7 (2012): 25772591. 10.1002/asia.201200450 22945691

[62] T. Lu and F. Chen , “Multiwfn: A Multifunctional Wavefunction Analyzer,” Journal of Computational Chemistry 33 (2012): 580. 22162017 10.1002/jcc.22885

[63] M. Jaboski and M. Palusiak , “The Halogenoxygen Interaction in 3‐Halogenopropenal Revisited the Dimer Model vs. Qtaim Indications,” Chemical Physics 415 (2013): 207.

[64] U. Gunawan , S. Ibrahim , A. Luqman Ivansyah , and S. Damayanti , “Insights Into the Selective Imprinted Polymer of Voriconazole From Host‐Guest Interaction Perspective,” Journal of Molecular Liquids 383 (2023): 122130.

[65] K. F. Andriani , G. Heinzelmann , and G. F. Caramori , “Shedding Light on the Hydrolysis Mechanism of Cis, Trans‐[Ru(dmso)4Cl2] Complexes and Their Interactions With DNA ‐ A Computational Perspective,” Journal of Physical Chemistry B 123 (2018): 457467. 10.1021/acs.jpcb.8b11287 30576133

[66] J. Fan , A. Fu , and L. Zhang , “Progress in Molecular Docking,” Quantitative Biology 7 (2019): 8389.

[67] E. K. Reville , E. H. Sylvester , S. J. Benware , S. S. Negi , and E. B. Berda , “Customizable Molecular Recognition: Advancements in Design, Synthesis, and Application of Molecularly Imprinted Polymers,” Polymer Chemistry 13 (2022): 3387.

[68] Y. Liu , L. Wang , H. Li , et al., “Rigorous Recognition Mode Analysis of Molecularly Imprinted Polymersrational Design, Challenges, and Opportunities,” Progress in Polymer Science 150 (2024): 101790.

[69] E. F. Pettersen , T. D. Goddard , C. C. Huang , et al., “UCSF Chimera ‐ A Visualization System for Exploratory Research and Analysis,” Journal of Computational Chemistry 25 (2004): 1605. 15264254 10.1002/jcc.20084

[70] R. A. Laskowski and M. B. Swindells , “Ligplot+: Multiple Ligand–Protein Interaction Diagrams for Drug Discovery,” Journal of Chemical Information and Modeling 51 (2011): 2778. 21919503 10.1021/ci200227u

[71] T. K. Harris and A. S. Mildvan , “High‐Precision Measurement of Hydrogen Bond Lengths in Proteins by Nuclear Magnetic Resonance Methods, Proteins: Structure,” Function, and Genetics 35 (1999): 275. 10.1002/(sici)1097-0134(19990515)35:3<275::aid-prot1>3.0.co;2-v 10328262

