# Supplementary material for: Exploring Monomer‐Amino Acid Interactions in Mimicking Mips for PSA Detection—Using the Novel MBASM Approach
Source: J Comput Chem. 2025 May 21;46(14):e70139. doi: 10.1002/jcc.70139 (PMC12093453; doi:10.1002/jcc.70139)
Supplement: Supplementary file 1 — Data S1. Supporting Information. [file JCC-46-0-s001.pdf]

Supplementary Material: Exploring Monomer-Amino Acid Interactions in Mimeticking MIPs for PSA Detection - Using the Novel MBASM Approach

Lariel Chagas da Silva Neres,[†] Johnatan Mucelini,[‡] Gabriel Augusto Pinheiro,[¶] Helen Luiza Brandão Silva Ambrósio,[§] Albérico Borges Ferreira da Silva,[‡] Maria Del Pilar Taboada Sotomayor,[†] and Karla Furtado Andriani[*,§]

†*Institute of Chemistry, State University of São Paulo (UNESP), 14801-970 Araraquara, SP, Brazil*

‡*São Carlos Chemistry Institute - University of São Paulo (USP), Avenida Trabalhador São-Carlense, 400 - Parque Arnold Schmidt, SP, Brazil*

¶*Institute of Science and Technology, Federal University of São Paulo, São José dos Campos, SP, Brazil*

§*Departament of Exact Sciences, State University of Santa Cruz, P.O. Box* 45662-900*, Ilhéus, BA, Brazil*

E-mail: kfandriani@uesc.br

Contents

1 Introduction **SM-2**

2 Infrared (IR) spectrum **SM-3**

2.1 IR spectrum for the aminoacids (AA) SM-3

2.2 IR spectrum for the monomers SM-13

3 t-SNE representation of *k*-means clustering SM-21

4 Performance of MBASM+DFT versus CREST+GFN2-xTB SM-22

5 Energetic properties of all amino acids-monomer complexes obtained with MBASM+DFT approach SM-22

6 Advanced insights for relevant amino acid-monomer complexes SM-255

7 Molecular Docking SM-258

7.1 Preparation and Validation of the Receptor Model SM-258

7.2 Molecular docking results for the complexes formed between 1GVZ aminoacids and monomers SM-260

8 Data and MBASM code availability SM-266

1 Introduction

This supplementary material provides additional details on the methodologies and computational analyses employed in this study, offering a deeper understanding of monomer-amino acid interactions in molecularly imprinted polymers (MIPs) for PSA detection using the MBASM, Density Functional Theory (DFT) and Molecular Docking approach.

The document begins with the infrared (IR) spectrum analysis of amino acids and monomers, which helps in characterizing their functional groups and potential interaction sites. Subsequently, a t-SNE representation of k-means clustering is presented to visualize the distribution and classification of amino acid-monomer complexes based on their interaction patterns. A comparative study between MBASM+DFT and CREST+GFN2-xTB is performed to evaluate the efficiency of the MBASM methodology.

The energetic properties of all amino acid-monomer complexes are examined to highlight

key interactions relevant to PSA detection. Additionally, advanced insights into specific amino acid-monomer complexes provide a refined understanding of the binding mechanisms.

Finally, molecular docking simulations are conducted, detailing the preparation and validation of the receptor model and the interaction results of 1GVZ amino acids with selected monomers. These docking studies complement the computational findings, reinforcing the reliability of the proposed MIP design strategy.

This supplementary material extends the main manuscript, enhancing transparency and reproducibility of the computational and experimental methods used.

2 Infrared (IR) spectrum

2.1 IR spectrum for the aminoacids (AA)

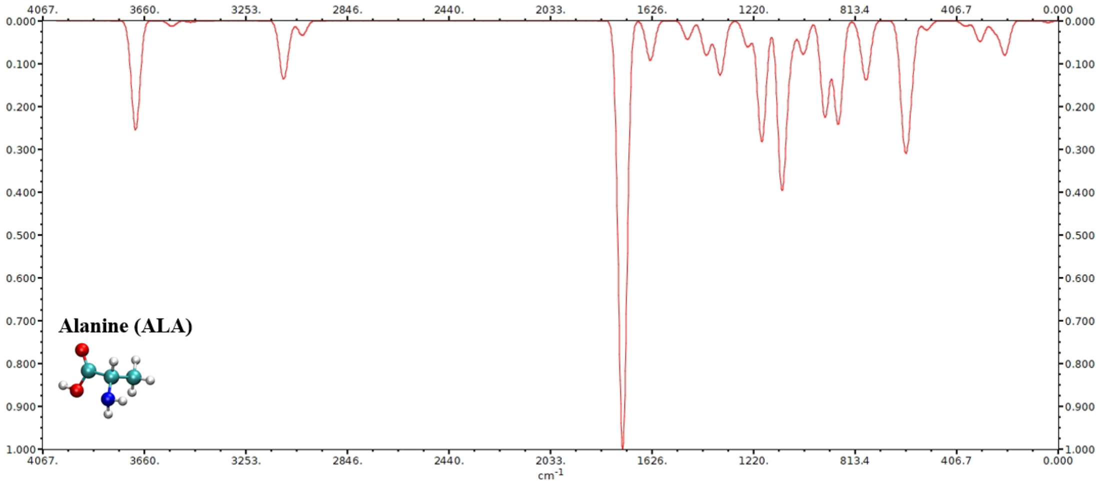

Figure SM1: IR spectra for ALA.

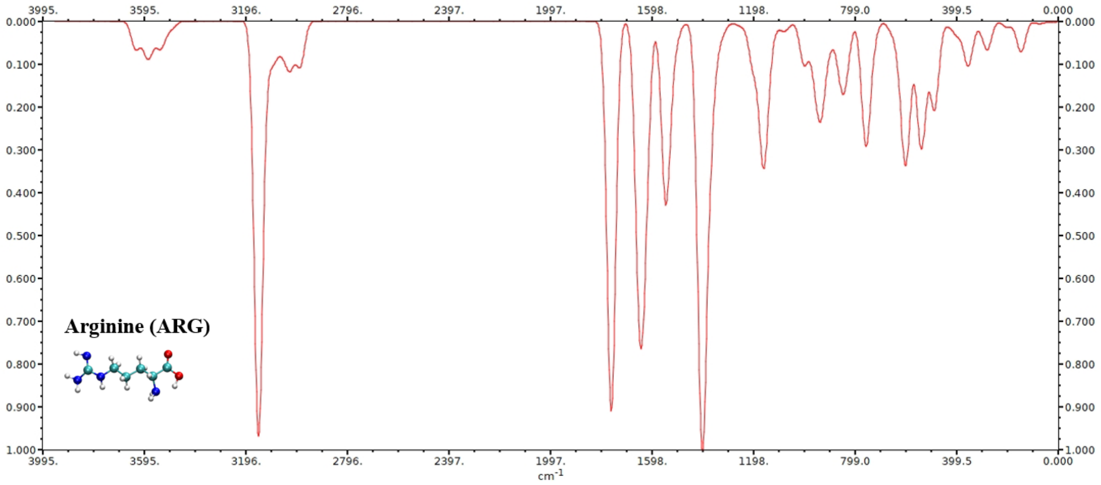

Figure SM2: IR spectra for ARG.

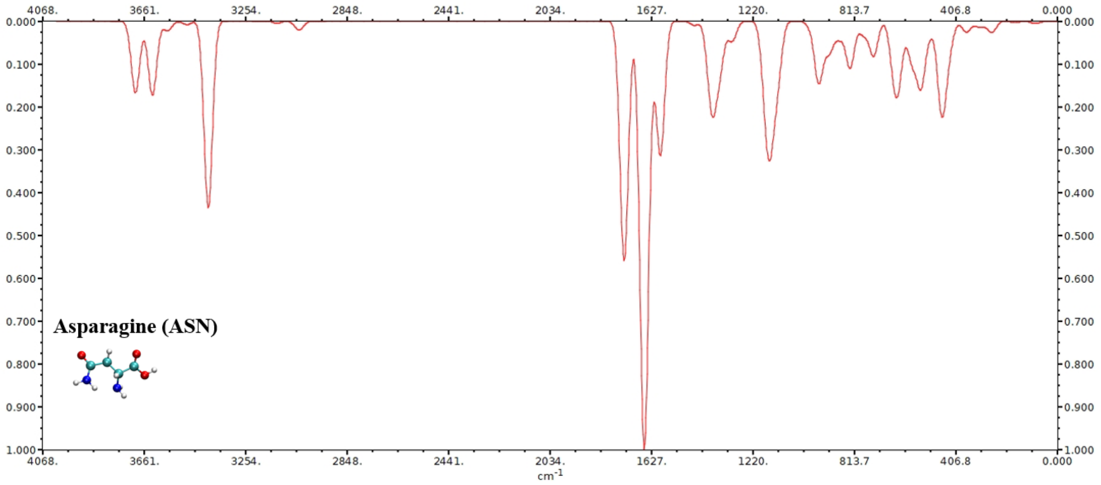

Figure SM3: IR spectra for ASN.

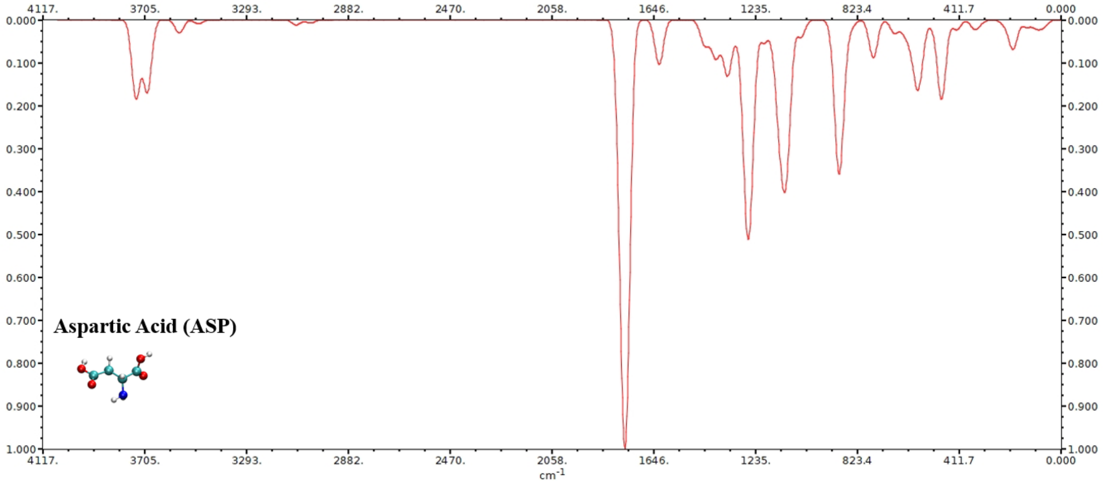

Figure SM4: IR spectra for ASP.

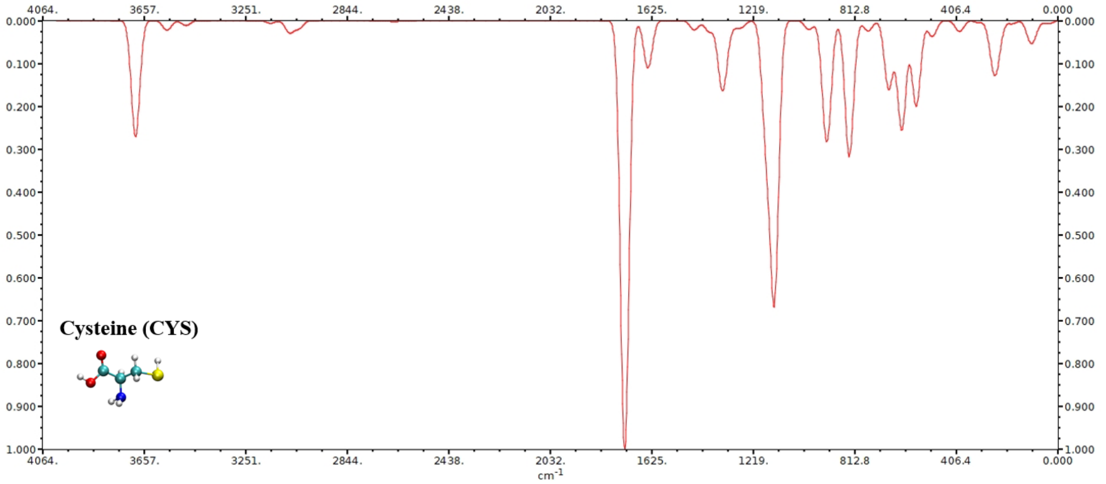

Figure SM5: IR spectra for CYS.

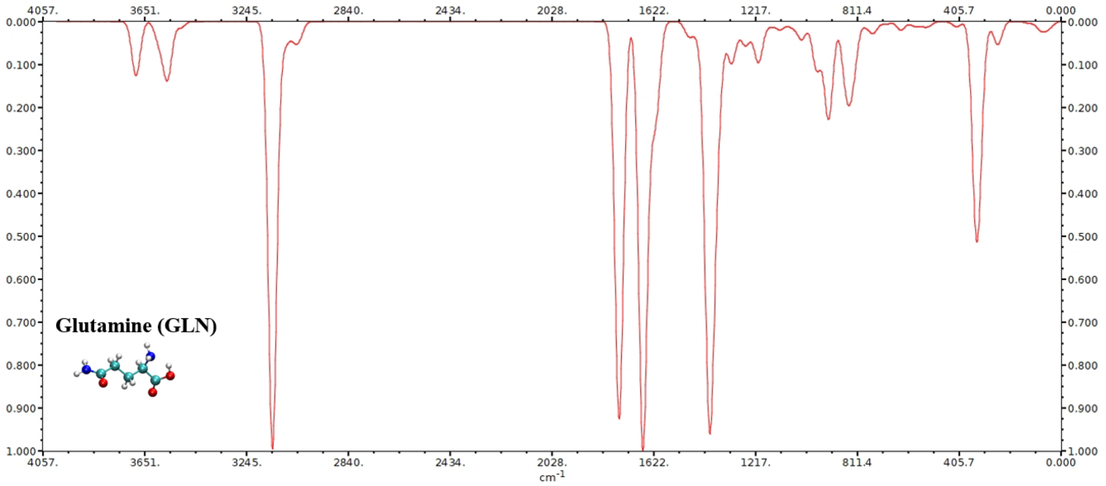

Figure SM6: IR spectra for GLN.

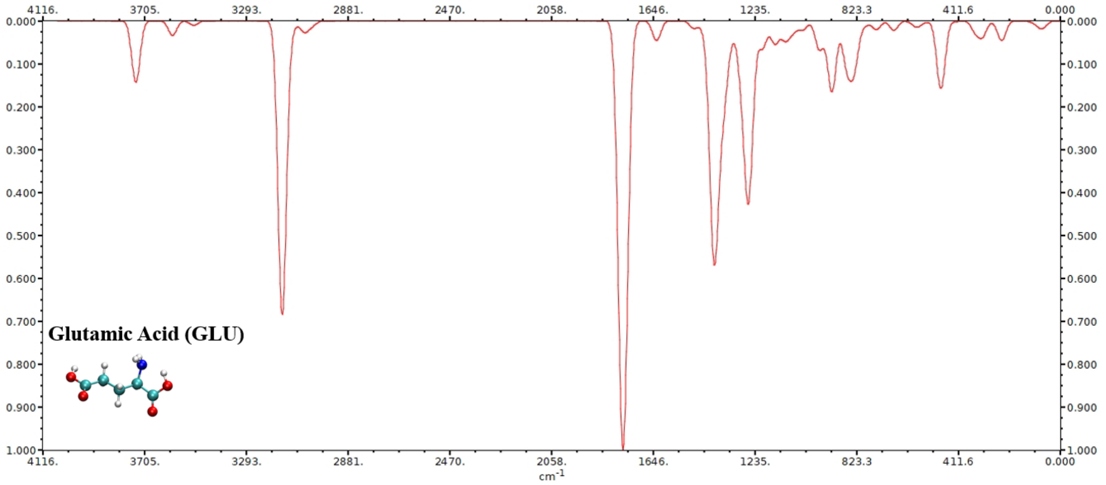

Figure SM7: IR spectra for GLU.

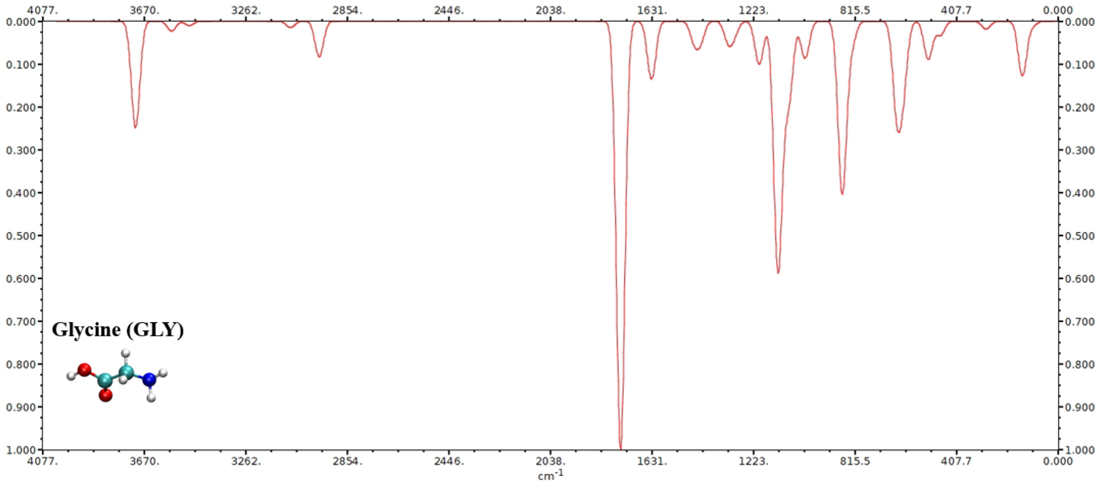

Figure SM8: IR spectra for GLY.

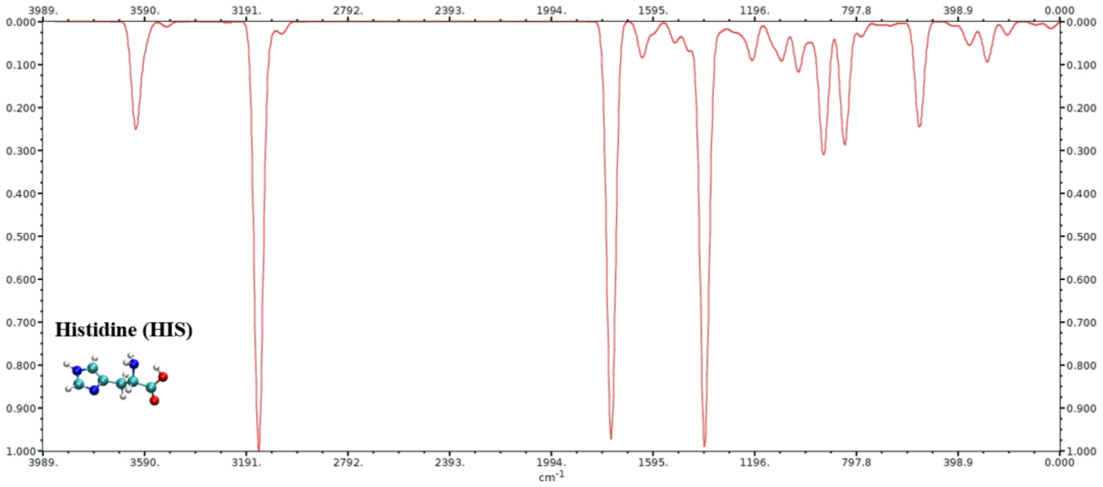

Figure SM9: IR spectra for HYS.

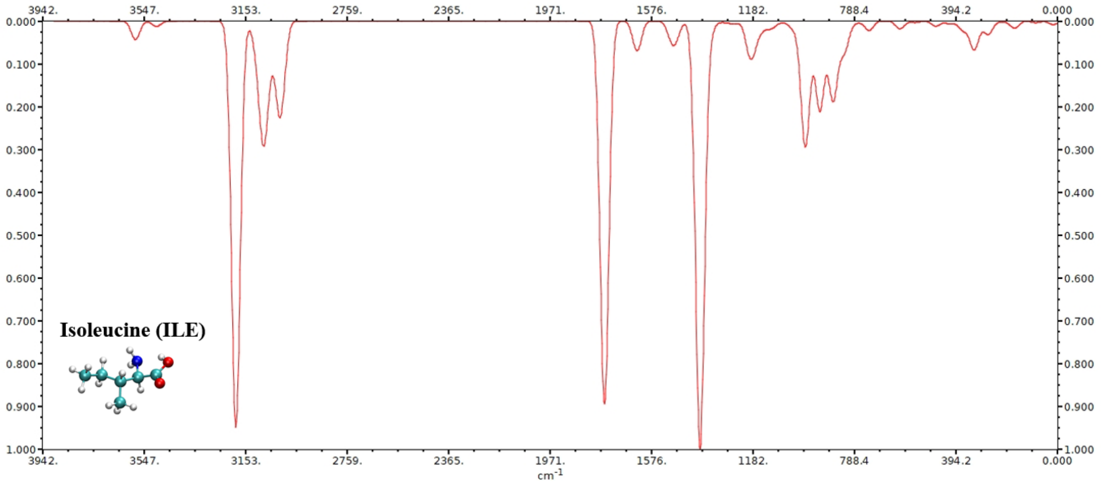

Figure SM10: IR spectra for ILE.

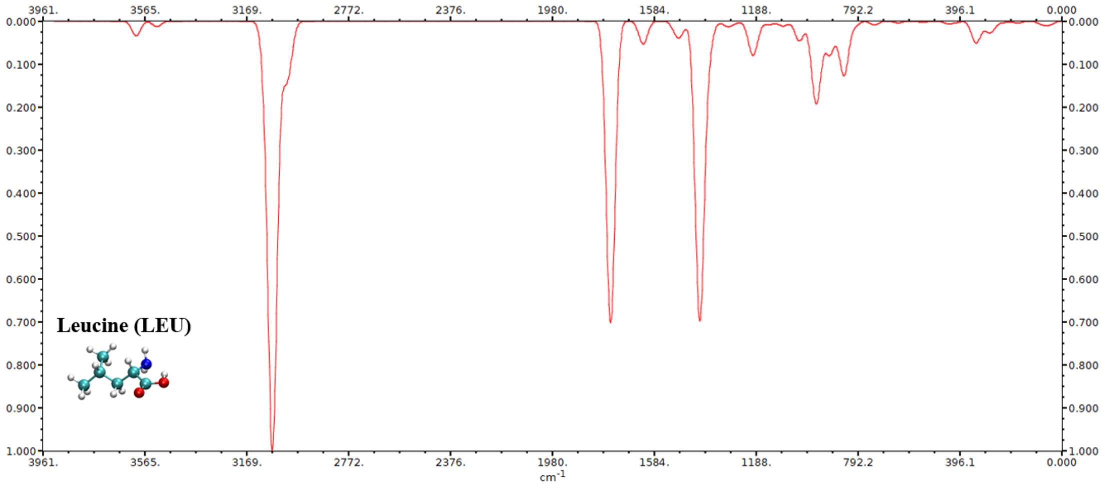

Figure SM11: IR spectra for LEU.

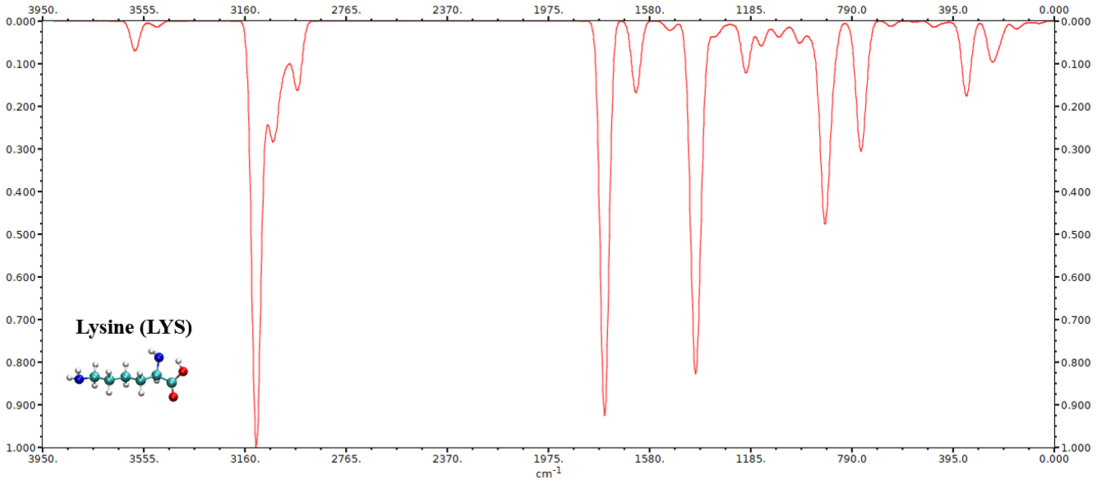

Figure SM12: IR spectra for LYS.

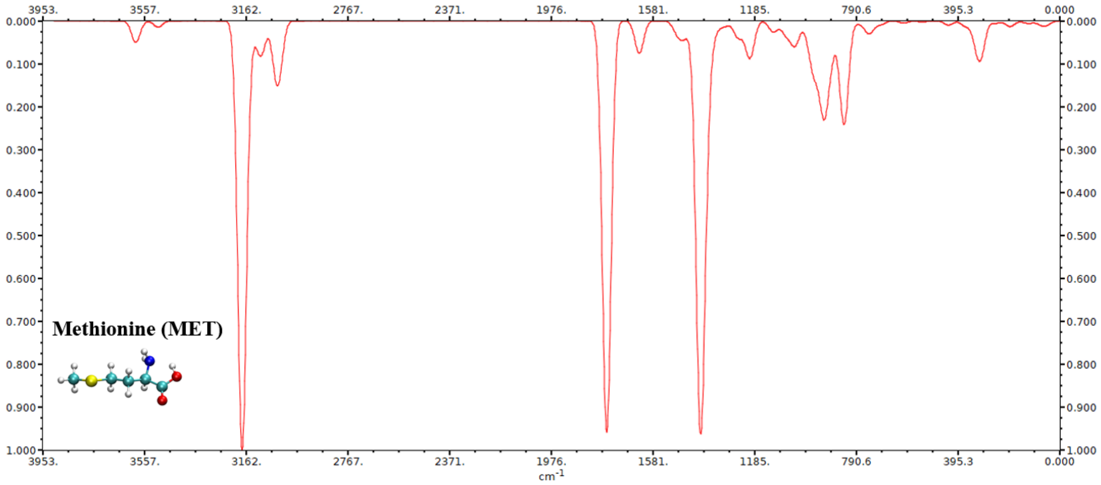

Figure SM13: IR spectra for MET.

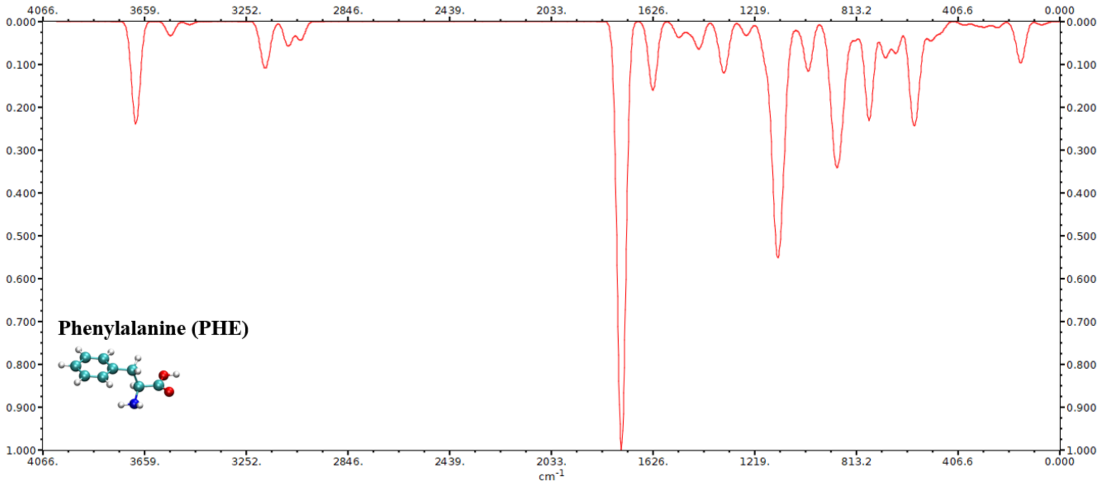

Figure SM14: IR spectra for PHE.

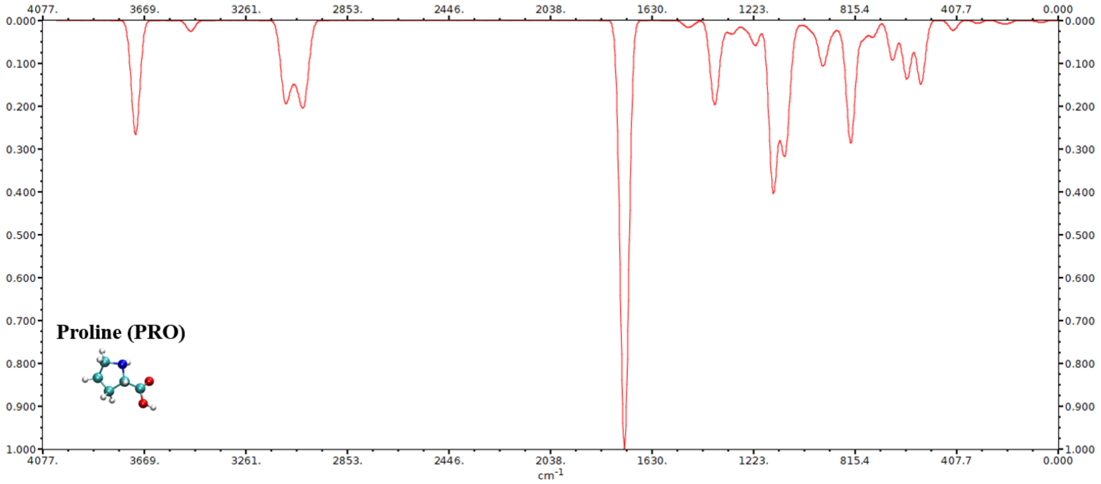

Figure SM15: IR spectra for PRO.

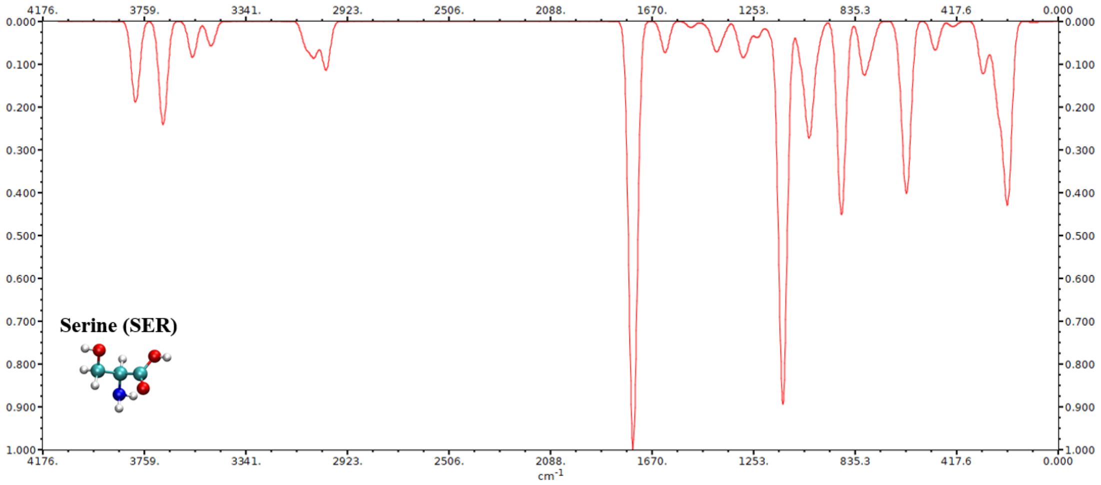

Figure SM16: IR spectra for SER.

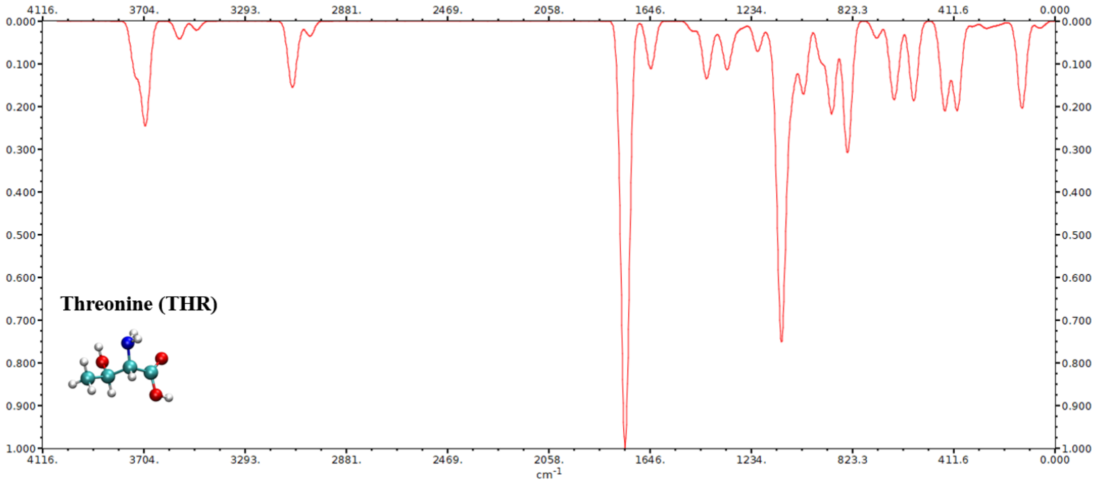

Figure SM17: IR spectra for THR.

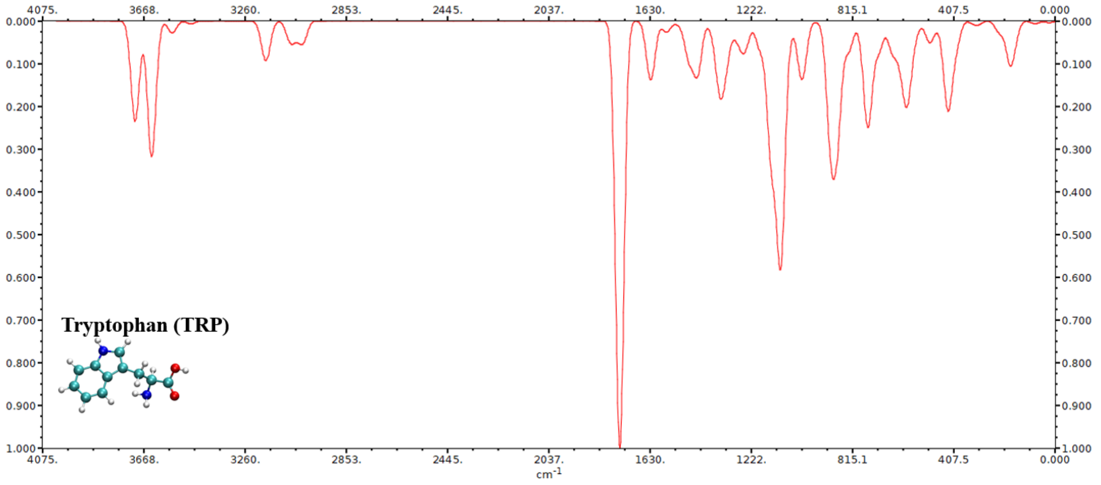

Figure SM18: IR spectra for TRP.

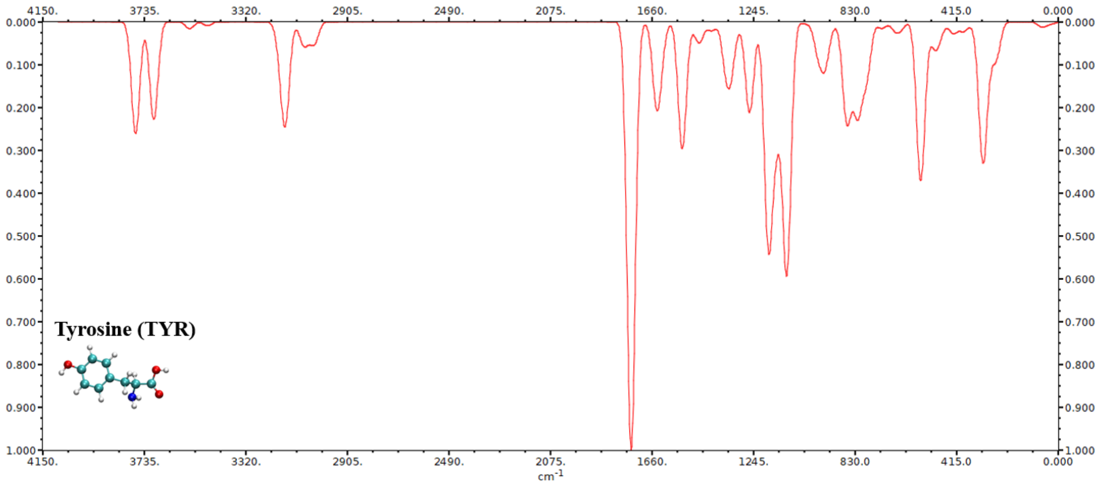

Figure SM19: IR spectra for TYR.

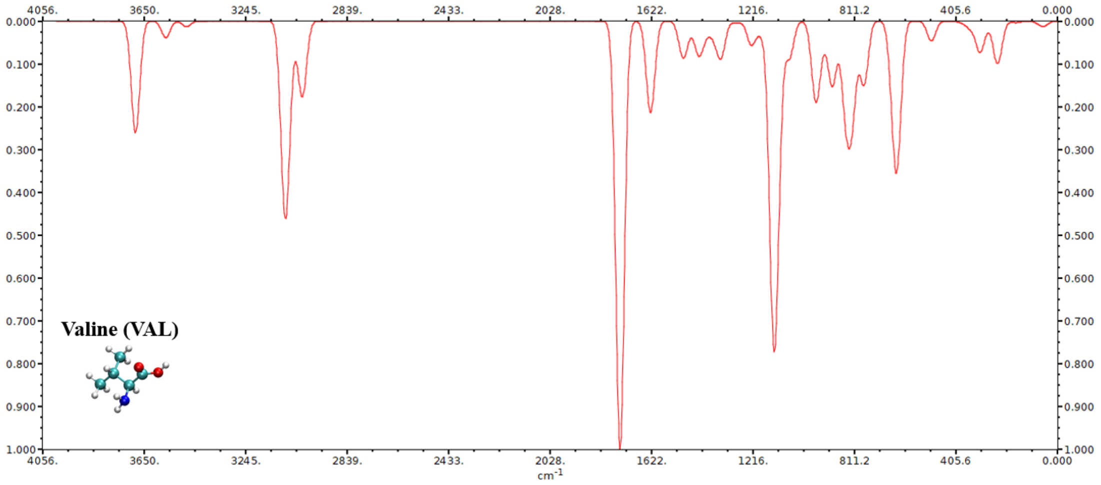

Figure SM20: IR spectra for VAL.

2.2 IR spectrum for the monomers

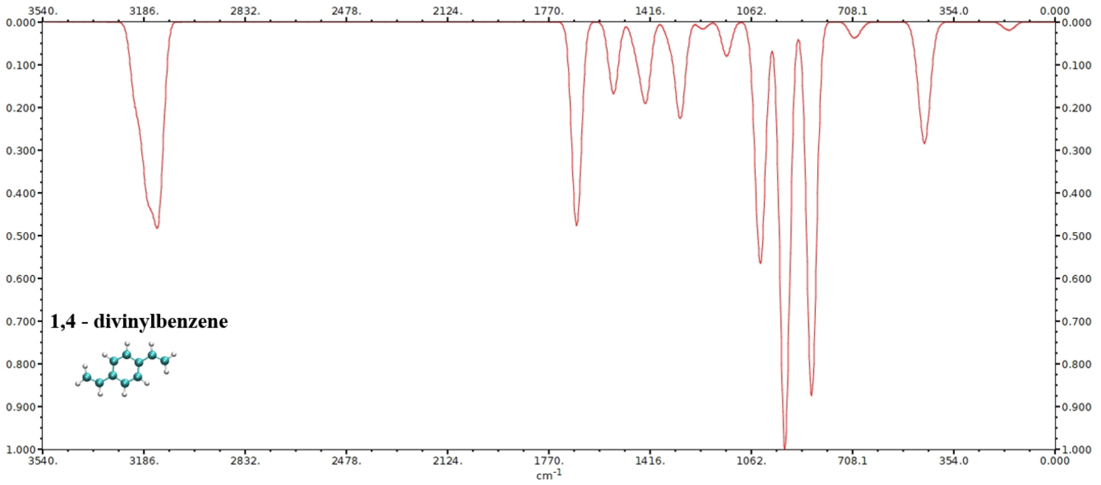

Figure SM21: IR for 1,4-Divinylbenzene.

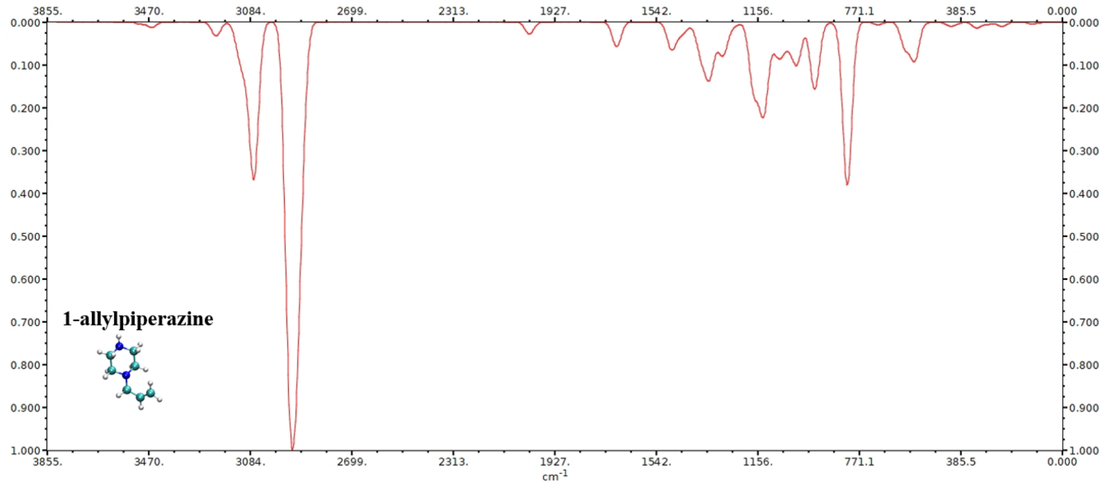

Figure SM22: IR for 1-allylpiperazine.

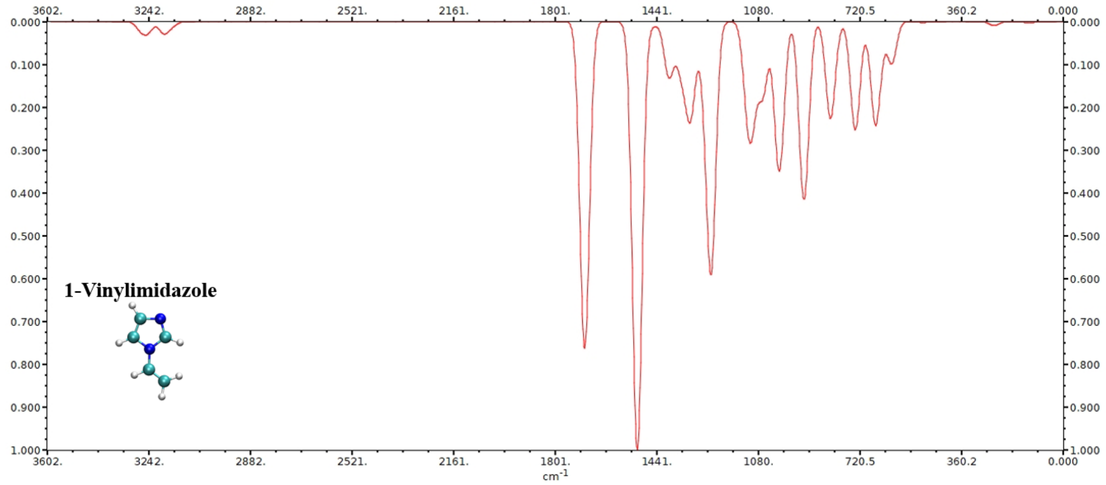

Figure SM23: IR for 1-vinylimidazole.

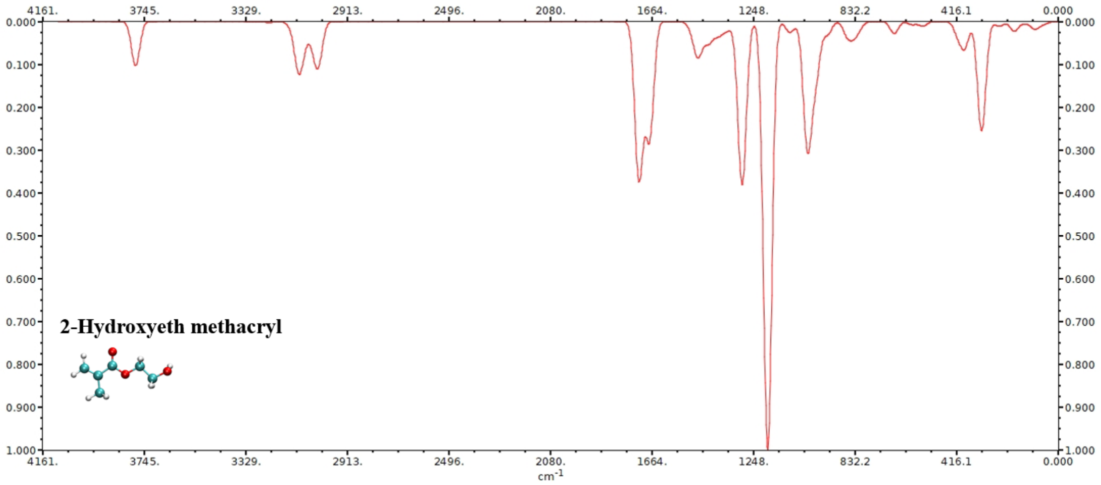

Figure SM24: IR for 2-hydroxyeth methacryl.

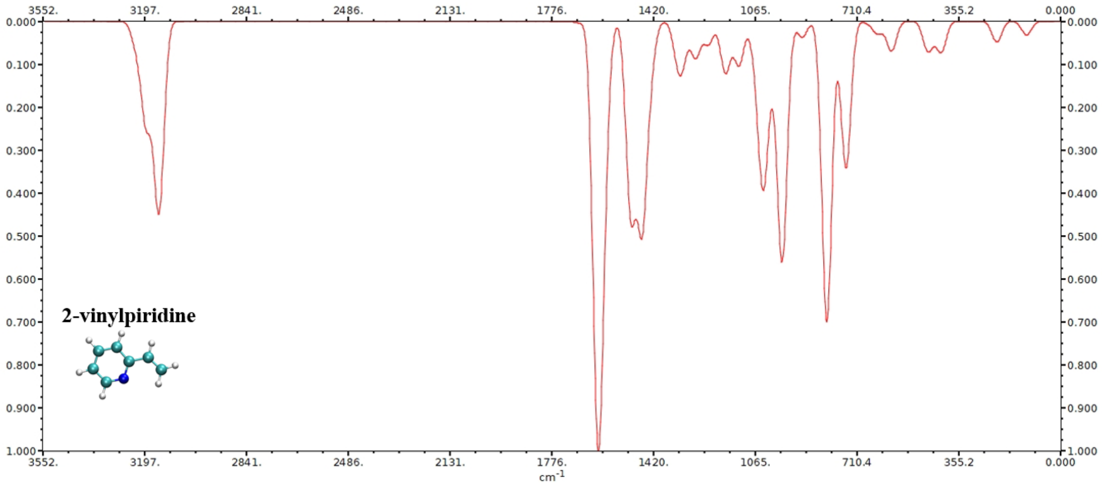

Figure SM25: IR for 2-vinylpiridine.

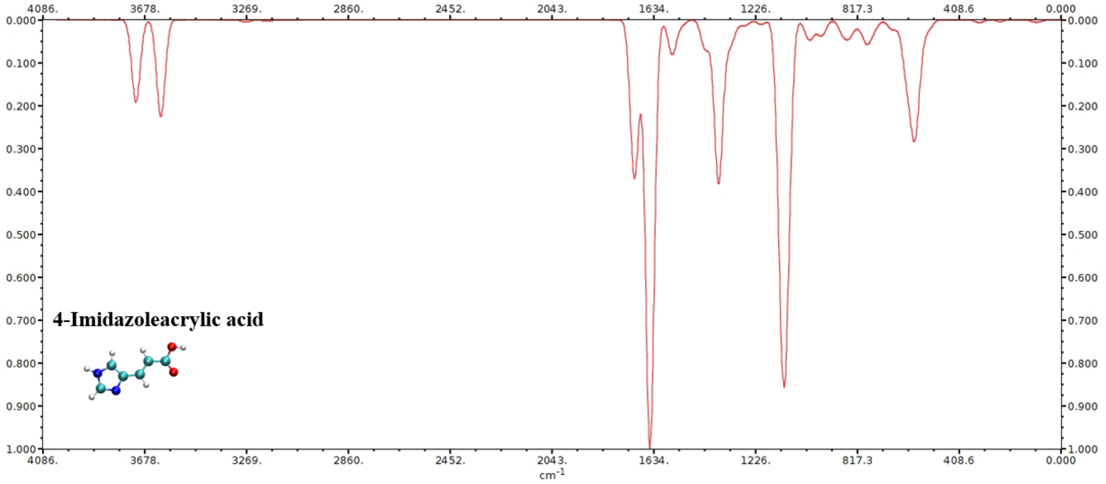

Figure SM26: IR for 4-imidazoleacrylic acid.

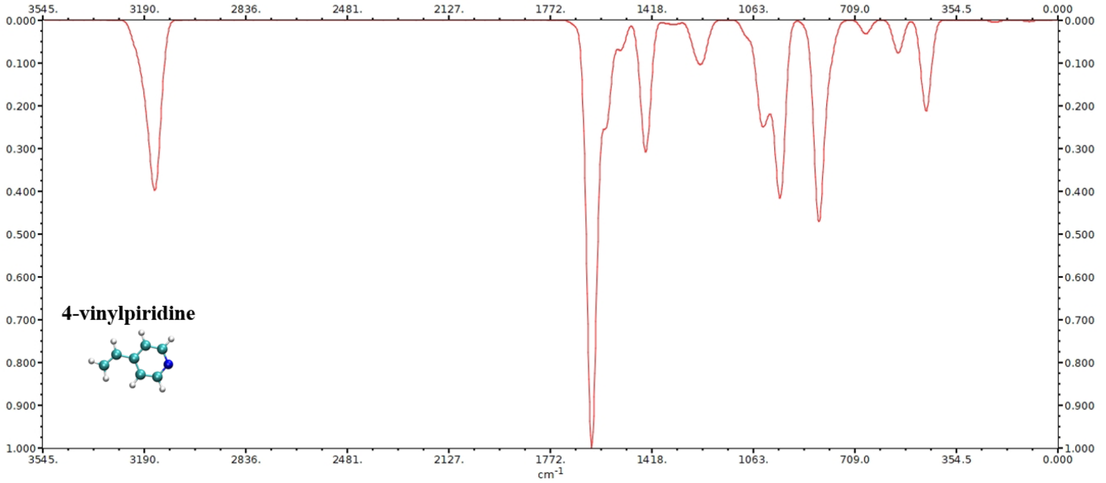

Figure SM27: IR for 4-vinylpiridine.

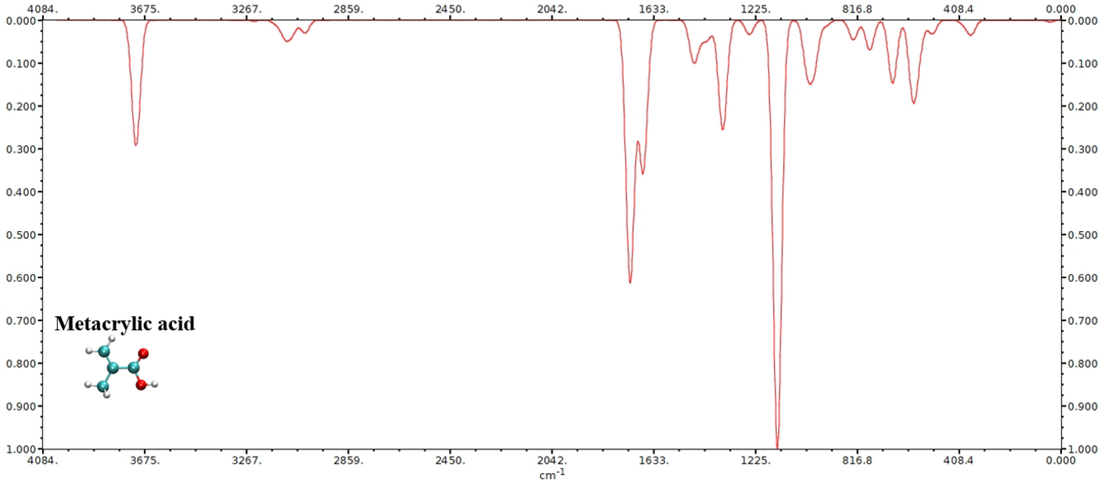

Figure SM28: IR for metacrylic acid.

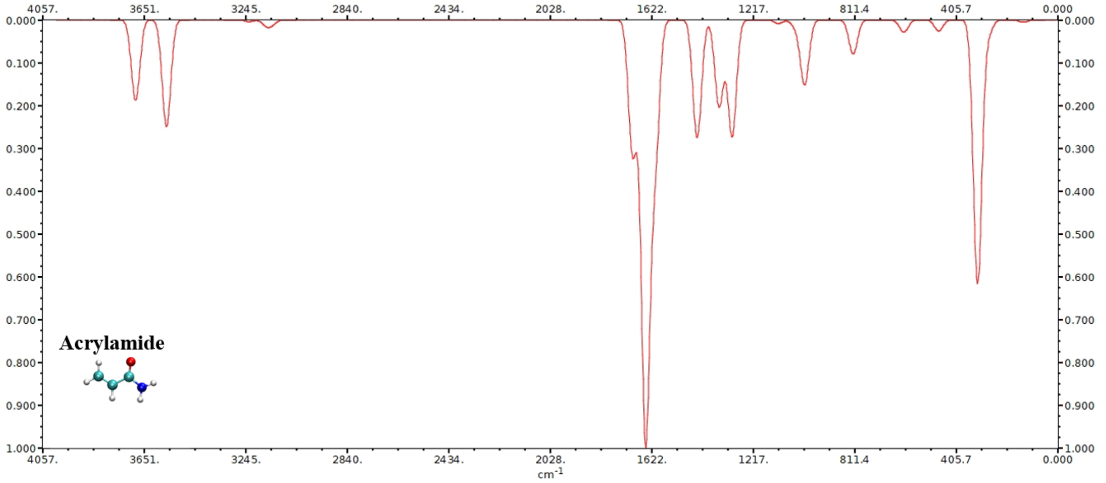

Figure SM29: IR for acrylamide.

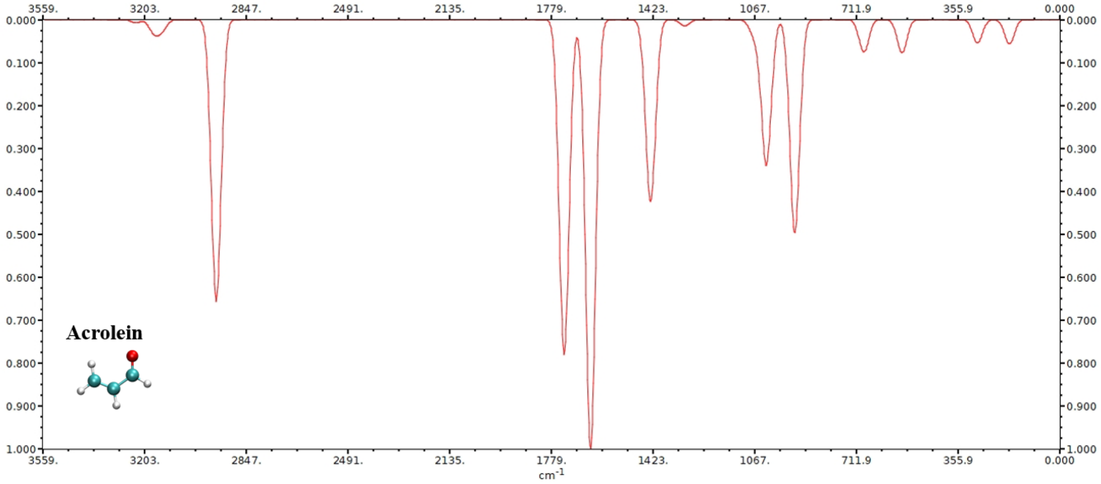

Figure SM30: IR for acrolein.

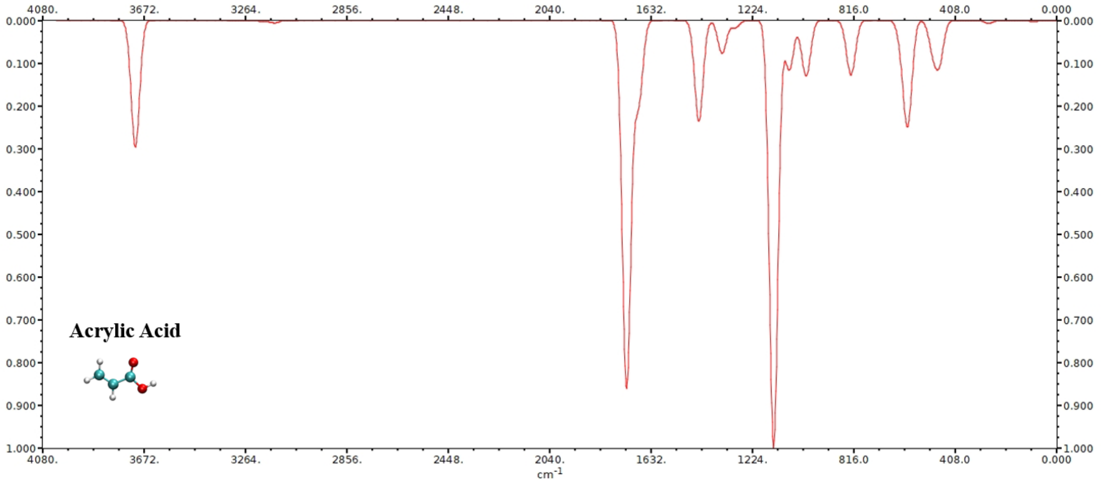

Figure SM31: IR for acrylic acid.

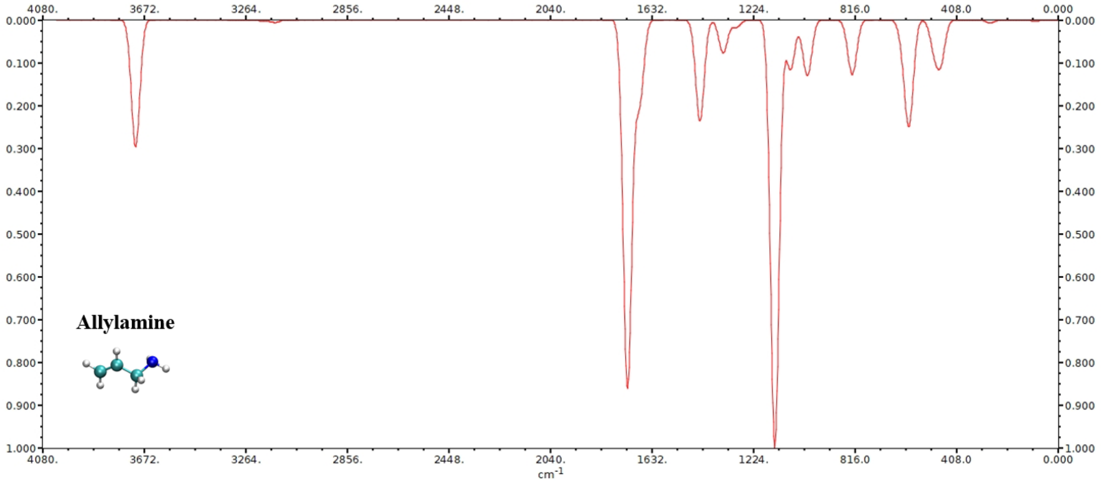

Figure SM32: IR for allylamine.

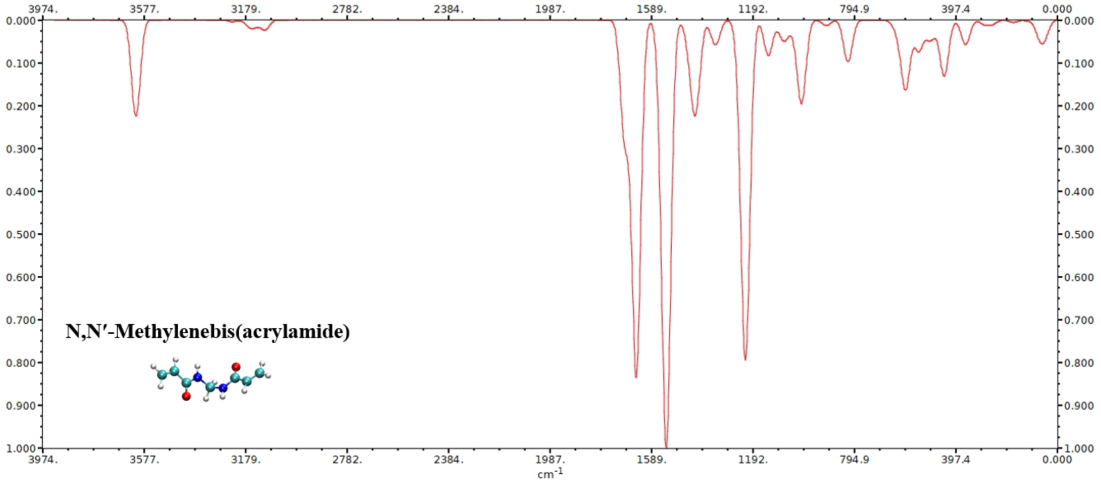

Figure SM33: IR for acrylamide.

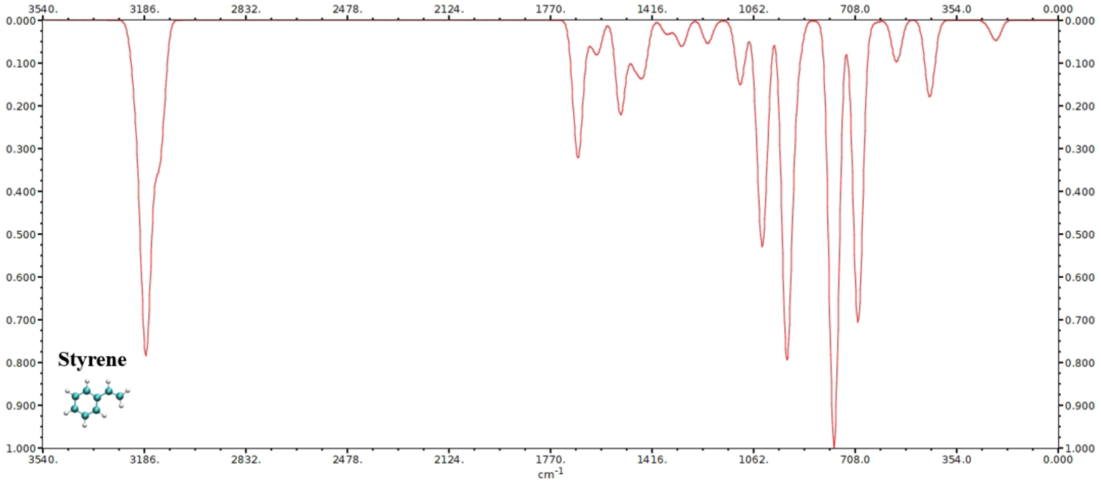

Figure SM34: IR for styrene.

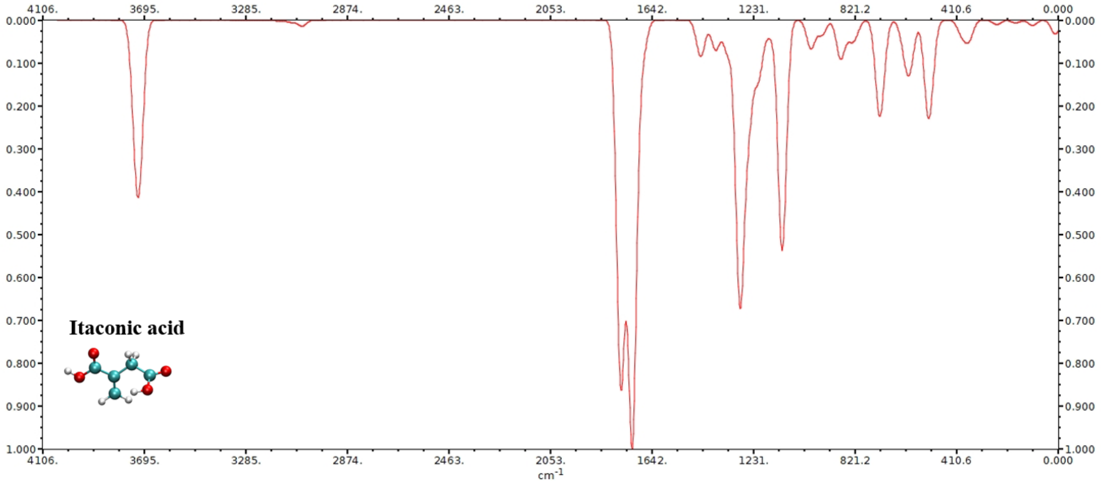

Figure SM35: IR for itaconic acid.

3 t-SNE representation of *k*-means clustering

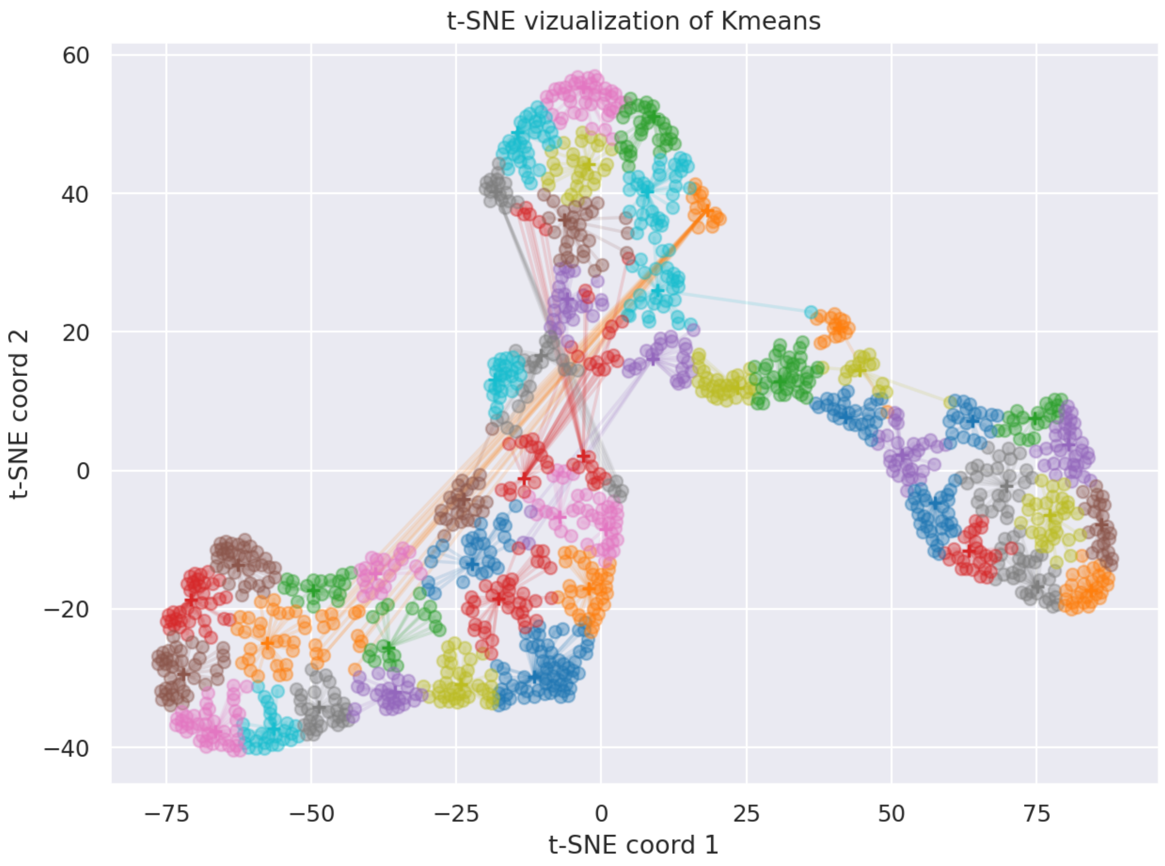

Figure SM36: Each mark represents an adsorbed analyte-monomer structure. Circles denote structures that were not utilized, while a plus sign indicates the representative structure of each group identified by *k-means*, each marked with a different color. Lines indicate structures that belong to the group identified by the corresponding color but were separated from it by the t-SNE procedure.

4 Performance of MBASM+DFT versus CREST+GFN2-xTB

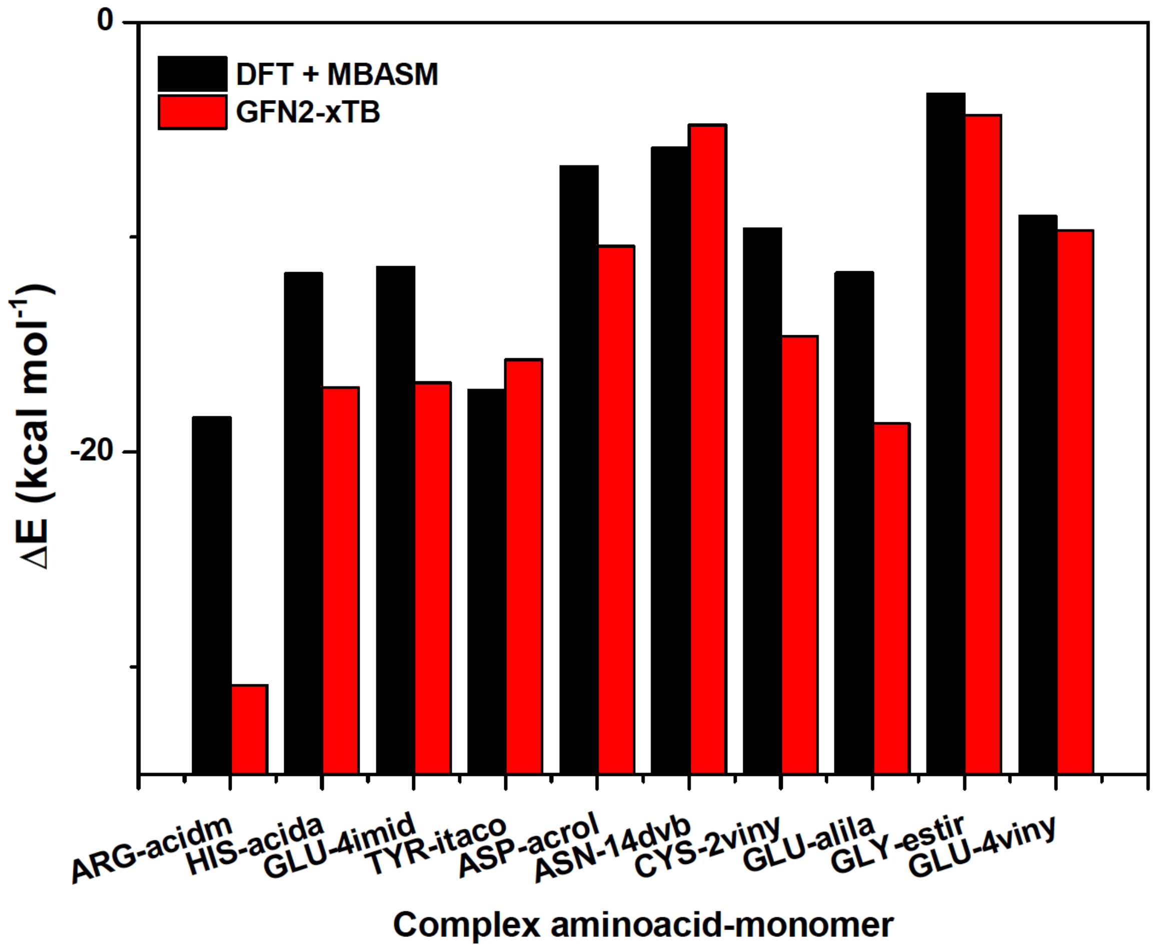

Figure SM37: Binding energy relationship (ΔE in $\mathrm{kcal\,mol^{-1}}$) for the most relevant amino acid-functional monomer complexes, comparing the MBASM+DFT methodology with CREST+GFN2-xTB.

5 Energetic properties of all amino acids-monomer complexes obtained with MBASM+DFT approach

Table SM1: Structures of various conformations are evaluated for their energetic properties and types of intermolecular interactions. In this context, Am stands for amino acid, FM for functional monomer, N° conf. for the spatial conformation number of the Amino acid-FM complex, E_{tot} represents the ground state electronic energy in kcal mol^{-1}, the ΔE represents the difference of the electronic energy in ascending order of energy between the complex and lastly, the type of interaction specifies the atoms that are in close proximity in the table. The symbols Hb denote a hydrogen bond, AmtoM indicates that an AM is complexing with an FM, and MtoAm is the reverse of AmtoM. The symbols SB denote a salt bridges. The symbols Hp denote a hydrophobics interactions. The symbols Cation-pi/pi-staquing/pi-T-sheped denote the type of π interactions interactions.

AA	FM	N° conf.	ΔE	E_{tot}	Type of interaction
ALA	14dvb	5	0.000000000000	-3.93	8C.-.C 25C.-.H 13H.-.H 4C.-.N H.-.N 8Hp Cation-pi(Amc)
		2	0.445938148394	-3.49	18C.-.H 6C.-.C 2C.-.N 10H.-.H 3H.-.N 6Hp
		3	0.770008642765	-3.16	4C.-.N 18C.-.H 2H.-.N 11H.-.H 8C.-.O 6C.-.C 2H.-.O 6Hp
		1	1.269727729843	-2.66	5C.-.H 6H.-.O 4H.-.H 5C.-.O C.-.C Hp
		4	1.695400499655	-2.24	5C.-.H 4C.-.O 4H.-.H C.-.C 5H.-.O Hp
		9	2.663292226543	-1.27	9C.-.H 2C.-.C C.-.O 7H.-.H 3H.-.O 2Hp
		7	3.023995034707	-0.91	10C.-.H 10H.-.H 2C.-.C C.-.N 2H.-.N 2Hp
		6	3.892995656371	-0.04	
		0	3.964030115179	0.03	
		8	4.019389504053	0.09	

Continue in the next page

Table SM1: Structures of various conformations are evaluated for their energetic properties and types of intermolecular interactions. In this context, Am stands for amino acid, FM for functional monomer, N° conf. for the spatial conformation number of the Amino acid-FM complex, E_{tot} represents the ground state electronic energy in kcal mol^{-1}, the ΔE represents the difference of the electronic energy in ascending order of energy between the complex and lastly, the type of interaction specifies the atoms that are in close proximity in the table. The symbols Hb denote a hydrogen bond, AmtoM indicates that an AM is complexing with an FM, and MtoAm is the reverse of AmtoM. The symbols SB denote a salt bridges. The symbols Hp denote a hydrophobics interactions. The symbols Cation-pi/pi-staquing/pi-T-sheped denote the type of π interactions interactions.

AA	FM	N° conf.	ΔE	E_{tot}	Type of interaction
	2viny	2	0.000000000000	-3.67	7C.-.C 12C.-.H 6C.-.O C.-.N 3H.-.N N.-.O 8H.-.H 5H.-.O 7Hp Cation-pi(Amc)
		0	2.164403366245	-1.50	9C.-.H 2C.-.O 6H.-.H 3H.-.O H.-.N
		7	2.788488799960	-0.88	2C.-.C 8C.-.H 8H.-.H H.-.N 2Hp
		9	3.695995373142	0.03	
		3	3.706712038641	0.04	
		6	3.731346462837	0.07	
	acidm	2	0.000000000000	-4.96	4C.-.H 3C.-.O 5H.-.O 2O.-.O 2H.-.H Hb(AmtoM)
		6	1.374754688537	-3.59	C.-.C 4C.-.O 6C.-.H 6H.-.H 7H.-.O 2O.-.O Hp Hb(AmtoM)
		8	2.202374744051	-2.76	12C.-.H 6C.-.O 2H.-.N 10H.-.H 7H.-.O 2C.-.N 3C.-.C 3O.-.O 3Hp SB

Continue in the next page

Table SM1: Structures of various conformations are evaluated for their energetic properties and types of intermolecular interactions. In this context, Am stands for amino acid, FM for functional monomer, N° conf. for the spatial conformation number of the Amino acid-FM complex, E_{tot} represents the ground state electronic energy in kcal mol^{-1}, the ΔE represents the difference of the electronic energy in ascending order of energy between the complex and lastly, the type of interaction specifies the atoms that are in close proximity in the table. The symbols Hb denote a hydrogen bond, AmtoM indicates that an AM is complexing with an FM, and MtoAm is the reverse of AmtoM. The symbols SB denote a salt bridges. The symbols Hp denote a hydrophobics interactions. The symbols Cation-pi/pi-staquing/pi-T-sheped denote the type of π interactions interactions.

AA	FM	N° conf.	ΔE	E_{tot}	Type of interaction
		4	3.071714734104	-1.89	5C.-.C C.-.N 8C.-.H 6C.-.O 2H.-.N 6H.-.H 7H.-.O 2O.-.O 5Hp SB
		7	3.253938434229	-1.71	6C.-.C 4C.-.N 14C.-.H 2H.-.N 8H.-.H 4C.-.O 2N.-.O 8H.-.O O.-.O 6Hp SB
		9	4.202061156731	-0.76	C.-.C 7C.-.H C.-.O 5H.-.H 2H.-.O Hp
		1	4.924930543075	-0.04	
		0	4.977816190123	0.01	
		3	4.989879804270	0.03	
	acida	0	0.000000000000	-1.80	6C.-.H C.-.O 7H.-.H C.-.N 2H.-.N 6H.-.O N.-.O Hb(AmtoM) SB
		6	0.164301781306	-1.63	C.-.N 6C.-.O 9C.-.H H.-.N 5H.-.O 3H.-.H C.-.C N.-.O 2O.-.O Hp Hb(AmtoM) SB

Continue in the next page

Table SM1: Structures of various conformations are evaluated for their energetic properties and types of intermolecular interactions. In this context, Am stands for amino acid, FM for functional monomer, Nº conf. for the spatial conformation number of the Amino acid-FM complex, E_{tot} represents the ground state electronic energy in kcal mol^{-1}, the ΔE represents the difference of the electronic energy in ascending order of energy between the complex and lastly, the type of interaction specifies the atoms that are in close proximity in the table. The symbols Hb denote a hydrogen bond, AmtoM indicates that an AM is complexing with an FM, and MtoAm is the reverse of AmtoM. The symbols SB denote a salt bridges. The symbols Hp denote a hydrophobics interactions. The symbols Cation-pi/pi-staquing/pi-T-sheped denote the type of π interactions interactions.

AA	FM	Nº conf.	ΔE	E_{tot}	Type of interaction
		4	0.253065357484	-1.54	5C.-.C 11C.-.H 7H.-.H H.-.N 5H.-.O 2C.-.O O.-.O 5Hp SB
		1	1.263544747906	-0.53	5C.-.H C.-.N 4H.-.H H.-.N 2H.-.O O.-.O SB
		5	1.827259838558	0.03	
		8	2.066417479696	0.27	
	bisac	0	0.000000000000	-6.14	C.-.C 4C.-.N 2C.-.O 7C.-.H 11H.-.H 6H.-.N 5H.-.O N.-.N N.-.O Hp Hb(MtoAm)
		2	0.101745650891	-6.04	3C.-.N 6C.-.H 7H.-.N 2C.-.O 7H.-.O N.-.O N.-.N 8H.-.H Hb(AmtoM) Hb(MtoAm)
		6	1.499145603122	-4.64	4C.-.N 9C.-.H 6H.-.N 4C.-.O 8H.-.O 2N.-.O C.-.C 5H.-.H N.-.N Hp Hb(AmtoM) Hb(MtoAm)
		1	5.355804386372	-0.79	4C.-.H C.-.N 2H.-.N C.-.C 6H.-.H Hp

Continue in the next page

Table SM1: Structures of various conformations are evaluated for their energetic properties and types of intermolecular interactions. In this context, Am stands for amino acid, FM for functional monomer, N° conf. for the spatial conformation number of the Amino acid-FM complex, E_{tot} represents the ground state electronic energy in kcal mol^{-1}, the ΔE represents the difference of the electronic energy in ascending order of energy between the complex and lastly, the type of interaction specifies the atoms that are in close proximity in the table. The symbols Hb denote a hydrogen bond, AmtoM indicates that an AM is complexing with an FM, and MtoAm is the reverse of AmtoM. The symbols SB denote a salt bridges. The symbols Hp denote a hydrophobics interactions. The symbols Cation-pi/pi-staquing/pi-T-sheped denote the type of π interactions interactions.

AA	FM	N° conf.	ΔE	E_{tot}	Type of interaction
		3	6.170099992837	0.03	
		8	6.234613117499	0.09	
	1ally	6	0.000000000000	-0.62	5C.-.H 10H.-.H H.-.O
		3	0.246118109003	-0.38	5H.-.N 9H.-.H C.-.N 9C.-.H 3C.-.O 5H.-.O N.-.O 2SB
		0	0.303992678290	-0.32	2C.-.O 2C.-.H H.-.N 4H.-.O 5H.-.H SB
		7	0.478542919458	-0.15	
		2	0.506983477836	-0.12	
		1	0.511722739277	-0.11	
		4	0.568445492588	-0.06	
		5	0.601218356198	-0.02	
	4imid	1	0.000000000000	-9.40	4C.-.O 4C.-.H 3H.-.O 2H.-.H C.-.N 2N.-.O 2H.-.N Hb(AmtoM)
		3	5.912765949091	-3.49	3C.-.O 2O.-.O 5H.-.O 5C.-.H 5H.-.H 2H.-.N Hb(AmtoM)

Continue in the next page

Table SM1: Structures of various conformations are evaluated for their energetic properties and types of intermolecular interactions. In this context, Am stands for amino acid, FM for functional monomer, N° conf. for the spatial conformation number of the Amino acid-FM complex, E_{tot} represents the ground state electronic energy in kcal mol^{-1}, the ΔE represents the difference of the electronic energy in ascending order of energy between the complex and lastly, the type of interaction specifies the atoms that are in close proximity in the table. The symbols Hb denote a hydrogen bond, AmtoM indicates that an AM is complexing with an FM, and MtoAm is the reverse of AmtoM. The symbols SB denote a salt bridges. The symbols Hp denote a hydrophobics interactions. The symbols Cation-pi/pi-staquing/pi-T-sheped denote the type of π interactions interactions.

AA	FM	N° conf.	ΔE	E_{tot}	Type of interaction
		0	6.073017835060	-3.33	13C.-.H 3C.-.C 5C.-.N 6H.-.H 7H.-.N N.-.N 3Hp Hb(AmtoM)
		8	6.527553656320	-2.88	7C.-.H 5H.-.N 6H.-.H 2C.-.N N.-.N C.-.C Hp Hb(AmtoM)
		9	7.214985580529	-2.19	C.-.O 2H.-.O 10C.-.H 8H.-.H C.-.C 4C.-.N 2H.-.N Hp SB
		6	7.719308906759	-1.68	7C.-.O 5H.-.O 11C.-.H 9H.-.H 2C.-.C 3H.-.N 2Hp
		2	9.721958382175	0.32	
	acril	7	0.000000000000	-6.96	3C.-.O 3C.-.H 2H.-.O H.-.H H.-.N 2O.-.O Hb(AmtoM)
		5	1.611560706358	-5.35	6C.-.H 3C.-.N 8H.-.H 5H.-.N N.-.N H.-.O Hb(MtoAm)
		9	3.658874486597	-3.30	C.-.N 3C.-.H 3C.-.O N.-.N 3H.-.N 2N.-.O 5H.-.O O.-.O 5H.-.H Hb(MtoAm) Hb(AmtoM)

Continue in the next page

Table SM1: Structures of various conformations are evaluated for their energetic properties and types of intermolecular interactions. In this context, Am stands for amino acid, FM for functional monomer, Nº conf. for the spatial conformation number of the Amino acid-FM complex, E_{tot} represents the ground state electronic energy in kcal mol^{-1}, the ΔE represents the difference of the electronic energy in ascending order of energy between the complex and lastly, the type of interaction specifies the atoms that are in close proximity in the table. The symbols Hb denote a hydrogen bond, AmtoM indicates that an AM is complexing with an FM, and MtoAm is the reverse of AmtoM. The symbols SB denote a salt bridges. The symbols Hp denote a hydrophobics interactions. The symbols Cation-pi/pi-staquing/pi-T-sheped denote the type of π interactions interactions.

AA	FM	Nº conf.	ΔE	E_{tot}	Type of interaction
		2	4.177759004403	-2.78	11C.-.H 4C.-.N 9H.-.H 4H.-.N 3C.-.C 2C.-.O 4H.-.O N.-.O 3Hp Hb(AmtoM)
		6	4.206989120287	-2.76	7C.-.H 2C.-.N 5H.-.H 2H.-.N 2C.-.O 4H.-.O N.-.O Hb(AmtoM)
		0	4.286498521123	-2.68	2C.-.O 3C.-.H 3H.-.O 2H.-.H N.-.O Hb(MtoAm)
		8	6.947979836735	-0.01	
	alila	2	0.000000000000	-3.43	10C.-.H 4C.-.N 13H.-.H 8H.-.N C.-.C N.-.N Hp Hb(AmtoM)
		4	0.777538246388	-2.65	3C.-.N 8C.-.H 11H.-.H 6H.-.N N.-.N H.-.O Hb(MtoAm) SB
		3	0.795361990530	-2.63	8C.-.H 2C.-.O 9H.-.H 6H.-.N 2H.-.O C.-.N N.-.N N.-.O Hb(AmtoM) SB

Continue in the next page

Table SM1: Structures of various conformations are evaluated for their energetic properties and types of intermolecular interactions. In this context, Am stands for amino acid, FM for functional monomer, N° conf. for the spatial conformation number of the Amino acid-FM complex, E_{tot} represents the ground state electronic energy in kcal mol^{-1}, the ΔE represents the difference of the electronic energy in ascending order of energy between the complex and lastly, the type of interaction specifies the atoms that are in close proximity in the table. The symbols Hb denote a hydrogen bond, AmtoM indicates that an AM is complexing with an FM, and MtoAm is the reverse of AmtoM. The symbols SB denote a salt bridges. The symbols Hp denote a hydrophobics interactions. The symbols Cation-pi/pi-staquing/pi-T-sheped denote the type of π interactions interactions.

AA	FM	N° conf.	ΔE	E_{tot}	Type of interaction
		0	0.921742370713	-2.50	2C.-.C 10C.-.H 9H.-.H C.-.O 2C.-.N 2H.-.N N.-.O 3H.-.O 2Hp Hb(MtoAm) SB
		8	2.522951480615	-0.90	2C.-.O 8C.-.H 2H.-.O 10H.-.H 2H.-.N SB
		9	3.459231823263	0.03	
		5	3.504179077908	0.08	
	estir	6	0.000000000000	-3.35	4C.-.C 18C.-.H 10H.-.H 4C.-.N 4Hp Cation-pi(Amc)
		5	0.202562067729	-3.15	6C.-.C 14C.-.H 12H.-.H C.-.O C.-.N H.-.O 2H.-.N 6Hp Cation-pi(Amc)
		8	1.023392289049	-2.33	15C.-.H C.-.O 2C.-.N 9H.-.H H.-.O 2H.-.N
		9	3.152894679573	-0.20	
	1viny	1	0.000000000000	-3.46	5C.-.H C.-.C 2C.-.N C.-.O 3H.-.H 4H.-.N N.-.N N.-.O 2H.-.O Hp Hb(AmtoM)

Continue in the next page

Table SM1: Structures of various conformations are evaluated for their energetic properties and types of intermolecular interactions. In this context, Am stands for amino acid, FM for functional monomer, N° conf. for the spatial conformation number of the Amino acid-FM complex, E_{tot} represents the ground state electronic energy in kcal mol^{-1}, the ΔE represents the difference of the electronic energy in ascending order of energy between the complex and lastly, the type of interaction specifies the atoms that are in close proximity in the table. The symbols Hb denote a hydrogen bond, AmtoM indicates that an AM is complexing with an FM, and MtoAm is the reverse of AmtoM. The symbols SB denote a salt bridges. The symbols Hp denote a hydrophobics interactions. The symbols Cation-pi/pi-staquing/pi-T-sheped denote the type of π interactions interactions.

AA	FM	N° conf.	ΔE	E_{tot}	Type of interaction
		6	0.351000821298	-3.11	C.-.C 6C.-.H 3C.-.N 5H.-.N N.-.N 4H.-.H Hp Hb(AmtoM)
		5	1.034802278959	-2.43	3C.-.C 4C.-.O 2C.-.N N.-.O 3H.-.N 10C.-.H 4H.-.O 6H.-.H 3Hp
		3	1.370221415949	-2.09	16C.-.H 3C.-.N 7H.-.N N.-.N C.-.C 9H.-.H Hp Hb(AmtoM) Cation-pi(Amc)
		9	2.723746290091	-0.74	8C.-.H 3C.-.O C.-.C 4H.-.H 3H.-.O Hp
		7	3.359105776102	-0.10	
		8	3.459920993852	-0.00	
		0	3.511927753798	0.05	H.-.H H.-.O
		4	3.540008794621	0.08	
	2hydr	7	0.000000000000	-7.66	6H.-.O 2C.-.C 2C.-.N 9C.-.H 10H.-.H 3H.-.N C.-.O N.-.O 2Hp Hb(MtoAm)

Continue in the next page

Table SM1: Structures of various conformations are evaluated for their energetic properties and types of intermolecular interactions. In this context, Am stands for amino acid, FM for functional monomer, N° conf. for the spatial conformation number of the Amino acid-FM complex, E_{tot} represents the ground state electronic energy in kcal mol^{-1}, the ΔE represents the difference of the electronic energy in ascending order of energy between the complex and lastly, the type of interaction specifies the atoms that are in close proximity in the table. The symbols Hb denote a hydrogen bond, AmtoM indicates that an AM is complexing with an FM, and MtoAm is the reverse of AmtoM. The symbols SB denote a salt bridges. The symbols Hp denote a hydrophobics interactions. The symbols Cation-pi/pi-staquing/pi-T-sheped denote the type of π interactions interactions.

AA	FM	N° conf.	ΔE	E_{tot}	Type of interaction
		6	1.659800630512	-6.00	3C.-.O 2C.-.H 5H.-.O 5H.-.H 2O.-.O Hb(AmtoM)
		3	6.267946906979	-1.39	5C.-.C 12C.-.H 4C.-.O 6H.-.O 9H.-.H 2C.-.N H.-.N N.-.O O.-.O 5Hp
		0	6.379723999192	-1.28	11C.-.O 9C.-.H 3C.-.C 6H.-.H 8H.-.O N.-.O 3O.-.O H.-.N 3Hp
		9	7.120797463606	-0.54	O.-.O 4H.-.O 6C.-.H C.-.O 5H.-.H
		1	7.281767156749	-0.38	C.-.O 2C.-.H 4H.-.H 2H.-.O
		8	7.792260343324	0.13	
		4	8.074912821325	0.42	
		5	19.217608201298	11.56	
	4viny	8	0.000000000000	-2.84	6C.-.O 15C.-.H N.-.O 2H.-.N 10H.-.H 4C.-.C 2H.-.O 3C.-.N 4Hp

Continue in the next page

Table SM1: Structures of various conformations are evaluated for their energetic properties and types of intermolecular interactions. In this context, Am stands for amino acid, FM for functional monomer, Nº conf. for the spatial conformation number of the Amino acid-FM complex, E_{tot} represents the ground state electronic energy in kcal mol^{-1}, the ΔE represents the difference of the electronic energy in ascending order of energy between the complex and lastly, the type of interaction specifies the atoms that are in close proximity in the table. The symbols Hb denote a hydrogen bond, AmtoM indicates that an AM is complexing with an FM, and MtoAm is the reverse of AmtoM. The symbols SB denote a salt bridges. The symbols Hp denote a hydrophobics interactions. The symbols Cation-pi/pi-staquing/pi-T-sheped denote the type of π interactions interactions.

AA	FM	Nº conf.	ΔE	E_{tot}	Type of interaction
		1	0.220755082921	-2.62	5C.-.C 16C.-.H 2C.-.N 4H.-.N N.-.N 9H.-.H 5Hp Cation-pi(Amc)
		6	0.992495044313	-1.85	2C.-.C 6C.-.H C.-.N 2C.-.O 7H.-.H 2H.-.N 3H.-.O 2Hp
		2	1.507769641322	-1.34	9C.-.H C.-.O 2H.-.O 3H.-.H
		4	1.656632947814	-1.19	2C.-.O 3H.-.O 5C.-.H 4H.-.H H.-.N
		0	1.885336566952	-0.96	5C.-.H 5H.-.H C.-.C Hp
		3	2.817914648096	-0.03	
		5	2.825144182467	-0.02	
		7	2.933811702556	0.09	
		9	3.158147157001	0.32	
	acrol	2	0.000000000000	-2.09	4C.-.O 3C.-.H C.-.N 4H.-.O N.-.O O.-.O H.-.N 3H.-.H Hb(AmtoM)
		4	0.042357776471	-2.05	3C.-.N 11C.-.H 3C.-.C 2H.-.N 6H.-.H 5H.-.O N.-.O 3Hp

Continue in the next page

Table SM1: Structures of various conformations are evaluated for their energetic properties and types of intermolecular interactions. In this context, Am stands for amino acid, FM for functional monomer, N° conf. for the spatial conformation number of the Amino acid-FM complex, E_{tot} represents the ground state electronic energy in kcal mol^{-1}, the ΔE represents the difference of the electronic energy in ascending order of energy between the complex and lastly, the type of interaction specifies the atoms that are in close proximity in the table. The symbols Hb denote a hydrogen bond, AmtoM indicates that an AM is complexing with an FM, and MtoAm is the reverse of AmtoM. The symbols SB denote a salt bridges. The symbols Hp denote a hydrophobics interactions. The symbols Cation-pi/pi-staquing/pi-T-sheped denote the type of π interactions interactions.

AA	FM	N° conf.	ΔE	E_{tot}	Type of interaction
		0	0.183906606440	-1.91	9C.-.H 2C.-.N 2H.-.N 10H.-.H 2C.-.O 4H.-.O O.-.O
		9	0.712569772684	-1.38	C.-.N 5C.-.H C.-.C 4H.-.O 4H.-.H H.-.N Hp
		7	1.544050294632	-0.55	2C.-.C 7C.-.H 5H.-.H 2Hp
		1	2.080787332733	-0.01	
		3	2.084120056232	-0.01	
		8	2.156907882355	0.07	
		5	2.181695335959	0.09	
		6	2.408214107428	0.32	
	itaco	9	0.000000000000	-13.28	3O.-.O 9H.-.O 9H.-.H 6C.-.C 8C.-.O 13C.-.H 3C.-.N 3H.-.N N.-.O 6Hp Hb(MtoAm) 2SB
		0	6.310476596409	-6.97	3C.-.O 3C.-.H 2H.-.H 4H.-.O 2O.-.O Hb(AmtoM)
		8	9.161407974472	-4.12	
		6	11.346377700135	-1.93	

Continue in the next page

Table SM1: Structures of various conformations are evaluated for their energetic properties and types of intermolecular interactions. In this context, Am stands for amino acid, FM for functional monomer, N° conf. for the spatial conformation number of the Amino acid-FM complex, E_{tot} represents the ground state electronic energy in kcal mol^{-1}, the ΔE represents the difference of the electronic energy in ascending order of energy between the complex and lastly, the type of interaction specifies the atoms that are in close proximity in the table. The symbols Hb denote a hydrogen bond, AmtoM indicates that an AM is complexing with an FM, and MtoAm is the reverse of AmtoM. The symbols SB denote a salt bridges. The symbols Hp denote a hydrophobics interactions. The symbols Cation-pi/pi-staquing/pi-T-sheped denote the type of π interactions interactions.

AA	FM	N° conf.	ΔE	E_{tot}	Type of interaction
		5	11.555525500334	-1.72	5O.-.O 10H.-.O 5H.-.H 6C.-.O 9C.-.H C.-.C Hp SB
ARG	14dvb	5	0.000000000000	-2.48	10C.-.H 6H.-.N 10H.-.H 3C.-.N C.-.C Hp
		0	1.483340125838	-1.00	C.-.N 8C.-.H 11H.-.H 2H.-.N
		7	2.142780222295	-0.34	
		6	2.158968925456	-0.32	
		3	2.206967274377	-0.27	
		4	2.696181171767	0.21	
		2	2.788485951850	0.31	
	2viny	8	0.000000000000	-5.64	15C.-.C 8C.-.N 31C.-.H N.-.N 8H.-.N 16H.-.H H.-.O 15Hp
		3	0.392582812786	-5.25	25C.-.H 9C.-.C 7H.-.N 6C.-.N 14H.-.H H.-.O 9Hp
		6	4.883575173869	-0.76	15C.-.H C.-.N 10H.-.H H.-.N H.-.O
		7	5.382314771469	-0.26	
		9	5.566493371372	-0.07	

Continue in the next page

Table SM1: Structures of various conformations are evaluated for their energetic properties and types of intermolecular interactions. In this context, Am stands for amino acid, FM for functional monomer, Nº conf. for the spatial conformation number of the Amino acid-FM complex, E_{tot} represents the ground state electronic energy in kcal mol^{-1}, the ΔE represents the difference of the electronic energy in ascending order of energy between the complex and lastly, the type of interaction specifies the atoms that are in close proximity in the table. The symbols Hb denote a hydrogen bond, AmtoM indicates that an AM is complexing with an FM, and MtoAm is the reverse of AmtoM. The symbols SB denote a salt bridges. The symbols Hp denote a hydrophobics interactions. The symbols Cation-pi/pi-staquing/pi-T-sheped denote the type of π interactions interactions.

AA	FM	Nº conf.	ΔE	E_{tot}	Type of interaction
		1	5.680208208322	0.04	
		0	5.745451401482	0.11	
	acidm	4	0.000000000000	-18.35	15C.-.H C.-.C 5C.-.O 7H.-.H 10H.-.O C.-.N 3N.-.O Hp Hb(AmtoM) SB
		8	11.932508874262	-6.42	C.-.C C.-.N 5H.-.O 2C.-.O 3N.-.O 3H.-.N 4H.-.H C.-.H Hp Hb(MtoAm) 2SB
		6	14.872537644691	-3.48	19C.-.H 3H.-.N 17H.-.H 3C.-.C 4C.-.N 2N.-.O 10H.-.O 6C.-.O 3Hp 2Hb(AmtoM) 2SB
		5	18.124110279013	-0.23	erro
		3	18.418118446010	0.07	
		7	18.753346610988	0.40	
	acida	0	0.000000000000	-6.41	4C.-.H 5C.-.O 7H.-.O 2O.-.O 3H.-.H Hb(MtoAm) SB

Continue in the next page

Table SM1: Structures of various conformations are evaluated for their energetic properties and types of intermolecular interactions. In this context, Am stands for amino acid, FM for functional monomer, N° conf. for the spatial conformation number of the Amino acid-FM complex, E_{tot} represents the ground state electronic energy in kcal mol^{-1}, the ΔE represents the difference of the electronic energy in ascending order of energy between the complex and lastly, the type of interaction specifies the atoms that are in close proximity in the table. The symbols Hb denote a hydrogen bond, AmtoM indicates that an AM is complexing with an FM, and MtoAm is the reverse of AmtoM. The symbols SB denote a salt bridges. The symbols Hp denote a hydrophobics interactions. The symbols Cation-pi/pi-staquing/pi-T-sheped denote the type of π interactions interactions.

AA	FM	N° conf.	ΔE	E_{tot}	Type of interaction
		5	4.179136980262	-2.23	6C.-.C 9C.-.H C.-.N 5C.-.O 9H.-.H 2H.-.N 3H.-.O 3O.-.O 6Hp
		3	4.739885338762	-1.67	C.-.N 16C.-.H C.-.C 3H.-.N 9H.-.H H.-.O Hp
		8	5.910155673205	-0.50	
		2	6.154700255081	-0.25	
	bisac	4	0.000000000000	-8.55	6C.-.C 11C.-.N 22C.-.H 16H.-.N 27H.-.H 2C.-.O 5H.-.O N.-.O N.-.N 6Hp Hb(AmtoM) Hb(MtoAm)
		8	0.123652388795	-8.42	5C.-.N 16C.-.H 10H.-.N 14H.-.H 4C.-.O 5H.-.O N.-.O C.-.C N.-.N Hp Hb(AmtoM) Hb(MtoAm)
		3	1.436554572872	-7.11	6C.-.N 12H.-.N 11H.-.H 10C.-.H 3N.-.N C.-.O 2N.-.O 3H.-.O Hb(MtoAm) 2Hb(AmtoM)

Continue in the next page

Table SM1: Structures of various conformations are evaluated for their energetic properties and types of intermolecular interactions. In this context, Am stands for amino acid, FM for functional monomer, N° conf. for the spatial conformation number of the Amino acid-FM complex, E_{tot} represents the ground state electronic energy in kcal mol^{-1}, the ΔE represents the difference of the electronic energy in ascending order of energy between the complex and lastly, the type of interaction specifies the atoms that are in close proximity in the table. The symbols Hb denote a hydrogen bond, AmtoM indicates that an AM is complexing with an FM, and MtoAm is the reverse of AmtoM. The symbols SB denote a salt bridges. The symbols Hp denote a hydrophobics interactions. The symbols Cation-pi/pi-staquing/pi-T-sheped denote the type of π interactions interactions.

AA	FM	N° conf.	ΔE	E_{tot}	Type of interaction
		9	3.526003339537	-5.02	16H.-.H 3C.-.C 6C.-.N 15C.-.H 11H.-.N N.-.N 2C.-.O 3H.-.O 3Hp Hb(MtoAm)
		5	3.819733588857	-4.73	7C.-.N 32C.-.H 15H.-.N 25H.-.H 8C.-.C 7H.-.O 3C.-.O 2N.-.O 8Hp Hb(AmtoM)
		2	5.786361577355	-2.76	6C.-.H 5H.-.H C.-.C 3C.-.O H.-.N 5H.-.O C.-.N N.-.O Hp Hb(MtoAm)
	1ally	6	0.000000000000	-6.34	N.-.N 8C.-.N 16H.-.N 10C.-.C 39C.-.H 37H.-.H 2H.-.O 10Hp SB
		4	4.900859216996	-1.44	2C.-.C 3C.-.O 6C.-.H 8H.-.H 6H.-.O H.-.N 2Hp 2SB
		8	5.947801057420	-0.39	
		3	6.061269412313	-0.28	
		5	6.246641419129	-0.09	
		1	6.571392493961	0.23	

Continue in the next page

Table SM1: Structures of various conformations are evaluated for their energetic properties and types of intermolecular interactions. In this context, Am stands for amino acid, FM for functional monomer, N° conf. for the spatial conformation number of the Amino acid-FM complex, E_{tot} represents the ground state electronic energy in kcal mol^{-1}, the ΔE represents the difference of the electronic energy in ascending order of energy between the complex and lastly, the type of interaction specifies the atoms that are in close proximity in the table. The symbols Hb denote a hydrogen bond, AmtoM indicates that an AM is complexing with an FM, and MtoAm is the reverse of AmtoM. The symbols SB denote a salt bridges. The symbols Hp denote a hydrophobics interactions. The symbols Cation-pi/pi-staquing/pi-T-sheped denote the type of π interactions interactions.

AA	FM	N° conf.	ΔE	E_{tot}	Type of interaction
	4imid	8	0.000000000000	-6.08	4H.-.O 7C.-.N 25C.-.H 3C.-.O 2N.-.O 5C.-.C 11H.-.H 9H.-.N 5Hp Hb(AmtoM) 2SB
		9	1.198335368172	-4.88	4C.-.O 4O.-.O 2C.-.H 2H.-.O H.-.H C.-.C Hp Hb(MtoAm)
		1	2.884980338931	-3.19	3C.-.O 3H.-.O 14H.-.H 22C.-.H 6C.-.N 6C.-.C 6H.-.N N.-.N 6Hp SB
		2	3.738005163674	-2.34	2N.-.O 3H.-.O 2H.-.N 4H.-.H 3C.-.H C.-.N Hb(AmtoM) SB
		4	5.712649317520	-0.36	
		7	5.855876617498	-0.22	
	acril	1	0.000000000000	-7.29	9C.-.H 4C.-.N C.-.C 9H.-.H 8H.-.N N.-.N Hp Hb(MtoAm)
		7	1.679636221067	-5.61	4C.-.N 6C.-.H C.-.O 2N.-.O 3H.-.O 2N.-.N 8H.-.N 9H.-.H Hb(AmtoM) Hb(MtoAm)

Continue in the next page

Table SM1: Structures of various conformations are evaluated for their energetic properties and types of intermolecular interactions. In this context, Am stands for amino acid, FM for functional monomer, Nº conf. for the spatial conformation number of the Amino acid-FM complex, E_{tot} represents the ground state electronic energy in $kcal\,mol^{-1}$, the ΔE represents the difference of the electronic energy in ascending order of energy between the complex and lastly, the type of interaction specifies the atoms that are in close proximity in the table. The symbols Hb denote a hydrogen bond, AmtoM indicates that an AM is complexing with an FM, and MtoAm is the reverse of AmtoM. The symbols SB denote a salt bridges. The symbols Hp denote a hydrophobics interactions. The symbols Cation-pi/pi-staquing/pi-T-sheped denote the type of π interactions interactions.

AA	FM	Nº conf.	ΔE	E_{tot}	Type of interaction
		9	2.530101379342	-4.76	9C.-.H C.-.C 4C.-.N 15H.-.H 10H.-.N N.-.N Hp Hb(MtoAm)
		2	3.880892042758	-3.41	2C.-.N 5C.-.H 2H.-.N 3H.-.H 2N.-.O 3H.-.O C.-.O 2Hb(AmtoM)
		4	5.408714551866	-1.88	4C.-.C 3C.-.N 8C.-.H 3H.-.N 10H.-.H 5H.-.O 2C.-.O 4Hp
		5	6.174567368508	-1.11	8C.-.H 2C.-.O 4H.-.H H.-.N 2H.-.O
	alila	9	0.000000000000	-5.08	3C.-.C 13C.-.H 8H.-.N 13H.-.H 2C.-.N N.-.N 3Hp Hb(AmtoM)
		8	0.724636217419	-4.36	4C.-.C 16C.-.H 23H.-.H 13H.-.N 3C.-.N 3N.-.N 4Hp Hb(MtoAm)
		0	1.778965588489	-3.30	18C.-.H 5C.-.N 3C.-.C 22H.-.H 7H.-.N H.-.O 3Hp SB
		4	2.033065603469	-3.05	2C.-.C 12C.-.H 2C.-.N 2C.-.O 10H.-.H 3H.-.N 4H.-.O N.-.O 2Hp Hb(MtoAm) SB

Continue in the next page

Table SM1: Structures of various conformations are evaluated for their energetic properties and types of intermolecular interactions. In this context, Am stands for amino acid, FM for functional monomer, Nº conf. for the spatial conformation number of the Amino acid-FM complex, E_{tot} represents the ground state electronic energy in kcal mol^{-1}, the ΔE represents the difference of the electronic energy in ascending order of energy between the complex and lastly, the type of interaction specifies the atoms that are in close proximity in the table. The symbols Hb denote a hydrogen bond, AmtoM indicates that an AM is complexing with an FM, and MtoAm is the reverse of AmtoM. The symbols SB denote a salt bridges. The symbols Hp denote a hydrophobics interactions. The symbols Cation-pi/pi-staquing/pi-T-sheped denote the type of π interactions interactions.

AA	FM	Nº conf.	ΔE	E_{tot}	Type of interaction
		5	3.051121203502	-2.03	11C.-.H 2C.-.C 12H.-.H 2C.-.N 3H.-.N 2Hp
	estir	4	0.000000000000	-3.85	11C.-.N 17C.-.H 11H.-.H 4C.-.C 3H.-.N 4Hp 2Cation-pi(Amc)
		5	1.005008902065	-2.84	6C.-.N 15C.-.H 8H.-.H 3H.-.N Cation-pi(Amc)
		0	1.754168434702	-2.09	6C.-.H 5H.-.H C.-.C 3C.-.O 4H.-.O Hp
		6	2.405631433001	-1.44	8C.-.H 10H.-.H 2C.-.N 3H.-.N C.-.C Hp
		6	2.410539472323	-1.44	erro
		3	3.467429495343	-0.38	
		1	3.514968882751	-0.33	
		8	3.856719884243	0.01	
	1viny	1	0.000000000000	-5.47	23C.-.H 8C.-.C C.-.O 10C.-.N 13H.-.N 17H.-.H H.-.O N.-.N 8Hp

Continue in the next page

Table SM1: Structures of various conformations are evaluated for their energetic properties and types of intermolecular interactions. In this context, Am stands for amino acid, FM for functional monomer, N° conf. for the spatial conformation number of the Amino acid-FM complex, E_{tot} represents the ground state electronic energy in $kcal\,mol^{-1}$, the ΔE represents the difference of the electronic energy in ascending order of energy between the complex and lastly, the type of interaction specifies the atoms that are in close proximity in the table. The symbols Hb denote a hydrogen bond, AmtoM indicates that an AM is complexing with an FM, and MtoAm is the reverse of AmtoM. The symbols SB denote a salt bridges. The symbols Hp denote a hydrophobics interactions. The symbols Cation-pi/pi-staquing/pi-T-sheped denote the type of π interactions interactions.

AA	FM	N° conf.	ΔE	E_{tot}	Type of interaction
		9	0.205901379623	-5.26	19C.-.H 4C.-.C 10C.-.N 2N.-.N 16H.-.N 19H.-.H 4Hp Hb(AmtoM)
		6	1.313397474999	-4.15	9C.-.N 21C.-.H 4N.-.N 9H.-.N 19H.-.H 7C.-.C 7Hp Cation-pi(Amc)
		0	2.866887335367	-2.60	16C.-.H C.-.O 3H.-.N 10H.-.H 2H.-.O 3C.-.C C.-.N 3Hp
		8	5.341726057652	-0.13	
	2hydr	8	0.000000000000	-5.30	4C.-.N 18C.-.H 14H.-.H 2N.-.O 8H.-.O 2H.-.N 2C.-.O O.-.O Hb(MtoAm)
		0	0.602919311012	-4.70	4C.-.N 21C.-.H 3C.-.C 20H.-.H 6H.-.N 3C.-.O 9H.-.O N.-.O 3Hp Hb(AmtoM)
		1	2.437022457103	-2.86	3C.-.N 12C.-.H 6H.-.N 14H.-.H 11H.-.O 2C.-.O 2N.-.O Hb(AmtoM) Hb(MtoAm)

Continue in the next page

Table SM1: Structures of various conformations are evaluated for their energetic properties and types of intermolecular interactions. In this context, Am stands for amino acid, FM for functional monomer, N° conf. for the spatial conformation number of the Amino acid-FM complex, E_{tot} represents the ground state electronic energy in kcal mol^{-1}, the ΔE represents the difference of the electronic energy in ascending order of energy between the complex and lastly, the type of interaction specifies the atoms that are in close proximity in the table. The symbols Hb denote a hydrogen bond, AmtoM indicates that an AM is complexing with an FM, and MtoAm is the reverse of AmtoM. The symbols SB denote a salt bridges. The symbols Hp denote a hydrophobics interactions. The symbols Cation-pi/pi-staquing/pi-T-sheped denote the type of π interactions interactions.

AA	FM	N° conf.	ΔE	E_{tot}	Type of interaction
		6	4.163824561751	-1.14	5C.-.H C.-.N N.-.O 2H.-.O 4H.-.N 8H.-.H
	4viny	7	0.000000000000	-3.46	5C.-.N 26C.-.H 4C.-.C 8H.-.N 19H.-.H 4Hp
		4	0.993815449634	-2.46	18C.-.H 5C.-.C 7C.-.N 7H.-.N N.-.N 11H.-.H 5Hp Hb(AmtoM)
		1	2.364999821536	-1.09	2C.-.O H.-.H 2C.-.H 3H.-.O
	acrol	6	0.000000000000	-2.30	5C.-.C 10C.-.H 9H.-.H 3C.-.O 2H.-.O 2O.-.O 5Hp
		9	0.720947450410	-1.57	2C.-.N C.-.C 8C.-.H 6H.-.H 3H.-.N 2N.-.O C.-.O 2H.-.O Hp
		3	0.746752413163	-1.55	2C.-.C 11C.-.H C.-.N 9H.-.H 3H.-.N 2Hp
		1	1.791792669171	-0.50	
		5	1.996254689958	-0.30	C.-.C 3C.-.O 3C.-.H 3H.-.O 2H.-.H Hp

Continue in the next page

Table SM1: Structures of various conformations are evaluated for their energetic properties and types of intermolecular interactions. In this context, Am stands for amino acid, FM for functional monomer, N° conf. for the spatial conformation number of the Amino acid-FM complex, E_{tot} represents the ground state electronic energy in kcal mol^{-1}, the ΔE represents the difference of the electronic energy in ascending order of energy between the complex and lastly, the type of interaction specifies the atoms that are in close proximity in the table. The symbols Hb denote a hydrogen bond, AmtoM indicates that an AM is complexing with an FM, and MtoAm is the reverse of AmtoM. The symbols SB denote a salt bridges. The symbols Hp denote a hydrophobics interactions. The symbols Cation-pi/pi-staquing/pi-T-sheped denote the type of π interactions interactions.

AA	FM	N° conf.	ΔE	E_{tot}	Type of interaction
	itaco	3	0.000000000000	-16.59	13H.-.O 7C.-.O 3N.-.O O.-.O 18C.-.H 14H.-.H 7H.-.N 3C.-.C 3C.-.N 3Hp 2Hb(MtoAm) 2SB
		9	7.310564881300	-9.28	3C.-.O 6N.-.O 13H.-.O 4H.-.N 6C.-.H 8H.-.H C.-.C 2C.-.N Hp Hb(MtoAm) Hb(AmtoM) 3SB
		4	11.135804147027	-5.45	11H.-.O 15C.-.H 9H.-.H 2C.-.O 3N.-.O C.-.C C.-.N 2H.-.N Hp 3Hb(AmtoM) 2SB
		6	11.430185294626	-5.16	2C.-.H C.-.N 2N.-.O 7H.-.O 4C.-.O H.-.H Hb(AmtoM) SB
		5	11.596436924797	-4.99	10H.-.H 14C.-.H 2C.-.O 3N.-.O 9H.-.O H.-.N 3Hb(AmtoM) 3SB
ASN	14dvb	3	0.000000000000	-5.83	8C.-.O 24C.-.H 5H.-.O 12H.-.H 15C.-.C 3H.-.N 2C.-.N 15Hp Cation-pi(Amc)
		0	2.474910175677	-3.35	6C.-.C 16C.-.H 4C.-.N 2C.-.O 15H.-.H 3H.-.O 4H.-.N 6Hp

Continue in the next page

Table SM1: Structures of various conformations are evaluated for their energetic properties and types of intermolecular interactions. In this context, Am stands for amino acid, FM for functional monomer, N° conf. for the spatial conformation number of the Amino acid-FM complex, E_{tot} represents the ground state electronic energy in kcal mol^{-1}, the ΔE represents the difference of the electronic energy in ascending order of energy between the complex and lastly, the type of interaction specifies the atoms that are in close proximity in the table. The symbols Hb denote a hydrogen bond, AmtoM indicates that an AM is complexing with an FM, and MtoAm is the reverse of AmtoM. The symbols SB denote a salt bridges. The symbols Hp denote a hydrophobics interactions. The symbols Cation-pi/pi-staquing/pi-T-sheped denote the type of π interactions interactions.

AA	FM	N° conf.	ΔE	E_{tot}	Type of interaction
		9	2.527138839048	-3.30	17C.-.H 3H.-.N 8H.-.H 11C.-.O 7C.-.C 2C.-.N 2H.-.O 7Hp
		2	3.272824176675	-2.56	6C.-.H 5H.-.H 4C.-.O 5H.-.O C.-.C Hp
		8	4.880960477347	-0.95	
		7	4.892595919797	-0.94	C.-.O 2C.-.C C.-.N 7C.-.H 2H.-.O H.-.N 4H.-.H 2Hp
		1	5.652586736639	-0.18	
		4	5.701234192721	-0.13	
	2viny	2	0.000000000000	-5.19	13C.-.H 3C.-.N 2C.-.C 4H.-.N N.-.N N.-.O 8H.-.H 2C.-.O H.-.O 2Hp Hb(AmtoM) Cation-pi(Amc)
		0	0.061883846775	-5.12	6C.-.C 16C.-.H 6C.-.N 5H.-.N N.-.N N.-.O 6C.-.O 9H.-.H 4H.-.O 6Hp Hb(AmtoM)

Continue in the next page

Table SM1: Structures of various conformations are evaluated for their energetic properties and types of intermolecular interactions. In this context, Am stands for amino acid, FM for functional monomer, N° conf. for the spatial conformation number of the Amino acid-FM complex, E_{tot} represents the ground state electronic energy in kcal mol^{-1}, the ΔE represents the difference of the electronic energy in ascending order of energy between the complex and lastly, the type of interaction specifies the atoms that are in close proximity in the table. The symbols Hb denote a hydrogen bond, AmtoM indicates that an AM is complexing with an FM, and MtoAm is the reverse of AmtoM. The symbols SB denote a salt bridges. The symbols Hp denote a hydrophobics interactions. The symbols Cation-pi/pi-staquing/pi-T-sheped denote the type of π interactions interactions.

AA	FM	N° conf.	ΔE	E_{tot}	Type of interaction
		4	1.158324442101	-4.03	8C.-.N 4C.-.C 18C.-.H 2N.-.N 6H.-.N 11H.-.H H.-.O 4Hp Cation-pi(Amc)
		6	3.607290566092	-1.58	13C.-.H 2C.-.O 3C.-.C 2C.-.N 3H.-.O 2H.-.N 8H.-.H 3Hp
		1	4.947437724686	-0.24	
		3	5.123663951077	-0.06	
		5	5.189075692272	0.00	
		8	5.308195569812	0.12	
	acidm	8	0.000000000000	-7.28	6C.-.O C.-.C 3C.-.H 2O.-.O 4H.-.O H.-.N 2H.-.H Hp Hb(MtoAm)
		1	2.858357316846	-4.42	6C.-.H C.-.N 3N.-.O 2C.-.O 7H.-.O 2H.-.N 6H.-.H Hb(MtoAm) SB
		2	3.145898493132	-4.13	C.-.N C.-.O 4C.-.H 7H.-.O 2O.-.O N.-.O 4H.-.H H.-.N Hb(MtoAm) Hb(AmtoM) SB

Continue in the next page

Table SM1: Structures of various conformations are evaluated for their energetic properties and types of intermolecular interactions. In this context, Am stands for amino acid, FM for functional monomer, N° conf. for the spatial conformation number of the Amino acid-FM complex, E_{tot} represents the ground state electronic energy in $kcal\,mol^{-1}$, the ΔE represents the difference of the electronic energy in ascending order of energy between the complex and lastly, the type of interaction specifies the atoms that are in close proximity in the table. The symbols Hb denote a hydrogen bond, AmtoM indicates that an AM is complexing with an FM, and MtoAm is the reverse of AmtoM. The symbols SB denote a salt bridges. The symbols Hp denote a hydrophobics interactions. The symbols Cation-pi/pi-staquing/pi-T-sheped denote the type of π interactions interactions.

AA	FM	N° conf.	ΔE	E_{tot}	Type of interaction
		7	5.226039895094	-2.05	4C.-.O 5C.-.C 2C.-.N 10C.-.H 2H.-.N 8H.-.H 4H.-.O 2O.-.O 5Hp
		0	5.850037659488	-1.43	8C.-.O 3C.-.C 6H.-.O 4H.-.H 5C.-.H 3O.-.O 3Hp SB
		6	6.331309713688	-0.95	C.-.O 2C.-.N 5C.-.H 2H.-.O 3H.-.N 6H.-.H
		9	7.370104561680	0.09	
		3	7.374118086226	0.09	
		5	7.567798045815	0.29	
		4	19.513139901183	12.23	2C.-.O 2C.-.N 5C.-.H 3H.-.N 5H.-.H 2H.-.O
	acida	5	0.000000000000	-7.59	3C.-.O 2O.-.O 3H.-.O 2C.-.H H.-.N 2H.-.H Hb(MtoAm)
		7	3.357867655352	-4.23	2C.-.N 6C.-.H 4C.-.O 4N.-.O 9H.-.O O.-.O 2H.-.N 4H.-.H Hb(MtoAm) Hb(AmtoM) SB

Continue in the next page

Table SM1: Structures of various conformations are evaluated for their energetic properties and types of intermolecular interactions. In this context, Am stands for amino acid, FM for functional monomer, N° conf. for the spatial conformation number of the Amino acid-FM complex, E_{tot} represents the ground state electronic energy in $kcal\,mol^{-1}$, the ΔE represents the difference of the electronic energy in ascending order of energy between the complex and lastly, the type of interaction specifies the atoms that are in close proximity in the table. The symbols Hb denote a hydrogen bond, AmtoM indicates that an AM is complexing with an FM, and MtoAm is the reverse of AmtoM. The symbols SB denote a salt bridges. The symbols Hp denote a hydrophobics interactions. The symbols Cation-pi/pi-staquing/pi-T-sheped denote the type of π interactions interactions.

AA	FM	N° conf.	ΔE	E_{tot}	Type of interaction
		8	5.833731285947	-1.76	5C.-.H C.-.N 5H.-.H 2H.-.N 2N.-.O 3H.-.O SB
		4	6.224127814509	-1.37	2C.-.N 8C.-.H 2C.-.C 3H.-.N 7H.-.H O.-.O 3C.-.O N.-.O 3H.-.O 2Hp SB
		0	6.905466975349	-0.69	C.-.O 2H.-.O C.-.H H.-.H
		6	7.676298647517	0.08	
		1	8.393018693055	0.80	O.-.O
	bisac	5	0.000000000000	-10.04	6C.-.H 6H.-.O 5H.-.H 5C.-.O 2O.-.O 2H.-.N C.-.N N.-.O Hb(AmtoM) Hb(MtoAm)
		8	3.267892578106	-6.77	6C.-.O 6C.-.C 4C.-.N 21C.-.H 11H.-.O 9H.-.N 18H.-.H N.-.N N.-.O 6Hp Hb(MtoAm)
		4	8.263380126311	-1.77	O.-.O 5H.-.O 3C.-.O N.-.O 5H.-.H 5C.-.H Hb(MtoAm)
		9	9.983060965151	-0.05	
		2	10.004305471169	-0.03	

Continue in the next page

Table SM1: Structures of various conformations are evaluated for their energetic properties and types of intermolecular interactions. In this context, Am stands for amino acid, FM for functional monomer, N° conf. for the spatial conformation number of the Amino acid-FM complex, E_{tot} represents the ground state electronic energy in kcal mol^{-1}, the ΔE represents the difference of the electronic energy in ascending order of energy between the complex and lastly, the type of interaction specifies the atoms that are in close proximity in the table. The symbols Hb denote a hydrogen bond, AmtoM indicates that an AM is complexing with an FM, and MtoAm is the reverse of AmtoM. The symbols SB denote a salt bridges. The symbols Hp denote a hydrophobics interactions. The symbols Cation-pi/pi-staquing/pi-T-sheped denote the type of π interactions interactions.

AA	FM	N° conf.	ΔE	E_{tot}	Type of interaction
		0	10.189490319478	0.15	
	1ally	7	0.000000000000	-3.05	N.-.O C.-.N 4H.-.N 6H.-.O 13C.-.H 10H.-.H 2C.-.O 3C.-.C 3Hp
		2	1.129351799142	-1.92	4C.-.N 10C.-.H 6H.-.N 13H.-.H C.-.O C.-.C 2H.-.O Hp
		9	2.176205501580	-0.87	3C.-.H 3H.-.N 7H.-.H C.-.N
		8	2.845154082947	-0.20	
		3	2.867552528826	-0.18	
		1	2.934764419430	-0.11	
	4imid	6	0.000000000000	-4.15	3H.-.O 4C.-.O 7C.-.C 17C.-.H O.-.O 5C.-.N 10H.-.H 8H.-.N 3N.-.N 7Hp Hb(AmtoM) Cation-pi(Amc)
		9	0.540464557981	-3.61	6C.-.O 9H.-.O 3O.-.O 5C.-.H C.-.N C.-.C H.-.N 4H.-.H N.-.O Hp Hb(MtoAm) Hb(AmtoM) SB

Continue in the next page

Table SM1: Structures of various conformations are evaluated for their energetic properties and types of intermolecular interactions. In this context, Am stands for amino acid, FM for functional monomer, N° conf. for the spatial conformation number of the Amino acid-FM complex, E_{tot} represents the ground state electronic energy in $\mathrm{kcal\,mol^{-1}}$, the ΔE represents the difference of the electronic energy in ascending order of energy between the complex and lastly, the type of interaction specifies the atoms that are in close proximity in the table. The symbols Hb denote a hydrogen bond, AmtoM indicates that an AM is complexing with an FM, and MtoAm is the reverse of AmtoM. The symbols SB denote a salt bridges. The symbols Hp denote a hydrophobics interactions. The symbols Cation-pi/pi-staquing/pi-T-sheped denote the type of π interactions interactions.

AA	FM	N° conf.	ΔE	E_{tot}	Type of interaction
		1	2.368900289930	-1.78	4C.-.O 2N.-.O 4H.-.N 4C.-.C 8C.-.H 3C.-.N N.-.N 7H.-.H 3H.-.O 4Hp
		0	2.476342430584	-1.67	7H.-.O C.-.O 3N.-.O 4C.-.N 10C.-.H 5H.-.H 2H.-.N SB
		4	3.716625698044	-0.43	
		8	4.165291834086	0.02	
		5	4.233780177224	0.08	
	acril	2	0.000000000000	-8.16	3C.-.O 3C.-.H 2H.-.O 2H.-.H 2O.-.O H.-.N Hb(AmtoM)
		3	4.094351164399	-4.06	2C.-.O 3H.-.O N.-.O 2C.-.N H.-.N 3C.-.H 3H.-.H Hb(MtoAm)
		1	5.247772909405	-2.91	9C.-.H 4C.-.N 3C.-.O 8H.-.H 5H.-.O 6H.-.N 2C.-.C O.-.O N.-.O N.-.N 2Hp
		9	5.392984643548	-2.76	2C.-.O 2H.-.H 2C.-.H 4H.-.O N.-.O Hb(MtoAm)

Continue in the next page

Table SM1: Structures of various conformations are evaluated for their energetic properties and types of intermolecular interactions. In this context, Am stands for amino acid, FM for functional monomer, N° conf. for the spatial conformation number of the Amino acid-FM complex, E_{tot} represents the ground state electronic energy in kcal mol^{-1}, the ΔE represents the difference of the electronic energy in ascending order of energy between the complex and lastly, the type of interaction specifies the atoms that are in close proximity in the table. The symbols Hb denote a hydrogen bond, AmtoM indicates that an AM is complexing with an FM, and MtoAm is the reverse of AmtoM. The symbols SB denote a salt bridges. The symbols Hp denote a hydrophobics interactions. The symbols Cation-pi/pi-staquing/pi-T-sheped denote the type of π interactions interactions.

AA	FM	N° conf.	ΔE	E_{tot}	Type of interaction
		4	5.625211431909	-2.53	2C.-.C 10C.-.H 7H.-.H 5C.-.O 7H.-.O C.-.N 2N.-.O H.-.N 2O.-.O 2Hp Hb(AmtoM)
		8	5.726550442976	-2.43	9C.-.H 6C.-.O 4H.-.H 5H.-.O 3C.-.C N.-.O 2C.-.N 3H.-.N 2O.-.O 3Hp
		7	5.813626200960	-2.34	10C.-.H 4C.-.N 9H.-.N 13H.-.H N.-.N H.-.O Hb(MtoAm)
		6	8.217628672716	0.06	
	alila	9	0.000000000000	-4.82	3C.-.N 3C.-.H 6H.-.N 7H.-.H H.-.O N.-.N Hb(AmtoM)
		8	1.266061390657	-3.56	8C.-.H 9H.-.H 8H.-.N 3C.-.N N.-.N N.-.O 2H.-.O Hb(AmtoM) SB
		2	1.848313754761	-2.97	3C.-.N 5C.-.H 5H.-.N 6H.-.H C.-.O 3H.-.O N.-.O N.-.N Hb(MtoAm)

Continue in the next page

Table SM1: Structures of various conformations are evaluated for their energetic properties and types of intermolecular interactions. In this context, Am stands for amino acid, FM for functional monomer, N° conf. for the spatial conformation number of the Amino acid-FM complex, E_{tot} represents the ground state electronic energy in kcal mol^{-1}, the ΔE represents the difference of the electronic energy in ascending order of energy between the complex and lastly, the type of interaction specifies the atoms that are in close proximity in the table. The symbols Hb denote a hydrogen bond, AmtoM indicates that an AM is complexing with an FM, and MtoAm is the reverse of AmtoM. The symbols SB denote a salt bridges. The symbols Hp denote a hydrophobics interactions. The symbols Cation-pi/pi-staquing/pi-T-sheped denote the type of π interactions interactions.

AA	FM	N° conf.	ΔE	E_{tot}	Type of interaction
		4	3.149184430192	-1.67	2C.-.C 9C.-.H 3C.-.N 11H.-.H 4H.-.N 2H.-.O 2Hp SB
		6	4.589372078475	-0.23	4C.-.O 6H.-.O C.-.C 4C.-.H 4H.-.H C.-.N 2N.-.O H.-.N Hp SB
		3	4.717387244080	-0.11	
		0	4.855449035586	0.03	
		7	5.250394802734	0.43	
	estir	1	0.000000000000	-4.38	20C.-.H 10C.-.N 2C.-.O 3C.-.C 14H.-.H 3H.-.O 2H.-.N 3Hp Cation-pi(Amc)
		0	2.433738283596	-1.94	2C.-.C 3C.-.N 10C.-.H 9H.-.H 3H.-.O 3H.-.N C.-.O 2Hp
		4	3.560941321456	-0.81	3C.-.C 9C.-.H C.-.O 4H.-.H 2H.-.O 3Hp
		6	3.948266525131	-0.43	
		9	4.310615078140	-0.06	
		7	4.339642165390	-0.04	

Continue in the next page

Table SM1: Structures of various conformations are evaluated for their energetic properties and types of intermolecular interactions. In this context, Am stands for amino acid, FM for functional monomer, N° conf. for the spatial conformation number of the Amino acid-FM complex, E_{tot} represents the ground state electronic energy in kcal mol^{-1}, the ΔE represents the difference of the electronic energy in ascending order of energy between the complex and lastly, the type of interaction specifies the atoms that are in close proximity in the table. The symbols Hb denote a hydrogen bond, AmtoM indicates that an AM is complexing with an FM, and MtoAm is the reverse of AmtoM. The symbols SB denote a salt bridges. The symbols Hp denote a hydrophobics interactions. The symbols Cation-pi/pi-staquing/pi-T-sheped denote the type of π interactions interactions.

AA	FM	N° conf.	ΔE	E_{tot}	Type of interaction
		8	4.362496590623	-0.01	
	1viny	3	0.000000000000	-10.59	3C.-.O 4C.-.H 3H.-.O 2H.-.H C.-.N 2N.-.O 2H.-.N C.-.C Hp Hb(AmtoM)
		5	6.759446362820	-3.84	13C.-.H 4C.-.C 8C.-.N 3N.-.N 9H.-.N 2C.-.O 9H.-.H 2H.-.O 4Hp Cation-pi(Amc)
		7	7.752393996420	-2.84	4H.-.N 2C.-.O 4C.-.C 8C.-.H 2H.-.O 7H.-.H 4Hp
		4	8.759728567372	-1.84	8C.-.H 2C.-.O 2C.-.C N.-.O H.-.N 2H.-.O 4H.-.H 2Hp
		8	9.205059979465	-1.39	H.-.N 3C.-.O 4C.-.H 4H.-.H C.-.C 5H.-.O Hp
		9	9.226594873248	-1.37	N.-.O 2C.-.O 3C.-.H 2H.-.O H.-.N H.-.H
		0	9.533235169880	-1.06	8C.-.H 2C.-.O 2H.-.N 8H.-.H 3H.-.O C.-.N
		1	10.507747698162	-0.09	

Continue in the next page

Table SM1: Structures of various conformations are evaluated for their energetic properties and types of intermolecular interactions. In this context, Am stands for amino acid, FM for functional monomer, N° conf. for the spatial conformation number of the Amino acid-FM complex, E_{tot} represents the ground state electronic energy in kcal mol^{-1}, the ΔE represents the difference of the electronic energy in ascending order of energy between the complex and lastly, the type of interaction specifies the atoms that are in close proximity in the table. The symbols Hb denote a hydrogen bond, AmtoM indicates that an AM is complexing with an FM, and MtoAm is the reverse of AmtoM. The symbols SB denote a salt bridges. The symbols Hp denote a hydrophobics interactions. The symbols Cation-pi/pi-staquing/pi-T-sheped denote the type of π interactions interactions.

AA	FM	N° conf.	ΔE	E_{tot}	Type of interaction
		2	13.941210851044	3.35	3C.-.N 6C.-.H C.-.O 4H.-.N N.-.N N.-.O 4H.-.H H.-.O Hb(AmtoM)
	2hydr	9	0.000000000000	-6.51	10C.-.H 7H.-.H 9H.-.O 2O.-.O 7C.-.O N.-.O 2C.-.C 2H.-.N 2Hp Hb(MtoAm)
		1	0.411672660161	-6.10	4C.-.O 5C.-.H 5H.-.O 4H.-.H O.-.O H.-.N Hb(MtoAm)
		6	0.919893638012	-5.59	18C.-.H 3C.-.N 2N.-.O 9H.-.O 4C.-.O 5H.-.N 14H.-.H 3C.-.C O.-.O 3Hp Hb(MtoAm)
		4	0.978087595685	-5.53	12C.-.H 5C.-.O 11H.-.O 2C.-.C 10H.-.H 2O.-.O 2Hp Hb(AmtoM)
		2	2.697948916244	-3.81	7C.-.C 17C.-.H C.-.N 11C.-.O 10H.-.H 2H.-.N 7H.-.O 3O.-.O 7Hp

Continue in the next page

Table SM1: Structures of various conformations are evaluated for their energetic properties and types of intermolecular interactions. In this context, Am stands for amino acid, FM for functional monomer, N° conf. for the spatial conformation number of the Amino acid-FM complex, E_{tot} represents the ground state electronic energy in kcal mol^{-1}, the ΔE represents the difference of the electronic energy in ascending order of energy between the complex and lastly, the type of interaction specifies the atoms that are in close proximity in the table. The symbols Hb denote a hydrogen bond, AmtoM indicates that an AM is complexing with an FM, and MtoAm is the reverse of AmtoM. The symbols SB denote a salt bridges. The symbols Hp denote a hydrophobics interactions. The symbols Cation-pi/pi-staquing/pi-T-sheped denote the type of π interactions interactions.

AA	FM	N° conf.	ΔE	E_{tot}	Type of interaction
		7	2.955878603355	-3.55	8C.-.C 18C.-.H 4C.-.N 13H.-.H 5H.-.O 4C.-.O 4H.-.N 2O.-.O N.-.O 8Hp
		5	3.421187926384	-3.09	17C.-.H 4C.-.N 16H.-.H 3H.-.N 2C.-.C 7H.-.O N.-.O O.-.O 2C.-.O 2Hp Hb(AmtoM)
		0	4.290115589154	-2.22	10C.-.H 9H.-.O N.-.O 2C.-.O 9H.-.H 2H.-.N O.-.O Hb(AmtoM)
		8	6.834309595387	0.33	
	4viny	1	0.000000000000	-4.55	C.-.C 9C.-.H 4C.-.N 6H.-.N 7H.-.H N.-.N N.-.O C.-.O H.-.O Hp Hb(AmtoM)
		5	0.367307045122	-4.18	3C.-.N 2C.-.H 4H.-.N 3H.-.H N.-.N Hb(AmtoM)
		8	0.513044626310	-4.03	7C.-.H 4C.-.N 6H.-.N 6H.-.H N.-.N N.-.O 2H.-.O Hb(AmtoM)

Continue in the next page

Table SM1: Structures of various conformations are evaluated for their energetic properties and types of intermolecular interactions. In this context, Am stands for amino acid, FM for functional monomer, N° conf. for the spatial conformation number of the Amino acid-FM complex, E_{tot} represents the ground state electronic energy in kcal mol^{-1}, the ΔE represents the difference of the electronic energy in ascending order of energy between the complex and lastly, the type of interaction specifies the atoms that are in close proximity in the table. The symbols Hb denote a hydrogen bond, AmtoM indicates that an AM is complexing with an FM, and MtoAm is the reverse of AmtoM. The symbols SB denote a salt bridges. The symbols Hp denote a hydrophobics interactions. The symbols Cation-pi/pi-staquing/pi-T-sheped denote the type of π interactions interactions.

AA	FM	N° conf.	ΔE	E_{tot}	Type of interaction
		6	0.577147053908	-3.97	4C.-.N 5C.-.H 5H.-.N 4H.-.H N.-.N N.-.O C.-.C C.-.O H.-.O Hp Hb(AmtoM)
		3	0.603813637182	-3.94	9C.-.C 14C.-.H 7C.-.N 5H.-.N 11H.-.H 3C.-.O 2H.-.O 9Hp
		2	0.875100672013	-3.67	18C.-.H 3C.-.C 6C.-.O 3H.-.N 5H.-.O 12H.-.H 3C.-.N 3Hp
		4	3.505479022131	-1.04	
	acrol	2	0.000000000000	-5.62	3C.-.O 4C.-.H 2H.-.O 2H.-.H 2O.-.O Hb(AmtoM)
		7	3.270332657577	-2.35	2C.-.C 7C.-.H 2C.-.O 7H.-.H 6H.-.O C.-.N N.-.O 2Hp
		0	3.285607711126	-2.34	3C.-.O 8C.-.C 12C.-.H 4H.-.O 8H.-.H H.-.N O.-.O 8Hp
		5	3.508305807077	-2.11	6C.-.C 10C.-.H 2C.-.O C.-.N 8H.-.H 3H.-.O 2H.-.N 6Hp
		1	3.602368352033	-2.02	3C.-.H 2H.-.H 5H.-.O 2C.-.O N.-.O Hb(AmtoM)

Continue in the next page

Table SM1: Structures of various conformations are evaluated for their energetic properties and types of intermolecular interactions. In this context, Am stands for amino acid, FM for functional monomer, N° conf. for the spatial conformation number of the Amino acid-FM complex, E_{tot} represents the ground state electronic energy in kcal mol^{-1}, the ΔE represents the difference of the electronic energy in ascending order of energy between the complex and lastly, the type of interaction specifies the atoms that are in close proximity in the table. The symbols Hb denote a hydrogen bond, AmtoM indicates that an AM is complexing with an FM, and MtoAm is the reverse of AmtoM. The symbols SB denote a salt bridges. The symbols Hp denote a hydrophobics interactions. The symbols Cation-pi/pi-staquing/pi-T-sheped denote the type of π interactions interactions.

AA	FM	N° conf.	ΔE	E_{tot}	Type of interaction
		8	4.624329245801	-1.00	C.-.O 6C.-.H H.-.O 4H.-.H
		3	4.663251235400	-0.96	C.-.O C.-.C C.-.N 2C.-.H 2H.-.O 2H.-.H H.-.N Hp
		6	5.010609532389	-0.61	9C.-.H 2C.-.O 6H.-.H 2H.-.N 3H.-.O C.-.N O.-.O
		4	5.149200095546	-0.47	C.-.C 7C.-.H C.-.O 5H.-.H 2H.-.O Hp
	itaco	4	0.000000000000	-10.10	2O.-.O 3C.-.O 2N.-.O 4H.-.O C.-.C C.-.N 3C.-.H H.-.N 2H.-.H Hp Hb(MtoAm) Hb(AmtoM)
		7	1.962140885960	-8.14	3O.-.O 5C.-.O 5H.-.O 3C.-.H H.-.N 2H.-.H C.-.C Hp Hb(MtoAm)
		5	5.972079382977	-4.13	6C.-.H 3N.-.O 7H.-.O 2C.-.N 4H.-.H H.-.N C.-.O Hb(AmtoM) 2SB

Continue in the next page

Table SM1: Structures of various conformations are evaluated for their energetic properties and types of intermolecular interactions. In this context, Am stands for amino acid, FM for functional monomer, N° conf. for the spatial conformation number of the Amino acid-FM complex, E_{tot} represents the ground state electronic energy in kcal mol^{-1}, the ΔE represents the difference of the electronic energy in ascending order of energy between the complex and lastly, the type of interaction specifies the atoms that are in close proximity in the table. The symbols Hb denote a hydrogen bond, AmtoM indicates that an AM is complexing with an FM, and MtoAm is the reverse of AmtoM. The symbols SB denote a salt bridges. The symbols Hp denote a hydrophobics interactions. The symbols Cation-pi/pi-staquing/pi-T-sheped denote the type of π interactions interactions.

AA	FM	N° conf.	ΔE	E_{tot}	Type of interaction
		8	6.354054355599	-3.74	4C.-.H 4C.-.O 4H.-.H 6H.-.O C.-.N N.-.O 2O.-.O Hb(AmtoM) SB
		0	8.043895194568	-2.05	
		3	8.096148462212	-2.00	
		6	11.157353867264	1.06	
ASP	14dvb	8	0.000000000000	-4.81	22C.-.H 6H.-.O 11H.-.H 11C.-.O 12C.-.C 12Hp
		0	1.451351672951	-3.36	23C.-.H 10C.-.O 6C.-.C 13H.-.H 5H.-.O 6Hp
		5	2.796710016329	-2.02	9C.-.O 3C.-.C 6C.-.H 6H.-.O 4H.-.H 3Hp Cation-pi(Amc)
		6	3.723258648013	-1.09	C.-.C 6C.-.H 2C.-.O 6H.-.H 2H.-.N 3H.-.O C.-.N Hp
		4	4.920695862549	0.11	
		7	5.018061203235	0.20	

Continue in the next page

Table SM1: Structures of various conformations are evaluated for their energetic properties and types of intermolecular interactions. In this context, Am stands for amino acid, FM for functional monomer, N° conf. for the spatial conformation number of the Amino acid-FM complex, E_{tot} represents the ground state electronic energy in kcal mol^{-1}, the ΔE represents the difference of the electronic energy in ascending order of energy between the complex and lastly, the type of interaction specifies the atoms that are in close proximity in the table. The symbols Hb denote a hydrogen bond, AmtoM indicates that an AM is complexing with an FM, and MtoAm is the reverse of AmtoM. The symbols SB denote a salt bridges. The symbols Hp denote a hydrophobics interactions. The symbols Cation-pi/pi-staquing/pi-T-sheped denote the type of π interactions interactions.

AA	FM	N° conf.	ΔE	E_{tot}	Type of interaction
	2viny	2	0.000000000000	-9.72	5C.-.O 8C.-.H C.-.N 2N.-.O H.-.N C.-.C 4H.-.O 4H.-.H Hp Hb(AmtoM)
		5	6.336413968887	-3.38	4C.-.C 15C.-.H 7C.-.O 2C.-.N 3H.-.N N.-.O 6H.-.H 4H.-.O 4Hp
		7	6.914893594282	-2.80	2C.-.N 13C.-.H 5H.-.N N.-.N C.-.C 9H.-.H 3H.-.O Hp Hb(AmtoM)
		3	7.653701022338	-2.06	N.-.O 4C.-.O 3C.-.C 12C.-.H C.-.N 3H.-.O 2H.-.N 6H.-.H 3Hp
		1	7.683639387948	-2.03	9C.-.O 7C.-.C 15C.-.H 2N.-.O C.-.N H.-.N 5H.-.O 9H.-.H 7Hp
		0	8.785423277566	-0.93	2C.-.O 6C.-.H 2H.-.O 2H.-.H H.-.N
		4	9.186470067297	-0.53	7H.-.H 7C.-.H C.-.O 2H.-.O
	acidm	1	0.000000000000	-3.49	6C.-.O 6H.-.O 6H.-.H 5C.-.H 3O.-.O Hb(MtoAm) SB

Continue in the next page

Table SM1: Structures of various conformations are evaluated for their energetic properties and types of intermolecular interactions. In this context, Am stands for amino acid, FM for functional monomer, N° conf. for the spatial conformation number of the Amino acid-FM complex, E_{tot} represents the ground state electronic energy in kcal mol^{-1}, the ΔE represents the difference of the electronic energy in ascending order of energy between the complex and lastly, the type of interaction specifies the atoms that are in close proximity in the table. The symbols Hb denote a hydrogen bond, AmtoM indicates that an AM is complexing with an FM, and MtoAm is the reverse of AmtoM. The symbols SB denote a salt bridges. The symbols Hp denote a hydrophobics interactions. The symbols Cation-pi/pi-staquing/pi-T-sheped denote the type of π interactions interactions.

AA	FM	N° conf.	ΔE	E_{tot}	Type of interaction
		5	0.016233259945	-3.47	C.-.C 4C.-.O 7C.-.H 7H.-.O 6H.-.H 2O.-.O Hp Hb(AmtoM)
		3	0.138835946341	-3.35	C.-.C 5C.-.O 5C.-.H 4O.-.O 7H.-.O N.-.O 3H.-.H Hp 2Hb(MtoAm) SB
		2	0.831623771470	-2.65	4C.-.O 4C.-.H 4H.-.H 5H.-.O 2O.-.O Hb(AmtoM)
		6	1.798698058762	-1.69	10C.-.H 5H.-.H 5C.-.O C.-.C 2O.-.O 5H.-.O Hp
		7	2.074691042185	-1.41	8C.-.H 6H.-.H 3H.-.O 2H.-.N C.-.O
		8	3.546073608808	0.06	
		4	3.838485608458	0.35	
	acida	0	0.000000000000	-3.54	2C.-.N 11C.-.H 2C.-.C 4C.-.O 2H.-.N 9H.-.H 8H.-.O O.-.O 2Hp Hb(AmtoM) SB
		9	0.740606695333	-2.80	8C.-.H C.-.C 6H.-.O 5H.-.H 2C.-.O O.-.O Hp Hb(AmtoM) SB

Continue in the next page

Table SM1: Structures of various conformations are evaluated for their energetic properties and types of intermolecular interactions. In this context, Am stands for amino acid, FM for functional monomer, N° conf. for the spatial conformation number of the Amino acid-FM complex, E_{tot} represents the ground state electronic energy in $kcal\,mol^{-1}$, the ΔE represents the difference of the electronic energy in ascending order of energy between the complex and lastly, the type of interaction specifies the atoms that are in close proximity in the table. The symbols Hb denote a hydrogen bond, AmtoM indicates that an AM is complexing with an FM, and MtoAm is the reverse of AmtoM. The symbols SB denote a salt bridges. The symbols Hp denote a hydrophobics interactions. The symbols Cation-pi/pi-staquing/pi-T-sheped denote the type of π interactions interactions.

AA	FM	N° conf.	ΔE	E_{tot}	Type of interaction
		2	0.917925803826	-2.62	8C.-.O 6C.-.C 12C.-.H 7H.-.O 8H.-.H 2O.-.O 6Hp SB
		8	2.023674675476	-1.51	6C.-.H 4H.-.H 5H.-.O 3O.-.O 3C.-.O
		5	2.129990811708	-1.41	4C.-.H H.-.N 2H.-.H C.-.N O.-.O C.-.O N.-.O 2H.-.O Hb(AmtoM) SB
		1	2.681959180557	-0.85	2C.-.O 4C.-.H 3H.-.O 3H.-.H H.-.N
		6	2.802066032492	-0.73	C.-.N 3C.-.H H.-.N 2H.-.H N.-.O 2H.-.O SB
		4	3.711099342587	0.18	
		3	4.674878412939	1.14	5C.-.C 11C.-.H C.-.N 4C.-.O 2H.-.N 6H.-.H 6H.-.O 2O.-.O 5Hp SB
	bisac	6	0.000000000000	-10.97	8C.-.O 10C.-.H 8H.-.O 8H.-.H 5H.-.N O.-.O 2N.-.O C.-.N 2Hb(AmtoM) Hb(MtoAm)

Continue in the next page

Table SM1: Structures of various conformations are evaluated for their energetic properties and types of intermolecular interactions. In this context, Am stands for amino acid, FM for functional monomer, N° conf. for the spatial conformation number of the Amino acid-FM complex, E_{tot} represents the ground state electronic energy in kcal mol^{-1}, the ΔE represents the difference of the electronic energy in ascending order of energy between the complex and lastly, the type of interaction specifies the atoms that are in close proximity in the table. The symbols Hb denote a hydrogen bond, AmtoM indicates that an AM is complexing with an FM, and MtoAm is the reverse of AmtoM. The symbols SB denote a salt bridges. The symbols Hp denote a hydrophobics interactions. The symbols Cation-pi/pi-staquing/pi-T-sheped denote the type of π interactions interactions.

AA	FM	N° conf.	ΔE	E_{tot}	Type of interaction
		9	6.000575159301	-4.97	9C.-.O 6C.-.C 16C.-.H 8H.-.O 9H.-.H O.-.O 3H.-.N N.-.O 6Hp Hb(MtoAm)
		2	6.159021958481	-4.81	14C.-.H 5H.-.N 7H.-.H 2C.-.N 2O.-.O 6C.-.O 9H.-.O 2N.-.O C.-.C Hp Hb(AmtoM) Hb(MtoAm)
		0	10.767163070571	-0.20	
	1ally	0	0.000000000000	-15.96	11H.-.O 20C.-.H 11H.-.H 2C.-.N 2N.-.O 2H.-.N 9C.-.O 3C.-.C 3Hp Hb(MtoAm) 3SB
		4	11.239809647595	-4.72	8H.-.N 29C.-.H 6C.-.O 8H.-.O 21H.-.H 6C.-.C C.-.N 6Hp 3SB
		5	12.735963287647	-3.22	4C.-.C 5C.-.O 18C.-.H 7H.-.O 20H.-.H 2H.-.N 4Hp 2SB
		3	15.641803578498	-0.31	
		7	15.716817002976	-0.24	
		9	15.731388939193	-0.22	

Continue in the next page

Table SM1: Structures of various conformations are evaluated for their energetic properties and types of intermolecular interactions. In this context, Am stands for amino acid, FM for functional monomer, N° conf. for the spatial conformation number of the Amino acid-FM complex, E_{tot} represents the ground state electronic energy in kcal mol^{-1}, the ΔE represents the difference of the electronic energy in ascending order of energy between the complex and lastly, the type of interaction specifies the atoms that are in close proximity in the table. The symbols Hb denote a hydrogen bond, AmtoM indicates that an AM is complexing with an FM, and MtoAm is the reverse of AmtoM. The symbols SB denote a salt bridges. The symbols Hp denote a hydrophobics interactions. The symbols Cation-pi/pi-staquing/pi-T-sheped denote the type of π interactions interactions.

AA	FM	N° conf.	ΔE	E_{tot}	Type of interaction
		1	15.994468762020	0.04	
	4imid	2	0.000000000000	-10.61	13C.-.H 3H.-.O 3C.-.C 3C.-.O 5H.-.H 2C.-.N N.-.O 4H.-.N 3Hp Hb(AmtoM)
		6	0.152874702167	-10.46	3C.-.O 8C.-.H 3H.-.O 4H.-.H 4H.-.N C.-.C 2C.-.N N.-.O Hp Hb(AmtoM)
		5	1.139612432650	-9.47	2C.-.O 2N.-.O 6H.-.O O.-.O C.-.N 4C.-.H H.-.N 3H.-.H Hb(MtoAm) SB
		4	5.794976331260	-4.82	4C.-.O O.-.O 7H.-.O 10C.-.H 7H.-.H 3C.-.C 3Hp Hb(AmtoM)
		1	6.270633206039	-4.34	4C.-.O 2O.-.O 6H.-.O 3C.-.H 2H.-.H Hb(AmtoM)
		9	9.164145926695	-1.45	2C.-.O 4H.-.O N.-.O O.-.O 2C.-.N 4C.-.H 5H.-.H 2H.-.N Hb(AmtoM) SB
		0	10.535588843401	-0.08	

Continue in the next page

Table SM1: Structures of various conformations are evaluated for their energetic properties and types of intermolecular interactions. In this context, Am stands for amino acid, FM for functional monomer, N° conf. for the spatial conformation number of the Amino acid-FM complex, E_{tot} represents the ground state electronic energy in kcal mol^{-1}, the ΔE represents the difference of the electronic energy in ascending order of energy between the complex and lastly, the type of interaction specifies the atoms that are in close proximity in the table. The symbols Hb denote a hydrogen bond, AmtoM indicates that an AM is complexing with an FM, and MtoAm is the reverse of AmtoM. The symbols SB denote a salt bridges. The symbols Hp denote a hydrophobics interactions. The symbols Cation-pi/pi-staquing/pi-T-sheped denote the type of π interactions interactions.

AA	FM	N° conf.	ΔE	E_{tot}	Type of interaction
	acril	1	0.000000000000	-3.63	3C.-.O 2C.-.N 4C.-.H O.-.O 2N.-.O 5H.-.O N.-.N 3H.-.N 3H.-.H Hb(AmtoM) Hb(MtoAm)
		2	0.602588375413	-3.03	3C.-.H C.-.O N.-.N C.-.N 3H.-.N N.-.O 4H.-.O O.-.O 4H.-.H Hb(MtoAm)
		8	1.310058583551	-2.32	2C.-.N 6C.-.H N.-.N 5H.-.N O.-.O N.-.O 3H.-.O 5H.-.H
		0	2.296881452271	-1.33	8C.-.H C.-.O O.-.O 4C.-.N 3H.-.N N.-.O 6H.-.H 4H.-.O Hb(MtoAm)
		3	2.770103937531	-0.86	3C.-.O 2C.-.C 7C.-.H 4H.-.O 5H.-.H 2Hp
		6	3.469193122392	-0.16	
	alila	0	0.000000000000	-14.92	4C.-.H 8H.-.O C.-.N 2N.-.O 3C.-.O C.-.C Hp Hb(MtoAm) SB
		8	1.782031157042	-13.14	erro

Continue in the next page

Table SM1: Structures of various conformations are evaluated for their energetic properties and types of intermolecular interactions. In this context, Am stands for amino acid, FM for functional monomer, N° conf. for the spatial conformation number of the Amino acid-FM complex, E_{tot} represents the ground state electronic energy in kcal mol^{-1}, the ΔE represents the difference of the electronic energy in ascending order of energy between the complex and lastly, the type of interaction specifies the atoms that are in close proximity in the table. The symbols Hb denote a hydrogen bond, AmtoM indicates that an AM is complexing with an FM, and MtoAm is the reverse of AmtoM. The symbols SB denote a salt bridges. The symbols Hp denote a hydrophobics interactions. The symbols Cation-pi/pi-staquing/pi-T-sheped denote the type of π interactions interactions.

AA	FM	N° conf.	ΔE	E_{tot}	Type of interaction
		2	12.555868160179	-2.36	3C.-.C 3C.-.O 14C.-.H 6H.-.O 13H.-.H 3N.-.O 4C.-.N 2H.-.N 3Hp Hb(MtoAm) 2SB
		4	12.741506023778	-2.18	C.-.O 2C.-.C 8C.-.H 4H.-.O 10H.-.H 2H.-.N N.-.O 2Hp Hb(MtoAm) SB
		1	14.748764107698	-0.17	
		3	14.949586953432	0.03	
	estir	7	0.000000000000	-4.75	6C.-.N 24C.-.H 6C.-.O 7C.-.C 16H.-.H 5H.-.O 2H.-.N 7Hp Cation-pi(Amc)
		9	0.852963746899	-3.90	20C.-.H 10C.-.C 7C.-.O 13H.-.H 6H.-.O 10Hp
		4	1.866677004475	-2.88	21C.-.H 6C.-.N 4H.-.O 14H.-.H 3C.-.C 5C.-.O H.-.N 3Hp Cation-pi(Amc)
		1	4.193960596745	-0.56	C.-.N 5C.-.H H.-.O H.-.N 5H.-.H

Continue in the next page

Table SM1: Structures of various conformations are evaluated for their energetic properties and types of intermolecular interactions. In this context, Am stands for amino acid, FM for functional monomer, N° conf. for the spatial conformation number of the Amino acid-FM complex, E_{tot} represents the ground state electronic energy in kcal mol^{-1}, the ΔE represents the difference of the electronic energy in ascending order of energy between the complex and lastly, the type of interaction specifies the atoms that are in close proximity in the table. The symbols Hb denote a hydrogen bond, AmtoM indicates that an AM is complexing with an FM, and MtoAm is the reverse of AmtoM. The symbols SB denote a salt bridges. The symbols Hp denote a hydrophobics interactions. The symbols Cation-pi/pi-staquing/pi-T-sheped denote the type of π interactions interactions.

AA	FM	N° conf.	ΔE	E_{tot}	Type of interaction
		2	4.274139983190	-0.48	4C.-.H C.-.N 5H.-.H 2H.-.N H.-.O
		6	4.591246329597	-0.16	
	1viny	6	0.000000000000	-10.38	3C.-.O 4C.-.H C.-.N 2N.-.O 2H.-.N C.-.C 3H.-.O 2H.-.H Hp Hb(AmtoM)
		7	7.767347076653	-2.62	7C.-.C 11C.-.H 6C.-.O 3N.-.O 5C.-.N 4H.-.N 7H.-.H 6H.-.O 7Hp Cation-pi(Amc)
		5	8.839865119347	-1.54	3C.-.O C.-.C 6C.-.H N.-.O C.-.N H.-.N 3H.-.O 3H.-.H Hp
		1	10.174651587934	-0.21	
		2	10.305911916623	-0.08	
		3	10.351572549580	-0.03	
	2hydr	1	0.000000000000	-3.87	3C.-.H 3C.-.O 5H.-.H 5H.-.O N.-.O O.-.O H.-.N Hb(AmtoM) Hb(MtoAm)

Continue in the next page

Table SM1: Structures of various conformations are evaluated for their energetic properties and types of intermolecular interactions. In this context, Am stands for amino acid, FM for functional monomer, N° conf. for the spatial conformation number of the Amino acid-FM complex, E_{tot} represents the ground state electronic energy in kcal mol^{-1}, the ΔE represents the difference of the electronic energy in ascending order of energy between the complex and lastly, the type of interaction specifies the atoms that are in close proximity in the table. The symbols Hb denote a hydrogen bond, AmtoM indicates that an AM is complexing with an FM, and MtoAm is the reverse of AmtoM. The symbols SB denote a salt bridges. The symbols Hp denote a hydrophobics interactions. The symbols Cation-pi/pi-staquing/pi-T-sheped denote the type of π interactions interactions.

AA	FM	N° conf.	ΔE	E_{tot}	Type of interaction
		0	1.044408645986	-2.83	17C.-.H 3C.-.N 6C.-.C 6C.-.O 14H.-.H 2H.-.N 14H.-.O 2O.-.O N.-.O 6Hp
		5	1.414403514403	-2.46	3C.-.C C.-.N 8C.-.H 2H.-.N 6H.-.H 4C.-.O N.-.O 4H.-.O 3O.-.O 3Hp
		2	1.484489672627	-2.39	6C.-.H 4C.-.O 6H.-.O 7H.-.H O.-.O Hb(MtoAm)
		6	1.955139996331	-1.92	6C.-.O 5C.-.C 11C.-.H 9H.-.O 7H.-.H N.-.O 5Hp
		3	3.756783083147	-0.12	
		8	3.760805618125	-0.11	
	4viny	6	0.000000000000	-9.66	2C.-.O 5C.-.H 3H.-.O 2H.-.H C.-.N 2N.-.O H.-.N Hb(AmtoM)
		5	6.860614354385	-2.80	C.-.N 3C.-.H C.-.O 4H.-.N 2H.-.H H.-.O N.-.N N.-.O Hb(AmtoM)

Continue in the next page

Table SM1: Structures of various conformations are evaluated for their energetic properties and types of intermolecular interactions. In this context, Am stands for amino acid, FM for functional monomer, N° conf. for the spatial conformation number of the Amino acid-FM complex, E_{tot} represents the ground state electronic energy in kcal mol^{-1}, the ΔE represents the difference of the electronic energy in ascending order of energy between the complex and lastly, the type of interaction specifies the atoms that are in close proximity in the table. The symbols Hb denote a hydrogen bond, AmtoM indicates that an AM is complexing with an FM, and MtoAm is the reverse of AmtoM. The symbols SB denote a salt bridges. The symbols Hp denote a hydrophobics interactions. The symbols Cation-pi/pi-staquing/pi-T-sheped denote the type of π interactions interactions.

AA	FM	N° conf.	ΔE	E_{tot}	Type of interaction
		8	7.306733587212	-2.35	2C.-.C 6C.-.O 6C.-.H 6H.-.O 4H.-.H 2Hp
		2	9.549757009521	-0.11	
		7	9.949322351467	0.29	
	acrol	6	0.000000000000	-6.69	5C.-.H 3C.-.O C.-.C O.-.O 4H.-.O 3H.-.H Hp Hb(AmtoM)
		4	3.708538724540	-2.98	6C.-.O 5C.-.C 13C.-.H 2C.-.N 7H.-.O 8H.-.H H.-.N N.-.O O.-.O 5Hp Hb(AmtoM)
		7	3.921124456080	-2.77	3C.-.C 2C.-.N 8C.-.H 6H.-.H 3C.-.O 2H.-.N N.-.O 5H.-.O 2O.-.O 3Hp
		1	5.747244304725	-0.94	3C.-.H C.-.O 2H.-.N 3H.-.H 2H.-.O
		8	6.477717355628	-0.21	10C.-.H C.-.O 7H.-.H 3H.-.O
		2	6.596698940541	-0.09	
		5	6.618573265392	-0.07	

Continue in the next page

Table SM1: Structures of various conformations are evaluated for their energetic properties and types of intermolecular interactions. In this context, Am stands for amino acid, FM for functional monomer, N° conf. for the spatial conformation number of the Amino acid-FM complex, E_{tot} represents the ground state electronic energy in kcal mol^{-1}, the ΔE represents the difference of the electronic energy in ascending order of energy between the complex and lastly, the type of interaction specifies the atoms that are in close proximity in the table. The symbols Hb denote a hydrogen bond, AmtoM indicates that an AM is complexing with an FM, and MtoAm is the reverse of AmtoM. The symbols SB denote a salt bridges. The symbols Hp denote a hydrophobics interactions. The symbols Cation-pi/pi-staquing/pi-T-sheped denote the type of π interactions interactions.

AA	FM	N° conf.	ΔE	E_{tot}	Type of interaction
	itaco	3	0.000000000000	-7.43	5C.-.O 8H.-.O 2O.-.O 6C.-.H 6H.-.H Hb(MtoAm) Hb(AmtoM)
		2	0.490191883860	-6.94	8H.-.O C.-.C 4C.-.O 8C.-.H 4H.-.H O.-.O Hp Hb(AmtoM) SB
		9	1.680652299929	-5.75	5O.-.O 5C.-.O 6H.-.O C.-.C 5C.-.H 5H.-.H Hp Hb(MtoAm) SB
		1	1.848939158515	-5.58	N.-.O 6H.-.O 5C.-.O 3C.-.H 2H.-.H 2O.-.O Hb(MtoAm) SB
		0	2.151681165126	-5.28	4C.-.O O.-.O 6H.-.O 13C.-.H 8H.-.H C.-.C Hp Hb(AmtoM)
CYS	14dvb	0	0.000000000000	-5.51	23C.-.H 2C.-.S 13H.-.H 6C.-.C 2C.-.O 2H.-.O 6Hp
		4	2.244456980863	-3.27	11H.-.H 4C.-.S 16C.-.H 2C.-.C 2Hp
		3	2.486419359543	-3.02	3C.-.C 8C.-.O 7C.-.H 7H.-.O 4H.-.H 3Hp

Continue in the next page

Table SM1: Structures of various conformations are evaluated for their energetic properties and types of intermolecular interactions. In this context, Am stands for amino acid, FM for functional monomer, N° conf. for the spatial conformation number of the Amino acid-FM complex, E_{tot} represents the ground state electronic energy in kcal mol^{-1}, the ΔE represents the difference of the electronic energy in ascending order of energy between the complex and lastly, the type of interaction specifies the atoms that are in close proximity in the table. The symbols Hb denote a hydrogen bond, AmtoM indicates that an AM is complexing with an FM, and MtoAm is the reverse of AmtoM. The symbols SB denote a salt bridges. The symbols Hp denote a hydrophobics interactions. The symbols Cation-pi/pi-staquing/pi-T-sheped denote the type of π interactions interactions.

AA	FM	N° conf.	ΔE	E_{tot}	Type of interaction
		1	2.667753296300	-2.84	4C.-.C 12C.-.H 3C.-.O 9H.-.H H.-.N 2H.-.O 4Hp
		7	3.847531956107	-1.66	3C.-.H 2H.-.S 4H.-.H
		8	5.250658877729	-0.26	
		5	5.767923451561	0.26	4H.-.H 2H.-.O
		6	5.912533016550	0.40	
	2viny	2	0.000000000000	-9.60	5C.-.O 8C.-.H C.-.N 2N.-.O H.-.N C.-.C 4H.-.O 4H.-.H Hp Hb(AmtoM)
		8	0.356554544451	-9.24	5C.-.O 9C.-.H C.-.N 2N.-.O H.-.N C.-.C 5H.-.H 4H.-.O Hp Hb(AmtoM)
		1	5.934079300280	-3.66	2C.-.S 12C.-.H 6C.-.C 2H.-.N C.-.N 8H.-.H H.-.S C.-.O H.-.O 6Hp Cation-pi(Amc)
		9	5.940032365238	-3.66	4C.-.C 16C.-.H 4C.-.N 4H.-.N 10H.-.H 4C.-.O 3H.-.O 4Hp Cation-pi(Amc)

Continue in the next page

Table SM1: Structures of various conformations are evaluated for their energetic properties and types of intermolecular interactions. In this context, Am stands for amino acid, FM for functional monomer, N° conf. for the spatial conformation number of the Amino acid-FM complex, E_{tot} represents the ground state electronic energy in kcal mol^{-1}, the ΔE represents the difference of the electronic energy in ascending order of energy between the complex and lastly, the type of interaction specifies the atoms that are in close proximity in the table. The symbols Hb denote a hydrogen bond, AmtoM indicates that an AM is complexing with an FM, and MtoAm is the reverse of AmtoM. The symbols SB denote a salt bridges. The symbols Hp denote a hydrophobics interactions. The symbols Cation-pi/pi-staquing/pi-T-sheped denote the type of π interactions interactions.

AA	FM	N° conf.	ΔE	E_{tot}	Type of interaction
		6	7.426202114731	-2.17	4C.-.H H.-.N 2C.-.S 2H.-.H H.-.S
		3	7.557502495663	-2.04	11C.-.H 8H.-.H C.-.C 2C.-.O H.-.N 2H.-.O Hp
		7	7.582249925870	-2.01	4C.-.C 13C.-.H 3C.-.O 8H.-.H 4H.-.O 4Hp
		5	8.674744201132	-0.92	
		0	8.825291817230	-0.77	9C.-.H C.-.C C.-.O 7H.-.H H.-.N H.-.O H.-.S Hp
		4	9.503889973532	-0.09	
	acidm	0	0.000000000000	-7.01	6C.-.H C.-.C 6C.-.O 4H.-.H 7H.-.O 2O.-.O O.-.S Hp Hb(MtoAm) SB
		3	2.131938371301	-4.88	8C.-.H 9H.-.H 2H.-.S 4C.-.O 5H.-.O 2O.-.O Hb(MtoAm)
		8	2.485132813873	-4.53	2C.-.O 4H.-.O 3O.-.O C.-.H 2H.-.H Hb(MtoAm) SB

Continue in the next page

Table SM1: Structures of various conformations are evaluated for their energetic properties and types of intermolecular interactions. In this context, Am stands for amino acid, FM for functional monomer, N° conf. for the spatial conformation number of the Amino acid-FM complex, E_{tot} represents the ground state electronic energy in kcal mol^{-1}, the ΔE represents the difference of the electronic energy in ascending order of energy between the complex and lastly, the type of interaction specifies the atoms that are in close proximity in the table. The symbols Hb denote a hydrogen bond, AmtoM indicates that an AM is complexing with an FM, and MtoAm is the reverse of AmtoM. The symbols SB denote a salt bridges. The symbols Hp denote a hydrophobics interactions. The symbols Cation-pi/pi-staquing/pi-T-sheped denote the type of π interactions interactions.

AA	FM	N° conf.	ΔE	E_{tot}	Type of interaction
		1	4.717870548609	-2.30	5C.-.H 3C.-.O 6H.-.O 5H.-.H H.-.N N.-.O O.-.O Hb(AmtoM) SB
		6	6.902689628225	-0.11	
		7	6.942210843496	-0.07	
		2	6.973828949572	-0.04	
		4	7.018975851651	0.00	
		9	7.043773823610	0.03	
	acida	3	0.000000000000	-6.17	9C.-.H 3H.-.H 3C.-.C 6C.-.O 7H.-.O 2O.-.O O.-.S 3Hp Hb(MtoAm) SB
		7	4.025951543158	-2.15	7H.-.H 8C.-.H 3C.-.O 2H.-.N 5H.-.O N.-.O Hb(AmtoM) SB
		5	4.065849637865	-2.11	3C.-.N 4C.-.H H.-.N 3H.-.H 2C.-.O N.-.O 3H.-.O O.-.O Hb(AmtoM) SB

Continue in the next page

Table SM1: Structures of various conformations are evaluated for their energetic properties and types of intermolecular interactions. In this context, Am stands for amino acid, FM for functional monomer, Nº conf. for the spatial conformation number of the Amino acid-FM complex, E_{tot} represents the ground state electronic energy in kcal mol^{-1}, the ΔE represents the difference of the electronic energy in ascending order of energy between the complex and lastly, the type of interaction specifies the atoms that are in close proximity in the table. The symbols Hb denote a hydrogen bond, AmtoM indicates that an AM is complexing with an FM, and MtoAm is the reverse of AmtoM. The symbols SB denote a salt bridges. The symbols Hp denote a hydrophobics interactions. The symbols Cation-pi/pi-staquing/pi-T-sheped denote the type of π interactions interactions.

AA	FM	Nº conf.	ΔE	E_{tot}	Type of interaction
		6	4.132046433166	-2.04	3C.-.N 9C.-.H 3C.-.C 4C.-.O 7H.-.H 7H.-.O H.-.N N.-.O 3Hp SB
		0	4.367161472143	-1.81	8C.-.H 6H.-.H 2C.-.C C.-.S 5H.-.O 2O.-.S C.-.O H.-.S 2Hp
		4	6.150166168167	-0.02	
		1	6.241408550831	0.07	
	bisac	9	0.000000000000	-9.74	5C.-.O 6H.-.O C.-.N N.-.O 2H.-.N 4C.-.H 3H.-.H 2O.-.O Hb(MtoAm) Hb(AmtoM)
		1	2.604126998576	-7.14	9C.-.H 11H.-.H C.-.S 8H.-.N O.-.S 2H.-.O 4C.-.N N.-.S N.-.N 2H.-.S Hb(MtoAm)
		5	4.641735256580	-5.10	7C.-.H 4H.-.N 7H.-.H O.-.S 2C.-.O 3H.-.O N.-.O O.-.O N.-.S 3H.-.S Hb(AmtoM) Hb(MtoAm)

Continue in the next page

Table SM1: Structures of various conformations are evaluated for their energetic properties and types of intermolecular interactions. In this context, Am stands for amino acid, FM for functional monomer, N° conf. for the spatial conformation number of the Amino acid-FM complex, E_{tot} represents the ground state electronic energy in kcal mol^{-1}, the ΔE represents the difference of the electronic energy in ascending order of energy between the complex and lastly, the type of interaction specifies the atoms that are in close proximity in the table. The symbols Hb denote a hydrogen bond, AmtoM indicates that an AM is complexing with an FM, and MtoAm is the reverse of AmtoM. The symbols SB denote a salt bridges. The symbols Hp denote a hydrophobics interactions. The symbols Cation-pi/pi-staquing/pi-T-sheped denote the type of π interactions interactions.

AA	FM	N° conf.	ΔE	E_{tot}	Type of interaction
		7	5.701956054073	-4.04	12C.-.H H.-.S 11H.-.H 4C.-.N 3H.-.N 2N.-.O 5C.-.C 8C.-.O 7H.-.O 5Hp Hb(AmtoM)
		8	9.469600983104	-0.27	
		3	9.551308422717	-0.19	
	1ally	3	0.000000000000	-16.82	8C.-.H 4H.-.H 13H.-.O C.-.N 2N.-.O 10C.-.O 3C.-.C 3Hp Hb(MtoAm) 2SB
		2	14.017453210339	-2.80	10C.-.H 13H.-.H 2H.-.N H.-.O C.-.S C.-.C C.-.N 2H.-.S Hp
		9	14.858407331893	-1.96	2C.-.N 10C.-.H 11H.-.H 2H.-.N H.-.O C.-.S C.-.C 3H.-.S Hp
		1	14.886331093188	-1.93	N.-.N 5H.-.N 9H.-.H 2C.-.N 3C.-.H H.-.O
		8	15.092405616198	-1.73	N.-.N 5H.-.N 8H.-.H C.-.N 5C.-.H C.-.O 3H.-.O SB
		7	15.185229265324	-1.63	5C.-.H H.-.N 2H.-.S 9H.-.H C.-.S C.-.C Hp

Continue in the next page

Table SM1: Structures of various conformations are evaluated for their energetic properties and types of intermolecular interactions. In this context, Am stands for amino acid, FM for functional monomer, N° conf. for the spatial conformation number of the Amino acid-FM complex, E_{tot} represents the ground state electronic energy in kcal mol^{-1}, the ΔE represents the difference of the electronic energy in ascending order of energy between the complex and lastly, the type of interaction specifies the atoms that are in close proximity in the table. The symbols Hb denote a hydrogen bond, AmtoM indicates that an AM is complexing with an FM, and MtoAm is the reverse of AmtoM. The symbols SB denote a salt bridges. The symbols Hp denote a hydrophobics interactions. The symbols Cation-pi/pi-staquing/pi-T-sheped denote the type of π interactions interactions.

AA	FM	N° conf.	ΔE	E_{tot}	Type of interaction
		0	15.363635414470	-1.46	3H.-.N 14C.-.H 3C.-.O 15H.-.H H.-.S 4H.-.O SB
		6	16.520300463136	-0.30	
		5	16.609290399770	-0.21	
	4imid	3	0.000000000000	-10.33	4C.-.O 4O.-.O 4H.-.O 3H.-.H 3C.-.H C.-.C Hp Hb(MtoAm) Hb(AmtoM)
		4	0.614508377996	-9.72	5C.-.O 5C.-.H 3H.-.O 2H.-.H C.-.N 2N.-.O 2H.-.N Hb(AmtoM)
		2	0.645626179275	-9.69	4C.-.O 5C.-.H 3H.-.O 2H.-.H C.-.N 2N.-.O 2H.-.N Hb(AmtoM)
		1	6.155237114361	-4.18	4H.-.O 2O.-.O 8H.-.H 11C.-.H 2C.-.O 3C.-.N 3C.-.C 4H.-.N N.-.S C.-.S 3Hp SB
		5	7.202351025012	-3.13	7C.-.H C.-.O 2H.-.O N.-.O H.-.S 4H.-.H 2H.-.N N.-.S SB

Continue in the next page

Table SM1: Structures of various conformations are evaluated for their energetic properties and types of intermolecular interactions. In this context, Am stands for amino acid, FM for functional monomer, N° conf. for the spatial conformation number of the Amino acid-FM complex, E_{tot} represents the ground state electronic energy in $\mathrm{kcal\,mol^{-1}}$, the ΔE represents the difference of the electronic energy in ascending order of energy between the complex and lastly, the type of interaction specifies the atoms that are in close proximity in the table. The symbols Hb denote a hydrogen bond, AmtoM indicates that an AM is complexing with an FM, and MtoAm is the reverse of AmtoM. The symbols SB denote a salt bridges. The symbols Hp denote a hydrophobics interactions. The symbols Cation-pi/pi-staquing/pi-T-sheped denote the type of π interactions interactions.

AA	FM	N° conf.	ΔE	E_{tot}	Type of interaction
		8	10.302638960269	-0.03	
		0	10.413331568057	0.08	
	acril	9	0.000000000000	-5.79	C.-.S 7C.-.H 2C.-.N 2H.-.S 9H.-.H 5H.-.N N.-.S N.-.N Hb(MtoAm)
		0	1.328216004172	-4.46	13C.-.H 3C.-.C 2C.-.O 10H.-.H 6H.-.O C.-.S 3C.-.N 3H.-.N N.-.O O.-.S 3Hp Hb(MtoAm)
		2	2.302506491777	-3.49	4C.-.C 10C.-.H 9H.-.H 4C.-.O 6H.-.O O.-.O C.-.N 2N.-.O H.-.N 4Hp Hb(AmtoM)
		7	3.073522974847	-2.72	5C.-.C 3C.-.N 11C.-.H 3H.-.N 9H.-.H 4C.-.O 7H.-.O O.-.O 2N.-.O 5Hp
		6	3.134993880372	-2.66	4C.-.C 10C.-.H 8H.-.H 4C.-.O 7H.-.O C.-.N 2N.-.O H.-.N 4Hp

Continue in the next page

Table SM1: Structures of various conformations are evaluated for their energetic properties and types of intermolecular interactions. In this context, Am stands for amino acid, FM for functional monomer, N° conf. for the spatial conformation number of the Amino acid-FM complex, E_{tot} represents the ground state electronic energy in kcal mol^{-1}, the ΔE represents the difference of the electronic energy in ascending order of energy between the complex and lastly, the type of interaction specifies the atoms that are in close proximity in the table. The symbols Hb denote a hydrogen bond, AmtoM indicates that an AM is complexing with an FM, and MtoAm is the reverse of AmtoM. The symbols SB denote a salt bridges. The symbols Hp denote a hydrophobics interactions. The symbols Cation-pi/pi-staquing/pi-T-sheped denote the type of π interactions interactions.

AA	FM	N° conf.	ΔE	E_{tot}	Type of interaction
		5	3.756362933196	-2.04	14C.-.H 4C.-.C 5C.-.O 5H.-.O 8H.-.H C.-.N 4H.-.N N.-.N N.-.O O.-.O 4Hp Hb(AmtoM)
		8	3.858028835727	-1.93	2H.-.H C.-.H H.-.N O.-.S H.-.O
		1	3.885418671750	-1.91	9C.-.H C.-.C C.-.O 9H.-.H 2H.-.S H.-.O H.-.N Hp
		4	5.932362161589	0.14	
	alila	0	0.000000000000	-3.59	8C.-.H 2C.-.C C.-.O 14H.-.H 6H.-.N 2H.-.O 2C.-.N N.-.N 2Hp Hb(AmtoM) SB
		1	0.306405520337	-3.29	4C.-.H C.-.N N.-.S 5H.-.N N.-.N 10H.-.H 2H.-.S Hb(MtoAm)
		2	0.821454796289	-2.77	5C.-.C 17C.-.H 3C.-.N 4C.-.O 14H.-.H 4H.-.N 7H.-.O N.-.O 5Hp Hb(MtoAm) SB
		5	2.814258153255	-0.78	C.-.C 2C.-.O 4C.-.H C.-.N 2N.-.O H.-.N 6H.-.O 4H.-.H Hp Hb(AmtoM) SB

Continue in the next page

Table SM1: Structures of various conformations are evaluated for their energetic properties and types of intermolecular interactions. In this context, Am stands for amino acid, FM for functional monomer, N° conf. for the spatial conformation number of the Amino acid-FM complex, E_{tot} represents the ground state electronic energy in kcal mol^{-1}, the ΔE represents the difference of the electronic energy in ascending order of energy between the complex and lastly, the type of interaction specifies the atoms that are in close proximity in the table. The symbols Hb denote a hydrogen bond, AmtoM indicates that an AM is complexing with an FM, and MtoAm is the reverse of AmtoM. The symbols SB denote a salt bridges. The symbols Hp denote a hydrophobics interactions. The symbols Cation-pi/pi-staquing/pi-T-sheped denote the type of π interactions interactions.

AA	FM	N° conf.	ΔE	E_{tot}	Type of interaction
		7	3.291837305497	-0.30	
		8	3.376769863730	-0.22	
		4	3.586469232283	-0.01	
		6	3.786875494915	0.19	
	estir	0	0.000000000000	-4.41	6C.-.C 19C.-.H 10H.-.H 2C.-.S 2H.-.S 6Hp Cation-pi(Amc)
		5	0.418805470263	-3.99	8C.-.C 16C.-.H 6C.-.O 12H.-.H 4H.-.O 8Hp Cation-pi(Amc)
		8	0.478411277264	-3.93	5C.-.O 20C.-.H 6C.-.C 4H.-.O 11H.-.H C.-.S H.-.S 6Hp Cation-pi(Amc)
		4	1.689767202788	-2.72	17C.-.H 3C.-.N 9H.-.H 2C.-.C H.-.N H.-.O H.-.S 2Hp Cation-pi(Amc)
		6	2.878212021654	-1.54	C.-.S C.-.C 6C.-.H H.-.S 5H.-.H Hp
		2	4.208206949135	-0.21	

Continue in the next page

Table SM1: Structures of various conformations are evaluated for their energetic properties and types of intermolecular interactions. In this context, Am stands for amino acid, FM for functional monomer, Nº conf. for the spatial conformation number of the Amino acid-FM complex, E_{tot} represents the ground state electronic energy in kcal mol^{-1}, the ΔE represents the difference of the electronic energy in ascending order of energy between the complex and lastly, the type of interaction specifies the atoms that are in close proximity in the table. The symbols Hb denote a hydrogen bond, AmtoM indicates that an AM is complexing with an FM, and MtoAm is the reverse of AmtoM. The symbols SB denote a salt bridges. The symbols Hp denote a hydrophobics interactions. The symbols Cation-pi/pi-staquing/pi-T-sheped denote the type of π interactions interactions.

AA	FM	Nº conf.	ΔE	E_{tot}	Type of interaction
	1viny	5	0.000000000000	-4.04	11C.-.H 6H.-.H 4C.-.C 6C.-.O 2C.-.N 5H.-.N 2N.-.O 6H.-.O 4Hp Cation-pi(Amc)
		9	1.907276175322	-2.13	N.-.O H.-.N 3C.-.H 2C.-.O 4H.-.H 5H.-.O
		9	1.907276175322	-2.13	erro
		7	2.446583090195	-1.59	2C.-.O 7C.-.H 6H.-.H 2H.-.O C.-.C H.-.N Hp
		3	3.681422574878	-0.36	
		6	3.833654216320	-0.20	
		0	3.875252616760	-0.16	
		4	4.148687982456	0.11	
		2	4.181826950163	0.14	
		8	4.210499681671	0.17	
	2hydr	7	0.000000000000	-6.47	3C.-.H C.-.C 3C.-.O 5H.-.O 3H.-.H 2O.-.O Hp Hb(AmtoM)
		1	6.169898642001	-0.30	
		2	6.283319627826	-0.19	

Continue in the next page

Table SM1: Structures of various conformations are evaluated for their energetic properties and types of intermolecular interactions. In this context, Am stands for amino acid, FM for functional monomer, N° conf. for the spatial conformation number of the Amino acid-FM complex, E_{tot} represents the ground state electronic energy in kcal mol^{-1}, the ΔE represents the difference of the electronic energy in ascending order of energy between the complex and lastly, the type of interaction specifies the atoms that are in close proximity in the table. The symbols Hb denote a hydrogen bond, AmtoM indicates that an AM is complexing with an FM, and MtoAm is the reverse of AmtoM. The symbols SB denote a salt bridges. The symbols Hp denote a hydrophobics interactions. The symbols Cation-pi/pi-staquing/pi-T-sheped denote the type of π interactions interactions.

AA	FM	N° conf.	ΔE	E_{tot}	Type of interaction
		3	6.432873587830	-0.04	
		5	6.813578981931	0.34	
	4viny	4	0.000000000000	-4.65	20C.-.H 2H.-.N 10H.-.H 2C.-.S 6C.-.C 2C.-.O 2H.-.O 6Hp
		0	0.520094349197	-4.13	C.-.S 13C.-.H 8H.-.H N.-.S 3H.-.N 2C.-.N N.-.O 3C.-.C C.-.O H.-.O 3Hp
		5	0.655247786008	-4.00	17C.-.H 4C.-.C 4C.-.O 3H.-.N C.-.N 2N.-.O 6H.-.H 3H.-.O 4Hp Cation-pi(Amc)
		3	0.706118757942	-3.95	12C.-.H 2C.-.N 4H.-.N 9H.-.H 3C.-.C 2H.-.O C.-.S C.-.O 3Hp Cation-pi(Amc)
		1	0.759752329678	-3.89	7C.-.H C.-.O 4H.-.H H.-.O N.-.S 2C.-.N 3H.-.N H.-.S
		2	1.325065117537	-3.33	10C.-.O 18C.-.H 11H.-.H 7C.-.C N.-.O 2H.-.O 7Hp
		6	3.730678013998	-0.92	

Continue in the next page

Table SM1: Structures of various conformations are evaluated for their energetic properties and types of intermolecular interactions. In this context, Am stands for amino acid, FM for functional monomer, Nº conf. for the spatial conformation number of the Amino acid-FM complex, E_{tot} represents the ground state electronic energy in kcal mol^{-1}, the ΔE represents the difference of the electronic energy in ascending order of energy between the complex and lastly, the type of interaction specifies the atoms that are in close proximity in the table. The symbols Hb denote a hydrogen bond, AmtoM indicates that an AM is complexing with an FM, and MtoAm is the reverse of AmtoM. The symbols SB denote a salt bridges. The symbols Hp denote a hydrophobics interactions. The symbols Cation-pi/pi-staquing/pi-T-sheped denote the type of π interactions interactions.

AA	FM	Nº conf.	ΔE	E_{tot}	Type of interaction
		9	4.672571749900	0.02	
		7	4.747449769391	0.10	
	acrol	7	0.000000000000	-6.76	3C.-.H C.-.C 3C.-.O 2O.-.O 3H.-.O H.-.H Hp Hb(AmtoM)
		2	6.195325144174	-0.57	2C.-.C 8C.-.H 5H.-.H H.-.O H.-.S 2Hp
		1	6.267538567491	-0.49	
		3	6.306493169363	-0.45	
		8	6.353466318019	-0.41	
		4	6.480930625678	-0.28	
		9	6.485773309400	-0.27	
		0	6.782678937305	0.02	
		6	6.789247302907	0.03	
	itaco	1	0.000000000000	-10.00	4C.-.O 4O.-.O 4H.-.O 4C.-.H H.-.H C.-.C Hp Hb(MtoAm) Hb(AmtoM)

Continue in the next page

Table SM1: Structures of various conformations are evaluated for their energetic properties and types of intermolecular interactions. In this context, Am stands for amino acid, FM for functional monomer, N° conf. for the spatial conformation number of the Amino acid-FM complex, E_{tot} represents the ground state electronic energy in kcal mol^{-1}, the ΔE represents the difference of the electronic energy in ascending order of energy between the complex and lastly, the type of interaction specifies the atoms that are in close proximity in the table. The symbols Hb denote a hydrogen bond, AmtoM indicates that an AM is complexing with an FM, and MtoAm is the reverse of AmtoM. The symbols SB denote a salt bridges. The symbols Hp denote a hydrophobics interactions. The symbols Cation-pi/pi-staquing/pi-T-sheped denote the type of π interactions interactions.

AA	FM	N° conf.	ΔE	E_{tot}	Type of interaction
		0	1.752366484060	-8.25	2O.-.S 8C.-.O 13H.-.O C.-.S 3C.-.C 11C.-.H H.-.S 8H.-.H O.-.O 3Hp Hb(MtoAm)
		9	2.913142004121	-7.09	11H.-.O 4H.-.H 9C.-.O 7C.-.H 5O.-.O 3C.-.C N.-.O 3Hp Hb(AmtoM) SB
		4	4.182274724010	-5.82	5C.-.H 8H.-.O N.-.O 3O.-.O 3C.-.O 4H.-.H 2Hb(AmtoM) 2SB
		5	4.466513121518	-5.54	3C.-.H 3H.-.H 3H.-.O O.-.S C.-.O N.-.O SB
		7	10.247791303817	0.24	
GLN	14dvb	1	0.000000000000	-5.69	27C.-.H 15C.-.C 13H.-.H 2H.-.O C.-.O 3C.-.N 2H.-.N 15Hp
		8	3.207193985372	-2.49	9C.-.C 34C.-.H C.-.O 20H.-.H 2H.-.O 5C.-.N 5H.-.N 9Hp
		6	5.685542994039	-0.01	
		2	5.686517331107	-0.01	

Continue in the next page

Table SM1: Structures of various conformations are evaluated for their energetic properties and types of intermolecular interactions. In this context, Am stands for amino acid, FM for functional monomer, N° conf. for the spatial conformation number of the Amino acid-FM complex, E_{tot} represents the ground state electronic energy in $\mathrm{kcal\,mol^{-1}}$, the ΔE represents the difference of the electronic energy in ascending order of energy between the complex and lastly, the type of interaction specifies the atoms that are in close proximity in the table. The symbols Hb denote a hydrogen bond, AmtoM indicates that an AM is complexing with an FM, and MtoAm is the reverse of AmtoM. The symbols SB denote a salt bridges. The symbols Hp denote a hydrophobics interactions. The symbols Cation-pi/pi-staquing/pi-T-sheped denote the type of π interactions interactions.

AA	FM	N° conf.	ΔE	E_{tot}	Type of interaction
		4	5.987954437278	0.29	
	2viny	4	0.000000000000	-6.06	9C.-.C 7C.-.N 22C.-.H 3C.-.O N.-.N 8H.-.N 15H.-.H 3H.-.O 9Hp Hb(AmtoM)
		7	1.417693137189	-4.64	8C.-.C 21C.-.H 4C.-.N 7H.-.N 13H.-.H 8Hp
		9	1.488968891812	-4.57	8C.-.C 22C.-.H 3C.-.N 4H.-.N N.-.N 11H.-.H C.-.O H.-.O 8Hp Cation-pi(Amc)
		6	2.211871897348	-3.85	4C.-.N 10C.-.H N.-.N 4H.-.N C.-.C C.-.O 9H.-.H H.-.O Hp Hb(AmtoM)
		5	3.208248913544	-2.85	8C.-.C 17C.-.H 3C.-.N 3C.-.O N.-.O 4H.-.N 14H.-.H 4H.-.O 8Hp Cation-pi(Amc)
		2	6.107753686789	0.05	
		1	6.388413639861	0.33	

Continue in the next page

Table SM1: Structures of various conformations are evaluated for their energetic properties and types of intermolecular interactions. In this context, Am stands for amino acid, FM for functional monomer, N° conf. for the spatial conformation number of the Amino acid-FM complex, E_{tot} represents the ground state electronic energy in kcal mol^{-1}, the ΔE represents the difference of the electronic energy in ascending order of energy between the complex and lastly, the type of interaction specifies the atoms that are in close proximity in the table. The symbols Hb denote a hydrogen bond, AmtoM indicates that an AM is complexing with an FM, and MtoAm is the reverse of AmtoM. The symbols SB denote a salt bridges. The symbols Hp denote a hydrophobics interactions. The symbols Cation-pi/pi-staquing/pi-T-sheped denote the type of π interactions interactions.

AA	FM	N° conf.	ΔE	E_{tot}	Type of interaction
	acidm	3	0.000000000000	-8.76	C.-.N 8C.-.H 4H.-.N 9H.-.H 5C.-.O N.-.O 6H.-.O 2O.-.O Hb(AmtoM) Hb(MtoAm) SB
		1	3.003467166689	-5.75	2C.-.O 3O.-.O C.-.H 2H.-.O H.-.H Hb(MtoAm)
		5	3.133253399744	-5.62	4C.-.H 4H.-.H 2C.-.O 2O.-.O N.-.O 3H.-.O H.-.N Hb(MtoAm)
		0	3.705686204395	-5.05	9C.-.C 23C.-.H 4C.-.O 15H.-.H 6H.-.O 2C.-.N H.-.N N.-.O 9Hp Hb(AmtoM)
		6	5.968680939201	-2.79	4C.-.N 16C.-.H 15H.-.H 2H.-.N C.-.C 2C.-.O 7H.-.O 2N.-.O Hp Hb(AmtoM) SB
		4	6.074945310090	-2.68	8C.-.H 2C.-.C 4C.-.O 5H.-.H 6H.-.O N.-.O 2Hp Hb(AmtoM) SB

Continue in the next page

Table SM1: Structures of various conformations are evaluated for their energetic properties and types of intermolecular interactions. In this context, Am stands for amino acid, FM for functional monomer, N° conf. for the spatial conformation number of the Amino acid-FM complex, E_{tot} represents the ground state electronic energy in kcal mol^{-1}, the ΔE represents the difference of the electronic energy in ascending order of energy between the complex and lastly, the type of interaction specifies the atoms that are in close proximity in the table. The symbols Hb denote a hydrogen bond, AmtoM indicates that an AM is complexing with an FM, and MtoAm is the reverse of AmtoM. The symbols SB denote a salt bridges. The symbols Hp denote a hydrophobics interactions. The symbols Cation-pi/pi-staquing/pi-T-sheped denote the type of π interactions interactions.

AA	FM	N° conf.	ΔE	E_{tot}	Type of interaction
		2	7.468328124700	-1.29	15C.-.H 3C.-.N 14H.-.H 5H.-.N 2C.-.C 5H.-.O 2C.-.O N.-.O 2Hp SB
		8	8.732107066842	-0.02	
		9	9.277341103994	0.52	
	acida	4	0.000000000000	-5.53	4C.-.O C.-.H 4O.-.O 2H.-.O H.-.H Hb(AmtoM)
		5	2.446964948962	-3.08	7C.-.C 13C.-.H 6C.-.O 8H.-.H 10H.-.O C.-.N N.-.O H.-.N 7Hp Hb(MtoAm) SB
		7	3.392610817066	-2.13	2C.-.C 4C.-.O 9C.-.H 6H.-.H 6H.-.O 3C.-.N H.-.N 2N.-.O 3O.-.O 2Hp SB
		8	3.506685088164	-2.02	4C.-.O 12C.-.H 4C.-.C 2O.-.O 6H.-.O 8H.-.H C.-.N 2H.-.N 4Hp SB
		1	4.414462290336	-1.11	6C.-.H C.-.O C.-.C 4H.-.H 3H.-.O C.-.N H.-.N N.-.O Hp

Continue in the next page

Table SM1: Structures of various conformations are evaluated for their energetic properties and types of intermolecular interactions. In this context, Am stands for amino acid, FM for functional monomer, Nº conf. for the spatial conformation number of the Amino acid-FM complex, E_{tot} represents the ground state electronic energy in $\mathrm{kcal\,mol^{-1}}$, the ΔE represents the difference of the electronic energy in ascending order of energy between the complex and lastly, the type of interaction specifies the atoms that are in close proximity in the table. The symbols Hb denote a hydrogen bond, AmtoM indicates that an AM is complexing with an FM, and MtoAm is the reverse of AmtoM. The symbols SB denote a salt bridges. The symbols Hp denote a hydrophobics interactions. The symbols Cation-pi/pi-staquing/pi-T-sheped denote the type of π interactions interactions.

AA	FM	Nº conf.	ΔE	E_{tot}	Type of interaction
		6	4.838986367927	-0.69	3C.-.O 2C.-.H 4H.-.O 4H.-.H SB
		2	5.647077582093	0.12	
	bisac	9	0.000000000000	-6.28	4C.-.C 25C.-.H 9C.-.O 13H.-.O 18H.-.H 5C.-.N O.-.O 2N.-.N 9H.-.N 2N.-.O 4Hp 2Hb(AmtoM) Hb(MtoAm)
		1	0.893987688762	-5.39	23C.-.H 5C.-.C 8C.-.O 10H.-.N 16H.-.H 10H.-.O 5C.-.N N.-.N 2N.-.O O.-.O 5Hp Hb(MtoAm) Hb(AmtoM)
		6	2.167594987255	-4.11	11C.-.H 6H.-.H 3H.-.N 2C.-.C 7C.-.O 7H.-.O O.-.O C.-.N N.-.O 2Hp Hb(MtoAm)
		0	2.731342966535	-3.55	13C.-.H 9H.-.O 10H.-.H 2C.-.N 6C.-.O 8H.-.N 2N.-.O O.-.O N.-.N Hb(AmtoM) Hb(MtoAm)
		0	2.731342966535	-3.55	erro

Continue in the next page

Table SM1: Structures of various conformations are evaluated for their energetic properties and types of intermolecular interactions. In this context, Am stands for amino acid, FM for functional monomer, N° conf. for the spatial conformation number of the Amino acid-FM complex, E_{tot} represents the ground state electronic energy in $kcal\,mol^{-1}$, the ΔE represents the difference of the electronic energy in ascending order of energy between the complex and lastly, the type of interaction specifies the atoms that are in close proximity in the table. The symbols Hb denote a hydrogen bond, AmtoM indicates that an AM is complexing with an FM, and MtoAm is the reverse of AmtoM. The symbols SB denote a salt bridges. The symbols Hp denote a hydrophobics interactions. The symbols Cation-pi/pi-staquing/pi-T-sheped denote the type of π interactions interactions.

AA	FM	N° conf.	ΔE	E_{tot}	Type of interaction
		7	3.541651106234	-2.74	3C.-.O 4H.-.O C.-.N N.-.O 7C.-.H 6H.-.H 2C.-.C 2Hp Hb(MtoAm)
		3	5.870583336544	-0.41	7C.-.H 4C.-.O 8H.-.H 2H.-.N 2N.-.O 7H.-.O O.-.O
		5	6.246364390992	-0.03	
	1ally	7	0.000000000000	-5.61	11H.-.N 27C.-.H 6C.-.N 29H.-.H N.-.N Hb(AmtoM)
		5	0.272611554517	-5.34	3C.-.N N.-.N N.-.O 8H.-.N 9H.-.H 6C.-.H 2C.-.O 2H.-.O Hb(AmtoM)
		9	2.546976324782	-3.07	18C.-.H 2C.-.C C.-.N 17H.-.H 3H.-.N 4H.-.O 2C.-.O 2Hp
		4	3.647995468648	-1.97	18C.-.H 3C.-.C 19H.-.H 2H.-.N 2H.-.O C.-.O 3Hp
		1	4.119701116469	-1.49	2C.-.O 2C.-.N 6C.-.H 3H.-.O 4H.-.N 8H.-.H C.-.C Hp
		0	5.477817922640	-0.14	

Continue in the next page

Table SM1: Structures of various conformations are evaluated for their energetic properties and types of intermolecular interactions. In this context, Am stands for amino acid, FM for functional monomer, N° conf. for the spatial conformation number of the Amino acid-FM complex, E_{tot} represents the ground state electronic energy in kcal mol^{-1}, the ΔE represents the difference of the electronic energy in ascending order of energy between the complex and lastly, the type of interaction specifies the atoms that are in close proximity in the table. The symbols Hb denote a hydrogen bond, AmtoM indicates that an AM is complexing with an FM, and MtoAm is the reverse of AmtoM. The symbols SB denote a salt bridges. The symbols Hp denote a hydrophobics interactions. The symbols Cation-pi/pi-staquing/pi-T-sheped denote the type of π interactions interactions.

AA	FM	N° conf.	ΔE	E_{tot}	Type of interaction
		2	5.626413807872	0.01	
	4imid	1	0.000000000000	-4.46	3H.-.N C.-.N N.-.O 2C.-.O C.-.C 7C.-.H 5H.-.H 4H.-.O Hp Hb(MtoAm)
		4	2.127823979418	-2.33	13C.-.H 2C.-.O 6C.-.C 7H.-.H 3H.-.O 4C.-.N 2N.-.O 7H.-.N N.-.N 6Hp
		5	2.701262337596	-1.75	2N.-.O 5C.-.O 6H.-.O 2O.-.O C.-.C 2C.-.N 8C.-.H 2H.-.N 5H.-.H Hp SB
		6	4.468015933795	0.01	
	acril	5	0.000000000000	-6.49	4H.-.H 6C.-.H 4C.-.O 3H.-.N C.-.N 2N.-.O 7H.-.O O.-.O Hb(MtoAm) Hb(AmtoM)
		3	3.379728686887	-3.11	5C.-.H C.-.C 2C.-.N 2C.-.O 4H.-.N 4H.-.H 3H.-.O N.-.O Hp Hb(MtoAm)

Continue in the next page

Table SM1: Structures of various conformations are evaluated for their energetic properties and types of intermolecular interactions. In this context, Am stands for amino acid, FM for functional monomer, N° conf. for the spatial conformation number of the Amino acid-FM complex, E_{tot} represents the ground state electronic energy in kcal mol^{-1}, the ΔE represents the difference of the electronic energy in ascending order of energy between the complex and lastly, the type of interaction specifies the atoms that are in close proximity in the table. The symbols Hb denote a hydrogen bond, AmtoM indicates that an AM is complexing with an FM, and MtoAm is the reverse of AmtoM. The symbols SB denote a salt bridges. The symbols Hp denote a hydrophobics interactions. The symbols Cation-pi/pi-staquing/pi-T-sheped denote the type of π interactions interactions.

AA	FM	N° conf.	ΔE	E_{tot}	Type of interaction
		8	4.004734893018	-2.49	2C.-.O 3C.-.H 4H.-.O 2H.-.H N.-.O H.-.N Hb(MtoAm)
		1	4.536965844173	-1.95	7C.-.H 2C.-.C 3C.-.N 12H.-.H 8H.-.N 3C.-.O 5H.-.O N.-.N 2Hp Hb(AmtoM)
		0	6.556558688685	0.07	
	alila	6	0.000000000000	-4.14	4C.-.N 8C.-.H 9H.-.N N.-.N 11H.-.H C.-.O N.-.O 2H.-.O Hb(MtoAm) SB
		7	0.040625475425	-4.10	15C.-.H 3C.-.N 8H.-.N N.-.N 14H.-.H 2C.-.C 2Hp Hb(MtoAm) SB
		3	1.141384496264	-3.00	3C.-.C 14C.-.H C.-.N 3H.-.N 2C.-.O 5H.-.O 11H.-.H N.-.O 3Hp Hb(AmtoM)
		1	1.415966836284	-2.73	C.-.N 3C.-.H N.-.N 4H.-.N N.-.O 2H.-.O 4H.-.H Hb(AmtoM)

Continue in the next page

Table SM1: Structures of various conformations are evaluated for their energetic properties and types of intermolecular interactions. In this context, Am stands for amino acid, FM for functional monomer, N° conf. for the spatial conformation number of the Amino acid-FM complex, E_{tot} represents the ground state electronic energy in $kcal\,mol^{-1}$, the ΔE represents the difference of the electronic energy in ascending order of energy between the complex and lastly, the type of interaction specifies the atoms that are in close proximity in the table. The symbols Hb denote a hydrogen bond, AmtoM indicates that an AM is complexing with an FM, and MtoAm is the reverse of AmtoM. The symbols SB denote a salt bridges. The symbols Hp denote a hydrophobics interactions. The symbols Cation-pi/pi-staquing/pi-T-sheped denote the type of π interactions interactions.

AA	FM	N° conf.	ΔE	E_{tot}	Type of interaction
		2	1.764652323715	-2.38	3C.-.C 15C.-.H 3C.-.N 12H.-.H 3H.-.N 4H.-.O 2N.-.O C.-.O 3Hp Hb(AmtoM) SB
		8	1.995567910138	-2.15	11H.-.H 9C.-.H 4C.-.C C.-.N 2C.-.O 3H.-.O 5H.-.N N.-.N 4Hp Hb(AmtoM)
		0	2.914597401532	-1.23	5C.-.H H.-.N 4H.-.H C.-.O N.-.O 4H.-.O Hb(AmtoM) SB
	estir	0	0.000000000000	-5.50	25C.-.H 16H.-.H 10C.-.C 3H.-.O C.-.O 3C.-.N 2H.-.N 10Hp
		3	1.428066459822	-4.07	16C.-.H 5C.-.C 2C.-.O H.-.O 7H.-.H 3C.-.N H.-.N 5Hp
		6	1.522714981650	-3.98	22C.-.H 3C.-.C 2C.-.O 12H.-.H 2H.-.O 2H.-.N 4C.-.N 3Hp Cation-pi(Amc)
		8	3.107178440773	-2.39	4H.-.O 2C.-.C 11C.-.H 3C.-.O 4H.-.H 2Hp
		1	5.175799355390	-0.33	

Continue in the next page

Table SM1: Structures of various conformations are evaluated for their energetic properties and types of intermolecular interactions. In this context, Am stands for amino acid, FM for functional monomer, N° conf. for the spatial conformation number of the Amino acid-FM complex, E_{tot} represents the ground state electronic energy in kcal mol^{-1}, the ΔE represents the difference of the electronic energy in ascending order of energy between the complex and lastly, the type of interaction specifies the atoms that are in close proximity in the table. The symbols Hb denote a hydrogen bond, AmtoM indicates that an AM is complexing with an FM, and MtoAm is the reverse of AmtoM. The symbols SB denote a salt bridges. The symbols Hp denote a hydrophobics interactions. The symbols Cation-pi/pi-staquing/pi-T-sheped denote the type of π interactions interactions.

AA	FM	N° conf.	ΔE	E_{tot}	Type of interaction
		5	5.259171679812	-0.24	
		2	5.580461292261	0.08	
		7	5.792148718027	0.29	
	1viny	9	0.000000000000	-3.64	4C.-.C 14C.-.H 2C.-.O 5H.-.N 11H.-.H 2H.-.O 2C.-.N 4Hp
		4	0.594277168543	-3.05	4C.-.C 12C.-.H 3C.-.N 2C.-.O 6H.-.N 2N.-.N N.-.O 4H.-.H 2H.-.O 4Hp Cation-pi(Amc)
		6	2.883873870988	-0.76	C.-.O 4C.-.H H.-.O 2H.-.H
		8	3.722875383246	0.08	
	2hydr	8	0.000000000000	-7.08	10H.-.H 12C.-.H 6C.-.O 2N.-.O 9H.-.O 3H.-.N C.-.C O.-.O Hp Hb(AmtoM) Hb(MtoAm)
		7	2.218684526464	-4.86	2C.-.O 2C.-.H 4H.-.O 2H.-.N 3H.-.H N.-.O O.-.O Hb(MtoAm)
		1	3.546955256910	-3.53	16C.-.H 8C.-.C 3C.-.N 5C.-.O 11H.-.H 7H.-.O 2N.-.O 3H.-.N 8Hp

Continue in the next page

Table SM1: Structures of various conformations are evaluated for their energetic properties and types of intermolecular interactions. In this context, Am stands for amino acid, FM for functional monomer, N° conf. for the spatial conformation number of the Amino acid-FM complex, E_{tot} represents the ground state electronic energy in kcal mol^{-1}, the ΔE represents the difference of the electronic energy in ascending order of energy between the complex and lastly, the type of interaction specifies the atoms that are in close proximity in the table. The symbols Hb denote a hydrogen bond, AmtoM indicates that an AM is complexing with an FM, and MtoAm is the reverse of AmtoM. The symbols SB denote a salt bridges. The symbols Hp denote a hydrophobics interactions. The symbols Cation-pi/pi-staquing/pi-T-sheped denote the type of π interactions interactions.

AA	FM	N° conf.	ΔE	E_{tot}	Type of interaction
	4viny	4	0.000000000000	-3.75	21C.-.H 2C.-.O 16H.-.H 3H.-.O 3C.-.C 4C.-.N H.-.N 3Hp
		9	1.055385440138	-2.69	3C.-.C 4C.-.N 15C.-.H C.-.O 5H.-.N N.-.O N.-.N 6H.-.H H.-.O 3Hp Cation-pi(Amc)
		0	3.339268434458	-0.41	H.-.H
		8	3.883874578012	0.14	
		1	3.890024777233	0.14	
	acrol	1	0.000000000000	-1.57	2C.-.C 7C.-.H 5C.-.O 4H.-.H 6H.-.O H.-.N 2O.-.O N.-.O 2Hp
		9	0.143890301318	-1.43	C.-.C 6C.-.H C.-.O 4H.-.H H.-.N 2H.-.O Hp
		5	1.710317991014	0.14	
		0	1.786840385747	0.21	
	itaco	6	0.000000000000	-10.26	14C.-.H 3C.-.C 3C.-.O C.-.N 11H.-.H 4H.-.O 3H.-.N O.-.O N.-.O 3Hp Hb(MtoAm)

Continue in the next page

Table SM1: Structures of various conformations are evaluated for their energetic properties and types of intermolecular interactions. In this context, Am stands for amino acid, FM for functional monomer, Nº conf. for the spatial conformation number of the Amino acid-FM complex, E_{tot} represents the ground state electronic energy in kcal mol^{-1}, the ΔE represents the difference of the electronic energy in ascending order of energy between the complex and lastly, the type of interaction specifies the atoms that are in close proximity in the table. The symbols Hb denote a hydrogen bond, AmtoM indicates that an AM is complexing with an FM, and MtoAm is the reverse of AmtoM. The symbols SB denote a salt bridges. The symbols Hp denote a hydrophobics interactions. The symbols Cation-pi/pi-staquing/pi-T-sheped denote the type of π interactions interactions.

AA	FM	Nº conf.	ΔE	E_{tot}	Type of interaction
		3	0.923328024013	-9.34	8C.-.O N.-.O 2O.-.O 12H.-.O 2H.-.N 12H.-.H 13C.-.H 2C.-.C 2Hp Hb(MtoAm) SB
		5	3.457402695313	-6.80	10H.-.O 3C.-.O 3N.-.O C.-.C 17C.-.H 3C.-.N 3H.-.N 8H.-.H Hp Hb(AmtoM) SB
GLU	14dvb	0	0.000000000000	-5.51	9C.-.C 3C.-.N 31C.-.H 3H.-.N 20H.-.H 4C.-.O 3H.-.O 9Hp
		6	0.176878114639	-5.33	8C.-.O 26C.-.H 6H.-.O 11C.-.C 2C.-.N 15H.-.H 3H.-.N 11Hp Cation-pi(Amc)
		7	0.196607518067	-5.31	24C.-.H 5C.-.O 15H.-.H 7H.-.O 10C.-.C 4C.-.N H.-.N 10Hp
		2	3.768906379823	-1.74	5C.-.C 4C.-.O 13C.-.H 4H.-.O 5H.-.H 5Hp
		8	3.969418929798	-1.54	C.-.C 9C.-.H 3C.-.O 5H.-.H 5H.-.O Hp
		1	5.518437398565	0.01	

Continue in the next page

Table SM1: Structures of various conformations are evaluated for their energetic properties and types of intermolecular interactions. In this context, Am stands for amino acid, FM for functional monomer, N° conf. for the spatial conformation number of the Amino acid-FM complex, E_{tot} represents the ground state electronic energy in kcal mol^{-1}, the ΔE represents the difference of the electronic energy in ascending order of energy between the complex and lastly, the type of interaction specifies the atoms that are in close proximity in the table. The symbols Hb denote a hydrogen bond, AmtoM indicates that an AM is complexing with an FM, and MtoAm is the reverse of AmtoM. The symbols SB denote a salt bridges. The symbols Hp denote a hydrophobics interactions. The symbols Cation-pi/pi-staquing/pi-T-sheped denote the type of π interactions interactions.

AA	FM	N° conf.	ΔE	E_{tot}	Type of interaction
		9	5.537425153090	0.03	
		3	11.320283472849	5.81	9C.-.C 24C.-.H 5C.-.O 14H.-.H 4H.-.O 9Hp
	2viny	1	0.000000000000	-10.69	23C.-.H 5C.-.C 4C.-.O 4C.-.N 5H.-.N N.-.O 13H.-.H 2H.-.O 5Hp Hb(AmtoM)
		8	4.666837161546	-6.02	21C.-.H 4C.-.N N.-.N 7H.-.N 5C.-.C 3C.-.O 3H.-.O 10H.-.H 5Hp Hb(AmtoM)
		4	6.492230337317	-4.20	7C.-.C 3C.-.N 22C.-.H N.-.N 6H.-.N 13H.-.H 7Hp Hb(AmtoM)
		0	7.210357614822	-3.48	8C.-.C 20C.-.H 2C.-.N 4H.-.N N.-.N 12H.-.H C.-.O 2H.-.O 8Hp
		7	7.545360638588	-3.14	8C.-.C 21C.-.H 6C.-.O 4H.-.N 3H.-.O 10H.-.H 2C.-.N 8Hp
		9	10.725262507159	0.04	
		2	10.741524859678	0.05	

Continue in the next page

Table SM1: Structures of various conformations are evaluated for their energetic properties and types of intermolecular interactions. In this context, Am stands for amino acid, FM for functional monomer, N° conf. for the spatial conformation number of the Amino acid-FM complex, E_{tot} represents the ground state electronic energy in $kcal\,mol^{-1}$, the ΔE represents the difference of the electronic energy in ascending order of energy between the complex and lastly, the type of interaction specifies the atoms that are in close proximity in the table. The symbols Hb denote a hydrogen bond, AmtoM indicates that an AM is complexing with an FM, and MtoAm is the reverse of AmtoM. The symbols SB denote a salt bridges. The symbols Hp denote a hydrophobics interactions. The symbols Cation-pi/pi-staquing/pi-T-sheped denote the type of π interactions interactions.

AA	FM	N° conf.	ΔE	E_{tot}	Type of interaction
	acidm	2	0.000000000000	-5.99	14C.-.H 4C.-.O 2C.-.C 5H.-.O O.-.O 8H.-.H 2Hp Hb(AmtoM)
		9	0.778000607975	-5.21	11C.-.H 5C.-.C 8C.-.O 8H.-.H 8H.-.O 3O.-.O 5Hp Hb(MtoAm) SB
		4	1.077753596207	-4.92	8C.-.O 8C.-.H 3H.-.H 7H.-.O 2C.-.C 3O.-.O 2Hp Hb(MtoAm) SB
		1	3.535613717009	-2.46	2C.-.O 3O.-.O 3H.-.O 2C.-.H 3H.-.H H.-.N Hb(MtoAm) SB
		6	4.169412006876	-1.82	2C.-.N 6C.-.H 5H.-.H C.-.O 7H.-.O 2N.-.O O.-.O H.-.N SB
		5	5.259121293402	-0.73	4C.-.C 12C.-.H 5C.-.O 9H.-.H 8H.-.O 2O.-.O 4Hp
		7	6.015341282312	0.02	
		3	6.039783659412	0.05	

Continue in the next page

Table SM1: Structures of various conformations are evaluated for their energetic properties and types of intermolecular interactions. In this context, Am stands for amino acid, FM for functional monomer, N° conf. for the spatial conformation number of the Amino acid-FM complex, E_{tot} represents the ground state electronic energy in kcal mol^{-1}, the ΔE represents the difference of the electronic energy in ascending order of energy between the complex and lastly, the type of interaction specifies the atoms that are in close proximity in the table. The symbols Hb denote a hydrogen bond, AmtoM indicates that an AM is complexing with an FM, and MtoAm is the reverse of AmtoM. The symbols SB denote a salt bridges. The symbols Hp denote a hydrophobics interactions. The symbols Cation-pi/pi-staquing/pi-T-sheped denote the type of π interactions interactions.

AA	FM	N° conf.	ΔE	E_{tot}	Type of interaction
	acida	4	0.000000000000	-7.98	6C.-.H C.-.N 4C.-.O 7H.-.O 2N.-.O 2O.-.O 4H.-.H H.-.N Hb(MtoAm) 2Hb(AmtoM) SB
		9	4.613352750513	-3.37	6C.-.H 6H.-.H 6H.-.O 3C.-.O O.-.O Hb(AmtoM)
		6	4.729518816920	-3.25	4C.-.O 4O.-.O 3H.-.O C.-.H H.-.H Hb(MtoAm)
		2	5.082708496118	-2.90	2C.-.O 3O.-.O 3H.-.O 2C.-.H 2H.-.H Hb(MtoAm)
		1	6.104941155426	-1.88	5C.-.H 2C.-.O 3H.-.H H.-.N 6H.-.O N.-.O Hb(AmtoM) SB
		5	8.439842933434	0.46	
	bisac	2	0.000000000000	-7.53	18C.-.H 2C.-.N 4C.-.C 14H.-.H 5H.-.N 4C.-.O 2N.-.O 5H.-.O O.-.O 4Hp Hb(AmtoM)
		7	0.725949337613	-6.81	6C.-.H 5C.-.O 6H.-.H 4H.-.O O.-.O H.-.N Hb(AmtoM)

Continue in the next page

Table SM1: Structures of various conformations are evaluated for their energetic properties and types of intermolecular interactions. In this context, Am stands for amino acid, FM for functional monomer, N° conf. for the spatial conformation number of the Amino acid-FM complex, E_{tot} represents the ground state electronic energy in kcal mol^{-1}, the ΔE represents the difference of the electronic energy in ascending order of energy between the complex and lastly, the type of interaction specifies the atoms that are in close proximity in the table. The symbols Hb denote a hydrogen bond, AmtoM indicates that an AM is complexing with an FM, and MtoAm is the reverse of AmtoM. The symbols SB denote a salt bridges. The symbols Hp denote a hydrophobics interactions. The symbols Cation-pi/pi-staquing/pi-T-sheped denote the type of π interactions interactions.

AA	FM	N° conf.	ΔE	E_{tot}	Type of interaction
		3	2.091206656112	-5.44	3C.-.N 9C.-.H 7H.-.N 9H.-.H N.-.N 3N.-.O 4C.-.O 5H.-.O O.-.O Hb(MtoAm) Hb(AmtoM)
		0	2.582551468069	-4.95	12C.-.H 8C.-.O 8H.-.H 12H.-.O 2N.-.O O.-.O C.-.N 3H.-.N Hb(AmtoM) Hb(MtoAm)
		9	2.889689139335	-4.64	18C.-.H 4C.-.O 9H.-.O 11H.-.H 5C.-.C 3H.-.N 2C.-.N N.-.O 5Hp Hb(AmtoM)
		6	3.985807731360	-3.55	6C.-.O 10C.-.H 10H.-.H 12H.-.O 2N.-.O 5H.-.N Hb(AmtoM)
	1ally	0	0.000000000000	-4.59	2C.-.N N.-.N 12H.-.N 20C.-.H 24H.-.H H.-.O Hb(AmtoM) SB
		7	0.133513012898	-4.45	9H.-.N 22C.-.H 28H.-.H 4C.-.N N.-.N H.-.O Hb(AmtoM)
		2	1.644912362070	-2.94	23C.-.H 2C.-.C 2C.-.O 4H.-.N 21H.-.H 8H.-.O 2C.-.N 2Hp 3SB

Continue in the next page

Table SM1: Structures of various conformations are evaluated for their energetic properties and types of intermolecular interactions. In this context, Am stands for amino acid, FM for functional monomer, Nº conf. for the spatial conformation number of the Amino acid-FM complex, E_{tot} represents the ground state electronic energy in kcal mol^{-1}, the ΔE represents the difference of the electronic energy in ascending order of energy between the complex and lastly, the type of interaction specifies the atoms that are in close proximity in the table. The symbols Hb denote a hydrogen bond, AmtoM indicates that an AM is complexing with an FM, and MtoAm is the reverse of AmtoM. The symbols SB denote a salt bridges. The symbols Hp denote a hydrophobics interactions. The symbols Cation-pi/pi-staquing/pi-T-sheped denote the type of π interactions interactions.

AA	FM	Nº conf.	ΔE	E_{tot}	Type of interaction
		4	2.000918196320	-2.59	18C.-.H 2C.-.C 4C.-.O 16H.-.H 7H.-.O 3H.-.N 2C.-.N 2Hp 2SB
		5	4.643806565611	0.06	
	4imid	6	0.000000000000	-11.38	15C.-.H 3C.-.O 2N.-.O 6H.-.O C.-.C 5H.-.N 7H.-.H 2C.-.N Hp 2Hb(AmtoM) SB
		7	6.355105958234	-5.02	4C.-.O 9C.-.H 4H.-.O 2C.-.C 2C.-.N 2H.-.N N.-.O 4H.-.H 2Hp Hb(MtoAm)
		5	7.495172857828	-3.88	5C.-.O N.-.O 6H.-.O 2O.-.O 8C.-.H 2H.-.N 6H.-.H Hb(AmtoM) Hb(MtoAm) SB
		9	7.586284781289	-3.79	9H.-.O 13H.-.H 5C.-.C 19C.-.H C.-.N 5C.-.O 3N.-.O 4H.-.N 5Hp 2Hb(AmtoM) SB
		4	8.561209517123	-2.81	6C.-.O 5H.-.O 2O.-.O 2C.-.C 13C.-.H 6H.-.H 2Hp

Continue in the next page

Table SM1: Structures of various conformations are evaluated for their energetic properties and types of intermolecular interactions. In this context, Am stands for amino acid, FM for functional monomer, N° conf. for the spatial conformation number of the Amino acid-FM complex, E_{tot} represents the ground state electronic energy in kcal mol^{-1}, the ΔE represents the difference of the electronic energy in ascending order of energy between the complex and lastly, the type of interaction specifies the atoms that are in close proximity in the table. The symbols Hb denote a hydrogen bond, AmtoM indicates that an AM is complexing with an FM, and MtoAm is the reverse of AmtoM. The symbols SB denote a salt bridges. The symbols Hp denote a hydrophobics interactions. The symbols Cation-pi/pi-staquing/pi-T-sheped denote the type of π interactions interactions.

AA	FM	N° conf.	ΔE	E_{tot}	Type of interaction
		8	11.695952232335	0.32	2C.-.H H.-.N
		2	11.739751444446	0.36	
	acril	6	0.000000000000	-3.84	3C.-.C 13C.-.H 4C.-.O 13H.-.H 8H.-.O C.-.N 4H.-.N N.-.O 3Hp Hb(AmtoM)
		4	0.394104927281	-3.44	11C.-.H 2C.-.C 2C.-.O 4H.-.O 5H.-.H 2C.-.N N.-.O 2H.-.N 2Hp Hb(MtoAm)
		2	0.446875920508	-3.39	9C.-.H C.-.C 5C.-.O 5H.-.H 2H.-.N 6H.-.O C.-.N N.-.O Hp Hb(AmtoM)
		5	1.744346157410	-2.09	2C.-.O 4H.-.O 3H.-.H C.-.N N.-.O H.-.N C.-.H Hb(MtoAm)
		3	3.836818252680	-0.00	
		0	4.012037568549	0.17	
	alila	8	0.000000000000	-11.64	4C.-.C 15C.-.H 18H.-.H 4H.-.N C.-.O 2C.-.N N.-.O 3H.-.O 4Hp Hb(AmtoM) SB

Continue in the next page

Table SM1: Structures of various conformations are evaluated for their energetic properties and types of intermolecular interactions. In this context, Am stands for amino acid, FM for functional monomer, N° conf. for the spatial conformation number of the Amino acid-FM complex, E_{tot} represents the ground state electronic energy in kcal mol^{-1}, the ΔE represents the difference of the electronic energy in ascending order of energy between the complex and lastly, the type of interaction specifies the atoms that are in close proximity in the table. The symbols Hb denote a hydrogen bond, AmtoM indicates that an AM is complexing with an FM, and MtoAm is the reverse of AmtoM. The symbols SB denote a salt bridges. The symbols Hp denote a hydrophobics interactions. The symbols Cation-pi/pi-staquing/pi-T-sheped denote the type of π interactions interactions.

AA	FM	N° conf.	ΔE	E_{tot}	Type of interaction
		3	7.819037931017	-3.82	3C.-.N 5C.-.H 7H.-.N 10H.-.H N.-.N Hb(AmtoM) SB
		7	9.332310306440	-2.31	2C.-.N H.-.N N.-.O 4H.-.O 4C.-.H 4H.-.H Hb(MtoAm) SB
		5	9.762653338248	-1.88	19C.-.H 7C.-.C 4C.-.O 3H.-.N 14H.-.H 4H.-.O 7Hp SB
		6	9.931643765238	-1.71	2C.-.C 14C.-.H 15H.-.H 4H.-.O C.-.O 3C.-.N 3H.-.N N.-.O 2Hp 2SB
		4	10.745865804398	-0.89	2C.-.O 17H.-.H 9C.-.H 4H.-.O 3C.-.C C.-.N 5H.-.N 3Hp SB
		1	11.709409856644	0.07	2C.-.N 13C.-.H 5H.-.N 14H.-.H C.-.C H.-.O Hp SB
		0	11.765560542219	0.13	
	estir	8	0.000000000000	-4.98	23C.-.H 10C.-.C 15H.-.H 5H.-.O 4C.-.O 10Hp
		0	0.516661146116	-4.47	29C.-.H 5C.-.O 7C.-.C 20H.-.H 4H.-.O 4C.-.N 2H.-.N 7Hp

Continue in the next page

Table SM1: Structures of various conformations are evaluated for their energetic properties and types of intermolecular interactions. In this context, Am stands for amino acid, FM for functional monomer, N° conf. for the spatial conformation number of the Amino acid-FM complex, E_{tot} represents the ground state electronic energy in kcal mol^{-1}, the ΔE represents the difference of the electronic energy in ascending order of energy between the complex and lastly, the type of interaction specifies the atoms that are in close proximity in the table. The symbols Hb denote a hydrogen bond, AmtoM indicates that an AM is complexing with an FM, and MtoAm is the reverse of AmtoM. The symbols SB denote a salt bridges. The symbols Hp denote a hydrophobics interactions. The symbols Cation-pi/pi-staquing/pi-T-sheped denote the type of π interactions interactions.

AA	FM	N° conf.	ΔE	E_{tot}	Type of interaction
		5	3.341189192672	-1.64	18C.-.H 6C.-.O 14H.-.H C.-.N 2C.-.C 2H.-.N H.-.O 2Hp
		3	3.403784449253	-1.58	11C.-.H C.-.N C.-.C 9H.-.H 2H.-.N 2H.-.O Hp
		4	5.043425694648	0.06	
		9	5.045873920652	0.06	
		1	5.089324254007	0.11	
	1viny	0	0.000000000000	-10.20	5C.-.H 2C.-.O 5H.-.H 2H.-.O 4H.-.N 2C.-.N N.-.O Hb(AmtoM)
		3	0.056421562643	-10.15	C.-.C 6C.-.H 2C.-.O 5H.-.H 2H.-.O 2C.-.N 4H.-.N N.-.O Hp Hb(AmtoM)
		9	0.278275263986	-9.93	6C.-.H 2C.-.O 2C.-.N 4H.-.N N.-.O 5H.-.H 2H.-.O Hb(AmtoM) Cation-pi(Amc)

Continue in the next page

Table SM1: Structures of various conformations are evaluated for their energetic properties and types of intermolecular interactions. In this context, Am stands for amino acid, FM for functional monomer, N° conf. for the spatial conformation number of the Amino acid-FM complex, E_{tot} represents the ground state electronic energy in kcal mol^{-1}, the ΔE represents the difference of the electronic energy in ascending order of energy between the complex and lastly, the type of interaction specifies the atoms that are in close proximity in the table. The symbols Hb denote a hydrogen bond, AmtoM indicates that an AM is complexing with an FM, and MtoAm is the reverse of AmtoM. The symbols SB denote a salt bridges. The symbols Hp denote a hydrophobics interactions. The symbols Cation-pi/pi-staquing/pi-T-sheped denote the type of π interactions interactions.

AA	FM	N° conf.	ΔE	E_{tot}	Type of interaction
		8	6.877174318328	-3.33	6C.-.H C.-.C 2C.-.N C.-.O 6H.-.N 5H.-.H N.-.N H.-.O Hp Hb(AmtoM)
		7	9.473881609390	-0.73	N.-.O 2C.-.O 2C.-.H H.-.H 2H.-.O
		1	9.614762216734	-0.59	3C.-.H 3H.-.O 2H.-.H C.-.O
		4	10.137835461258	-0.07	
		6	10.208478998729	0.00	
	2hydr	5	0.000000000000	-6.04	5C.-.H 3C.-.O 6H.-.H 6H.-.O O.-.O Hb(AmtoM)
		4	1.732521632429	-4.31	C.-.N 10C.-.H 9H.-.H H.-.N 3C.-.O 6H.-.O O.-.O Hb(AmtoM)
		8	2.103595328511	-3.94	5H.-.O 2C.-.O 2O.-.O C.-.H 2Hb(MtoAm)
		7	3.594244650611	-2.45	C.-.N 7C.-.H 4C.-.O N.-.O 6H.-.O 3H.-.N 10H.-.H Hb(AmtoM)

Continue in the next page

Table SM1: Structures of various conformations are evaluated for their energetic properties and types of intermolecular interactions. In this context, Am stands for amino acid, FM for functional monomer, Nº conf. for the spatial conformation number of the Amino acid-FM complex, E_{tot} represents the ground state electronic energy in kcal mol^{-1}, the ΔE represents the difference of the electronic energy in ascending order of energy between the complex and lastly, the type of interaction specifies the atoms that are in close proximity in the table. The symbols Hb denote a hydrogen bond, AmtoM indicates that an AM is complexing with an FM, and MtoAm is the reverse of AmtoM. The symbols SB denote a salt bridges. The symbols Hp denote a hydrophobics interactions. The symbols Cation-pi/pi-staquing/pi-T-sheped denote the type of π interactions interactions.

AA	FM	Nº conf.	ΔE	E_{tot}	Type of interaction
		6	4.308994679244	-1.73	2C.-.H 8H.-.H N.-.O 4H.-.O H.-.N Hb(AmtoM)
		9	5.396507698169	-0.64	3C.-.C 11C.-.H 2C.-.O 11H.-.H H.-.N 3H.-.O 3Hp
		3	6.092444611835	0.05	
		2	6.344571667782	0.30	
	4viny	0	0.000000000000	-9.01	5C.-.C 24C.-.H 2C.-.O 3C.-.N 3H.-.N N.-.O 12H.-.H 2H.-.O 5Hp Hb(AmtoM) Cation-pi(Amc)
		4	4.342528344828	-4.67	16C.-.C 26C.-.H C.-.N H.-.N 12H.-.H 5C.-.O 6H.-.O 16Hp Cation-pi(Amc)
		8	4.363960836926	-4.65	12C.-.C 21C.-.H 7C.-.O 12H.-.H 6H.-.O C.-.N 2H.-.N 12Hp
		2	8.400703656561	-0.61	2C.-.O 3C.-.H 3H.-.O 2H.-.H
		1	9.005925773160	-0.01	
		6	9.025539645692	0.01	

Continue in the next page

Table SM1: Structures of various conformations are evaluated for their energetic properties and types of intermolecular interactions. In this context, Am stands for amino acid, FM for functional monomer, N° conf. for the spatial conformation number of the Amino acid-FM complex, E_{tot} represents the ground state electronic energy in kcal mol^{-1}, the ΔE represents the difference of the electronic energy in ascending order of energy between the complex and lastly, the type of interaction specifies the atoms that are in close proximity in the table. The symbols Hb denote a hydrogen bond, AmtoM indicates that an AM is complexing with an FM, and MtoAm is the reverse of AmtoM. The symbols SB denote a salt bridges. The symbols Hp denote a hydrophobics interactions. The symbols Cation-pi/pi-staquing/pi-T-sheped denote the type of π interactions interactions.

AA	FM	N° conf.	ΔE	E_{tot}	Type of interaction
	acrol	2	0.000000000000	-2.59	2C.-.N 4C.-.H H.-.N 4H.-.H 5H.-.O C.-.O N.-.O Hb(AmtoM)
		1	0.227925842466	-2.37	6C.-.C 3C.-.N 14C.-.H 5C.-.O 10H.-.H 7H.-.O 2H.-.N N.-.O O.-.O 6Hp
		4	0.237900302716	-2.36	2C.-.C 10C.-.H 8H.-.H C.-.N C.-.O 4H.-.O N.-.O 2Hp Hb(AmtoM)
		3	0.339000451972	-2.26	C.-.N 2C.-.H C.-.O 4H.-.O N.-.O H.-.N 3H.-.H Hb(AmtoM)
		9	1.908322340430	-0.69	C.-.C 5C.-.H C.-.O 4H.-.O O.-.O 2H.-.H Hp
		8	2.172293763541	-0.42	2H.-.O C.-.O H.-.H
		0	2.663458366287	0.07	
		7	2.729410416874	0.13	
	itaco	0	0.000000000000	-6.25	C.-.C 4C.-.O 5C.-.H 7H.-.O 4H.-.H 2O.-.O Hp Hb(MtoAm)

Continue in the next page

Table SM1: Structures of various conformations are evaluated for their energetic properties and types of intermolecular interactions. In this context, Am stands for amino acid, FM for functional monomer, Nº conf. for the spatial conformation number of the Amino acid-FM complex, E_{tot} represents the ground state electronic energy in $kcal\,mol^{-1}$, the ΔE represents the difference of the electronic energy in ascending order of energy between the complex and lastly, the type of interaction specifies the atoms that are in close proximity in the table. The symbols Hb denote a hydrogen bond, AmtoM indicates that an AM is complexing with an FM, and MtoAm is the reverse of AmtoM. The symbols SB denote a salt bridges. The symbols Hp denote a hydrophobics interactions. The symbols Cation-pi/pi-staquing/pi-T-sheped denote the type of π interactions interactions.

AA	FM	Nº conf.	ΔE	E_{tot}	Type of interaction
		6	0.036999234780	-6.21	C.-.C 4C.-.O 4C.-.H 7H.-.O 4H.-.H 2O.-.O Hp Hb(MtoAm)
		8	0.565463779921	-5.68	2C.-.O 3O.-.O C.-.H 2H.-.O H.-.H Hb(MtoAm)
		1	1.310061924117	-4.94	5C.-.N 15C.-.H 8H.-.O 9H.-.H 3H.-.N 3C.-.O N.-.O O.-.O Hb(MtoAm) Hb(AmtoM) 2SB
		2	6.771539881515	0.52	
GLY	14dvb	7	0.000000000000	-4.39	25C.-.H 8C.-.C 5C.-.O 4H.-.O 10H.-.H 6C.-.N H.-.N 8Hp Cation-pi(Amc)
		0	0.076240258060	-4.32	23C.-.H 3C.-.N 10C.-.C 13H.-.H 2H.-.N 8C.-.O 3H.-.O 10Hp
		8	3.692187878190	-0.70	3C.-.H C.-.C 6H.-.H H.-.N Hp
		9	4.323936704150	-0.07	
		6	4.436690293001	0.05	
		2	4.453332996363	0.06	
		3	6.893025209386	2.50	

Continue in the next page

Table SM1: Structures of various conformations are evaluated for their energetic properties and types of intermolecular interactions. In this context, Am stands for amino acid, FM for functional monomer, N° conf. for the spatial conformation number of the Amino acid-FM complex, E_{tot} represents the ground state electronic energy in kcal mol^{-1}, the ΔE represents the difference of the electronic energy in ascending order of energy between the complex and lastly, the type of interaction specifies the atoms that are in close proximity in the table. The symbols Hb denote a hydrogen bond, AmtoM indicates that an AM is complexing with an FM, and MtoAm is the reverse of AmtoM. The symbols SB denote a salt bridges. The symbols Hp denote a hydrophobics interactions. The symbols Cation-pi/pi-staquing/pi-T-sheped denote the type of π interactions interactions.

AA	FM	N° conf.	ΔE	E_{tot}	Type of interaction
		5	56.391960647506	52.00	2C.-.O 7C.-.H 7H.-.H 3H.-.O C.-.C Hp
	2viny	5	0.000000000000	-9.14	5C.-.O 8C.-.H C.-.N 2N.-.O H.-.N C.-.C 4H.-.H 3H.-.O Hp Hb(AmtoM)
		7	4.684678377793	-4.46	4C.-.C 12C.-.H 6C.-.N 5H.-.N N.-.N N.-.O 2C.-.O 8H.-.H H.-.O 4Hp Hb(AmtoM)
		3	6.400100844521	-2.74	6C.-.C 10C.-.H 5C.-.O 3H.-.N 2C.-.N N.-.O 3H.-.O 6H.-.H 6Hp
		4	6.740844083414	-2.40	2C.-.C 14C.-.H 6C.-.N 2H.-.N N.-.N 7H.-.H 2Hp Cation-pi(Amc)
		9	6.794153441352	-2.35	12C.-.H 3H.-.N 2C.-.C 7H.-.H 2C.-.N 2Hp Cation-pi(Amc)
		2	9.170008687687	0.03	
		0	9.171993244004	0.03	
		8	9.205598383564	0.06	

Continue in the next page

Table SM1: Structures of various conformations are evaluated for their energetic properties and types of intermolecular interactions. In this context, Am stands for amino acid, FM for functional monomer, N° conf. for the spatial conformation number of the Amino acid-FM complex, E_{tot} represents the ground state electronic energy in $kcal\,mol^{-1}$, the ΔE represents the difference of the electronic energy in ascending order of energy between the complex and lastly, the type of interaction specifies the atoms that are in close proximity in the table. The symbols Hb denote a hydrogen bond, AmtoM indicates that an AM is complexing with an FM, and MtoAm is the reverse of AmtoM. The symbols SB denote a salt bridges. The symbols Hp denote a hydrophobics interactions. The symbols Cation-pi/pi-staquing/pi-T-sheped denote the type of π interactions interactions.

AA	FM	N° conf.	ΔE	E_{tot}	Type of interaction
		6	9.240446160521	0.10	
	acidm	9	0.000000000000	-11.44	2C.-.C 4C.-.H C.-.N 5C.-.O 6H.-.O 2N.-.O 3O.-.O 4H.-.H H.-.N 2Hp Hb(MtoAm) SB
		4	0.941547380038	-10.50	4C.-.H C.-.N 6H.-.O 2N.-.O 2C.-.O O.-.O 4H.-.H H.-.N Hb(MtoAm) SB
		7	6.284494583244	-5.16	4C.-.O 4C.-.H 2O.-.O 4H.-.O 2H.-.H Hb(AmtoM)
		3	9.248052126246	-2.19	10C.-.H 2C.-.C 3C.-.N 10H.-.H 3H.-.N 5H.-.O 2C.-.O 2Hp SB
		2	9.321655542585	-2.12	9C.-.H 9H.-.H 3C.-.C 3C.-.N H.-.N 5H.-.O 2C.-.O N.-.O 3Hp SB
		6	9.513849835536	-1.93	3C.-.C 8C.-.O 5C.-.H 7H.-.O 4H.-.H 2O.-.O 3Hp
		0	11.111113418475	-0.33	C.-.O C.-.H 2H.-.O 2H.-.H
		8	11.236103734874	-0.21	H.-.H SB

Continue in the next page

Table SM1: Structures of various conformations are evaluated for their energetic properties and types of intermolecular interactions. In this context, Am stands for amino acid, FM for functional monomer, N° conf. for the spatial conformation number of the Amino acid-FM complex, E_{tot} represents the ground state electronic energy in kcal mol^{-1}, the ΔE represents the difference of the electronic energy in ascending order of energy between the complex and lastly, the type of interaction specifies the atoms that are in close proximity in the table. The symbols Hb denote a hydrogen bond, AmtoM indicates that an AM is complexing with an FM, and MtoAm is the reverse of AmtoM. The symbols SB denote a salt bridges. The symbols Hp denote a hydrophobics interactions. The symbols Cation-pi/pi-staquing/pi-T-sheped denote the type of π interactions interactions.

AA	FM	N° conf.	ΔE	E_{tot}	Type of interaction
		5	11.313589260989	-0.13	
	acida	9	0.000000000000	-10.50	4C.-.H C.-.C 4C.-.O 4O.-.O 4H.-.O H.-.H Hp Hb(MtoAm) Hb(AmtoM)
		5	0.303734476795	-10.19	4C.-.H C.-.C 4C.-.O 4O.-.O 4H.-.O H.-.H Hp Hb(AmtoM) Hb(MtoAm) SB
		8	5.210130723660	-5.29	C.-.N 3C.-.O 3C.-.H 2N.-.O 2O.-.O 4H.-.O 4H.-.H H.-.N Hb(MtoAm) SB
		4	6.731740098773	-3.77	2C.-.H 2H.-.H 2C.-.O 2H.-.O 2O.-.O Hb(AmtoM)
		3	7.565992385016	-2.93	9C.-.H 5C.-.C 3C.-.O 8H.-.O 5H.-.H H.-.N C.-.N N.-.O 5Hp Hb(AmtoM) SB
		2	8.880087086323	-1.62	5C.-.C 2C.-.N 9C.-.H 2H.-.N 7H.-.H 6C.-.O 4H.-.O 3O.-.O 5Hp SB

Continue in the next page

Table SM1: Structures of various conformations are evaluated for their energetic properties and types of intermolecular interactions. In this context, Am stands for amino acid, FM for functional monomer, N° conf. for the spatial conformation number of the Amino acid-FM complex, E_{tot} represents the ground state electronic energy in kcal mol^{-1}, the ΔE represents the difference of the electronic energy in ascending order of energy between the complex and lastly, the type of interaction specifies the atoms that are in close proximity in the table. The symbols Hb denote a hydrogen bond, AmtoM indicates that an AM is complexing with an FM, and MtoAm is the reverse of AmtoM. The symbols SB denote a salt bridges. The symbols Hp denote a hydrophobics interactions. The symbols Cation-pi/pi-staquing/pi-T-sheped denote the type of π interactions interactions.

AA	FM	N° conf.	ΔE	E_{tot}	Type of interaction
		7	9.046548756391	-1.45	6C.-.H 2H.-.H 2C.-.N C.-.C 2H.-.N 3C.-.O 5H.-.O 2N.-.O Hp SB
		0	10.501251015299	0.00	
	bisac	8	0.000000000000	-6.59	3C.-.O 4C.-.H 2H.-.O H.-.H H.-.N 2O.-.O Hb(AmtoM)
		0	0.032676464416	-6.56	8C.-.H 2N.-.O 3H.-.O 4C.-.N 7H.-.H 6H.-.N N.-.N C.-.O Hb(MtoAm)
		4	2.631289810674	-3.96	7C.-.H 9H.-.H 3H.-.N 8H.-.O 5C.-.O 2N.-.O 2O.-.O C.-.N Hb(MtoAm)
		2	3.253085244647	-3.34	8H.-.O 7H.-.H 3H.-.N C.-.N 2N.-.O 7C.-.H 3C.-.O 2O.-.O Hb(MtoAm)
		7	3.893156134297	-2.70	11C.-.H 4H.-.N 5H.-.O 3C.-.O 2C.-.N N.-.O 6H.-.H 2C.-.C 2Hp Hb(MtoAm)

Continue in the next page

Table SM1: Structures of various conformations are evaluated for their energetic properties and types of intermolecular interactions. In this context, Am stands for amino acid, FM for functional monomer, N° conf. for the spatial conformation number of the Amino acid-FM complex, E_{tot} represents the ground state electronic energy in kcal mol^{-1}, the ΔE represents the difference of the electronic energy in ascending order of energy between the complex and lastly, the type of interaction specifies the atoms that are in close proximity in the table. The symbols Hb denote a hydrogen bond, AmtoM indicates that an AM is complexing with an FM, and MtoAm is the reverse of AmtoM. The symbols SB denote a salt bridges. The symbols Hp denote a hydrophobics interactions. The symbols Cation-pi/pi-staquing/pi-T-sheped denote the type of π interactions interactions.

AA	FM	N° conf.	ΔE	E_{tot}	Type of interaction
		5	3.974033486946	-2.62	7C.-.H 4H.-.N 5H.-.O 3C.-.O C.-.N N.-.O 6H.-.H Hb(MtoAm)
		6	4.128042543703	-2.46	C.-.C 4C.-.O 8C.-.H 8H.-.H 8H.-.O 2C.-.N 3H.-.N 2N.-.O Hp Hb(MtoAm)
		3	5.680944009365	-0.91	5H.-.O C.-.N 3N.-.O H.-.N 3C.-.O 5C.-.H 5H.-.H O.-.O Hb(MtoAm)
		9	6.562872273416	-0.03	
	1ally	1	0.000000000000	-3.91	4C.-.C 18C.-.H 2C.-.O 3H.-.N 17H.-.H 5H.-.O 2C.-.N N.-.O 4Hp 2SB
		8	0.620826526242	-3.29	7H.-.N 3C.-.N N.-.N 15H.-.H 8C.-.H C.-.C Hp Hb(AmtoM)
		3	1.639112007072	-2.27	14C.-.H 2C.-.C 2C.-.O 14H.-.H 3H.-.N 5H.-.O C.-.N 2Hp SB
		9	2.285404274973	-1.62	3C.-.C 10C.-.H 2C.-.O 10H.-.H 4H.-.O C.-.N 3H.-.N 3Hp 2SB

Continue in the next page

Table SM1: Structures of various conformations are evaluated for their energetic properties and types of intermolecular interactions. In this context, Am stands for amino acid, FM for functional monomer, Nº conf. for the spatial conformation number of the Amino acid-FM complex, E_{tot} represents the ground state electronic energy in kcal mol^{-1}, the ΔE represents the difference of the electronic energy in ascending order of energy between the complex and lastly, the type of interaction specifies the atoms that are in close proximity in the table. The symbols Hb denote a hydrogen bond, AmtoM indicates that an AM is complexing with an FM, and MtoAm is the reverse of AmtoM. The symbols SB denote a salt bridges. The symbols Hp denote a hydrophobics interactions. The symbols Cation-pi/pi-staquing/pi-T-sheped denote the type of π interactions interactions.

AA	FM	Nº conf.	ΔE	E_{tot}	Type of interaction
		7	2.602592448538	-1.30	2C.-.C 3C.-.O 7C.-.H 6H.-.O 8H.-.H C.-.N 3H.-.N 2Hp SB
		2	3.454995938592	-0.45	
		4	3.537153136904	-0.37	C.-.N 4C.-.H 4H.-.N 8H.-.H
		6	3.824005361266	-0.08	
		5	3.832686355467	-0.07	
	4imid	9	0.000000000000	-3.36	7C.-.O 3O.-.O 4H.-.O 16C.-.H 6C.-.C C.-.N 8H.-.H 3H.-.N 6Hp SB
		1	0.862451865904	-2.50	4H.-.O 4C.-.N 13C.-.H N.-.O 2C.-.C 3H.-.H 2H.-.N 2Hp Hb(AmtoM) SB
		5	1.723269178831	-1.64	2H.-.N N.-.O C.-.O 4C.-.H 5H.-.H 2H.-.O Hb(MtoAm)
		8	2.176544975882	-1.19	4C.-.H 3H.-.H C.-.C 2C.-.N 4H.-.N Hp
		6	3.121558713944	-0.24	SB
		7	3.401670309526	0.04	

Continue in the next page

Table SM1: Structures of various conformations are evaluated for their energetic properties and types of intermolecular interactions. In this context, Am stands for amino acid, FM for functional monomer, N° conf. for the spatial conformation number of the Amino acid-FM complex, E_{tot} represents the ground state electronic energy in kcal mol^{-1}, the ΔE represents the difference of the electronic energy in ascending order of energy between the complex and lastly, the type of interaction specifies the atoms that are in close proximity in the table. The symbols Hb denote a hydrogen bond, AmtoM indicates that an AM is complexing with an FM, and MtoAm is the reverse of AmtoM. The symbols SB denote a salt bridges. The symbols Hp denote a hydrophobics interactions. The symbols Cation-pi/pi-staquing/pi-T-sheped denote the type of π interactions interactions.

AA	FM	N° conf.	ΔE	E_{tot}	Type of interaction
		2	3.657963311656	0.29	
	acril	1	0.000000000000	-6.92	4C.-.O 4C.-.H 3H.-.O H.-.H 2O.-.O H.-.N Hb(AmtoM)
		8	4.078618876637	-2.84	6C.-.H 4C.-.O 4H.-.N 4H.-.H 2C.-.C 2C.-.N 7H.-.O 3N.-.O O.-.O 2Hp Hb(AmtoM)
		7	4.228001834019	-2.70	2C.-.O 2C.-.H 2H.-.N 4H.-.O 3H.-.H C.-.N N.-.O Hb(MtoAm)
		4	4.433929384814	-2.49	9C.-.H 6C.-.C C.-.N 6C.-.O 7H.-.H 2H.-.N 5H.-.O N.-.O 6Hp
		3	5.403275907183	-1.52	C.-.O 3H.-.O 5H.-.H C.-.N 2H.-.N N.-.O 2C.-.H Hb(MtoAm)
		9	5.545202312607	-1.38	3C.-.C 6C.-.H 2H.-.N 4H.-.H 3H.-.O C.-.O N.-.O 3Hp
		6	6.157521670948	-0.77	2C.-.C 6C.-.H C.-.O 7H.-.H 2H.-.O 2Hp

Continue in the next page

Table SM1: Structures of various conformations are evaluated for their energetic properties and types of intermolecular interactions. In this context, Am stands for amino acid, FM for functional monomer, N° conf. for the spatial conformation number of the Amino acid-FM complex, E_{tot} represents the ground state electronic energy in kcal mol^{-1}, the ΔE represents the difference of the electronic energy in ascending order of energy between the complex and lastly, the type of interaction specifies the atoms that are in close proximity in the table. The symbols Hb denote a hydrogen bond, AmtoM indicates that an AM is complexing with an FM, and MtoAm is the reverse of AmtoM. The symbols SB denote a salt bridges. The symbols Hp denote a hydrophobics interactions. The symbols Cation-pi/pi-staquing/pi-T-sheped denote the type of π interactions interactions.

AA	FM	N° conf.	ΔE	E_{tot}	Type of interaction
		0	6.512287920258	-0.41	C.-.C 5C.-.H 2C.-.O 3H.-.O 3H.-.H Hp
		2	6.704208191660	-0.22	C.-.H H.-.H
	alila	2	0.000000000000	-3.35	C.-.C 8C.-.H 3C.-.N 8H.-.H 7H.-.N 3H.-.O N.-.N N.-.O Hp Hb(MtoAm) SB
		0	0.000097402196	-3.35	8C.-.H 2C.-.C C.-.O 2C.-.N 11H.-.H 7H.-.N 2H.-.O N.-.N 2Hp Hb(AmtoM) SB
		7	0.066623663885	-3.28	3C.-.N 7C.-.H N.-.N 7H.-.N N.-.O 9H.-.H 2H.-.O Hb(MtoAm) SB
		9	1.378080941413	-1.97	3H.-.N 2C.-.C 3C.-.N 9C.-.H 2C.-.O 11H.-.H 6H.-.O N.-.O 2Hp SB
		5	1.659536295471	-1.69	4C.-.O 6C.-.H 8H.-.O 6H.-.H C.-.C Hp SB

Continue in the next page

Table SM1: Structures of various conformations are evaluated for their energetic properties and types of intermolecular interactions. In this context, Am stands for amino acid, FM for functional monomer, N° conf. for the spatial conformation number of the Amino acid-FM complex, E_{tot} represents the ground state electronic energy in kcal mol^{-1}, the ΔE represents the difference of the electronic energy in ascending order of energy between the complex and lastly, the type of interaction specifies the atoms that are in close proximity in the table. The symbols Hb denote a hydrogen bond, AmtoM indicates that an AM is complexing with an FM, and MtoAm is the reverse of AmtoM. The symbols SB denote a salt bridges. The symbols Hp denote a hydrophobics interactions. The symbols Cation-pi/pi-staquing/pi-T-sheped denote the type of π interactions interactions.

AA	FM	N° conf.	ΔE	E_{tot}	Type of interaction
		1	1.713449921945	-1.64	4C.-.C 10C.-.H 2C.-.O 8H.-.H 5H.-.O C.-.N 2H.-.N 4Hp SB
		6	1.970309426007	-1.38	2C.-.C 7C.-.H 2C.-.N 9H.-.H 3H.-.N N.-.O H.-.O 2Hp SB
		3	2.893149808084	-0.46	C.-.O 3H.-.O C.-.H H.-.H SB
		8	3.028016024118	-0.32	3H.-.N C.-.N 6H.-.H 3C.-.H H.-.O SB
	estir	6	0.000000000000	-3.34	6C.-.N 21C.-.H 11H.-.H 3C.-.C 3Hp Cation-pi(Amc)
		4	2.783287932531	-0.55	2H.-.H C.-.H
		0	2.857888108776	-0.48	2C.-.O C.-.H 3H.-.O 2H.-.H
		8	2.977162903923	-0.36	6C.-.O 6C.-.H 2H.-.O 3H.-.H
		3	3.268738636694	-0.07	
		1	3.328103071598	-0.01	
		9	3.330598295226	-0.01	
		5	3.371434035479	0.03	
		2	8.014562539747	4.68	7C.-.H 2H.-.N 7H.-.H C.-.C C.-.N Hp

Continue in the next page

Table SM1: Structures of various conformations are evaluated for their energetic properties and types of intermolecular interactions. In this context, Am stands for amino acid, FM for functional monomer, Nº conf. for the spatial conformation number of the Amino acid-FM complex, E_{tot} represents the ground state electronic energy in kcal mol^{-1}, the ΔE represents the difference of the electronic energy in ascending order of energy between the complex and lastly, the type of interaction specifies the atoms that are in close proximity in the table. The symbols Hb denote a hydrogen bond, AmtoM indicates that an AM is complexing with an FM, and MtoAm is the reverse of AmtoM. The symbols SB denote a salt bridges. The symbols Hp denote a hydrophobics interactions. The symbols Cation-pi/pi-staquing/pi-T-sheped denote the type of π interactions interactions.

AA	FM	Nº conf.	ΔE	E_{tot}	Type of interaction
	1viny	7	0.000000000000	-3.93	12C.-.H 7C.-.C 5C.-.N 7H.-.N 2N.-.O 5C.-.O 10H.-.H 4H.-.O 7Hp Cation-pi(Amc)
		3	0.227889742404	-3.70	4C.-.C 4C.-.N 10C.-.H 2N.-.N 6H.-.N N.-.O 4C.-.O 9H.-.H 4H.-.O 4Hp Hb(AmtoM) Cation-pi(Amc)
		9	0.272443344734	-3.66	13C.-.H 7C.-.C 4C.-.O 10H.-.H 5H.-.N 5C.-.N N.-.N N.-.O 3H.-.O 7Hp
		5	0.776521467055	-3.15	9C.-.H 4C.-.C 5C.-.N 5C.-.O 6H.-.N N.-.O 6H.-.H 3H.-.O 4Hp Cation-pi(Amc)
		0	1.190228993446	-2.74	11C.-.H 3C.-.N 7H.-.N 2N.-.N 3C.-.C 7H.-.H 3Hp Cation-pi(Amc)
		1	1.333313187351	-2.60	10C.-.H 6H.-.N 3C.-.N 9H.-.H 3C.-.C C.-.O 3H.-.O 3Hp

Continue in the next page

Table SM1: Structures of various conformations are evaluated for their energetic properties and types of intermolecular interactions. In this context, Am stands for amino acid, FM for functional monomer, N° conf. for the spatial conformation number of the Amino acid-FM complex, E_{tot} represents the ground state electronic energy in $\mathrm{kcal\,mol^{-1}}$, the ΔE represents the difference of the electronic energy in ascending order of energy between the complex and lastly, the type of interaction specifies the atoms that are in close proximity in the table. The symbols Hb denote a hydrogen bond, AmtoM indicates that an AM is complexing with an FM, and MtoAm is the reverse of AmtoM. The symbols SB denote a salt bridges. The symbols Hp denote a hydrophobics interactions. The symbols Cation-pi/pi-staquing/pi-T-sheped denote the type of π interactions interactions.

AA	FM	N° conf.	ΔE	E_{tot}	Type of interaction
		2	1.515246811040	-2.42	5C.-.H 2C.-.N 4H.-.N N.-.N 4H.-.H Hb(AmtoM)
		8	1.896602151913	-2.03	5C.-.H 8H.-.H 4H.-.O 2C.-.C C.-.N C.-.O 2H.-.N 2Hp
		4	3.940231387128	0.01	
	2hydr	3	0.000000000000	-1.64	10C.-.H 7H.-.O 2C.-.O 2C.-.C 2C.-.N 9H.-.H 3H.-.N N.-.O 2Hp Hb(AmtoM)
		5	0.856792664107	-0.78	7C.-.H 12H.-.H C.-.C C.-.N 3H.-.N 3H.-.O C.-.O N.-.O Hp Hb(AmtoM)
		4	1.746302734416	0.11	
		9	1.840640791323	0.20	
		1	2.152770590208	0.52	
	4viny	0	0.000000000000	-9.80	3C.-.O 5C.-.H 3H.-.O 2H.-.H C.-.N 2N.-.O H.-.N Hb(AmtoM)

Continue in the next page

Table SM1: Structures of various conformations are evaluated for their energetic properties and types of intermolecular interactions. In this context, Am stands for amino acid, FM for functional monomer, N° conf. for the spatial conformation number of the Amino acid-FM complex, E_{tot} represents the ground state electronic energy in $kcal\,mol^{-1}$, the ΔE represents the difference of the electronic energy in ascending order of energy between the complex and lastly, the type of interaction specifies the atoms that are in close proximity in the table. The symbols Hb denote a hydrogen bond, AmtoM indicates that an AM is complexing with an FM, and MtoAm is the reverse of AmtoM. The symbols SB denote a salt bridges. The symbols Hp denote a hydrophobics interactions. The symbols Cation-pi/pi-staquing/pi-T-sheped denote the type of π interactions interactions.

AA	FM	N° conf.	ΔE	E_{tot}	Type of interaction
		2	5.594018203261	-4.21	18C.-.H 7C.-.C 5C.-.O 2H.-.N 5C.-.N 12H.-.H 4H.-.O 7Hp Cation-pi(Amc)
		5	5.712364420345	-4.09	8C.-.C 4C.-.N 15C.-.H 6C.-.O N.-.N 5H.-.N 4H.-.O 7H.-.H 8Hp Hb(AmtoM) Cation-pi(Amc)
		1	8.046405872293	-1.75	4C.-.C 6C.-.H C.-.N C.-.O 4H.-.H H.-.N 2H.-.O 4Hp
		8	8.349161606413	-1.45	6C.-.H 2C.-.O 6H.-.H 2H.-.O
		4	9.706440101949	-0.09	
		6	9.800526987999	-0.00	
	acrol	1	0.000000000000	-4.86	3C.-.H C.-.C 3C.-.O 2O.-.O 3H.-.O H.-.H Hp Hb(AmtoM)
		7	2.347978650578	-2.51	11C.-.H 6C.-.C C.-.N 5C.-.O 11H.-.H 2H.-.N 4H.-.O O.-.O 6Hp

Continue in the next page

Table SM1: Structures of various conformations are evaluated for their energetic properties and types of intermolecular interactions. In this context, Am stands for amino acid, FM for functional monomer, N° conf. for the spatial conformation number of the Amino acid-FM complex, E_{tot} represents the ground state electronic energy in kcal mol^{-1}, the ΔE represents the difference of the electronic energy in ascending order of energy between the complex and lastly, the type of interaction specifies the atoms that are in close proximity in the table. The symbols Hb denote a hydrogen bond, AmtoM indicates that an AM is complexing with an FM, and MtoAm is the reverse of AmtoM. The symbols SB denote a salt bridges. The symbols Hp denote a hydrophobics interactions. The symbols Cation-pi/pi-staquing/pi-T-sheped denote the type of π interactions interactions.

AA	FM	N° conf.	ΔE	E_{tot}	Type of interaction
		9	2.469568836679	-2.39	8C.-.H C.-.C 2C.-.N 5H.-.H H.-.N 4H.-.O C.-.O N.-.O Hp Hb(AmtoM)
		8	2.530918075944	-2.33	8C.-.H 6C.-.C 2C.-.N 4C.-.O 9H.-.H 3H.-.N 3H.-.O 2O.-.O 6Hp
		4	3.369206896544	-1.49	5C.-.H 2C.-.C C.-.N 3H.-.H H.-.N 3H.-.O 2C.-.O N.-.O 2O.-.O 2Hp
		2	3.720623975832	-1.14	C.-.C C.-.N 4C.-.H 5H.-.H H.-.N H.-.O Hp
		5	3.796860211054	-1.06	2C.-.C 2C.-.N 6C.-.H 2H.-.N 7H.-.H H.-.O 2Hp
		3	4.008682975460	-0.85	7C.-.H 2C.-.C 2C.-.O 6H.-.H 3H.-.O 2Hp
		0	4.534066333206	-0.32	C.-.C 5C.-.H 3H.-.H H.-.O Hp
		6	4.862071022216	0.00	

Continue in the next page

Table SM1: Structures of various conformations are evaluated for their energetic properties and types of intermolecular interactions. In this context, Am stands for amino acid, FM for functional monomer, N° conf. for the spatial conformation number of the Amino acid-FM complex, E_{tot} represents the ground state electronic energy in kcal mol^{-1}, the ΔE represents the difference of the electronic energy in ascending order of energy between the complex and lastly, the type of interaction specifies the atoms that are in close proximity in the table. The symbols Hb denote a hydrogen bond, AmtoM indicates that an AM is complexing with an FM, and MtoAm is the reverse of AmtoM. The symbols SB denote a salt bridges. The symbols Hp denote a hydrophobics interactions. The symbols Cation-pi/pi-staquing/pi-T-sheped denote the type of π interactions interactions.

AA	FM	N° conf.	ΔE	E_{tot}	Type of interaction
	itaco	8	0.000000000000	-13.62	11H.-.O 9C.-.O 3O.-.O 11H.-.H 7C.-.C 15C.-.H 3C.-.N 2H.-.N N.-.O 7Hp Hb(MtoAm) 2SB
		5	4.001778171096	-9.62	8H.-.O 3C.-.O 2O.-.O 5H.-.H 3C.-.H 2H.-.N C.-.N N.-.O Hb(MtoAm) Hb(AmtoM) 2SB
		0	10.453846063635	-3.17	5C.-.O 6H.-.O 3O.-.O 3H.-.H 5C.-.H 2C.-.C N.-.O 2Hp Hb(MtoAm) SB
		3	11.279882317259	-2.34	3C.-.O 6H.-.O 3N.-.O 3O.-.O 11C.-.H 8H.-.H 4C.-.C 4C.-.N 2H.-.N 4Hp 2SB
		7	12.056597313122	-1.57	2N.-.O 7H.-.O 2H.-.N 4H.-.H C.-.C 2C.-.N 7C.-.H C.-.O Hp SB
		6	12.177072395460	-1.45	8C.-.H 8H.-.H H.-.N C.-.C C.-.N 7H.-.O 2N.-.O C.-.O Hp Hb(AmtoM) SB
		4	13.579511179625	-0.04	

Continue in the next page

Table SM1: Structures of various conformations are evaluated for their energetic properties and types of intermolecular interactions. In this context, Am stands for amino acid, FM for functional monomer, N° conf. for the spatial conformation number of the Amino acid-FM complex, E_{tot} represents the ground state electronic energy in kcal mol^{-1}, the ΔE represents the difference of the electronic energy in ascending order of energy between the complex and lastly, the type of interaction specifies the atoms that are in close proximity in the table. The symbols Hb denote a hydrogen bond, AmtoM indicates that an AM is complexing with an FM, and MtoAm is the reverse of AmtoM. The symbols SB denote a salt bridges. The symbols Hp denote a hydrophobics interactions. The symbols Cation-pi/pi-staquing/pi-T-sheped denote the type of π interactions interactions.

AA	FM	N° conf.	ΔE	E_{tot}	Type of interaction
		2	14.162644346096	0.54	O.-.O 2H.-.O 2H.-.H
HIS	14dvb	7	0.000000000000	-1.21	12C.-.H 2C.-.O 6H.-.H 2H.-.O 2H.-.N C.-.N
		8	0.184277864158	-1.02	4C.-.O 2C.-.H 4H.-.O 2H.-.H
		4	1.246131654496	0.04	
		1	1.260861066469	0.05	
		6	1.298424763128	0.09	
	2viny	6	0.000000000000	-5.72	8C.-.N 6C.-.C 19C.-.H N.-.N 8H.-.N 13H.-.H H.-.O 6Hp Hb(AmtoM)
		0	3.131175441561	-2.59	6C.-.C 12C.-.H C.-.N 2H.-.N 8H.-.H 6Hp pi-T-sheped
		4	4.413604039797	-1.30	2C.-.C 4C.-.N 10C.-.H 5H.-.N 9H.-.H 2Hp
		1	4.760101060216	-0.96	8C.-.H 2C.-.C C.-.O 4H.-.H 2H.-.O 2Hp
		3	5.734672758485	0.02	C.-.C 5C.-.H H.-.N 4H.-.H Hp
		8	5.793985599700	0.08	

Continue in the next page

Table SM1: Structures of various conformations are evaluated for their energetic properties and types of intermolecular interactions. In this context, Am stands for amino acid, FM for functional monomer, N° conf. for the spatial conformation number of the Amino acid-FM complex, E_{tot} represents the ground state electronic energy in kcal mol^{-1}, the ΔE represents the difference of the electronic energy in ascending order of energy between the complex and lastly, the type of interaction specifies the atoms that are in close proximity in the table. The symbols Hb denote a hydrogen bond, AmtoM indicates that an AM is complexing with an FM, and MtoAm is the reverse of AmtoM. The symbols SB denote a salt bridges. The symbols Hp denote a hydrophobics interactions. The symbols Cation-pi/pi-staquing/pi-T-sheped denote the type of π interactions interactions.

AA	FM	N° conf.	ΔE	E_{tot}	Type of interaction
	acidm	8	0.000000000000	-2.88	4C.-.C 12C.-.H 2H.-.N 10H.-.H C.-.N 2C.-.O 2N.-.O 5H.-.O 4Hp Hb(AmtoM) SB
		9	1.145432712594	-1.74	9C.-.H C.-.N C.-.C 3C.-.O 2H.-.N 4H.-.H N.-.O 2H.-.O O.-.O Hp SB
		5	2.405994935217	-0.48	6C.-.O C.-.C 4H.-.O 4O.-.O C.-.H H.-.H Hp SB
		1	3.055477911866	0.17	
		2	3.146865974352	0.26	
		4	27.763764808898	24.88	
	acida	1	0.000000000000	-11.68	8C.-.H 2C.-.N C.-.C 4C.-.O 3N.-.O 6H.-.O 3H.-.N 4H.-.H Hp Hb(MtoAm) Hb(AmtoM) SB
		9	8.386263873156	-3.29	5C.-.H 3H.-.H H.-.N C.-.N 2C.-.O 3H.-.O N.-.O Hb(AmtoM)

Continue in the next page

Table SM1: Structures of various conformations are evaluated for their energetic properties and types of intermolecular interactions. In this context, Am stands for amino acid, FM for functional monomer, N° conf. for the spatial conformation number of the Amino acid-FM complex, E_{tot} represents the ground state electronic energy in kcal mol^{-1}, the ΔE represents the difference of the electronic energy in ascending order of energy between the complex and lastly, the type of interaction specifies the atoms that are in close proximity in the table. The symbols Hb denote a hydrogen bond, AmtoM indicates that an AM is complexing with an FM, and MtoAm is the reverse of AmtoM. The symbols SB denote a salt bridges. The symbols Hp denote a hydrophobics interactions. The symbols Cation-pi/pi-staquing/pi-T-sheped denote the type of π interactions interactions.

AA	FM	N° conf.	ΔE	E_{tot}	Type of interaction
		7	9.332981875899	-2.35	4C.-.C 10C.-.H 4C.-.N 7H.-.H 3H.-.N 6H.-.O 4C.-.O 4N.-.O 4Hp Hb(AmtoM) SB
		0	9.391347219032	-2.29	5C.-.C 4C.-.N 11C.-.H 3H.-.N 4H.-.H 2C.-.O N.-.O 3H.-.O 5Hp
		4	9.516225083843	-2.16	10C.-.H 3C.-.N 7H.-.H 3C.-.C 3H.-.N N.-.O 2C.-.O 2H.-.O 3Hp SB
		5	10.208731332421	-1.47	4C.-.H 2C.-.N C.-.O 6H.-.O 7H.-.H 2H.-.N N.-.O Hb(AmtoM) SB
		6	11.806264667842	0.13	
		8	11.868569529678	0.19	
	bisac	1	0.000000000000	-6.84	14C.-.H 3C.-.O 3H.-.O N.-.O 7H.-.N 2C.-.C 4C.-.N 6H.-.H N.-.N 2Hp Hb(MtoAm)
		4	3.984490454280	-2.86	14C.-.H 8H.-.H 8H.-.N 3C.-.N C.-.C C.-.O 3H.-.O Hp

Continue in the next page

Table SM1: Structures of various conformations are evaluated for their energetic properties and types of intermolecular interactions. In this context, Am stands for amino acid, FM for functional monomer, Nº conf. for the spatial conformation number of the Amino acid-FM complex, E_{tot} represents the ground state electronic energy in kcal mol^{-1}, the ΔE represents the difference of the electronic energy in ascending order of energy between the complex and lastly, the type of interaction specifies the atoms that are in close proximity in the table. The symbols Hb denote a hydrogen bond, AmtoM indicates that an AM is complexing with an FM, and MtoAm is the reverse of AmtoM. The symbols SB denote a salt bridges. The symbols Hp denote a hydrophobics interactions. The symbols Cation-pi/pi-staquing/pi-T-sheped denote the type of π interactions interactions.

AA	FM	Nº conf.	ΔE	E_{tot}	Type of interaction
		3	4.796809194901	-2.04	4C.-.C 16C.-.H 3C.-.N 11H.-.H 2C.-.O 2H.-.N 3H.-.O N.-.O 4Hp
	1ally	3	0.000000000000	-8.21	10H.-.N 9C.-.N 17C.-.H 17H.-.H 2C.-.C N.-.N 2Hp Hb(AmtoM)
		9	5.645228355619	-2.56	C.-.N 5H.-.N 14C.-.H 12H.-.H 2C.-.C 2Hp
		5	6.075356482088	-2.13	C.-.C 15C.-.H 6H.-.N 14H.-.H 3C.-.N H.-.O Hp SB
		0	6.531060164167	-1.68	3C.-.C 8C.-.H 2H.-.N 5H.-.H 3Hp
		1	6.586124477687	-1.62	9C.-.H 8H.-.H 2C.-.C 2C.-.O H.-.N 4H.-.O 2Hp SB
		8	8.022656511979	-0.18	
		6	8.159480776617	-0.05	
		7	8.169078331861	-0.04	
	4imid	0	0.000000000000	-5.55	4C.-.H 4H.-.H 6H.-.N 4C.-.N N.-.N Hb(AmtoM)

Continue in the next page

Table SM1: Structures of various conformations are evaluated for their energetic properties and types of intermolecular interactions. In this context, Am stands for amino acid, FM for functional monomer, N° conf. for the spatial conformation number of the Amino acid-FM complex, E_{tot} represents the ground state electronic energy in kcal mol^{-1}, the ΔE represents the difference of the electronic energy in ascending order of energy between the complex and lastly, the type of interaction specifies the atoms that are in close proximity in the table. The symbols Hb denote a hydrogen bond, AmtoM indicates that an AM is complexing with an FM, and MtoAm is the reverse of AmtoM. The symbols SB denote a salt bridges. The symbols Hp denote a hydrophobics interactions. The symbols Cation-pi/pi-staquing/pi-T-sheped denote the type of π interactions interactions.

AA	FM	N° conf.	ΔE	E_{tot}	Type of interaction
		5	0.837063530056	-4.71	4C.-.O 6H.-.O 2O.-.O 4C.-.H 3H.-.H C.-.C Hp Hb(MtoAm)
		6	2.456738104103	-3.09	3H.-.O C.-.N C.-.H H.-.N H.-.H 2C.-.O N.-.O Hb(AmtoM)
		3	3.907693069967	-1.64	N.-.N 4H.-.N 2C.-.N 2C.-.C 5C.-.H 5H.-.H 2Hp
		1	4.691284830731	-0.86	2C.-.O 2H.-.O C.-.C 2C.-.H H.-.N H.-.H Hp
	acril	7	0.000000000000	-5.97	14C.-.H 2C.-.O 3C.-.N 2C.-.C 9H.-.H 2H.-.O 6H.-.N N.-.N 2Hp Hb(MtoAm)
		1	2.639811764785	-3.33	C.-.C 4C.-.N 9C.-.H 6H.-.N 7H.-.H N.-.N Hp Hb(MtoAm)
		2	2.690945900807	-3.28	8C.-.H 5H.-.H 2C.-.O 4H.-.O N.-.O Hb(MtoAm)
		4	4.239329231092	-1.73	C.-.C 2C.-.O 4C.-.H 4H.-.H 5H.-.O C.-.N 2N.-.O H.-.N Hp 2Hb(MtoAm)

Continue in the next page

Table SM1: Structures of various conformations are evaluated for their energetic properties and types of intermolecular interactions. In this context, Am stands for amino acid, FM for functional monomer, N° conf. for the spatial conformation number of the Amino acid-FM complex, E_{tot} represents the ground state electronic energy in kcal mol^{-1}, the ΔE represents the difference of the electronic energy in ascending order of energy between the complex and lastly, the type of interaction specifies the atoms that are in close proximity in the table. The symbols Hb denote a hydrogen bond, AmtoM indicates that an AM is complexing with an FM, and MtoAm is the reverse of AmtoM. The symbols SB denote a salt bridges. The symbols Hp denote a hydrophobics interactions. The symbols Cation-pi/pi-staquing/pi-T-sheped denote the type of π interactions interactions.

AA	FM	N° conf.	ΔE	E_{tot}	Type of interaction
		6	5.726027939632	-0.24	C.-.H C.-.O H.-.O
		5	6.030687205983	0.06	
		8	6.035278358017	0.07	
		9	6.164036381656	0.20	
	alila	1	0.000000000000	-4.12	3C.-.N 2C.-.O 8C.-.H 8H.-.N 3H.-.O 10H.-.H C.-.C N.-.N Hp Hb(AmtoM) SB
		4	0.937472108552	-3.18	14C.-.H 13H.-.H 3C.-.C 3C.-.N N.-.N 9H.-.N 3Hp
		0	2.325521268939	-1.79	2C.-.C 2C.-.N 10C.-.H 2C.-.O 3H.-.N 2N.-.O 7H.-.H 7H.-.O 2Hp Hb(MtoAm) SB
		2	2.894354834113	-1.22	2C.-.C 11C.-.H 10H.-.H 3H.-.N C.-.N 2Hp
		7	4.108505689158	-0.01	
		8	4.167156983822	0.05	
		3	4.221553236969	0.10	
		9	4.303548232619	0.19	H.-.H

Continue in the next page

Table SM1: Structures of various conformations are evaluated for their energetic properties and types of intermolecular interactions. In this context, Am stands for amino acid, FM for functional monomer, N° conf. for the spatial conformation number of the Amino acid-FM complex, E_{tot} represents the ground state electronic energy in kcal mol^{-1}, the ΔE represents the difference of the electronic energy in ascending order of energy between the complex and lastly, the type of interaction specifies the atoms that are in close proximity in the table. The symbols Hb denote a hydrogen bond, AmtoM indicates that an AM is complexing with an FM, and MtoAm is the reverse of AmtoM. The symbols SB denote a salt bridges. The symbols Hp denote a hydrophobics interactions. The symbols Cation-pi/pi-staquing/pi-T-sheped denote the type of π interactions interactions.

AA	FM	N° conf.	ΔE	E_{tot}	Type of interaction
	estir	5	0.000000000000	-2.41	2C.-.N 12C.-.H 5H.-.N 11H.-.H 3C.-.C 3Hp pi-T-sheped
		9	0.073594833761	-2.33	5C.-.O 14C.-.H 7H.-.H C.-.N 6C.-.C 2H.-.N 6Hp
		6	1.848268845908	-0.56	2C.-.N 6C.-.H 2H.-.N 4H.-.H C.-.C Hp
		2	2.081443450588	-0.33	2H.-.H
		1	2.511311920133	0.10	
		0	2.577441151404	0.17	
	1viny	7	0.000000000000	-4.65	4C.-.N 5C.-.H N.-.N 3H.-.N 3H.-.H Hb(AmtoM)
		2	1.372646237480	-3.28	11C.-.C 8C.-.N 15C.-.H 3N.-.N 7H.-.N 11H.-.H 11Hp pi-staquing
		8	1.379943211806	-3.27	4C.-.C 4C.-.N 13C.-.H 7H.-.N N.-.N 6H.-.H 4Hp pi-T-sheped Cation-pi(Amc)

Continue in the next page

Table SM1: Structures of various conformations are evaluated for their energetic properties and types of intermolecular interactions. In this context, Am stands for amino acid, FM for functional monomer, Nº conf. for the spatial conformation number of the Amino acid-FM complex, E_{tot} represents the ground state electronic energy in kcal mol^{-1}, the ΔE represents the difference of the electronic energy in ascending order of energy between the complex and lastly, the type of interaction specifies the atoms that are in close proximity in the table. The symbols Hb denote a hydrogen bond, AmtoM indicates that an AM is complexing with an FM, and MtoAm is the reverse of AmtoM. The symbols SB denote a salt bridges. The symbols Hp denote a hydrophobics interactions. The symbols Cation-pi/pi-staquing/pi-T-sheped denote the type of π interactions interactions.

AA	FM	Nº conf.	ΔE	E_{tot}	Type of interaction
		1	1.415242444632	-3.24	4C.-.C 14C.-.H 4C.-.N 11H.-.H 7H.-.N H.-.O 4Hp pi-T-sheped
		5	1.485909224965	-3.17	7C.-.H 6H.-.H H.-.O 5H.-.N 2C.-.N N.-.N C.-.C Hp Hb(AmtoM)
		9	4.274334365926	-0.38	4H.-.N 6C.-.H 2C.-.N 6H.-.H C.-.C Hp
		4	4.587289711551	-0.07	
		0	4.618436616016	-0.04	
		3	4.639707118418	-0.01	
		6	4.995780904523	0.34	
	2hydr	8	0.000000000000	-5.42	18C.-.H 6C.-.C 2C.-.N 5C.-.O 7H.-.O 4H.-.N 14H.-.H O.-.O 6Hp Hb(MtoAm)
		4	2.875945456372	-2.54	C.-.C C.-.N 6C.-.H 3H.-.N 6H.-.H C.-.O N.-.O 2H.-.O Hp Hb(AmtoM)

Continue in the next page

Table SM1: Structures of various conformations are evaluated for their energetic properties and types of intermolecular interactions. In this context, Am stands for amino acid, FM for functional monomer, N° conf. for the spatial conformation number of the Amino acid-FM complex, E_{tot} represents the ground state electronic energy in kcal mol^{-1}, the ΔE represents the difference of the electronic energy in ascending order of energy between the complex and lastly, the type of interaction specifies the atoms that are in close proximity in the table. The symbols Hb denote a hydrogen bond, AmtoM indicates that an AM is complexing with an FM, and MtoAm is the reverse of AmtoM. The symbols SB denote a salt bridges. The symbols Hp denote a hydrophobics interactions. The symbols Cation-pi/pi-staquing/pi-T-sheped denote the type of π interactions interactions.

AA	FM	N° conf.	ΔE	E_{tot}	Type of interaction
		3	3.628867704880	-1.79	5C.-.C 15C.-.H C.-.O 9H.-.H 2H.-.O 2C.-.N 3H.-.N 5Hp
		6	5.501266877765	0.08	
		9	5.693816534519	0.28	
	4viny	4	0.000000000000	-4.17	14C.-.C 4C.-.N 18C.-.H 13H.-.H 2H.-.N N.-.N 14Hp pi-staquing
		7	0.902888520766	-3.27	22C.-.H 7C.-.C 3H.-.N 12H.-.H 2H.-.O C.-.N 7Hp
		9	1.944694099567	-2.23	10C.-.H 8H.-.H C.-.C 3C.-.N 5H.-.N Hp
		8	2.584136031791	-1.59	10C.-.H C.-.C 2C.-.N 3H.-.N 7H.-.H Hp
		6	2.758084348894	-1.41	C.-.C 3C.-.H C.-.N 3H.-.N 3H.-.H Hp
		5	4.110990709534	-0.06	
		2	4.182688980304	0.01	
		3	4.425951830027	0.26	

Continue in the next page

Table SM1: Structures of various conformations are evaluated for their energetic properties and types of intermolecular interactions. In this context, Am stands for amino acid, FM for functional monomer, N° conf. for the spatial conformation number of the Amino acid-FM complex, E_{tot} represents the ground state electronic energy in kcal mol^{-1}, the ΔE represents the difference of the electronic energy in ascending order of energy between the complex and lastly, the type of interaction specifies the atoms that are in close proximity in the table. The symbols Hb denote a hydrogen bond, AmtoM indicates that an AM is complexing with an FM, and MtoAm is the reverse of AmtoM. The symbols SB denote a salt bridges. The symbols Hp denote a hydrophobics interactions. The symbols Cation-pi/pi-staquing/pi-T-sheped denote the type of π interactions interactions.

AA	FM	N° conf.	ΔE	E_{tot}	Type of interaction
	acrol	2	0.000000000000	-2.90	C.-.C C.-.N 9C.-.H 2H.-.N 4H.-.H 4C.-.O 3N.-.O 3H.-.O Hp Hb(AmtoM)
		3	0.676979858143	-2.22	2C.-.N 4C.-.H H.-.N 4H.-.O 4H.-.H C.-.O N.-.O Hb(AmtoM)
		8	1.281610647381	-1.61	4C.-.C 9C.-.H 4C.-.O 7H.-.H 4H.-.O O.-.O 4Hp
		6	1.707371679185	-1.19	C.-.N C.-.C 6C.-.H H.-.N 6H.-.H H.-.O Hp
		0	2.942858276988	0.05	
		7	2.961907676316	0.07	
	itaco	4	0.000000000000	-5.87	5C.-.C 16C.-.H 4C.-.N 7H.-.N 13H.-.H C.-.O 4H.-.O N.-.O 5Hp Hb(AmtoM) SB
		2	1.867298002622	-4.00	11H.-.O 3H.-.H 6C.-.H 4C.-.O 2N.-.O 2O.-.O C.-.N Hb(AmtoM) 2SB

Continue in the next page

Table SM1: Structures of various conformations are evaluated for their energetic properties and types of intermolecular interactions. In this context, Am stands for amino acid, FM for functional monomer, N° conf. for the spatial conformation number of the Amino acid-FM complex, E_{tot} represents the ground state electronic energy in kcal mol^{-1}, the ΔE represents the difference of the electronic energy in ascending order of energy between the complex and lastly, the type of interaction specifies the atoms that are in close proximity in the table. The symbols Hb denote a hydrogen bond, AmtoM indicates that an AM is complexing with an FM, and MtoAm is the reverse of AmtoM. The symbols SB denote a salt bridges. The symbols Hp denote a hydrophobics interactions. The symbols Cation-pi/pi-staquing/pi-T-sheped denote the type of π interactions interactions.

AA	FM	N° conf.	ΔE	E_{tot}	Type of interaction
		8	2.257136298651	-3.61	4C.-.O 2N.-.O 8H.-.O 3H.-.N 12H.-.H 2C.-.C 5C.-.N 20C.-.H O.-.O 2Hp 2SB
		9	4.052663538994	-1.81	
		1	5.822360010659	-0.04	
		7	6.235599620164	0.37	
ILE	14dvb	3	0.000000000000	-3.36	12C.-.H 8H.-.H 3C.-.O 2H.-.O
		6	2.122448205548	-1.23	
		2	2.125019380270	-1.23	
		5	2.179714096346	-1.18	
		8	2.299006466412	-1.06	
		1	2.922685134376	-0.43	
	2viny	6	0.000000000000	-4.61	11C.-.H 6H.-.N N.-.N C.-.C C.-.N 11H.-.H Hp Hb(AmtoM)
		5	0.377592292305	-4.23	3C.-.C 13C.-.H C.-.N 2H.-.N 11H.-.H H.-.O 3Hp
		9	1.926025497171	-2.68	3C.-.C 10C.-.H 8H.-.H 3Hp

Continue in the next page

Table SM1: Structures of various conformations are evaluated for their energetic properties and types of intermolecular interactions. In this context, Am stands for amino acid, FM for functional monomer, N° conf. for the spatial conformation number of the Amino acid-FM complex, E_{tot} represents the ground state electronic energy in $kcal\,mol^{-1}$, the ΔE represents the difference of the electronic energy in ascending order of energy between the complex and lastly, the type of interaction specifies the atoms that are in close proximity in the table. The symbols Hb denote a hydrogen bond, AmtoM indicates that an AM is complexing with an FM, and MtoAm is the reverse of AmtoM. The symbols SB denote a salt bridges. The symbols Hp denote a hydrophobics interactions. The symbols Cation-pi/pi-staquing/pi-T-sheped denote the type of π interactions interactions.

AA	FM	N° conf.	ΔE	E_{tot}	Type of interaction
		4	2.680017490121	-1.93	2C.-.C C.-.N 8C.-.H 2H.-.N 6H.-.H 3H.-.O 2Hp
		1	2.700424726185	-1.91	3C.-.C 7C.-.H 2H.-.N 5H.-.H C.-.N C.-.O 2H.-.O 3Hp
		3	3.374878480642	-1.23	
		8	4.486632651758	-0.12	8C.-.O 2N.-.O C.-.C H.-.O H.-.H Hp
	acidm	5	0.000000000000	-4.87	5C.-.H C.-.C 6C.-.O 4H.-.H 6H.-.O N.-.O 4O.-.O H.-.N Hp Hb(MtoAm) SB
		9	0.223210876534	-4.65	17C.-.H C.-.N 14H.-.H 2H.-.N 9H.-.O 3C.-.C 4C.-.O N.-.O 3Hp Hb(AmtoM) SB
		6	1.115636611665	-3.76	22C.-.H 14H.-.H 4H.-.N 8C.-.C 2C.-.N 4C.-.O 8H.-.O 8Hp SB
		4	1.354788326026	-3.52	22C.-.H 5C.-.C 2C.-.O 13H.-.H 6H.-.O 5Hp SB

Continue in the next page

Table SM1: Structures of various conformations are evaluated for their energetic properties and types of intermolecular interactions. In this context, Am stands for amino acid, FM for functional monomer, Nº conf. for the spatial conformation number of the Amino acid-FM complex, E_{tot} represents the ground state electronic energy in kcal mol^{-1}, the ΔE represents the difference of the electronic energy in ascending order of energy between the complex and lastly, the type of interaction specifies the atoms that are in close proximity in the table. The symbols Hb denote a hydrogen bond, AmtoM indicates that an AM is complexing with an FM, and MtoAm is the reverse of AmtoM. The symbols SB denote a salt bridges. The symbols Hp denote a hydrophobics interactions. The symbols Cation-pi/pi-staquing/pi-T-sheped denote the type of π interactions interactions.

AA	FM	Nº conf.	ΔE	E_{tot}	Type of interaction
		1	2.468930577577	-2.40	3C.-.C 11C.-.H 10H.-.H 4H.-.O C.-.O 3Hp
		0	3.304159727627	-1.57	3H.-.H
		3	3.689241389720	-1.18	
		7	4.284678048971	-0.59	
	acida	5	0.000000000000	-4.35	2C.-.O 2C.-.H 3O.-.O 4H.-.O H.-.H Hb(MtoAm) SB
		1	0.941784204505	-3.41	5C.-.O 2C.-.C 5C.-.H C.-.N 2N.-.O 3O.-.O 7H.-.O H.-.N 3H.-.H 2Hp SB
		9	1.152572109098	-3.20	9C.-.H C.-.C 12H.-.H 4H.-.O N.-.O H.-.N Hp Hb(AmtoM) SB
		8	2.671068725083	-1.68	C.-.C 4C.-.H 4H.-.H C.-.O 3H.-.O Hp
		6	2.873068261154	-1.48	C.-.O C.-.H 3H.-.O O.-.O
		7	3.016355934766	-1.34	
		3	3.091573883265	-1.26	
		2	3.886560186680	-0.47	

Continue in the next page

Table SM1: Structures of various conformations are evaluated for their energetic properties and types of intermolecular interactions. In this context, Am stands for amino acid, FM for functional monomer, N° conf. for the spatial conformation number of the Amino acid-FM complex, E_{tot} represents the ground state electronic energy in kcal mol^{-1}, the ΔE represents the difference of the electronic energy in ascending order of energy between the complex and lastly, the type of interaction specifies the atoms that are in close proximity in the table. The symbols Hb denote a hydrogen bond, AmtoM indicates that an AM is complexing with an FM, and MtoAm is the reverse of AmtoM. The symbols SB denote a salt bridges. The symbols Hp denote a hydrophobics interactions. The symbols Cation-pi/pi-staquing/pi-T-sheped denote the type of π interactions interactions.

AA	FM	N° conf.	ΔE	E_{tot}	Type of interaction
	bisac	2	0.000000000000	-6.00	25C.-.H 19H.-.H 2C.-.N 7H.-.N 6H.-.O C.-.O N.-.O N.-.N C.-.C Hp Hb(AmtoM)
		1	1.558588156879	-4.44	O.-.O 3C.-.O C.-.N 2N.-.O 5H.-.O 3C.-.H 3H.-.H Hb(MtoAm)
		3	1.872841438619	-4.13	4C.-.C 18C.-.H 16H.-.H C.-.O 4H.-.O 3H.-.N C.-.N 4Hp
		5	2.036097014076	-3.97	5H.-.H 4C.-.H 2H.-.N C.-.N 2C.-.O 4H.-.O N.-.O Hb(AmtoM)
		7	2.891542715076	-3.11	15C.-.H 12H.-.H 2C.-.C C.-.O 5H.-.O 3H.-.N C.-.N 2Hp
		0	4.891416984135	-1.11	
		6	5.315760924597	-0.69	
	1ally	6	0.000000000000	-3.87	4C.-.N 18C.-.H 5H.-.N 22H.-.H 3C.-.C 3Hp

Continue in the next page

Table SM1: Structures of various conformations are evaluated for their energetic properties and types of intermolecular interactions. In this context, Am stands for amino acid, FM for functional monomer, N° conf. for the spatial conformation number of the Amino acid-FM complex, E_{tot} represents the ground state electronic energy in kcal mol^{-1}, the ΔE represents the difference of the electronic energy in ascending order of energy between the complex and lastly, the type of interaction specifies the atoms that are in close proximity in the table. The symbols Hb denote a hydrogen bond, AmtoM indicates that an AM is complexing with an FM, and MtoAm is the reverse of AmtoM. The symbols SB denote a salt bridges. The symbols Hp denote a hydrophobics interactions. The symbols Cation-pi/pi-staquing/pi-T-sheped denote the type of π interactions interactions.

AA	FM	N° conf.	ΔE	E_{tot}	Type of interaction
		5	2.108551538334	-1.76	N.-.O 12C.-.H C.-.C 2C.-.O 11H.-.H 3H.-.O Hp SB
		7	2.667765798368	-1.21	
		2	3.211542829756	-0.66	
	4imid	2	0.000000000000	-5.99	7H.-.O 11H.-.H 19C.-.H 5C.-.C 5C.-.O 4C.-.N 5H.-.N 2N.-.N 5Hp Cation-pi(Amc)
		0	0.584360315156	-5.41	8H.-.O 8C.-.C 20C.-.H C.-.N 10H.-.H 3C.-.O N.-.O 2H.-.N 8Hp Hb(AmtoM) SB
		9	0.727834529532	-5.26	2C.-.O 2O.-.O 2H.-.H C.-.H 2H.-.O Hb(MtoAm)
		1	3.282745456894	-2.71	9C.-.H 9H.-.H 4C.-.C 2C.-.N 5H.-.N 4Hp
		3	3.364717075563	-2.63	8C.-.H C.-.C 2C.-.N 3H.-.N 8H.-.H Hp
		8	3.570509815324	-2.42	4H.-.O 12C.-.H C.-.C 12H.-.H C.-.N N.-.N 5H.-.N Hp

Continue in the next page

Table SM1: Structures of various conformations are evaluated for their energetic properties and types of intermolecular interactions. In this context, Am stands for amino acid, FM for functional monomer, Nº conf. for the spatial conformation number of the Amino acid-FM complex, E_{tot} represents the ground state electronic energy in kcal mol^{-1}, the ΔE represents the difference of the electronic energy in ascending order of energy between the complex and lastly, the type of interaction specifies the atoms that are in close proximity in the table. The symbols Hb denote a hydrogen bond, AmtoM indicates that an AM is complexing with an FM, and MtoAm is the reverse of AmtoM. The symbols SB denote a salt bridges. The symbols Hp denote a hydrophobics interactions. The symbols Cation-pi/pi-staquing/pi-T-sheped denote the type of π interactions interactions.

AA	FM	Nº conf.	ΔE	E_{tot}	Type of interaction
		6	4.730012933113	-1.26	
	acril	7	0.000000000000	-5.45	10C.-.H 2C.-.O 7H.-.H 3H.-.O 2C.-.N 3H.-.N N.-.O Hb(MtoAm)
		4	1.002678870877	-4.45	2C.-.O 3C.-.H 4H.-.H 4H.-.O H.-.N C.-.N N.-.O Hb(MtoAm)
		1	1.493834010124	-3.96	C.-.C 10C.-.H 9H.-.H C.-.N H.-.N C.-.O 3H.-.O N.-.O Hp Hb(AmtoM)
		6	2.537562265466	-2.92	16C.-.H 3C.-.N 7C.-.C 14H.-.H 4H.-.N 7H.-.O C.-.O 7Hp
		3	3.001309424391	-2.45	3C.-.C 13C.-.H 13H.-.H 2C.-.N 4H.-.N 2H.-.O N.-.N 3Hp
		0	3.196280956702	-2.26	3C.-.C 2C.-.N 11C.-.H 4H.-.N 11H.-.H 4H.-.O 3Hp
		9	4.151909209150	-1.30	
		5	4.246227247165	-1.21	
		8	4.314495473081	-1.14	

Continue in the next page

Table SM1: Structures of various conformations are evaluated for their energetic properties and types of intermolecular interactions. In this context, Am stands for amino acid, FM for functional monomer, N° conf. for the spatial conformation number of the Amino acid-FM complex, E_{tot} represents the ground state electronic energy in kcal mol^{-1}, the ΔE represents the difference of the electronic energy in ascending order of energy between the complex and lastly, the type of interaction specifies the atoms that are in close proximity in the table. The symbols Hb denote a hydrogen bond, AmtoM indicates that an AM is complexing with an FM, and MtoAm is the reverse of AmtoM. The symbols SB denote a salt bridges. The symbols Hp denote a hydrophobics interactions. The symbols Cation-pi/pi-staquing/pi-T-sheped denote the type of π interactions interactions.

AA	FM	N° conf.	ΔE	E_{tot}	Type of interaction
	alila	7	0.000000000000	-2.77	7C.-.H 3C.-.C 3C.-.O 10H.-.H 4H.-.O C.-.N 2H.-.N 3Hp SB
		8	1.129930512326	-1.64	2C.-.O 6C.-.H 2H.-.O C.-.C C.-.N 2H.-.N N.-.O 8H.-.H Hp SB
		1	1.267563823300	-1.50	6C.-.H 9H.-.H C.-.N 3H.-.N N.-.N SB
		9	1.381270925972	-1.39	
		5	2.184660312036	-0.58	
	estir	1	0.000000000000	-6.31	5C.-.C 28C.-.H 19H.-.H C.-.O 2H.-.O C.-.N H.-.N 5Hp
		3	0.725891740151	-5.58	23C.-.H 12H.-.H 5C.-.C 3C.-.N 4H.-.N H.-.O 5Hp
		4	1.200114830163	-5.10	22C.-.H 3C.-.C 6C.-.N 12H.-.H 3Hp Cation-pi(Amc)
		5	3.043217047999	-3.26	2C.-.O 18C.-.H 2H.-.O 16H.-.H H.-.N 2C.-.C 2Hp

Continue in the next page

Table SM1: Structures of various conformations are evaluated for their energetic properties and types of intermolecular interactions. In this context, Am stands for amino acid, FM for functional monomer, N° conf. for the spatial conformation number of the Amino acid-FM complex, E_{tot} represents the ground state electronic energy in kcal mol^{-1}, the ΔE represents the difference of the electronic energy in ascending order of energy between the complex and lastly, the type of interaction specifies the atoms that are in close proximity in the table. The symbols Hb denote a hydrogen bond, AmtoM indicates that an AM is complexing with an FM, and MtoAm is the reverse of AmtoM. The symbols SB denote a salt bridges. The symbols Hp denote a hydrophobics interactions. The symbols Cation-pi/pi-staquing/pi-T-sheped denote the type of π interactions interactions.

AA	FM	N° conf.	ΔE	E_{tot}	Type of interaction
		6	4.051202352556	-2.25	15C.-.H 14H.-.H 2C.-.C H.-.N 2Hp
		0	4.538061334353	-1.77	2C.-.O 2H.-.O C.-.H H.-.H
		7	4.790698732536	-1.51	2C.-.O H.-.H 2C.-.H 2H.-.O
		8	4.960892856160	-1.34	
		9	5.033076639319	-1.27	2C.-.O C.-.H 2H.-.O H.-.H
		2	5.810231191768	-0.49	
	1viny	0	0.000000000000	-6.37	7C.-.C 16C.-.H 6C.-.N 2C.-.O 12H.-.H 10H.-.N 2H.-.O N.-.N 7Hp Hb(AmtoM)
		6	1.904380154673	-4.46	5C.-.C 20C.-.H 14H.-.H 4C.-.N 9H.-.N C.-.O H.-.O 5Hp
		7	2.124710800556	-4.24	6H.-.N 12C.-.H 6C.-.C 3C.-.O 3C.-.N 9H.-.H 4H.-.O 6Hp
		8	4.079912074521	-2.29	2C.-.O 2C.-.H 5H.-.O H.-.H
		4	4.551759780896	-1.81	2C.-.N 8C.-.H 2C.-.O 2H.-.N 7H.-.H 3H.-.O
		3	5.006628699745	-1.36	

Continue in the next page

Table SM1: Structures of various conformations are evaluated for their energetic properties and types of intermolecular interactions. In this context, Am stands for amino acid, FM for functional monomer, N° conf. for the spatial conformation number of the Amino acid-FM complex, E_{tot} represents the ground state electronic energy in kcal mol^{-1}, the ΔE represents the difference of the electronic energy in ascending order of energy between the complex and lastly, the type of interaction specifies the atoms that are in close proximity in the table. The symbols Hb denote a hydrogen bond, AmtoM indicates that an AM is complexing with an FM, and MtoAm is the reverse of AmtoM. The symbols SB denote a salt bridges. The symbols Hp denote a hydrophobics interactions. The symbols Cation-pi/pi-staquing/pi-T-sheped denote the type of π interactions interactions.

AA	FM	N° conf.	ΔE	E_{tot}	Type of interaction
		9	5.152794778987	-1.21	
		1	5.209154646116	-1.16	
		5	6.061067636757	-0.30	
	2hydr	6	0.000000000000	-6.39	6H.-.O 2C.-.C 6C.-.H 4C.-.O 6H.-.H 2O.-.O 2Hp Hb(MtoAm)
		5	1.681735600866	-4.71	5C.-.C 13C.-.H 4C.-.N 11H.-.H 2H.-.N 6C.-.O 8H.-.O 2N.-.O 3O.-.O 5Hp
		2	2.994548727217	-3.40	11C.-.H 10H.-.H C.-.N 8H.-.O 3C.-.O N.-.O Hb(AmtoM)
		4	4.777961768995	-1.62	8C.-.H 6H.-.O C.-.O C.-.C 13H.-.H Hp
		0	5.561154483898	-0.83	
	4viny	5	0.000000000000	-5.59	10C.-.C 25C.-.H C.-.N 2H.-.N 2C.-.O 15H.-.H 3H.-.O 10Hp
		1	0.327835776441	-5.26	27C.-.H 5C.-.N 3C.-.C N.-.N 2H.-.N 17H.-.H 3Hp Cation-pi(Amc)

Continue in the next page

Table SM1: Structures of various conformations are evaluated for their energetic properties and types of intermolecular interactions. In this context, Am stands for amino acid, FM for functional monomer, N° conf. for the spatial conformation number of the Amino acid-FM complex, E_{tot} represents the ground state electronic energy in kcal mol^{-1}, the ΔE represents the difference of the electronic energy in ascending order of energy between the complex and lastly, the type of interaction specifies the atoms that are in close proximity in the table. The symbols Hb denote a hydrogen bond, AmtoM indicates that an AM is complexing with an FM, and MtoAm is the reverse of AmtoM. The symbols SB denote a salt bridges. The symbols Hp denote a hydrophobics interactions. The symbols Cation-pi/pi-staquing/pi-T-sheped denote the type of π interactions interactions.

AA	FM	N° conf.	ΔE	E_{tot}	Type of interaction
		7	3.612775381467	-1.98	2C.-.O 2C.-.H 3H.-.O H.-.H
		2	4.231429664816	-1.36	
	acrol	6	0.000000000000	-3.63	6C.-.H C.-.N 5H.-.H H.-.N 4H.-.O C.-.O N.-.O Hb(AmtoM)
		7	1.108761639164	-2.52	14C.-.H 4C.-.C 2C.-.N H.-.N 11H.-.H 3H.-.O C.-.O 4Hp
		8	1.261677371507	-2.37	2C.-.O 4C.-.H C.-.N 2H.-.O 4H.-.H H.-.N
		0	1.944900110927	-1.68	C.-.C 3C.-.O 4C.-.H 5H.-.O 2H.-.H 2O.-.O Hp
		4	2.348184195231	-1.28	
		5	3.203917525559	-0.42	
		9	3.435889801927	-0.19	
	itaco	8	0.000000000000	-7.72	2C.-.C 5C.-.O 4C.-.H 9H.-.O 4H.-.H C.-.N 2N.-.O 2Hp Hb(MtoAm) SB
		2	4.293645448270	-3.43	4C.-.H 2C.-.C 3C.-.O 3H.-.H 4H.-.O H.-.N 3O.-.O 2Hp SB

Continue in the next page

Table SM1: Structures of various conformations are evaluated for their energetic properties and types of intermolecular interactions. In this context, Am stands for amino acid, FM for functional monomer, Nº conf. for the spatial conformation number of the Amino acid-FM complex, E_{tot} represents the ground state electronic energy in kcal mol^{-1}, the ΔE represents the difference of the electronic energy in ascending order of energy between the complex and lastly, the type of interaction specifies the atoms that are in close proximity in the table. The symbols Hb denote a hydrogen bond, AmtoM indicates that an AM is complexing with an FM, and MtoAm is the reverse of AmtoM. The symbols SB denote a salt bridges. The symbols Hp denote a hydrophobics interactions. The symbols Cation-pi/pi-staquing/pi-T-sheped denote the type of π interactions interactions.

AA	FM	Nº conf.	ΔE	E_{tot}	Type of interaction
		6	6.402237445904	-1.32	
LEU	14dvb	3	0.000000000000	-5.51	20C.-.H 14H.-.H 10C.-.C 3C.-.N 2H.-.N 7C.-.O 4H.-.O 10Hp Cation-pi(Amc)
		0	0.058083200834	-5.45	27C.-.H 17H.-.H 7C.-.C 3C.-.N 2H.-.N 2C.-.O 2H.-.O 7Hp
		1	0.726668082690	-4.78	26C.-.H 2C.-.N 17H.-.H 3H.-.N 9C.-.C 9Hp
		6	2.688976696590	-2.82	C.-.C 21C.-.H 4C.-.N 15H.-.H 2H.-.N Hp
		8	5.422992041084	-0.09	
		4	5.466319027284	-0.04	
		5	5.537198901111	0.03	
	2viny	8	0.000000000000	-5.19	8C.-.C 16C.-.H 5C.-.N 5C.-.O 5H.-.N N.-.N 4H.-.O 12H.-.H 8Hp Cation-pi(Amc)

Continue in the next page

Table SM1: Structures of various conformations are evaluated for their energetic properties and types of intermolecular interactions. In this context, Am stands for amino acid, FM for functional monomer, N° conf. for the spatial conformation number of the Amino acid-FM complex, E_{tot} represents the ground state electronic energy in kcal mol^{-1}, the ΔE represents the difference of the electronic energy in ascending order of energy between the complex and lastly, the type of interaction specifies the atoms that are in close proximity in the table. The symbols Hb denote a hydrogen bond, AmtoM indicates that an AM is complexing with an FM, and MtoAm is the reverse of AmtoM. The symbols SB denote a salt bridges. The symbols Hp denote a hydrophobics interactions. The symbols Cation-pi/pi-staquing/pi-T-sheped denote the type of π interactions interactions.

AA	FM	N° conf.	ΔE	E_{tot}	Type of interaction
		7	1.273305393572	-3.92	24C.-.H 5C.-.C 5H.-.N 3C.-.N N.-.N 14H.-.H 5Hp Cation-pi(Amc)
		9	2.640564132046	-2.55	13C.-.H 2C.-.N 6H.-.N 9H.-.H C.-.O 3H.-.O
		5	3.974684827680	-1.22	C.-.N 2C.-.O 6C.-.H 6H.-.H 2H.-.N 3H.-.O
		2	4.112251756831	-1.08	7C.-.H 2C.-.N 9H.-.H 2H.-.N
		0	5.147246656113	-0.04	
	acidm	4	0.000000000000	-3.24	12C.-.H 3C.-.C C.-.N 11H.-.H 3C.-.O 8H.-.O N.-.O H.-.N 3Hp Hb(AmtoM) SB
		7	0.824788256548	-2.42	5C.-.C 15C.-.H 13H.-.H 8H.-.O 3C.-.O 5Hp
		0	0.911087730897	-2.33	9C.-.H 2C.-.C 10H.-.H 2C.-.O 4H.-.O 2Hp SB
		3	1.179238833327	-2.06	4C.-.C 16C.-.H 11H.-.H 3H.-.O 4Hp

Continue in the next page

Table SM1: Structures of various conformations are evaluated for their energetic properties and types of intermolecular interactions. In this context, Am stands for amino acid, FM for functional monomer, N° conf. for the spatial conformation number of the Amino acid-FM complex, E_{tot} represents the ground state electronic energy in kcal mol^{-1}, the ΔE represents the difference of the electronic energy in ascending order of energy between the complex and lastly, the type of interaction specifies the atoms that are in close proximity in the table. The symbols Hb denote a hydrogen bond, AmtoM indicates that an AM is complexing with an FM, and MtoAm is the reverse of AmtoM. The symbols SB denote a salt bridges. The symbols Hp denote a hydrophobics interactions. The symbols Cation-pi/pi-staquing/pi-T-sheped denote the type of π interactions interactions.

AA	FM	N° conf.	ΔE	E_{tot}	Type of interaction
		1	1.434222865810	-1.81	3C.-.C 16C.-.H 12H.-.H H.-.O 3Hp
		9	3.173702958815	-0.07	
		2	3.220427450247	-0.02	
		6	3.258076589065	0.01	
	acida	4	0.000000000000	-5.09	2C.-.C 13C.-.H 2C.-.N 9H.-.H H.-.N 9H.-.O 4C.-.O N.-.O 2Hp Hb(AmtoM) SB
		3	1.484445533156	-3.60	4C.-.O 4O.-.O 3H.-.O C.-.H H.-.H Hb(MtoAm) SB
		5	3.100929634569	-1.99	14C.-.H C.-.C 6C.-.O 10H.-.H 4H.-.O O.-.O Hp
		1	3.147622920084	-1.94	11C.-.H 2C.-.C 2C.-.N 13H.-.H H.-.N 4C.-.O 9H.-.O N.-.O O.-.O 2Hp SB
		2	5.040533663646	-0.05	
		0	5.053921385223	-0.03	
		7	5.060073939457	-0.03	

Continue in the next page

Table SM1: Structures of various conformations are evaluated for their energetic properties and types of intermolecular interactions. In this context, Am stands for amino acid, FM for functional monomer, N° conf. for the spatial conformation number of the Amino acid-FM complex, E_{tot} represents the ground state electronic energy in kcal mol^{-1}, the ΔE represents the difference of the electronic energy in ascending order of energy between the complex and lastly, the type of interaction specifies the atoms that are in close proximity in the table. The symbols Hb denote a hydrogen bond, AmtoM indicates that an AM is complexing with an FM, and MtoAm is the reverse of AmtoM. The symbols SB denote a salt bridges. The symbols Hp denote a hydrophobics interactions. The symbols Cation-pi/pi-staquing/pi-T-sheped denote the type of π interactions interactions.

AA	FM	N° conf.	ΔE	E_{tot}	Type of interaction
		9	5.081249880934	-0.01	
	bisac	8	0.000000000000	-6.72	3C.-.C 15C.-.H 14H.-.H 6C.-.O 7H.-.O 3H.-.N C.-.N N.-.O 3Hp Hb(MtoAm)
		9	3.960664778767	-2.76	16C.-.H 9H.-.N 14H.-.H N.-.N 2N.-.O C.-.C C.-.N 8H.-.O 4C.-.O Hp Hb(MtoAm) Hb(AmtoM)
		5	4.101194664812	-2.62	4C.-.O 2H.-.H 3C.-.H 7H.-.O C.-.N N.-.O 2O.-.O Hb(MtoAm)
		0	4.113327722569	-2.61	2C.-.C 15C.-.H 4C.-.O 4H.-.N C.-.N 13H.-.H 7H.-.O N.-.O 2O.-.O 2Hp
		7	6.797887231889	0.08	
	1ally	6	0.000000000000	-2.97	18C.-.H 3C.-.C 2C.-.O 21H.-.H 6H.-.O 4H.-.N 2C.-.N 3Hp 2SB
		7	0.441303941445	-2.53	5H.-.N 25C.-.H 4C.-.C 27H.-.H 2C.-.N 4Hp

Continue in the next page

Table SM1: Structures of various conformations are evaluated for their energetic properties and types of intermolecular interactions. In this context, Am stands for amino acid, FM for functional monomer, N° conf. for the spatial conformation number of the Amino acid-FM complex, E_{tot} represents the ground state electronic energy in kcal mol^{-1}, the ΔE represents the difference of the electronic energy in ascending order of energy between the complex and lastly, the type of interaction specifies the atoms that are in close proximity in the table. The symbols Hb denote a hydrogen bond, AmtoM indicates that an AM is complexing with an FM, and MtoAm is the reverse of AmtoM. The symbols SB denote a salt bridges. The symbols Hp denote a hydrophobics interactions. The symbols Cation-pi/pi-staquing/pi-T-sheped denote the type of π interactions interactions.

AA	FM	N° conf.	ΔE	E_{tot}	Type of interaction
		5	1.314673066197	-1.65	4H.-.N 17C.-.H 4C.-.C 20H.-.H 4Hp
		2	2.758036019275	-0.21	
		3	2.864101511578	-0.10	
		8	2.880407345085	-0.09	
	4imid	2	0.000000000000	-4.94	4H.-.O 3C.-.O 2O.-.O 4H.-.H 4C.-.H Hb(MtoAm)
		3	1.633535544054	-3.30	2C.-.O 4H.-.O 13H.-.H 6C.-.C 22C.-.H 3H.-.N 6Hp
		9	3.207645654991	-1.73	3C.-.O 7H.-.O N.-.O 5C.-.H 8H.-.H 2H.-.N 2C.-.N Hb(AmtoM) SB
		0	4.897530585211	-0.04	
		1	16.518087811085	11.58	
		4	44.760932020156	39.82	9C.-.O 9H.-.O 11C.-.H 7H.-.H C.-.C O.-.O H.-.N C.-.N N.-.O Hp Hb(MtoAm) SB

Continue in the next page

Table SM1: Structures of various conformations are evaluated for their energetic properties and types of intermolecular interactions. In this context, Am stands for amino acid, FM for functional monomer, N° conf. for the spatial conformation number of the Amino acid-FM complex, E_{tot} represents the ground state electronic energy in kcal mol^{-1}, the ΔE represents the difference of the electronic energy in ascending order of energy between the complex and lastly, the type of interaction specifies the atoms that are in close proximity in the table. The symbols Hb denote a hydrogen bond, AmtoM indicates that an AM is complexing with an FM, and MtoAm is the reverse of AmtoM. The symbols SB denote a salt bridges. The symbols Hp denote a hydrophobics interactions. The symbols Cation-pi/pi-staquing/pi-T-sheped denote the type of π interactions interactions.

AA	FM	N° conf.	ΔE	E_{tot}	Type of interaction
	acril	6	0.000000000000	-2.74	3C.-.O 2C.-.H 5H.-.O H.-.H C.-.N 2N.-.O Hb(MtoAm)
		0	0.547071289057	-2.19	4C.-.C 8C.-.H 9H.-.H H.-.N 3C.-.O 4H.-.O 4Hp
		2	0.645553804149	-2.09	C.-.N 2N.-.O 2C.-.H 4H.-.O 2H.-.H Hb(MtoAm)
		9	2.110755814006	-0.63	7C.-.H 7H.-.H
		1	2.652427773720	-0.09	
		8	2.767592008011	0.03	
		5	3.891695916460	1.15	
		3	5.293059777421	2.55	3C.-.C 13C.-.H 9H.-.H 5H.-.N 6H.-.O C.-.N 3C.-.O N.-.O 3Hp Hb(AmtoM)
	alila	6	0.000000000000	-2.93	4C.-.C 2C.-.N 16C.-.H 4H.-.N 17H.-.H H.-.O 4Hp
		5	1.186735269507	-1.74	14C.-.H 18H.-.H C.-.C H.-.N Hp
		8	1.247642449842	-1.68	11H.-.H 3C.-.H C.-.N 6H.-.N N.-.N

Continue in the next page

Table SM1: Structures of various conformations are evaluated for their energetic properties and types of intermolecular interactions. In this context, Am stands for amino acid, FM for functional monomer, N° conf. for the spatial conformation number of the Amino acid-FM complex, E_{tot} represents the ground state electronic energy in kcal mol^{-1}, the ΔE represents the difference of the electronic energy in ascending order of energy between the complex and lastly, the type of interaction specifies the atoms that are in close proximity in the table. The symbols Hb denote a hydrogen bond, AmtoM indicates that an AM is complexing with an FM, and MtoAm is the reverse of AmtoM. The symbols SB denote a salt bridges. The symbols Hp denote a hydrophobics interactions. The symbols Cation-pi/pi-staquing/pi-T-sheped denote the type of π interactions interactions.

AA	FM	N° conf.	ΔE	E_{tot}	Type of interaction
		1	1.397785025067	-1.53	C.-.C 10C.-.H 3C.-.N 13H.-.H 3H.-.N H.-.O Hp
		7	1.945824184379	-0.98	4C.-.C 15C.-.H 14H.-.H H.-.N 4Hp
		4	2.057444059031	-0.87	10C.-.H 12H.-.H 3H.-.N 2H.-.O C.-.N C.-.O SB
		9	2.125625320977	-0.80	C.-.C 6C.-.H C.-.N 11H.-.H 5H.-.N 3H.-.O Hp SB
		3	2.987844219866	0.06	
		0	3.004147742235	0.07	
	estir	2	0.000000000000	-5.39	25C.-.H 9C.-.C 16H.-.H 5C.-.O 5C.-.N 4H.-.O 2H.-.N 9Hp Cation-pi(Amc)
		5	0.479884307857	-4.91	26C.-.H 6C.-.C 19H.-.H 4C.-.N 3C.-.O 2H.-.N 3H.-.O 6Hp
		6	0.480819798250	-4.91	6C.-.C 21C.-.H 2C.-.N 18H.-.H 2H.-.N H.-.O 6Hp

Continue in the next page

Table SM1: Structures of various conformations are evaluated for their energetic properties and types of intermolecular interactions. In this context, Am stands for amino acid, FM for functional monomer, N° conf. for the spatial conformation number of the Amino acid-FM complex, E_{tot} represents the ground state electronic energy in $\mathrm{kcal\,mol^{-1}}$, the ΔE represents the difference of the electronic energy in ascending order of energy between the complex and lastly, the type of interaction specifies the atoms that are in close proximity in the table. The symbols Hb denote a hydrogen bond, AmtoM indicates that an AM is complexing with an FM, and MtoAm is the reverse of AmtoM. The symbols SB denote a salt bridges. The symbols Hp denote a hydrophobics interactions. The symbols Cation-pi/pi-staquing/pi-T-sheped denote the type of π interactions interactions.

AA	FM	N° conf.	ΔE	E_{tot}	Type of interaction
		4	0.508042483975	-4.88	27C.-.H 6C.-.N 17H.-.H 7C.-.C 7Hp Cation-pi(Amc)
		1	4.590444756924	-0.80	3C.-.H 2C.-.O H.-.H 2H.-.O
		0	5.283064344776	-0.11	
		3	5.410906425827	0.02	
		9	5.430025084504	0.04	
	1viny	9	0.000000000000	-5.43	20C.-.H 3C.-.C 6C.-.N 11H.-.N 14H.-.H N.-.N 3Hp Hb(AmtoM)
		0	1.087520023208	-4.34	7C.-.C 21C.-.H 5C.-.N 7H.-.N N.-.O 3C.-.O 17H.-.H 3H.-.O 7Hp
		7	1.979928001794	-3.45	14C.-.H 5C.-.C 11H.-.H 8H.-.N 3C.-.N N.-.O 2C.-.O 4H.-.O 5Hp
		3	2.299339912296	-3.13	10C.-.H 6C.-.N 6H.-.H 6H.-.N 2N.-.N 2N.-.O C.-.C 2C.-.O 2H.-.O Hp Cation-pi(Amc)
		8	5.406193365616	-0.02	
		4	5.409199957061	-0.02	

Continue in the next page

Table SM1: Structures of various conformations are evaluated for their energetic properties and types of intermolecular interactions. In this context, Am stands for amino acid, FM for functional monomer, N° conf. for the spatial conformation number of the Amino acid-FM complex, E_{tot} represents the ground state electronic energy in kcal mol^{-1}, the ΔE represents the difference of the electronic energy in ascending order of energy between the complex and lastly, the type of interaction specifies the atoms that are in close proximity in the table. The symbols Hb denote a hydrogen bond, AmtoM indicates that an AM is complexing with an FM, and MtoAm is the reverse of AmtoM. The symbols SB denote a salt bridges. The symbols Hp denote a hydrophobics interactions. The symbols Cation-pi/pi-staquing/pi-T-sheped denote the type of π interactions interactions.

AA	FM	N° conf.	ΔE	E_{tot}	Type of interaction
		1	5.412209359009	-0.02	
	2hydr	4	0.000000000000	-0.64	3C.-.O 3C.-.H H.-.N 5H.-.H 3H.-.O
		6	0.607423562965	-0.04	
		7	0.610996223359	-0.03	
		5	0.794079796620	0.15	
		1	32.853460107588	32.21	5H.-.O 2C.-.N 7C.-.H 3H.-.N 10H.-.H C.-.O N.-.O Hb(MtoAm)
	4viny	8	0.000000000000	-3.87	C.-.C 7C.-.H 7H.-.H 2C.-.N 7H.-.N N.-.N H.-.O Hp Hb(AmtoM)
		1	0.451764435886	-3.42	5C.-.C 23C.-.H 3H.-.N 16H.-.H 2C.-.N 5Hp
		3	2.216110995376	-1.65	3C.-.C 11C.-.H 9H.-.H 2C.-.O H.-.N 3H.-.O 3Hp
		5	3.795810660223	-0.07	
		4	3.852167720552	-0.02	

Continue in the next page

Table SM1: Structures of various conformations are evaluated for their energetic properties and types of intermolecular interactions. In this context, Am stands for amino acid, FM for functional monomer, N° conf. for the spatial conformation number of the Amino acid-FM complex, E_{tot} represents the ground state electronic energy in kcal mol^{-1}, the ΔE represents the difference of the electronic energy in ascending order of energy between the complex and lastly, the type of interaction specifies the atoms that are in close proximity in the table. The symbols Hb denote a hydrogen bond, AmtoM indicates that an AM is complexing with an FM, and MtoAm is the reverse of AmtoM. The symbols SB denote a salt bridges. The symbols Hp denote a hydrophobics interactions. The symbols Cation-pi/pi-staquing/pi-T-sheped denote the type of π interactions interactions.

AA	FM	N° conf.	ΔE	E_{tot}	Type of interaction
		6	3.861619865351	-0.01	
		0	3.919038826299	0.05	
	acrol	4	0.000000000000	-2.70	C.-.C 9C.-.H 6H.-.H H.-.N 3C.-.O 6H.-.O N.-.O Hp Hb(AmtoM)
		2	0.541853629981	-2.16	7C.-.H 5C.-.C 7H.-.H 4C.-.O 4H.-.O 2O.-.O 5Hp
		5	0.693561730531	-2.01	5C.-.C 13C.-.H 12H.-.H C.-.O H.-.O 5Hp
		6	1.365735200601	-1.33	10C.-.H C.-.C 7H.-.H C.-.O 2H.-.O O.-.O Hp
		9	2.663279874334	-0.04	
		8	2.697738268006	-0.00	2H.-.H
		0	2.828285031094	0.13	
		1	2.830669284847	0.13	
	itaco	0	0.000000000000	-8.68	9H.-.H 14C.-.H 8H.-.O 10C.-.O 3C.-.C 3O.-.O 3Hp Hb(AmtoM) SB

Continue in the next page

Table SM1: Structures of various conformations are evaluated for their energetic properties and types of intermolecular interactions. In this context, Am stands for amino acid, FM for functional monomer, N° conf. for the spatial conformation number of the Amino acid-FM complex, E_{tot} represents the ground state electronic energy in kcal mol^{-1}, the ΔE represents the difference of the electronic energy in ascending order of energy between the complex and lastly, the type of interaction specifies the atoms that are in close proximity in the table. The symbols Hb denote a hydrogen bond, AmtoM indicates that an AM is complexing with an FM, and MtoAm is the reverse of AmtoM. The symbols SB denote a salt bridges. The symbols Hp denote a hydrophobics interactions. The symbols Cation-pi/pi-staquing/pi-T-sheped denote the type of π interactions interactions.

AA	FM	N° conf.	ΔE	E_{tot}	Type of interaction
		3	4.417259509698	-4.26	4C.-.O 10C.-.H 13H.-.O 5H.-.H C.-.C 2N.-.O Hp 2SB
		4	7.059743297879	-1.62	
		8	7.646556819226	-1.03	8H.-.H 4C.-.O 6H.-.O 5C.-.H C.-.C 2O.-.O Hp SB
		2	8.522060229735	-0.15	
LYS	14dvb	7	0.000000000000	-5.44	30C.-.H 2C.-.O 11C.-.C 3H.-.O 2C.-.N 15H.-.H 3H.-.N 11Hp
		1	0.342664223179	-5.10	25C.-.H 3C.-.N 10C.-.C 2H.-.N 10H.-.H 3C.-.O 4H.-.O 10Hp
		0	3.284156498734	-2.16	2C.-.N 11C.-.H 2H.-.N 13H.-.H C.-.C Hp
		6	5.421265861761	-0.02	
		5	5.716373008652	0.27	
		9	5.781924508445	0.34	
	2viny	1	0.000000000000	-6.61	7C.-.C 26C.-.H 6C.-.N 9H.-.N N.-.N 18H.-.H 7Hp Hb(AmtoM)

Continue in the next page

Table SM1: Structures of various conformations are evaluated for their energetic properties and types of intermolecular interactions. In this context, Am stands for amino acid, FM for functional monomer, N° conf. for the spatial conformation number of the Amino acid-FM complex, E_{tot} represents the ground state electronic energy in kcal mol^{-1}, the ΔE represents the difference of the electronic energy in ascending order of energy between the complex and lastly, the type of interaction specifies the atoms that are in close proximity in the table. The symbols Hb denote a hydrogen bond, AmtoM indicates that an AM is complexing with an FM, and MtoAm is the reverse of AmtoM. The symbols SB denote a salt bridges. The symbols Hp denote a hydrophobics interactions. The symbols Cation-pi/pi-staquing/pi-T-sheped denote the type of π interactions interactions.

AA	FM	N° conf.	ΔE	E_{tot}	Type of interaction
		6	1.832167714416	-4.78	6C.-.C 13C.-.H 5C.-.N 6H.-.N N.-.N 2N.-.O 10H.-.H C.-.O H.-.O 6Hp
		9	2.697381042288	-3.92	11C.-.C 20C.-.H 4C.-.N 3H.-.N N.-.N 15H.-.H C.-.O 2H.-.O 11Hp Cation-pi(Amc)
		5	5.208752207623	-1.40	8C.-.H 2C.-.N C.-.C 3H.-.N 7H.-.H Hp
		2	5.497269500343	-1.12	9C.-.H 8H.-.H C.-.O H.-.O
		0	6.614059286261	0.00	
	acidm	6	0.000000000000	-14.86	5C.-.H C.-.N C.-.C 2N.-.O 10H.-.O 3C.-.O Hp Hb(AmtoM) SB
		9	9.628114586191	-5.23	2C.-.N 9C.-.C 20C.-.H 3H.-.N 18H.-.H 9H.-.O 3C.-.O N.-.O 9Hp Hb(AmtoM) 2SB

Continue in the next page

Table SM1: Structures of various conformations are evaluated for their energetic properties and types of intermolecular interactions. In this context, Am stands for amino acid, FM for functional monomer, N° conf. for the spatial conformation number of the Amino acid-FM complex, E_{tot} represents the ground state electronic energy in kcal mol^{-1}, the ΔE represents the difference of the electronic energy in ascending order of energy between the complex and lastly, the type of interaction specifies the atoms that are in close proximity in the table. The symbols Hb denote a hydrogen bond, AmtoM indicates that an AM is complexing with an FM, and MtoAm is the reverse of AmtoM. The symbols SB denote a salt bridges. The symbols Hp denote a hydrophobics interactions. The symbols Cation-pi/pi-staquing/pi-T-sheped denote the type of π interactions interactions.

AA	FM	N° conf.	ΔE	E_{tot}	Type of interaction
		7	11.975462049082	-2.88	8H.-.H 7C.-.H C.-.C 4C.-.O 10H.-.O N.-.O Hp Hb(AmtoM) SB
		8	12.968791732620	-1.89	12C.-.H 7H.-.H 3C.-.C 4C.-.O 7H.-.O 3Hp SB
		1	13.774060088024	-1.08	10C.-.H C.-.N 13H.-.H 2H.-.N
		4	14.929113807914	0.07	
		2	15.014610916489	0.16	
		3	15.218541232329	0.36	
	acida	9	0.000000000000	-5.66	8C.-.H 5H.-.H C.-.C 6C.-.O 9H.-.O 2O.-.O Hp Hb(MtoAm) SB
		7	0.027285827269	-5.64	3C.-.O 3O.-.O 3H.-.O 2H.-.H 2C.-.H Hb(MtoAm)
		8	2.630903051849	-3.03	17C.-.H 4C.-.C 15H.-.H 2H.-.N 4H.-.O N.-.O 4Hp Hb(AmtoM) SB

Continue in the next page

Table SM1: Structures of various conformations are evaluated for their energetic properties and types of intermolecular interactions. In this context, Am stands for amino acid, FM for functional monomer, N° conf. for the spatial conformation number of the Amino acid-FM complex, E_{tot} represents the ground state electronic energy in kcal mol^{-1}, the ΔE represents the difference of the electronic energy in ascending order of energy between the complex and lastly, the type of interaction specifies the atoms that are in close proximity in the table. The symbols Hb denote a hydrogen bond, AmtoM indicates that an AM is complexing with an FM, and MtoAm is the reverse of AmtoM. The symbols SB denote a salt bridges. The symbols Hp denote a hydrophobics interactions. The symbols Cation-pi/pi-staquing/pi-T-sheped denote the type of π interactions interactions.

AA	FM	N° conf.	ΔE	E_{tot}	Type of interaction
		1	4.034294851753	-1.63	2C.-.N 7C.-.H C.-.C H.-.N 6H.-.H N.-.O 4H.-.O C.-.O Hp Hb(AmtoM) SB
		0	5.053073757813	-0.61	2C.-.H C.-.N 2N.-.O 4H.-.O H.-.N 2H.-.H Hb(AmtoM) SB
		6	5.714199294089	0.05	
		2	5.717098482367	0.05	
	bisac	5	0.000000000000	-6.55	32C.-.H 10C.-.C 4C.-.O 23H.-.H 11H.-.O 8H.-.N 3C.-.N N.-.O 10Hp Hb(AmtoM)
		4	3.619184292089	-2.93	25C.-.H 4C.-.N 4C.-.C 20H.-.H 10H.-.N 7H.-.O 2C.-.O 4Hp
		2	3.933263037762	-2.62	7C.-.O 7H.-.O 13C.-.H 9H.-.H 4C.-.C 2O.-.O 3N.-.O C.-.N 4Hp Hb(MtoAm)
	1ally	3	0.000000000000	-5.81	11H.-.N 2C.-.C 26C.-.H 6C.-.N 28H.-.H N.-.N 2Hp Hb(AmtoM)

Continue in the next page

Table SM1: Structures of various conformations are evaluated for their energetic properties and types of intermolecular interactions. In this context, Am stands for amino acid, FM for functional monomer, Nº conf. for the spatial conformation number of the Amino acid-FM complex, E_{tot} represents the ground state electronic energy in kcal mol^{-1}, the ΔE represents the difference of the electronic energy in ascending order of energy between the complex and lastly, the type of interaction specifies the atoms that are in close proximity in the table. The symbols Hb denote a hydrogen bond, AmtoM indicates that an AM is complexing with an FM, and MtoAm is the reverse of AmtoM. The symbols SB denote a salt bridges. The symbols Hp denote a hydrophobics interactions. The symbols Cation-pi/pi-staquing/pi-T-sheped denote the type of π interactions interactions.

AA	FM	Nº conf.	ΔE	E_{tot}	Type of interaction
		2	3.043460586717	-2.76	22C.-.H 4C.-.C C.-.O H.-.N 18H.-.H 4H.-.O 4Hp 2SB
		4	3.330341179997	-2.48	C.-.N N.-.O 3H.-.N 19C.-.H 6H.-.O 20H.-.H 3C.-.O 2C.-.C 2Hp 2SB
		1	3.721943588623	-2.09	3C.-.C 2C.-.O 9H.-.H 11C.-.H 5H.-.O 3Hp SB
		9	5.501760216527	-0.31	
		6	5.649907024692	-0.16	
		8	5.941688967473	0.13	
	4imid	9	0.000000000000	-8.96	2N.-.O 7H.-.O 4C.-.O H.-.N 6H.-.H 5C.-.H C.-.N C.-.C Hp Hb(MtoAm) SB
		1	4.076733660359	-4.88	2C.-.O 7H.-.O N.-.O 5C.-.C 21C.-.H 13H.-.H 5H.-.N 5Hp Hb(AmtoM) Cation-pi(Amc) SB

Continue in the next page

Table SM1: Structures of various conformations are evaluated for their energetic properties and types of intermolecular interactions. In this context, Am stands for amino acid, FM for functional monomer, N° conf. for the spatial conformation number of the Amino acid-FM complex, E_{tot} represents the ground state electronic energy in kcal mol^{-1}, the ΔE represents the difference of the electronic energy in ascending order of energy between the complex and lastly, the type of interaction specifies the atoms that are in close proximity in the table. The symbols Hb denote a hydrogen bond, AmtoM indicates that an AM is complexing with an FM, and MtoAm is the reverse of AmtoM. The symbols SB denote a salt bridges. The symbols Hp denote a hydrophobics interactions. The symbols Cation-pi/pi-staquing/pi-T-sheped denote the type of π interactions interactions.

AA	FM	N° conf.	ΔE	E_{tot}	Type of interaction
		4	5.141269893587	-3.82	8H.-.H 2C.-.N 2H.-.N N.-.O 2C.-.O 10C.-.H C.-.C 3H.-.O Hp Hb(MtoAm)
	acril	1	0.000000000000	-2.13	C.-.C 5C.-.O 3C.-.H 7H.-.O 3N.-.O 2O.-.O C.-.N 2H.-.H Hp Hb(MtoAm)
		6	0.635422491584	-1.50	3C.-.H C.-.N N.-.N 2H.-.N N.-.O 3H.-.O 3H.-.H Hb(AmtoM)
		2	2.110146562220	-0.02	
		7	2.174255528070	0.04	
		9	2.536867411835	0.40	
	alila	7	0.000000000000	-4.42	3C.-.N 6C.-.H 8H.-.N 10H.-.H N.-.N Hb(AmtoM) SB
		9	0.952144625350	-3.46	3C.-.N 2C.-.C 15C.-.H N.-.N 7H.-.N 15H.-.H 2Hp Hb(MtoAm)
		5	2.167473142396	-2.25	15C.-.H 4C.-.C 2C.-.N 20H.-.H 8H.-.N 4Hp

Continue in the next page

Table SM1: Structures of various conformations are evaluated for their energetic properties and types of intermolecular interactions. In this context, Am stands for amino acid, FM for functional monomer, N° conf. for the spatial conformation number of the Amino acid-FM complex, E_{tot} represents the ground state electronic energy in kcal mol^{-1}, the ΔE represents the difference of the electronic energy in ascending order of energy between the complex and lastly, the type of interaction specifies the atoms that are in close proximity in the table. The symbols Hb denote a hydrogen bond, AmtoM indicates that an AM is complexing with an FM, and MtoAm is the reverse of AmtoM. The symbols SB denote a salt bridges. The symbols Hp denote a hydrophobics interactions. The symbols Cation-pi/pi-staquing/pi-T-sheped denote the type of π interactions interactions.

AA	FM	N° conf.	ΔE	E_{tot}	Type of interaction
		1	2.309166681733	-2.11	9C.-.H 3C.-.C 5C.-.O 6H.-.H 7H.-.O C.-.N 2N.-.O H.-.N 3Hp Hb(MtoAm) SB
		3	3.052886914978	-1.36	C.-.N 5C.-.H 6H.-.N 12H.-.H N.-.N
		6	3.686727879555	-0.73	C.-.C 8C.-.H 13H.-.H 3H.-.N C.-.N Hp
		8	4.391410903063	-0.02	
		2	4.511674929966	0.10	
	estir	1	0.000000000000	-4.95	26C.-.H 8C.-.C 3C.-.N 12H.-.H 2H.-.N C.-.O 2H.-.O 8Hp Cation-pi(Amc)
		0	0.211620912460	-4.74	11C.-.C 22C.-.H 12H.-.H 2C.-.N 4C.-.O 2H.-.N 3H.-.O 11Hp
		2	3.067767333899	-1.89	11C.-.H 7H.-.H 3C.-.C 4C.-.O H.-.N 5H.-.O 3Hp
		6	4.799587379000	-0.15	
		7	4.838414342712	-0.12	

Continue in the next page

Table SM1: Structures of various conformations are evaluated for their energetic properties and types of intermolecular interactions. In this context, Am stands for amino acid, FM for functional monomer, N° conf. for the spatial conformation number of the Amino acid-FM complex, E_{tot} represents the ground state electronic energy in kcal mol^{-1}, the ΔE represents the difference of the electronic energy in ascending order of energy between the complex and lastly, the type of interaction specifies the atoms that are in close proximity in the table. The symbols Hb denote a hydrogen bond, AmtoM indicates that an AM is complexing with an FM, and MtoAm is the reverse of AmtoM. The symbols SB denote a salt bridges. The symbols Hp denote a hydrophobics interactions. The symbols Cation-pi/pi-staquing/pi-T-sheped denote the type of π interactions interactions.

AA	FM	N° conf.	ΔE	E_{tot}	Type of interaction
		4	4.976585071164	0.02	
		8	5.160799097118	0.21	
	1viny	3	0.000000000000	-4.19	26C.-.H 6C.-.C 6C.-.N 10H.-.N N.-.N 16H.-.H 6Hp
		6	0.353854853854	-3.84	4C.-.N 6C.-.C 21C.-.H 9H.-.N 15H.-.H N.-.N H.-.O 6Hp Hb(AmtoM)
		4	0.526882554525	-3.66	6C.-.N 18C.-.H 8H.-.N 2N.-.N N.-.O 2C.-.O 4C.-.C 12H.-.H 2H.-.O 4Hp Cation-pi(Amc)
		0	0.655555122830	-3.53	19C.-.H 6C.-.C 12H.-.H 3C.-.N 5H.-.N 3H.-.O C.-.O 6Hp
		1	1.747899443649	-2.44	12C.-.H 2C.-.C 3C.-.O H.-.N 8H.-.H 3H.-.O 2Hp
		8	2.274753372273	-1.91	3C.-.H 4H.-.H N.-.N 3H.-.N Hb(AmtoM)
	2hydr	2	0.000000000000	-2.52	21C.-.H 5C.-.C 20H.-.H 2H.-.N 3H.-.O 5Hp

Continue in the next page

Table SM1: Structures of various conformations are evaluated for their energetic properties and types of intermolecular interactions. In this context, Am stands for amino acid, FM for functional monomer, N° conf. for the spatial conformation number of the Amino acid-FM complex, E_{tot} represents the ground state electronic energy in kcal mol^{-1}, the ΔE represents the difference of the electronic energy in ascending order of energy between the complex and lastly, the type of interaction specifies the atoms that are in close proximity in the table. The symbols Hb denote a hydrogen bond, AmtoM indicates that an AM is complexing with an FM, and MtoAm is the reverse of AmtoM. The symbols SB denote a salt bridges. The symbols Hp denote a hydrophobics interactions. The symbols Cation-pi/pi-staquing/pi-T-sheped denote the type of π interactions interactions.

AA	FM	N° conf.	ΔE	E_{tot}	Type of interaction
		4	2.655018751876	0.14	
	4viny	6	0.000000000000	-5.20	28C.-.H 16H.-.H 14C.-.C 2H.-.N 3H.-.O C.-.O 14Hp
		9	0.931922078604	-4.27	3C.-.N 24C.-.H 9C.-.C N.-.N 6H.-.N 17H.-.H H.-.O 9Hp Hb(AmtoM)
		1	1.020894707844	-4.18	7C.-.C 17C.-.H 2C.-.O 4C.-.N 2H.-.N 2N.-.O 13H.-.H 2H.-.O 7Hp Cation-pi(Amc)
		8	2.099102747135	-3.10	20C.-.H 3C.-.C 10H.-.H 2H.-.N 3C.-.N 3Hp Cation-pi(Amc)
		0	5.083276554264	-0.11	
		2	5.172223515400	-0.03	
		4	15.490870099383	10.29	7C.-.C 25C.-.H 16H.-.H 5H.-.N 2C.-.N 7Hp Cation-pi(Amc)
	acrol	9	0.000000000000	-2.60	4C.-.C 14C.-.H 14H.-.H H.-.N N.-.O 2C.-.O 4H.-.O 4Hp Hb(AmtoM)

Continue in the next page

Table SM1: Structures of various conformations are evaluated for their energetic properties and types of intermolecular interactions. In this context, Am stands for amino acid, FM for functional monomer, N° conf. for the spatial conformation number of the Amino acid-FM complex, E_{tot} represents the ground state electronic energy in kcal mol^{-1}, the ΔE represents the difference of the electronic energy in ascending order of energy between the complex and lastly, the type of interaction specifies the atoms that are in close proximity in the table. The symbols Hb denote a hydrogen bond, AmtoM indicates that an AM is complexing with an FM, and MtoAm is the reverse of AmtoM. The symbols SB denote a salt bridges. The symbols Hp denote a hydrophobics interactions. The symbols Cation-pi/pi-staquing/pi-T-sheped denote the type of π interactions interactions.

AA	FM	N° conf.	ΔE	E_{tot}	Type of interaction
		8	0.479551937626	-2.12	6C.-.O 3C.-.C 6C.-.H C.-.N 6H.-.O N.-.O O.-.O 4H.-.H H.-.N 3Hp
		4	0.489519851504	-2.11	13C.-.H 2C.-.N 12H.-.H 3C.-.C 2H.-.N 2C.-.O 4H.-.O 3Hp
		3	0.572241504287	-2.03	5C.-.C 2C.-.N 12C.-.H 2H.-.N 8H.-.H 4C.-.O 4H.-.O 2O.-.O 5Hp
		2	0.772819460646	-1.83	10C.-.H 2C.-.N 2C.-.C 2H.-.N 7H.-.H H.-.O 2Hp
		0	0.822137349707	-1.78	13C.-.H 5C.-.C 4C.-.O 7H.-.O 7H.-.H 5Hp
		7	2.580360260782	-0.02	
		1	2.700584524973	0.10	
		6	2.782461065832	0.18	
	itaco	4	0.000000000000	-5.60	2C.-.O C.-.H 3O.-.O 4H.-.O H.-.H Hb(AmtoM) SB
		9	3.588217868204	-2.01	

Continue in the next page

Table SM1: Structures of various conformations are evaluated for their energetic properties and types of intermolecular interactions. In this context, Am stands for amino acid, FM for functional monomer, N° conf. for the spatial conformation number of the Amino acid-FM complex, E_{tot} represents the ground state electronic energy in kcal mol^{-1}, the ΔE represents the difference of the electronic energy in ascending order of energy between the complex and lastly, the type of interaction specifies the atoms that are in close proximity in the table. The symbols Hb denote a hydrogen bond, AmtoM indicates that an AM is complexing with an FM, and MtoAm is the reverse of AmtoM. The symbols SB denote a salt bridges. The symbols Hp denote a hydrophobics interactions. The symbols Cation-pi/pi-staquing/pi-T-sheped denote the type of π interactions interactions.

AA	FM	N° conf.	ΔE	E_{tot}	Type of interaction
		8	4.328970044139	-1.27	8H.-.H 8H.-.O 13C.-.H C.-.C 2C.-.O N.-.O H.-.N Hp Hb(MtoAm) SB
MET	14dvb	8	0.000000000000	-4.80	35C.-.H 2C.-.N 3H.-.N 18H.-.H 7C.-.C 7Hp
		9	2.006795753565	-2.79	5C.-.C 17C.-.H 14H.-.H 2H.-.S C.-.S 2C.-.O 2H.-.O 5Hp
		9	2.006795753565	-2.79	erro
		2	3.518901249164	-1.28	14C.-.H 13H.-.H H.-.N 2C.-.C H.-.S 2Hp
		0	4.185468572824	-0.61	
		7	4.837880372853	0.04	
	2viny	9	0.000000000000	-3.93	12C.-.C 23C.-.H 3C.-.N 5H.-.N 16H.-.H C.-.O H.-.O 12Hp Cation-pi(Amc)
		0	0.031605224446	-3.90	6C.-.C 20C.-.H H.-.N 2C.-.O 3C.-.S 11H.-.H 2H.-.O 6Hp

Continue in the next page

Table SM1: Structures of various conformations are evaluated for their energetic properties and types of intermolecular interactions. In this context, Am stands for amino acid, FM for functional monomer, Nº conf. for the spatial conformation number of the Amino acid-FM complex, E_{tot} represents the ground state electronic energy in kcal mol^{-1}, the ΔE represents the difference of the electronic energy in ascending order of energy between the complex and lastly, the type of interaction specifies the atoms that are in close proximity in the table. The symbols Hb denote a hydrogen bond, AmtoM indicates that an AM is complexing with an FM, and MtoAm is the reverse of AmtoM. The symbols SB denote a salt bridges. The symbols Hp denote a hydrophobics interactions. The symbols Cation-pi/pi-staquing/pi-T-sheped denote the type of π interactions interactions.

AA	FM	Nº conf.	ΔE	E_{tot}	Type of interaction
		5	0.606144284117	-3.32	24C.-.H 4C.-.C 5H.-.N 17H.-.H 2C.-.N 4Hp
		3	1.535570136161	-2.39	6C.-.C 14C.-.H 3C.-.S 2C.-.N 2H.-.N N.-.O 8H.-.H 2H.-.S 3C.-.O 3H.-.O 6Hp Cation-pi(Amc)
		1	1.688168154069	-2.24	11C.-.H 8H.-.H 2C.-.C C.-.S 2H.-.S 2Hp
		6	1.936517370964	-1.99	C.-.C 9C.-.H 5H.-.H 2H.-.S 2H.-.O C.-.O Hp
		8	3.887449318987	-0.04	
		7	3.921980193555	-0.01	
	acidm	4	0.000000000000	-6.96	9C.-.H 2C.-.C C.-.N C.-.S 9H.-.H 7C.-.O 10H.-.O 2O.-.S N.-.O H.-.S 2Hp Hb(MtoAm) Hb(AmtoM) SB

Continue in the next page

Table SM1: Structures of various conformations are evaluated for their energetic properties and types of intermolecular interactions. In this context, Am stands for amino acid, FM for functional monomer, N° conf. for the spatial conformation number of the Amino acid-FM complex, E_{tot} represents the ground state electronic energy in kcal mol^{-1}, the ΔE represents the difference of the electronic energy in ascending order of energy between the complex and lastly, the type of interaction specifies the atoms that are in close proximity in the table. The symbols Hb denote a hydrogen bond, AmtoM indicates that an AM is complexing with an FM, and MtoAm is the reverse of AmtoM. The symbols SB denote a salt bridges. The symbols Hp denote a hydrophobics interactions. The symbols Cation-pi/pi-staquing/pi-T-sheped denote the type of π interactions interactions.

AA	FM	N° conf.	ΔE	E_{tot}	Type of interaction
		0	4.513788652381	-2.45	13C.-.H 12H.-.H H.-.S 2C.-.C 2C.-.N 3C.-.O 2H.-.N 4H.-.O O.-.O 2Hp SB
		5	5.877357467991	-1.08	10C.-.H 11H.-.H C.-.C H.-.S Hp
		8	7.258343687888	0.30	
	acida	7	0.000000000000	-3.78	2C.-.O 15C.-.H 4H.-.O 11H.-.H 4C.-.C 2H.-.N N.-.O 2C.-.S 2O.-.S 4Hp Hb(MtoAm) SB
		8	0.718372702000	-3.06	4C.-.C 16C.-.H 9H.-.H C.-.S H.-.S 5H.-.O C.-.O 4Hp
		0	2.726117714850	-1.05	9C.-.H 7H.-.H H.-.S 3H.-.O 2C.-.N 2H.-.N N.-.O Hb(MtoAm) SB
		4	3.635701158596	-0.14	5C.-.H 4H.-.H 3H.-.O H.-.N C.-.O O.-.O
		6	3.769849013105	-0.01	
		3	3.787389159554	0.01	
		1	3.889367257296	0.11	

Continue in the next page

Table SM1: Structures of various conformations are evaluated for their energetic properties and types of intermolecular interactions. In this context, Am stands for amino acid, FM for functional monomer, N° conf. for the spatial conformation number of the Amino acid-FM complex, E_{tot} represents the ground state electronic energy in kcal mol^{-1}, the ΔE represents the difference of the electronic energy in ascending order of energy between the complex and lastly, the type of interaction specifies the atoms that are in close proximity in the table. The symbols Hb denote a hydrogen bond, AmtoM indicates that an AM is complexing with an FM, and MtoAm is the reverse of AmtoM. The symbols SB denote a salt bridges. The symbols Hp denote a hydrophobics interactions. The symbols Cation-pi/pi-staquing/pi-T-sheped denote the type of π interactions interactions.

AA	FM	N° conf.	ΔE	E_{tot}	Type of interaction
		2	4.323659708634	0.55	
	bisac	4	0.000000000000	-7.17	22C.-.H C.-.S 3C.-.C 16H.-.H 2H.-.S 7H.-.N N.-.S C.-.N 3C.-.O 5H.-.O N.-.O 3Hp Hb(MtoAm) Hb(AmtoM)
		0	2.032476977065	-5.14	6C.-.O 6H.-.H 10H.-.O 2H.-.N C.-.N 2N.-.O 10C.-.H 2O.-.O Hb(MtoAm)
		9	2.088042906793	-5.08	15C.-.H 13H.-.H 3H.-.N 5C.-.O 8H.-.O C.-.C H.-.S C.-.N N.-.O Hp Hb(MtoAm)
		1	3.780314857394	-3.39	4C.-.O 3C.-.H 6H.-.O C.-.N 2N.-.O H.-.H Hb(MtoAm)
		8	3.805529158107	-3.36	5C.-.C 19C.-.H 16H.-.H 5H.-.N 2C.-.O 4H.-.O 2C.-.N 5Hp
		2	4.434932999358	-2.73	13C.-.H 8H.-.N C.-.O 4H.-.O N.-.O 2C.-.N 15H.-.H C.-.C Hp Hb(AmtoM)

Continue in the next page

Table SM1: Structures of various conformations are evaluated for their energetic properties and types of intermolecular interactions. In this context, Am stands for amino acid, FM for functional monomer, N° conf. for the spatial conformation number of the Amino acid-FM complex, E_{tot} represents the ground state electronic energy in kcal mol^{-1}, the ΔE represents the difference of the electronic energy in ascending order of energy between the complex and lastly, the type of interaction specifies the atoms that are in close proximity in the table. The symbols Hb denote a hydrogen bond, AmtoM indicates that an AM is complexing with an FM, and MtoAm is the reverse of AmtoM. The symbols SB denote a salt bridges. The symbols Hp denote a hydrophobics interactions. The symbols Cation-pi/pi-staquing/pi-T-sheped denote the type of π interactions interactions.

AA	FM	N° conf.	ΔE	E_{tot}	Type of interaction
		7	7.131564785674	-0.04	
	1ally	7	0.000000000000	-2.80	3C.-.C 19C.-.H 20H.-.H C.-.N 3H.-.N C.-.S H.-.S 3Hp
		1	1.942432758277	-0.85	3H.-.H
		3	2.646697706288	-0.15	
		9	2.651341387277	-0.15	
		4	2.850023623960	0.05	
	4imid	5	0.000000000000	-5.36	2O.-.S 25C.-.H 3C.-.S 12C.-.C 2C.-.O 4H.-.O 8H.-.H H.-.S C.-.N 3H.-.N N.-.O 12Hp Cation-pi(Amc)
		4	0.728099488014	-4.63	4C.-.C 17C.-.H 2C.-.O 2H.-.O O.-.S 7H.-.H 4C.-.N 6H.-.N N.-.N 4Hp Hb(AmtoM)
		0	2.127212933381	-3.24	5H.-.O 9C.-.C 23C.-.H 2C.-.O 9H.-.H 2H.-.N 9Hp SB
		7	2.460268433737	-2.90	3C.-.H 7H.-.H 5H.-.N 2C.-.N N.-.N Hb(AmtoM)

Continue in the next page

Table SM1: Structures of various conformations are evaluated for their energetic properties and types of intermolecular interactions. In this context, Am stands for amino acid, FM for functional monomer, N° conf. for the spatial conformation number of the Amino acid-FM complex, E_{tot} represents the ground state electronic energy in $\mathrm{kcal\,mol^{-1}}$, the ΔE represents the difference of the electronic energy in ascending order of energy between the complex and lastly, the type of interaction specifies the atoms that are in close proximity in the table. The symbols Hb denote a hydrogen bond, AmtoM indicates that an AM is complexing with an FM, and MtoAm is the reverse of AmtoM. The symbols SB denote a salt bridges. The symbols Hp denote a hydrophobics interactions. The symbols Cation-pi/pi-staquing/pi-T-sheped denote the type of π interactions interactions.

AA	FM	N° conf.	ΔE	E_{tot}	Type of interaction
		8	5.301479572300	-0.06	
	acril	8	0.000000000000	-3.71	4C.-.C 14C.-.H 2C.-.N 12H.-.H H.-.N 5H.-.O C.-.S 2H.-.S 2C.-.O N.-.O 4Hp Hb(AmtoM)
		2	0.610582802189	-3.09	13C.-.H 14H.-.H 3C.-.C 2C.-.O 4H.-.O C.-.S H.-.S 6H.-.N 2C.-.N N.-.N 3Hp
		9	1.875571813261	-1.83	C.-.N 2C.-.H 4H.-.O 2C.-.O N.-.O 2H.-.H H.-.N Hb(AmtoM)
		4	3.610613315045	-0.09	
		7	3.954186579807	0.25	
	alila	3	0.000000000000	-4.22	C.-.C 14C.-.H 2C.-.N 19H.-.H 8H.-.N N.-.N Hp Hb(MtoAm)
		9	1.451239666882	-2.77	2C.-.C 10C.-.H C.-.N 10H.-.H H.-.N H.-.S 3C.-.O 4H.-.O N.-.O 2Hp Hb(AmtoM) SB

Continue in the next page

Table SM1: Structures of various conformations are evaluated for their energetic properties and types of intermolecular interactions. In this context, Am stands for amino acid, FM for functional monomer, N° conf. for the spatial conformation number of the Amino acid-FM complex, E_{tot} represents the ground state electronic energy in kcal mol^{-1}, the ΔE represents the difference of the electronic energy in ascending order of energy between the complex and lastly, the type of interaction specifies the atoms that are in close proximity in the table. The symbols Hb denote a hydrogen bond, AmtoM indicates that an AM is complexing with an FM, and MtoAm is the reverse of AmtoM. The symbols SB denote a salt bridges. The symbols Hp denote a hydrophobics interactions. The symbols Cation-pi/pi-staquing/pi-T-sheped denote the type of π interactions interactions.

AA	FM	N° conf.	ΔE	E_{tot}	Type of interaction
		0	1.640977943141	-2.58	2C.-.C 12C.-.H 12H.-.H 3H.-.N C.-.S 2H.-.S 3H.-.O N.-.O 2Hp Hb(AmtoM) SB
		7	2.829598257913	-1.39	2C.-.C 16C.-.H 2C.-.N 20H.-.H 6H.-.N C.-.S 3H.-.S N.-.N 2Hp Hb(MtoAm) SB
		4	4.368134284336	0.15	
		2	4.609970133701	0.39	
		5	4.694448146071	0.47	
	estir	6	0.000000000000	-4.93	29C.-.H 8C.-.C 18H.-.H 4C.-.N 3H.-.N 8Hp
		0	0.603542225634	-4.33	8C.-.C 33C.-.H 18H.-.H 3C.-.N 2H.-.N 8Hp
		9	1.293159983635	-3.64	17C.-.H 6C.-.C 11H.-.H 3H.-.S H.-.O C.-.S 6Hp
		5	1.778168274910	-3.15	16C.-.H 11H.-.H 5C.-.C 2C.-.S 4H.-.O C.-.O H.-.N 5Hp
		1	3.891509735386	-1.04	11H.-.H C.-.C 9C.-.H H.-.N Hp

Continue in the next page

Table SM1: Structures of various conformations are evaluated for their energetic properties and types of intermolecular interactions. In this context, Am stands for amino acid, FM for functional monomer, N° conf. for the spatial conformation number of the Amino acid-FM complex, E_{tot} represents the ground state electronic energy in kcal mol^{-1}, the ΔE represents the difference of the electronic energy in ascending order of energy between the complex and lastly, the type of interaction specifies the atoms that are in close proximity in the table. The symbols Hb denote a hydrogen bond, AmtoM indicates that an AM is complexing with an FM, and MtoAm is the reverse of AmtoM. The symbols SB denote a salt bridges. The symbols Hp denote a hydrophobics interactions. The symbols Cation-pi/pi-staquing/pi-T-sheped denote the type of π interactions interactions.

AA	FM	N° conf.	ΔE	E_{tot}	Type of interaction
		4	4.865132421952	-0.06	
		2	5.285516027872	0.36	
		7	5.354554721472	0.43	
	1viny	9	0.000000000000	-5.95	12C.-.H 3C.-.C C.-.S 4C.-.N 7H.-.N N.-.N 11H.-.H 2H.-.S 3Hp Hb(AmtoM)
		1	0.574244589452	-5.37	5C.-.C 15C.-.H C.-.S 7H.-.N 5C.-.N 11H.-.H H.-.S N.-.N 5Hp Hb(AmtoM)
		7	1.768723744017	-4.18	23C.-.H 3C.-.N 8H.-.N 5C.-.C 15H.-.H 5Hp Cation-pi(Amc)
		4	2.426197538142	-3.52	21C.-.H 5C.-.N N.-.N 10H.-.N 4C.-.C 16H.-.H 4Hp Hb(AmtoM)
		5	5.975007562901	0.03	
		2	6.063839964235	0.12	
		0	9.269153558029	3.32	2C.-.N 6C.-.H N.-.N 4H.-.N 5H.-.H H.-.O

Continue in the next page

Table SM1: Structures of various conformations are evaluated for their energetic properties and types of intermolecular interactions. In this context, Am stands for amino acid, FM for functional monomer, Nº conf. for the spatial conformation number of the Amino acid-FM complex, E_{tot} represents the ground state electronic energy in kcal mol^{-1}, the ΔE represents the difference of the electronic energy in ascending order of energy between the complex and lastly, the type of interaction specifies the atoms that are in close proximity in the table. The symbols Hb denote a hydrogen bond, AmtoM indicates that an AM is complexing with an FM, and MtoAm is the reverse of AmtoM. The symbols SB denote a salt bridges. The symbols Hp denote a hydrophobics interactions. The symbols Cation-pi/pi-staquing/pi-T-sheped denote the type of π interactions interactions.

AA	FM	Nº conf.	ΔE	E_{tot}	Type of interaction
	2hydr	5	0.000000000000	-7.47	21C.-.H 16H.-.H 2C.-.S 4C.-.C 8H.-.O 2O.-.S 2H.-.S 3C.-.O O.-.O 4Hp Hb(MtoAm)
		0	3.427763668568	-4.04	24C.-.H 8C.-.C C.-.S 20H.-.H 2H.-.S 4C.-.O 8H.-.O 2O.-.O H.-.N 8Hp
		7	5.451599496124	-2.02	19C.-.H 3C.-.N 3C.-.O N.-.O 7H.-.O 4H.-.N 25H.-.H 3C.-.C 3Hp Hb(AmtoM)
		4	7.535132496553	0.07	
		9	7.658826858342	0.19	
	4viny	6	0.000000000000	-4.37	9C.-.C 26C.-.H 19H.-.H 6C.-.N 6H.-.N C.-.O H.-.O 9Hp
		3	0.803412534908	-3.57	9C.-.C 26C.-.H 2C.-.O 2H.-.N 14H.-.H 2H.-.O 9Hp
		1	1.908839135019	-2.46	7C.-.H C.-.O 6H.-.H 2H.-.O 2C.-.N 4H.-.N
		5	4.503264315962	0.13	

Continue in the next page

Table SM1: Structures of various conformations are evaluated for their energetic properties and types of intermolecular interactions. In this context, Am stands for amino acid, FM for functional monomer, N° conf. for the spatial conformation number of the Amino acid-FM complex, E_{tot} represents the ground state electronic energy in kcal mol^{-1}, the ΔE represents the difference of the electronic energy in ascending order of energy between the complex and lastly, the type of interaction specifies the atoms that are in close proximity in the table. The symbols Hb denote a hydrogen bond, AmtoM indicates that an AM is complexing with an FM, and MtoAm is the reverse of AmtoM. The symbols SB denote a salt bridges. The symbols Hp denote a hydrophobics interactions. The symbols Cation-pi/pi-staquing/pi-T-sheped denote the type of π interactions interactions.

AA	FM	N° conf.	ΔE	E_{tot}	Type of interaction
	acrol	6	0.000000000000	-4.07	5C.-.C 15C.-.H 12H.-.H C.-.N H.-.N 2C.-.S 2H.-.S O.-.S 3H.-.O C.-.O 5Hp
		5	1.848838749731	-2.22	5C.-.C 17C.-.H 2C.-.O 14H.-.H 4H.-.O 2C.-.N 2H.-.N 5Hp
		2	1.891653734132	-2.18	5C.-.C 16C.-.H 2C.-.O 16H.-.H 4H.-.O C.-.N H.-.N 5Hp
		0	2.950550204891	-1.12	3C.-.C 8C.-.H 5H.-.H 2H.-.O C.-.O O.-.O 3Hp
		7	3.215201637801	-0.86	C.-.C 3C.-.H 3C.-.O 4H.-.O 2H.-.H Hp
		3	4.089983291661	0.02	
		4	4.116352281032	0.04	
		8	4.231909629420	0.16	
	itaco	8	0.000000000000	-14.06	5C.-.O 8H.-.O 2N.-.O 2O.-.O 2C.-.C 6C.-.H C.-.N 2H.-.H 2Hp 2Hb(AmtoM) SB

Continue in the next page

Table SM1: Structures of various conformations are evaluated for their energetic properties and types of intermolecular interactions. In this context, Am stands for amino acid, FM for functional monomer, N° conf. for the spatial conformation number of the Amino acid-FM complex, E_{tot} represents the ground state electronic energy in kcal mol^{-1}, the ΔE represents the difference of the electronic energy in ascending order of energy between the complex and lastly, the type of interaction specifies the atoms that are in close proximity in the table. The symbols Hb denote a hydrogen bond, AmtoM indicates that an AM is complexing with an FM, and MtoAm is the reverse of AmtoM. The symbols SB denote a salt bridges. The symbols Hp denote a hydrophobics interactions. The symbols Cation-pi/pi-staquing/pi-T-sheped denote the type of π interactions interactions.

AA	FM	N° conf.	ΔE	E_{tot}	Type of interaction
		3	2.830847199728	-11.23	9H.-.O 4C.-.O 2O.-.S 5C.-.H 9H.-.H H.-.S 2C.-.C C.-.S 2Hp Hb(MtoAm)
		2	8.205070171071	-5.86	2C.-.C 7C.-.O 9H.-.O 5C.-.H 3H.-.H O.-.O 2Hp Hb(MtoAm)
		4	8.465494881951	-5.60	10H.-.O 7H.-.H 12C.-.H 4C.-.O 2C.-.N H.-.N N.-.O Hb(AmtoM) 2SB
		6	9.580785073222	-4.48	6C.-.O 14H.-.O 2C.-.C 16C.-.H C.-.N 11H.-.H H.-.N N.-.O O.-.S H.-.S 2Hp Hb(AmtoM) 2SB
		9	12.660667272289	-1.40	11C.-.H 2C.-.C 11H.-.H H.-.N H.-.S C.-.O 2H.-.O 2Hp
PHE	14dvb	3	0.000000000000	-5.16	31C.-.H 15H.-.H 22C.-.C 22Hp pi-staquing
		6	2.136672805549	-3.03	16C.-.H 2C.-.O 10H.-.H 2H.-.O C.-.C Hp

Continue in the next page

Table SM1: Structures of various conformations are evaluated for their energetic properties and types of intermolecular interactions. In this context, Am stands for amino acid, FM for functional monomer, Nº conf. for the spatial conformation number of the Amino acid-FM complex, E_{tot} represents the ground state electronic energy in kcal mol^{-1}, the ΔE represents the difference of the electronic energy in ascending order of energy between the complex and lastly, the type of interaction specifies the atoms that are in close proximity in the table. The symbols Hb denote a hydrogen bond, AmtoM indicates that an AM is complexing with an FM, and MtoAm is the reverse of AmtoM. The symbols SB denote a salt bridges. The symbols Hp denote a hydrophobics interactions. The symbols Cation-pi/pi-staquing/pi-T-sheped denote the type of π interactions interactions.

AA	FM	Nº conf.	ΔE	E_{tot}	Type of interaction
		2	2.422269959463	-2.74	7C.-.C 30C.-.H 13H.-.H 2C.-.O 2H.-.O 7Hp Cation-pi(Amc)
		5	3.618261469863	-1.55	4C.-.O H.-.N 4H.-.H 5C.-.H 5H.-.O C.-.C Hp
		0	4.396028801418	-0.77	H.-.H
		9	5.253300031751	0.09	
		7	5.472159795286	0.31	
	2viny	5	0.000000000000	-5.09	2C.-.N 17C.-.H 2C.-.C N.-.N 6H.-.N 9H.-.H C.-.O H.-.O 2Hp Hb(AmtoM) pi-T-sheped
		2	0.582061912041	-4.51	4C.-.N 20C.-.H 7C.-.C 3C.-.O 4H.-.N 13H.-.H 3H.-.O 7Hp
		4	0.727372775039	-4.37	15C.-.C 26C.-.H C.-.N 2H.-.N 14H.-.H 15Hp pi-staquing
		8	0.917853554770	-4.18	4C.-.N 19C.-.H 3C.-.C 4H.-.N 2C.-.O 15H.-.H 2H.-.O 3Hp pi-T-sheped

Continue in the next page

Table SM1: Structures of various conformations are evaluated for their energetic properties and types of intermolecular interactions. In this context, Am stands for amino acid, FM for functional monomer, N° conf. for the spatial conformation number of the Amino acid-FM complex, E_{tot} represents the ground state electronic energy in kcal mol^{-1}, the ΔE represents the difference of the electronic energy in ascending order of energy between the complex and lastly, the type of interaction specifies the atoms that are in close proximity in the table. The symbols Hb denote a hydrogen bond, AmtoM indicates that an AM is complexing with an FM, and MtoAm is the reverse of AmtoM. The symbols SB denote a salt bridges. The symbols Hp denote a hydrophobics interactions. The symbols Cation-pi/pi-staquing/pi-T-sheped denote the type of π interactions interactions.

AA	FM	N° conf.	ΔE	E_{tot}	Type of interaction
		6	1.241369741012	-3.85	16C.-.C 22C.-.H 6C.-.N 11H.-.H 16Hp pi-staquing
		3	1.330856653843	-3.76	2C.-.N 18C.-.H 6C.-.C 2H.-.N 12H.-.H 6Hp
		9	1.987352364988	-3.11	4C.-.C 13C.-.H 3C.-.O C.-.N 2H.-.N N.-.O 7H.-.H 4H.-.O 4Hp
		0	5.102977958935	0.01	
		7	5.237652385355	0.14	
	acidm	2	0.000000000000	-5.84	6H.-.H 9C.-.H C.-.C 5C.-.O 9H.-.O 2O.-.O N.-.O H.-.N Hp Hb(AmtoM) Hb(MtoAm) SB
		9	2.290652332905	-3.55	6C.-.H 6C.-.O 5H.-.H 7H.-.O 2O.-.O Hb(MtoAm) SB
		8	4.872111546016	-0.97	2C.-.C 11C.-.H 4C.-.O 6H.-.H 6H.-.O H.-.N 2Hp
		0	5.795711507723	-0.04	
		6	5.894524517241	0.05	
		4	5.983792891644	0.14	

Continue in the next page

Table SM1: Structures of various conformations are evaluated for their energetic properties and types of intermolecular interactions. In this context, Am stands for amino acid, FM for functional monomer, N° conf. for the spatial conformation number of the Amino acid-FM complex, E_{tot} represents the ground state electronic energy in kcal mol^{-1}, the ΔE represents the difference of the electronic energy in ascending order of energy between the complex and lastly, the type of interaction specifies the atoms that are in close proximity in the table. The symbols Hb denote a hydrogen bond, AmtoM indicates that an AM is complexing with an FM, and MtoAm is the reverse of AmtoM. The symbols SB denote a salt bridges. The symbols Hp denote a hydrophobics interactions. The symbols Cation-pi/pi-staquing/pi-T-sheped denote the type of π interactions interactions.

AA	FM	N° conf.	ΔE	E_{tot}	Type of interaction
		3	6.336368648419	0.50	
		1	6.990013950056	1.15	C.-.N 19C.-.H 11C.-.C 16H.-.H 2H.-.N 4H.-.O 4C.-.O 11Hp SB
	acida	9	0.000000000000	-6.09	5C.-.H C.-.N 3C.-.O 2N.-.O 6H.-.O 2O.-.O H.-.N 3H.-.H Hb(MtoAm) Hb(AmtoM) SB
		5	2.437888642847	-3.65	5C.-.O 3C.-.H 6H.-.O 2H.-.H 2O.-.O Hb(AmtoM)
		1	3.336911583657	-2.75	8C.-.C 16C.-.H 10H.-.H 8C.-.O 5H.-.O 8Hp
		0	4.086126735884	-2.01	C.-.N 12C.-.H C.-.O 7H.-.H H.-.N 3H.-.O
		6	5.857931345283	-0.23	
		2	6.042935919852	-0.05	
		4	6.255372326016	0.16	
	bisac	6	0.000000000000	-4.25	4C.-.C 19C.-.H 13H.-.H 6C.-.N H.-.N 2H.-.O C.-.O 4Hp

Continue in the next page

Table SM1: Structures of various conformations are evaluated for their energetic properties and types of intermolecular interactions. In this context, Am stands for amino acid, FM for functional monomer, N° conf. for the spatial conformation number of the Amino acid-FM complex, E_{tot} represents the ground state electronic energy in kcal mol^{-1}, the ΔE represents the difference of the electronic energy in ascending order of energy between the complex and lastly, the type of interaction specifies the atoms that are in close proximity in the table. The symbols Hb denote a hydrogen bond, AmtoM indicates that an AM is complexing with an FM, and MtoAm is the reverse of AmtoM. The symbols SB denote a salt bridges. The symbols Hp denote a hydrophobics interactions. The symbols Cation-pi/pi-staquing/pi-T-sheped denote the type of π interactions interactions.

AA	FM	N° conf.	ΔE	E_{tot}	Type of interaction
		0	0.105993179286	-4.14	7H.-.H 6C.-.H 2N.-.O 5H.-.O O.-.O 4H.-.N 3C.-.O Hb(AmtoM) Hb(MtoAm)
		4	1.101521107158	-3.15	C.-.N 6C.-.C 15C.-.H N.-.N 4H.-.N 10H.-.H 2C.-.O 3H.-.O 6Hp
		3	1.577635714234	-2.67	13C.-.H 3C.-.C 2C.-.O C.-.N 4H.-.N 9H.-.H 5H.-.O 3Hp
		2	2.539683770450	-1.71	2H.-.N 13C.-.H 7H.-.H 4C.-.C C.-.N 3C.-.O 3H.-.O 4Hp
	1ally	9	0.000000000000	-4.30	32C.-.H 9C.-.C 24H.-.H 4H.-.N C.-.N 9Hp Cation-pi(Mc)
		1	0.501624739491	-3.80	9C.-.C 22H.-.H 29C.-.H 3C.-.N 6H.-.N 9Hp
		4	0.513029507332	-3.79	N.-.N 7H.-.N 2C.-.N 15H.-.H 10C.-.H 2H.-.O Hb(AmtoM) SB
		5	0.846588337520	-3.46	18C.-.H 5C.-.C 4H.-.N 18H.-.H 5Hp

Continue in the next page

Table SM1: Structures of various conformations are evaluated for their energetic properties and types of intermolecular interactions. In this context, Am stands for amino acid, FM for functional monomer, N° conf. for the spatial conformation number of the Amino acid-FM complex, E_{tot} represents the ground state electronic energy in kcal mol^{-1}, the ΔE represents the difference of the electronic energy in ascending order of energy between the complex and lastly, the type of interaction specifies the atoms that are in close proximity in the table. The symbols Hb denote a hydrogen bond, AmtoM indicates that an AM is complexing with an FM, and MtoAm is the reverse of AmtoM. The symbols SB denote a salt bridges. The symbols Hp denote a hydrophobics interactions. The symbols Cation-pi/pi-staquing/pi-T-sheped denote the type of π interactions interactions.

AA	FM	N° conf.	ΔE	E_{tot}	Type of interaction
		7	1.993575556365	-2.31	C.-.N H.-.N 2C.-.C 10C.-.H 12H.-.H 2Hp
		6	3.170908011132	-1.13	2C.-.H 6H.-.H
		0	4.193699150483	-0.11	
		2	4.379038056916	0.08	
		8	5.262944531271	0.96	
	4imid	5	0.000000000000	-5.19	8H.-.O 9C.-.O 19C.-.H 9C.-.C 11H.-.H 4C.-.N 5H.-.N 2N.-.O 9Hp SB
		8	1.815377191749	-3.37	15C.-.H 8H.-.H 5C.-.C 3C.-.O 5H.-.N 2N.-.O 3C.-.N 3H.-.O 5Hp
		3	1.904554205336	-3.28	11C.-.H 7H.-.H 3C.-.C 5H.-.N C.-.N C.-.O H.-.O 3Hp pi-T-sheped
		9	3.125022459330	-2.06	8C.-.H C.-.O H.-.O 2C.-.C 2H.-.H C.-.N H.-.N 2Hp

Continue in the next page

Table SM1: Structures of various conformations are evaluated for their energetic properties and types of intermolecular interactions. In this context, Am stands for amino acid, FM for functional monomer, Nº conf. for the spatial conformation number of the Amino acid-FM complex, E_{tot} represents the ground state electronic energy in $kcal\,mol^{-1}$, the ΔE represents the difference of the electronic energy in ascending order of energy between the complex and lastly, the type of interaction specifies the atoms that are in close proximity in the table. The symbols Hb denote a hydrogen bond, AmtoM indicates that an AM is complexing with an FM, and MtoAm is the reverse of AmtoM. The symbols SB denote a salt bridges. The symbols Hp denote a hydrophobics interactions. The symbols Cation-pi/pi-staquing/pi-T-sheped denote the type of π interactions interactions.

AA	FM	Nº conf.	ΔE	E_{tot}	Type of interaction
		7	3.240557239010	-1.94	8C.-.H N.-.O 3H.-.O O.-.O 3H.-.N 6H.-.H 2C.-.N Hb(AmtoM) SB
		6	3.918764118701	-1.27	N.-.O 5H.-.O 2C.-.N 5C.-.H 2H.-.N 3H.-.H 2C.-.O SB
		6	3.918764118701	-1.27	erro
		1	4.943877375118	-0.24	4H.-.H 4C.-.H 2C.-.N 2H.-.N C.-.C Hp
	acril	0	0.000000000000	-9.90	3C.-.H C.-.C 3C.-.O 2O.-.O 4H.-.O C.-.N 2N.-.O H.-.N 2H.-.H Hp Hb(AmtoM) Hb(MtoAm)
		2	4.695989780022	-5.21	8C.-.H 6H.-.H 3C.-.N 4C.-.O 4H.-.N 2N.-.O 6H.-.O O.-.O Hb(MtoAm) Hb(AmtoM)
		1	7.131022869783	-2.77	4C.-.H 2C.-.O 3H.-.H C.-.N 2N.-.O H.-.N O.-.O 5H.-.O Hb(AmtoM)

Continue in the next page

Table SM1: Structures of various conformations are evaluated for their energetic properties and types of intermolecular interactions. In this context, Am stands for amino acid, FM for functional monomer, N° conf. for the spatial conformation number of the Amino acid-FM complex, E_{tot} represents the ground state electronic energy in $kcal\,mol^{-1}$, the ΔE represents the difference of the electronic energy in ascending order of energy between the complex and lastly, the type of interaction specifies the atoms that are in close proximity in the table. The symbols Hb denote a hydrogen bond, AmtoM indicates that an AM is complexing with an FM, and MtoAm is the reverse of AmtoM. The symbols SB denote a salt bridges. The symbols Hp denote a hydrophobics interactions. The symbols Cation-pi/pi-staquing/pi-T-sheped denote the type of π interactions interactions.

AA	FM	N° conf.	ΔE	E_{tot}	Type of interaction
		8	7.582865397029	-2.32	12C.-.H 9H.-.H 4C.-.C 2H.-.O C.-.O 3C.-.N 3H.-.N 4Hp
		4	9.236911704700	-0.66	C.-.N 2C.-.C 5C.-.H C.-.O 2H.-.N 6H.-.H 5H.-.O 2Hp
		7	9.504925547563	-0.40	2C.-.H 2C.-.O 2H.-.O H.-.N H.-.H
		6	9.643315286884	-0.26	
		5	9.904923734978	0.00	H.-.O
	alila	2	0.000000000000	-3.57	2C.-.C 12C.-.H 13H.-.H 2C.-.N 5H.-.N N.-.N 2Hp Hb(MtoAm) SB
		0	1.386808586119	-2.18	3C.-.N 12C.-.H 2C.-.O 3H.-.N 10H.-.H 3H.-.O
		3	1.671546625026	-1.90	C.-.C 3C.-.O 5C.-.H 5H.-.O 5H.-.H Hp
		1	3.399903136424	-0.17	
		6	3.672716930676	0.10	
		8	3.690189890805	0.12	

Continue in the next page

Table SM1: Structures of various conformations are evaluated for their energetic properties and types of intermolecular interactions. In this context, Am stands for amino acid, FM for functional monomer, N° conf. for the spatial conformation number of the Amino acid-FM complex, E_{tot} represents the ground state electronic energy in $kcal\,mol^{-1}$, the ΔE represents the difference of the electronic energy in ascending order of energy between the complex and lastly, the type of interaction specifies the atoms that are in close proximity in the table. The symbols Hb denote a hydrogen bond, AmtoM indicates that an AM is complexing with an FM, and MtoAm is the reverse of AmtoM. The symbols SB denote a salt bridges. The symbols Hp denote a hydrophobics interactions. The symbols Cation-pi/pi-staquing/pi-T-sheped denote the type of π interactions interactions.

AA	FM	N° conf.	ΔE	E_{tot}	Type of interaction
		7	3.733529494245	0.16	
		4	3.787811629903	0.22	
	estir	9	0.000000000000	-4.50	2C.-.N 7C.-.C 21C.-.H 2C.-.O 2H.-.N 12H.-.H 2H.-.O 7Hp
		8	0.284761430845	-4.22	24C.-.C 28C.-.H 16H.-.H C.-.O 2H.-.O 24Hp pi-staquing
		6	0.398210090780	-4.10	19C.-.H 3C.-.C 2C.-.N C.-.O 9H.-.H 2H.-.N H.-.O 3Hp pi-T-sheped Cation-pi(Amc)
		3	4.487578477670	-0.01	
		5	4.563466856583	0.06	
		4	4.677617498580	0.18	
		2	4.927646383290	0.43	
		1	5.194958697729	0.70	
	1viny	4	0.000000000000	-3.32	10C.-.H 4C.-.N 2C.-.C C.-.O N.-.N 6H.-.N N.-.O 4H.-.H 2H.-.O 2Hp Hb(AmtoM)

Continue in the next page

Table SM1: Structures of various conformations are evaluated for their energetic properties and types of intermolecular interactions. In this context, Am stands for amino acid, FM for functional monomer, N° conf. for the spatial conformation number of the Amino acid-FM complex, E_{tot} represents the ground state electronic energy in kcal mol^{-1}, the ΔE represents the difference of the electronic energy in ascending order of energy between the complex and lastly, the type of interaction specifies the atoms that are in close proximity in the table. The symbols Hb denote a hydrogen bond, AmtoM indicates that an AM is complexing with an FM, and MtoAm is the reverse of AmtoM. The symbols SB denote a salt bridges. The symbols Hp denote a hydrophobics interactions. The symbols Cation-pi/pi-staquing/pi-T-sheped denote the type of π interactions interactions.

AA	FM	N° conf.	ΔE	E_{tot}	Type of interaction
		2	0.074168516117	-3.25	7C.-.C 13C.-.H 9H.-.H 7Hp pi-T-sheped
		1	1.822986463566	-1.50	2C.-.C 9C.-.H C.-.O 2H.-.N N.-.O 6H.-.H H.-.O 2Hp
		0	2.326385287136	-1.00	2C.-.O 12C.-.H N.-.O 2H.-.O 8H.-.H
		6	2.585902549615	-0.74	3H.-.N C.-.N 6C.-.H C.-.C 6H.-.H Hp
		9	3.511897680729	0.19	
	2hydr	9	0.000000000000	-4.89	4C.-.N 26C.-.H 3H.-.N 23H.-.H 8C.-.C 2N.-.O 9H.-.O 3C.-.O O.-.O 8Hp Hb(MtoAm)
		2	1.833475853925	-3.05	4H.-.O 4C.-.C 23C.-.H 9H.-.H 4C.-.O 4Hp
		5	4.823604721263	-0.06	
		8	4.979587994333	0.09	
		4	5.275052625777	0.39	8C.-.H 6C.-.O 7H.-.H 7H.-.O O.-.O

Continue in the next page

Table SM1: Structures of various conformations are evaluated for their energetic properties and types of intermolecular interactions. In this context, Am stands for amino acid, FM for functional monomer, N° conf. for the spatial conformation number of the Amino acid-FM complex, E_{tot} represents the ground state electronic energy in kcal mol^{-1}, the ΔE represents the difference of the electronic energy in ascending order of energy between the complex and lastly, the type of interaction specifies the atoms that are in close proximity in the table. The symbols Hb denote a hydrogen bond, AmtoM indicates that an AM is complexing with an FM, and MtoAm is the reverse of AmtoM. The symbols SB denote a salt bridges. The symbols Hp denote a hydrophobics interactions. The symbols Cation-pi/pi-staquing/pi-T-sheped denote the type of π interactions interactions.

AA	FM	N° conf.	ΔE	E_{tot}	Type of interaction
	4viny	6	0.000000000000	-2.88	14C.-.H 3C.-.N 3H.-.N 8H.-.H 7C.-.C 8C.-.O 4H.-.O 7Hp
		1	0.925488415202	-1.96	4C.-.C 6C.-.O 8C.-.H C.-.N 2H.-.N 7H.-.H 4H.-.O 4Hp
		7	1.061663104012	-1.82	C.-.C 8C.-.H C.-.O C.-.N 2H.-.N 6H.-.H 4H.-.O Hp
		0	2.879073869527	-0.00	
		2	3.039759034516	0.16	
	acrol	9	0.000000000000	-5.45	4C.-.O 4C.-.H 4H.-.H 3H.-.O 2O.-.O Hb(AmtoM)
		5	1.162339664530	-4.28	2C.-.N 24C.-.H 15C.-.C 12H.-.H H.-.N N.-.O 4H.-.O 2C.-.O 15Hp Hb(AmtoM)
		1	1.658919315675	-3.79	3C.-.N 21C.-.H 7C.-.C 13H.-.H 2H.-.N N.-.O 2H.-.O 2C.-.O 7Hp
		0	3.416049368438	-2.03	5C.-.C 12C.-.H 9H.-.H 2C.-.O 2H.-.O 5Hp

Continue in the next page

Table SM1: Structures of various conformations are evaluated for their energetic properties and types of intermolecular interactions. In this context, Am stands for amino acid, FM for functional monomer, N° conf. for the spatial conformation number of the Amino acid-FM complex, E_{tot} represents the ground state electronic energy in kcal mol^{-1}, the ΔE represents the difference of the electronic energy in ascending order of energy between the complex and lastly, the type of interaction specifies the atoms that are in close proximity in the table. The symbols Hb denote a hydrogen bond, AmtoM indicates that an AM is complexing with an FM, and MtoAm is the reverse of AmtoM. The symbols SB denote a salt bridges. The symbols Hp denote a hydrophobics interactions. The symbols Cation-pi/pi-staquing/pi-T-sheped denote the type of π interactions interactions.

AA	FM	N° conf.	ΔE	E_{tot}	Type of interaction
		6	3.463180482101	-1.98	9C.-.H 2C.-.C 7H.-.H N.-.O 3H.-.O 2Hp Hb(AmtoM)
		2	4.313345792885	-1.13	5C.-.C 14C.-.H 9H.-.H 3C.-.O 5H.-.O O.-.O 5Hp
		3	5.326619467522	-0.12	
		7	5.358248004417	-0.09	
		4	5.455572135153	0.01	
	itaco	5	0.000000000000	-4.48	3H.-.O 13C.-.C 19C.-.H 9H.-.H C.-.O 13Hp
		7	0.279714627167	-4.20	10C.-.H 5C.-.O 7H.-.O O.-.O 6H.-.H N.-.O Hb(AmtoM) SB
		9	0.967056233165	-3.51	8H.-.O 6C.-.O 3C.-.N 13C.-.H 5C.-.C 10H.-.H N.-.O H.-.N 5Hp 2SB
PRO	14dvb	6	0.000000000000	-0.41	
		8	0.297734526465	-0.12	
		7	0.433813041084	0.02	

Continue in the next page

Table SM1: Structures of various conformations are evaluated for their energetic properties and types of intermolecular interactions. In this context, Am stands for amino acid, FM for functional monomer, N° conf. for the spatial conformation number of the Amino acid-FM complex, E_{tot} represents the ground state electronic energy in $kcal\,mol^{-1}$, the ΔE represents the difference of the electronic energy in ascending order of energy between the complex and lastly, the type of interaction specifies the atoms that are in close proximity in the table. The symbols Hb denote a hydrogen bond, AmtoM indicates that an AM is complexing with an FM, and MtoAm is the reverse of AmtoM. The symbols SB denote a salt bridges. The symbols Hp denote a hydrophobics interactions. The symbols Cation-pi/pi-staquing/pi-T-sheped denote the type of π interactions interactions.

AA	FM	N° conf.	ΔE	E_{tot}	Type of interaction
	2viny	6	0.000000000000	-9.72	4C.-.O 8C.-.H C.-.N 2N.-.O H.-.N C.-.C 4H.-.O 4H.-.H Hp Hb(AmtoM)
		1	0.631623200715	-9.09	5C.-.O 8C.-.H C.-.N 2N.-.O H.-.N C.-.C 4H.-.O 4H.-.H Hp Hb(AmtoM)
		0	4.211819234276	-5.51	7C.-.N 20C.-.H 8C.-.C N.-.N N.-.O 6H.-.N C.-.O 9H.-.H H.-.O 8Hp Hb(AmtoM)
		8	5.169572991968	-4.55	14C.-.H 7C.-.C 4H.-.N 4C.-.N N.-.N 11H.-.H H.-.O 7Hp Cation-pi(Amc)
		9	6.362917503050	-3.35	16C.-.H 8C.-.C 3C.-.O 2H.-.N C.-.N N.-.O H.-.O 10H.-.H 8Hp Cation-pi(Amc)
		7	7.019206278678	-2.70	7C.-.C 16C.-.H 2C.-.N 3H.-.N 8H.-.H 7Hp Cation-pi(Amc)

Continue in the next page

Table SM1: Structures of various conformations are evaluated for their energetic properties and types of intermolecular interactions. In this context, Am stands for amino acid, FM for functional monomer, Nº conf. for the spatial conformation number of the Amino acid-FM complex, E_{tot} represents the ground state electronic energy in $kcal\,mol^{-1}$, the ΔE represents the difference of the electronic energy in ascending order of energy between the complex and lastly, the type of interaction specifies the atoms that are in close proximity in the table. The symbols Hb denote a hydrogen bond, AmtoM indicates that an AM is complexing with an FM, and MtoAm is the reverse of AmtoM. The symbols SB denote a salt bridges. The symbols Hp denote a hydrophobics interactions. The symbols Cation-pi/pi-staquing/pi-T-sheped denote the type of π interactions interactions.

AA	FM	Nº conf.	ΔE	E_{tot}	Type of interaction
		3	8.188216295106	-1.53	4C.-.H 2C.-.O 2H.-.N 3H.-.H 2H.-.O
	acidm	9	0.000000000000	-12.51	7C.-.H 2C.-.C C.-.N 8H.-.O 4C.-.O 2N.-.O 4H.-.H H.-.N 2Hp Hb(MtoAm) SB
		5	7.634477219067	-4.87	2C.-.O C.-.H 2H.-.H 3O.-.O 3H.-.O Hb(AmtoM)
		1	8.661836422935	-3.85	C.-.C 3C.-.O 4C.-.H 6H.-.O 3H.-.H 2O.-.O Hp Hb(AmtoM)
		6	8.782988030986	-3.72	4C.-.C 4C.-.O 19C.-.H 14H.-.H 2C.-.N 4H.-.O 2O.-.O 2H.-.N 4Hp SB
		2	10.008232142074	-2.50	12C.-.H 7H.-.H 8H.-.O 4C.-.C C.-.N 5C.-.O H.-.N 4Hp SB
		7	10.592008371191	-1.92	4C.-.O 8C.-.H 2C.-.C 6H.-.O 7H.-.H 2Hp
		4	12.210188189110	-0.30	
		3	12.381202371234	-0.13	

Continue in the next page

Table SM1: Structures of various conformations are evaluated for their energetic properties and types of intermolecular interactions. In this context, Am stands for amino acid, FM for functional monomer, N° conf. for the spatial conformation number of the Amino acid-FM complex, E_{tot} represents the ground state electronic energy in kcal mol^{-1}, the ΔE represents the difference of the electronic energy in ascending order of energy between the complex and lastly, the type of interaction specifies the atoms that are in close proximity in the table. The symbols Hb denote a hydrogen bond, AmtoM indicates that an AM is complexing with an FM, and MtoAm is the reverse of AmtoM. The symbols SB denote a salt bridges. The symbols Hp denote a hydrophobics interactions. The symbols Cation-pi/pi-staquing/pi-T-sheped denote the type of π interactions interactions.

AA	FM	N° conf.	ΔE	E_{tot}	Type of interaction
		8	12.589340152684	0.08	
	acida	7	0.000000000000	-3.81	8C.-.H 7H.-.H 2C.-.C 5C.-.O 6H.-.O 3O.-.O 2Hp Hb(AmtoM)
		0	1.333190786788	-2.48	6C.-.C 9C.-.H 7H.-.H 6C.-.O 6H.-.O 6Hp SB
		4	1.523630594748	-2.29	2C.-.O 4C.-.H 2H.-.N 4H.-.O 4H.-.H N.-.O O.-.O Hb(AmtoM) SB
		1	1.825817642866	-1.98	9C.-.H 3C.-.N 4C.-.C 8H.-.H 3C.-.O H.-.N 4H.-.O N.-.O 2O.-.O 4Hp SB
		3	1.893945595790	-1.92	4C.-.C 3C.-.N 4C.-.O 8C.-.H 7H.-.O 4H.-.H 2N.-.O H.-.N 4Hp SB
		9	2.261000223316	-1.55	12C.-.H C.-.C 7H.-.H H.-.O Hp
		8	3.561404867708	-0.25	
		2	3.585465244943	-0.22	H.-.H

Continue in the next page

Table SM1: Structures of various conformations are evaluated for their energetic properties and types of intermolecular interactions. In this context, Am stands for amino acid, FM for functional monomer, N° conf. for the spatial conformation number of the Amino acid-FM complex, E_{tot} represents the ground state electronic energy in kcal mol^{-1}, the ΔE represents the difference of the electronic energy in ascending order of energy between the complex and lastly, the type of interaction specifies the atoms that are in close proximity in the table. The symbols Hb denote a hydrogen bond, AmtoM indicates that an AM is complexing with an FM, and MtoAm is the reverse of AmtoM. The symbols SB denote a salt bridges. The symbols Hp denote a hydrophobics interactions. The symbols Cation-pi/pi-staquing/pi-T-sheped denote the type of π interactions interactions.

AA	FM	N° conf.	ΔE	E_{tot}	Type of interaction
	bisac	2	0.000000000000	-9.97	5C.-.O 6C.-.H 6H.-.O 5H.-.H C.-.N N.-.O 2H.-.N 2O.-.O Hb(MtoAm) Hb(AmtoM)
		3	1.732353035604	-8.24	13C.-.H 7H.-.N 2H.-.O C.-.O N.-.O 5C.-.N 12H.-.H N.-.N Hb(MtoAm)
		5	1.734172631251	-8.24	10C.-.H 6H.-.O 10H.-.H C.-.N 2N.-.O 3H.-.N 3C.-.O 2O.-.O Hb(MtoAm) Hb(AmtoM)
		0	2.596223284744	-7.38	5C.-.N C.-.C 14C.-.H 6H.-.N 10H.-.H N.-.N 4H.-.O 2C.-.O N.-.O 2O.-.O Hp Hb(MtoAm)
		8	7.984833617588	-1.99	9C.-.H 3C.-.C 10H.-.H H.-.O H.-.N 3Hp
		4	9.844140442545	-0.13	
		1	10.025243587670	0.05	
		6	10.070525488035	0.10	

Continue in the next page

Table SM1: Structures of various conformations are evaluated for their energetic properties and types of intermolecular interactions. In this context, Am stands for amino acid, FM for functional monomer, N° conf. for the spatial conformation number of the Amino acid-FM complex, E_{tot} represents the ground state electronic energy in kcal mol^{-1}, the ΔE represents the difference of the electronic energy in ascending order of energy between the complex and lastly, the type of interaction specifies the atoms that are in close proximity in the table. The symbols Hb denote a hydrogen bond, AmtoM indicates that an AM is complexing with an FM, and MtoAm is the reverse of AmtoM. The symbols SB denote a salt bridges. The symbols Hp denote a hydrophobics interactions. The symbols Cation-pi/pi-staquing/pi-T-sheped denote the type of π interactions interactions.

AA	FM	N° conf.	ΔE	E_{tot}	Type of interaction
	1ally	9	0.000000000000	-16.91	6C.-.H 10H.-.O 3H.-.H C.-.N 2N.-.O C.-.C 6C.-.O Hp Hb(MtoAm) 2SB
		7	13.770835449395	-3.14	15C.-.H 3C.-.C 16H.-.H H.-.N 5H.-.O 3C.-.O 3Hp SB
		4	14.771483739347	-2.14	2H.-.N 14C.-.H 17H.-.H 2C.-.C 2Hp
		1	16.378476735182	-0.53	
		8	16.424229669351	-0.48	
		0	16.434148963506	-0.47	
		2	16.450166944676	-0.46	
		6	16.560759562844	-0.35	
		3	16.797659294001	-0.11	
		5	16.850805515719	-0.06	2H.-.N 3C.-.H C.-.O 5H.-.H 3H.-.O
	4imid	0	0.000000000000	-9.65	4C.-.O 5C.-.H 3H.-.O 2H.-.H 2H.-.N C.-.C C.-.N 2N.-.O Hp Hb(AmtoM)

Continue in the next page

Table SM1: Structures of various conformations are evaluated for their energetic properties and types of intermolecular interactions. In this context, Am stands for amino acid, FM for functional monomer, N° conf. for the spatial conformation number of the Amino acid-FM complex, E_{tot} represents the ground state electronic energy in kcal mol^{-1}, the ΔE represents the difference of the electronic energy in ascending order of energy between the complex and lastly, the type of interaction specifies the atoms that are in close proximity in the table. The symbols Hb denote a hydrogen bond, AmtoM indicates that an AM is complexing with an FM, and MtoAm is the reverse of AmtoM. The symbols SB denote a salt bridges. The symbols Hp denote a hydrophobics interactions. The symbols Cation-pi/pi-staquing/pi-T-sheped denote the type of π interactions interactions.

AA	FM	N° conf.	ΔE	E_{tot}	Type of interaction
		5	5.643163904359	-4.01	5H.-.O 17C.-.H 8H.-.H 8C.-.C 5C.-.O 2C.-.N 3N.-.O 3H.-.N 8Hp
		1	6.139630454266	-3.51	9C.-.O 7H.-.O 10H.-.H 4C.-.N 20C.-.H 8C.-.C 4N.-.O 3H.-.N 8Hp Hb(AmtoM) SB
		4	7.771434242437	-1.88	8H.-.H 9C.-.H 2C.-.O 2C.-.C 3H.-.N 3C.-.N N.-.O 2H.-.O 2Hp Hb(MtoAm)
		3	8.944762513292	-0.70	C.-.C 5C.-.H H.-.N 6H.-.H Hp
		9	9.329614902360	-0.32	
		7	9.365026871891	-0.28	
		6	9.549208331799	-0.10	
	acril	7	0.000000000000	-4.60	3C.-.N 6C.-.H 4H.-.N N.-.N 2N.-.O 2H.-.O 6H.-.H Hb(MtoAm)
		9	2.188791345351	-2.41	11C.-.H 8H.-.H 2C.-.C 4H.-.N 2C.-.N 4H.-.O 3C.-.O 2Hp

Continue in the next page

Table SM1: Structures of various conformations are evaluated for their energetic properties and types of intermolecular interactions. In this context, Am stands for amino acid, FM for functional monomer, Nº conf. for the spatial conformation number of the Amino acid-FM complex, E_{tot} represents the ground state electronic energy in kcal mol^{-1}, the ΔE represents the difference of the electronic energy in ascending order of energy between the complex and lastly, the type of interaction specifies the atoms that are in close proximity in the table. The symbols Hb denote a hydrogen bond, AmtoM indicates that an AM is complexing with an FM, and MtoAm is the reverse of AmtoM. The symbols SB denote a salt bridges. The symbols Hp denote a hydrophobics interactions. The symbols Cation-pi/pi-staquing/pi-T-sheped denote the type of π interactions interactions.

AA	FM	Nº conf.	ΔE	E_{tot}	Type of interaction
		3	2.393287521591	-2.21	C.-.N 5C.-.C 11C.-.H 2C.-.O 3H.-.N 8H.-.H 2H.-.O 5Hp
		8	2.965811509064	-1.63	8C.-.H C.-.C C.-.N 6H.-.H 2H.-.N Hp
		1	3.133995867531	-1.47	9C.-.H C.-.C C.-.O 7H.-.H 2H.-.O Hp
		5	3.267607453579	-1.33	13C.-.H 12H.-.H 2C.-.C 4H.-.N 2Hp
		0	4.301862585389	-0.30	
	alila	0	0.000000000000	-2.00	15C.-.H 13H.-.H 3C.-.C 5H.-.N 3Hp
		2	0.123531170511	-1.87	9C.-.H 10H.-.H C.-.C 2C.-.O 4H.-.O 2C.-.N 2H.-.N 2N.-.O Hp SB
		4	0.791890364853	-1.21	C.-.O N.-.O H.-.N 5H.-.O C.-.H 2H.-.H Hb(MtoAm) SB
		1	0.816274933876	-1.18	5H.-.N 2C.-.N C.-.C 5C.-.H 7H.-.H Hp

Continue in the next page

Table SM1: Structures of various conformations are evaluated for their energetic properties and types of intermolecular interactions. In this context, Am stands for amino acid, FM for functional monomer, N° conf. for the spatial conformation number of the Amino acid-FM complex, E_{tot} represents the ground state electronic energy in kcal mol^{-1}, the ΔE represents the difference of the electronic energy in ascending order of energy between the complex and lastly, the type of interaction specifies the atoms that are in close proximity in the table. The symbols Hb denote a hydrogen bond, AmtoM indicates that an AM is complexing with an FM, and MtoAm is the reverse of AmtoM. The symbols SB denote a salt bridges. The symbols Hp denote a hydrophobics interactions. The symbols Cation-pi/pi-staquing/pi-T-sheped denote the type of π interactions interactions.

AA	FM	N° conf.	ΔE	E_{tot}	Type of interaction
		5	1.716834067478	-0.28	
		8	1.766031132917	-0.23	
		3	1.800476435697	-0.20	
		9	1.890114960043	-0.11	
	estir	9	0.000000000000	-4.17	20C.-.H 6C.-.C 8H.-.H C.-.O H.-.O 6Hp Cation-pi(Amc)
		1	0.941613613426	-3.23	14C.-.H 4C.-.N 5C.-.O 2H.-.N 7H.-.H 5C.-.C 4H.-.O 5Hp
		4	2.039850853492	-2.13	10C.-.H C.-.C C.-.O 2H.-.N 8H.-.H H.-.O Hp
		0	3.200799438663	-0.97	3C.-.H C.-.O 4H.-.H 2H.-.O
		5	3.309601979760	-0.86	2H.-.H
		3	3.796670542831	-0.37	
		8	3.799595349711	-0.37	
		6	3.818756850978	-0.35	

Continue in the next page

Table SM1: Structures of various conformations are evaluated for their energetic properties and types of intermolecular interactions. In this context, Am stands for amino acid, FM for functional monomer, Nº conf. for the spatial conformation number of the Amino acid-FM complex, E_{tot} represents the ground state electronic energy in kcal mol^{-1}, the ΔE represents the difference of the electronic energy in ascending order of energy between the complex and lastly, the type of interaction specifies the atoms that are in close proximity in the table. The symbols Hb denote a hydrogen bond, AmtoM indicates that an AM is complexing with an FM, and MtoAm is the reverse of AmtoM. The symbols SB denote a salt bridges. The symbols Hp denote a hydrophobics interactions. The symbols Cation-pi/pi-staquing/pi-T-sheped denote the type of π interactions interactions.

AA	FM	Nº conf.	ΔE	E_{tot}	Type of interaction
	1viny	4	0.000000000000	-4.58	5C.-.N 17C.-.H 8C.-.C N.-.O 8H.-.N 4C.-.O N.-.N 12H.-.H 3H.-.O 8Hp Hb(AmtoM) Cation-pi(Amc)
		3	1.151041397637	-3.43	17C.-.H 6H.-.N 6C.-.C 2C.-.N 12H.-.H 6Hp
		9	1.371453927659	-3.21	5C.-.O 8C.-.H C.-.N 3N.-.O 3H.-.N C.-.C 4H.-.O 6H.-.H Hp
		5	2.765762697081	-1.81	C.-.C 4C.-.O 4C.-.H 2N.-.O 2H.-.N 3H.-.O 2H.-.H Hp
		6	2.967153269618	-1.61	2C.-.N 4C.-.H C.-.O 2H.-.N 3H.-.H 2H.-.O
		8	4.300314472018	-0.28	
		2	4.497530773845	-0.08	
		0	4.649449634742	0.07	
	2hydr	0	0.000000000000	-9.04	2C.-.N 3C.-.O 9C.-.H 3H.-.N 6H.-.O 7H.-.H N.-.O Hb(MtoAm)

Continue in the next page

Table SM1: Structures of various conformations are evaluated for their energetic properties and types of intermolecular interactions. In this context, Am stands for amino acid, FM for functional monomer, N° conf. for the spatial conformation number of the Amino acid-FM complex, E_{tot} represents the ground state electronic energy in $\mathrm{kcal\,mol^{-1}}$, the ΔE represents the difference of the electronic energy in ascending order of energy between the complex and lastly, the type of interaction specifies the atoms that are in close proximity in the table. The symbols Hb denote a hydrogen bond, AmtoM indicates that an AM is complexing with an FM, and MtoAm is the reverse of AmtoM. The symbols SB denote a salt bridges. The symbols Hp denote a hydrophobics interactions. The symbols Cation-pi/pi-staquing/pi-T-sheped denote the type of π interactions interactions.

AA	FM	N° conf.	ΔE	E_{tot}	Type of interaction
		2	4.542744216723	-4.49	20C.-.H 4C.-.N 6C.-.C 11H.-.H 3H.-.N 2N.-.O 10H.-.O 6C.-.O 3O.-.O 6Hp Hb(MtoAm)
		9	5.292332867720	-3.75	2C.-.N 22C.-.H 4C.-.C 17H.-.H 4C.-.O H.-.N 6H.-.O 4Hp
		7	6.893771688046	-2.14	2C.-.N 2C.-.O 7C.-.H H.-.N 4H.-.O 3H.-.H N.-.O
		6	8.776597198138	-0.26	
		3	8.871772162245	-0.17	
		1	8.920320211976	-0.12	3C.-.H 3H.-.O C.-.O 3H.-.H
	4viny	0	0.000000000000	-3.89	3C.-.N 4C.-.C 12C.-.H N.-.N 5H.-.N N.-.O 8H.-.H H.-.O 4Hp Hb(AmtoM)
		8	0.187902722807	-3.70	16C.-.H 8C.-.C 4C.-.O 11H.-.H C.-.N 2H.-.N 2H.-.O 8Hp
		9	2.549887823316	-1.34	2C.-.N 3C.-.C 12C.-.H 3H.-.N 6H.-.H 3Hp Cation-pi(Amc)

Continue in the next page

Table SM1: Structures of various conformations are evaluated for their energetic properties and types of intermolecular interactions. In this context, Am stands for amino acid, FM for functional monomer, N° conf. for the spatial conformation number of the Amino acid-FM complex, E_{tot} represents the ground state electronic energy in kcal mol^{-1}, the ΔE represents the difference of the electronic energy in ascending order of energy between the complex and lastly, the type of interaction specifies the atoms that are in close proximity in the table. The symbols Hb denote a hydrogen bond, AmtoM indicates that an AM is complexing with an FM, and MtoAm is the reverse of AmtoM. The symbols SB denote a salt bridges. The symbols Hp denote a hydrophobics interactions. The symbols Cation-pi/pi-staquing/pi-T-sheped denote the type of π interactions interactions.

AA	FM	N° conf.	ΔE	E_{tot}	Type of interaction
		3	2.855848942780	-1.03	2C.-.N 7C.-.H 7H.-.H C.-.O 2H.-.N H.-.O
		6	3.299574331928	-0.59	H.-.H
		4	3.504553858495	-0.38	
		7	3.520586026938	-0.37	
		5	3.575396871568	-0.31	
	acrol	1	0.000000000000	-5.44	3C.-.O 4C.-.H 3H.-.O 2H.-.H 2O.-.O Hb(AmtoM)
		2	1.642399474633	-3.80	3C.-.N 5C.-.C 14C.-.H 8H.-.H 2H.-.N 6H.-.O 2C.-.O N.-.O 5Hp
		9	2.827197130389	-2.62	3C.-.C 12C.-.H 8H.-.H 4C.-.O 5H.-.O O.-.O C.-.N 2H.-.N 3Hp
		5	2.946561773300	-2.50	12C.-.H 3C.-.C 7H.-.H 3H.-.O 2C.-.O 3Hp
		8	5.119511857049	-0.32	
		0	5.182777230792	-0.26	
		7	5.189853801568	-0.25	
		3	5.423408042269	-0.02	

Continue in the next page

Table SM1: Structures of various conformations are evaluated for their energetic properties and types of intermolecular interactions. In this context, Am stands for amino acid, FM for functional monomer, N° conf. for the spatial conformation number of the Amino acid-FM complex, E_{tot} represents the ground state electronic energy in kcal mol^{-1}, the ΔE represents the difference of the electronic energy in ascending order of energy between the complex and lastly, the type of interaction specifies the atoms that are in close proximity in the table. The symbols Hb denote a hydrogen bond, AmtoM indicates that an AM is complexing with an FM, and MtoAm is the reverse of AmtoM. The symbols SB denote a salt bridges. The symbols Hp denote a hydrophobics interactions. The symbols Cation-pi/pi-staquing/pi-T-sheped denote the type of π interactions interactions.

AA	FM	N° conf.	ΔE	E_{tot}	Type of interaction
	itaco	9	0.000000000000	-16.78	7H.-.O 3C.-.O 2N.-.O C.-.N 4C.-.H H.-.H Hb(AmtoM) SB
		1	10.042571963860	-6.74	10C.-.O 5O.-.O 11H.-.O 2C.-.C 14C.-.H 7H.-.H 2H.-.N N.-.O 2Hp Hb(AmtoM) Hb(MtoAm) 2SB
		8	13.303640212145	-3.48	2C.-.O 3O.-.O 4H.-.O 2C.-.H 2H.-.H Hb(MtoAm) Hb(AmtoM)
		0	13.884263311654	-2.90	6H.-.O 4C.-.O 13H.-.H 17C.-.H 8C.-.C 8Hp 2SB
SER	14dvb	7	0.000000000000	-3.90	12C.-.H 8H.-.H 6C.-.C 3C.-.N 2H.-.N 3C.-.O 2H.-.O 6Hp Cation-pi(Amc)
		0	0.794957795963	-3.10	12C.-.H 5C.-.N 8H.-.H 2H.-.N C.-.C H.-.O Hp Cation-pi(Amc)
		5	1.570631871152	-2.33	2C.-.O 9H.-.H 10C.-.H 3H.-.O 4C.-.C H.-.N 4Hp
		2	1.603937695207	-2.29	3C.-.O 5C.-.H 4H.-.O 5H.-.H

Continue in the next page

Table SM1: Structures of various conformations are evaluated for their energetic properties and types of intermolecular interactions. In this context, Am stands for amino acid, FM for functional monomer, N° conf. for the spatial conformation number of the Amino acid-FM complex, E_{tot} represents the ground state electronic energy in kcal mol^{-1}, the ΔE represents the difference of the electronic energy in ascending order of energy between the complex and lastly, the type of interaction specifies the atoms that are in close proximity in the table. The symbols Hb denote a hydrogen bond, AmtoM indicates that an AM is complexing with an FM, and MtoAm is the reverse of AmtoM. The symbols SB denote a salt bridges. The symbols Hp denote a hydrophobics interactions. The symbols Cation-pi/pi-staquing/pi-T-sheped denote the type of π interactions interactions.

AA	FM	N° conf.	ΔE	E_{tot}	Type of interaction
		6	1.988232725612	-1.91	6C.-.H 2C.-.O 4H.-.O 5H.-.H
		8	3.739114858720	-0.16	
		4	3.776873539617	-0.12	
		9	3.843124133334	-0.05	
		3	3.947114001171	0.05	
	2viny	0	0.000000000000	-3.07	4C.-.C 5C.-.O 14C.-.H C.-.N N.-.O 2H.-.N 9H.-.H 3H.-.O 4Hp Cation-pi(Amc)
		6	0.181149334549	-2.89	14C.-.H 4C.-.C 3C.-.O 4H.-.N 3C.-.N N.-.N 8H.-.H 2H.-.O 4Hp Cation-pi(Amc)
		5	0.964066128307	-2.10	3C.-.N 9C.-.H 3C.-.C 2H.-.N C.-.O 8H.-.H 2H.-.O 3Hp
		8	2.151183209074	-0.92	2C.-.N 5C.-.H 2H.-.O C.-.O 2H.-.N 5H.-.H
		4	2.316703226427	-0.75	2C.-.H C.-.N 2H.-.N 3H.-.H
		9	2.891733027395	-0.18	
		7	3.027025238605	-0.04	2H.-.H

Continue in the next page

Table SM1: Structures of various conformations are evaluated for their energetic properties and types of intermolecular interactions. In this context, Am stands for amino acid, FM for functional monomer, N° conf. for the spatial conformation number of the Amino acid-FM complex, E_{tot} represents the ground state electronic energy in $\mathrm{kcal\,mol^{-1}}$, the ΔE represents the difference of the electronic energy in ascending order of energy between the complex and lastly, the type of interaction specifies the atoms that are in close proximity in the table. The symbols Hb denote a hydrogen bond, AmtoM indicates that an AM is complexing with an FM, and MtoAm is the reverse of AmtoM. The symbols SB denote a salt bridges. The symbols Hp denote a hydrophobics interactions. The symbols Cation-pi/pi-staquing/pi-T-sheped denote the type of π interactions interactions.

AA	FM	N° conf.	ΔE	E_{tot}	Type of interaction
		1	3.069693811410	0.00	
		2	77.417130378239	74.35	erro
	acidm	2	0.000000000000	-6.16	9H.-.O 6H.-.H 4C.-.O 5C.-.H 4O.-.O Hb(MtoAm) SB
		3	3.583115538242	-2.58	6C.-.O 10C.-.H 6H.-.O 6H.-.H 4C.-.C 2O.-.O 4Hp SB
		9	4.284108992166	-1.88	4C.-.H 2C.-.N 3H.-.O 2C.-.O N.-.O H.-.N 3H.-.H Hb(AmtoM) SB
		5	4.710889847533	-1.45	10C.-.H 8H.-.H 4C.-.C C.-.N H.-.N C.-.O 5H.-.O O.-.O 4Hp SB
		7	5.035847324186	-1.13	8C.-.H 2C.-.O 6H.-.O 7H.-.H O.-.O SB
		0	5.776749710122	-0.38	C.-.C C.-.N 3C.-.O 5C.-.H 7H.-.H H.-.N 5H.-.O Hp
		1	6.115355395342	-0.05	
		8	6.251657833851	0.09	

Continue in the next page

Table SM1: Structures of various conformations are evaluated for their energetic properties and types of intermolecular interactions. In this context, Am stands for amino acid, FM for functional monomer, N° conf. for the spatial conformation number of the Amino acid-FM complex, E_{tot} represents the ground state electronic energy in kcal mol^{-1}, the ΔE represents the difference of the electronic energy in ascending order of energy between the complex and lastly, the type of interaction specifies the atoms that are in close proximity in the table. The symbols Hb denote a hydrogen bond, AmtoM indicates that an AM is complexing with an FM, and MtoAm is the reverse of AmtoM. The symbols SB denote a salt bridges. The symbols Hp denote a hydrophobics interactions. The symbols Cation-pi/pi-staquing/pi-T-sheped denote the type of π interactions interactions.

AA	FM	N° conf.	ΔE	E_{tot}	Type of interaction
	acida	0	0.000000000000	-10.12	6C.-.H 2C.-.C C.-.N 8H.-.O 4C.-.O 2N.-.O 5H.-.H H.-.N 2Hp Hb(MtoAm) SB
		9	0.379742802254	-9.74	7C.-.H 2C.-.C C.-.N 7H.-.O 4C.-.O 2N.-.O 5H.-.H H.-.N 2Hp Hb(MtoAm) SB
		8	0.631698624951	-9.48	C.-.N 6C.-.H 6H.-.O 2C.-.O 2N.-.O 5H.-.H H.-.N Hb(MtoAm) SB
		5	6.628099015674	-3.49	6C.-.O 7C.-.H C.-.C 6H.-.O 5H.-.H 2O.-.O Hp Hb(AmtoM)
		4	8.076473779432	-2.04	6C.-.H 2C.-.O 4H.-.H 2H.-.N 4H.-.O N.-.O Hb(AmtoM) SB
		7	8.337460944903	-1.78	6C.-.H 3C.-.O 5H.-.H 2H.-.N 4H.-.O N.-.O O.-.O Hb(AmtoM) SB

Continue in the next page

Table SM1: Structures of various conformations are evaluated for their energetic properties and types of intermolecular interactions. In this context, Am stands for amino acid, FM for functional monomer, N° conf. for the spatial conformation number of the Amino acid-FM complex, E_{tot} represents the ground state electronic energy in kcal mol^{-1}, the ΔE represents the difference of the electronic energy in ascending order of energy between the complex and lastly, the type of interaction specifies the atoms that are in close proximity in the table. The symbols Hb denote a hydrogen bond, AmtoM indicates that an AM is complexing with an FM, and MtoAm is the reverse of AmtoM. The symbols SB denote a salt bridges. The symbols Hp denote a hydrophobics interactions. The symbols Cation-pi/pi-staquing/pi-T-sheped denote the type of π interactions interactions.

AA	FM	N° conf.	ΔE	E_{tot}	Type of interaction
		6	8.382337943428	-1.73	2C.-.N 9C.-.H 2H.-.N 6H.-.H C.-.C 2C.-.O 4H.-.O O.-.O Hp SB
		1	9.101722940200	-1.01	7C.-.H 2C.-.C 2C.-.O 7H.-.H H.-.N 2H.-.O 2Hp
		3	9.816834671028	-0.30	C.-.N 2C.-.H H.-.N 4H.-.H
	bisac	9	0.000000000000	-9.97	10C.-.H 9H.-.H 6C.-.O 2O.-.O 8H.-.O 3N.-.O 3H.-.N C.-.C C.-.N Hp Hb(AmtoM) Hb(MtoAm)
		5	0.178538361360	-9.79	9C.-.H 7H.-.O 6H.-.H 5C.-.O 2O.-.O 2H.-.N C.-.N N.-.O Hb(AmtoM) Hb(MtoAm)
		4	2.735576588359	-7.23	5H.-.H 4H.-.O 2N.-.O 2H.-.N 3C.-.O 6C.-.H 2O.-.O Hb(MtoAm) Hb(AmtoM)
		4	2.735576588359	-7.23	erro

Continue in the next page

Table SM1: Structures of various conformations are evaluated for their energetic properties and types of intermolecular interactions. In this context, Am stands for amino acid, FM for functional monomer, N° conf. for the spatial conformation number of the Amino acid-FM complex, E_{tot} represents the ground state electronic energy in kcal mol^{-1}, the ΔE represents the difference of the electronic energy in ascending order of energy between the complex and lastly, the type of interaction specifies the atoms that are in close proximity in the table. The symbols Hb denote a hydrogen bond, AmtoM indicates that an AM is complexing with an FM, and MtoAm is the reverse of AmtoM. The symbols SB denote a salt bridges. The symbols Hp denote a hydrophobics interactions. The symbols Cation-pi/pi-staquing/pi-T-sheped denote the type of π interactions interactions.

AA	FM	N° conf.	ΔE	E_{tot}	Type of interaction
		8	2.864144386149	-7.11	9H.-.H 10C.-.H 5C.-.O 3H.-.N O.-.O 6H.-.O C.-.N N.-.O Hb(AmtoM) Hb(MtoAm)
		3	5.917690565243	-4.05	6H.-.H 7C.-.H 5H.-.N 7H.-.O 5C.-.O 2N.-.O 2O.-.O 2C.-.N Hb(AmtoM) Hb(MtoAm)
		2	7.846897082234	-2.12	2C.-.H 3H.-.O C.-.O N.-.O 4H.-.N 4H.-.H Hb(AmtoM)
		7	8.649946439645	-1.32	8C.-.H 2H.-.O C.-.C 4H.-.H Hp
		1	9.816564061484	-0.15	3C.-.H 4H.-.H N.-.O H.-.N 2C.-.O 3H.-.O O.-.O
		6	9.968362479676	-0.00	
	1ally	9	0.000000000000	-14.44	3H.-.H 5C.-.H 11H.-.O C.-.N 2N.-.O 2C.-.C 7C.-.O 2Hp Hb(MtoAm) 2SB
		0	4.756776435030	-9.68	C.-.N N.-.O 4H.-.N 4C.-.O 11C.-.H 9H.-.O 15H.-.H Hb(AmtoM) 2SB

Continue in the next page

Table SM1: Structures of various conformations are evaluated for their energetic properties and types of intermolecular interactions. In this context, Am stands for amino acid, FM for functional monomer, N° conf. for the spatial conformation number of the Amino acid-FM complex, E_{tot} represents the ground state electronic energy in kcal mol^{-1}, the ΔE represents the difference of the electronic energy in ascending order of energy between the complex and lastly, the type of interaction specifies the atoms that are in close proximity in the table. The symbols Hb denote a hydrogen bond, AmtoM indicates that an AM is complexing with an FM, and MtoAm is the reverse of AmtoM. The symbols SB denote a salt bridges. The symbols Hp denote a hydrophobics interactions. The symbols Cation-pi/pi-staquing/pi-T-sheped denote the type of π interactions interactions.

AA	FM	N° conf.	ΔE	E_{tot}	Type of interaction
		2	6.456523103255	-7.98	4H.-.N 2C.-.C 11C.-.O 22C.-.H 21H.-.H 12H.-.O C.-.N N.-.O 2Hp Hb(AmtoM) 2SB
		1	13.844864348439	-0.59	
		7	13.901421181382	-0.54	2C.-.N 5C.-.H 3H.-.N 6H.-.H
		6	14.217157569900	-0.22	
		4	14.298713236316	-0.14	
	4imid	9	0.000000000000	-8.49	4C.-.O 3O.-.O 7H.-.O 5C.-.H 4H.-.H Hb(MtoAm) Hb(AmtoM) SB
		7	3.996813930767	-4.50	4H.-.O 2C.-.O 6C.-.H 4H.-.H O.-.O Hb(AmtoM)
		7	3.996813930767	-4.50	erro
		0	4.790852931615	-3.70	7C.-.H 5H.-.N 4H.-.H 3C.-.N N.-.N N.-.O 2C.-.C C.-.O H.-.O 2Hp Hb(AmtoM)

Continue in the next page

Table SM1: Structures of various conformations are evaluated for their energetic properties and types of intermolecular interactions. In this context, Am stands for amino acid, FM for functional monomer, N° conf. for the spatial conformation number of the Amino acid-FM complex, E_{tot} represents the ground state electronic energy in kcal mol^{-1}, the ΔE represents the difference of the electronic energy in ascending order of energy between the complex and lastly, the type of interaction specifies the atoms that are in close proximity in the table. The symbols Hb denote a hydrogen bond, AmtoM indicates that an AM is complexing with an FM, and MtoAm is the reverse of AmtoM. The symbols SB denote a salt bridges. The symbols Hp denote a hydrophobics interactions. The symbols Cation-pi/pi-staquing/pi-T-sheped denote the type of π interactions interactions.

AA	FM	N° conf.	ΔE	E_{tot}	Type of interaction
		2	5.493699534567	-3.00	7H.-.O 2O.-.O 8C.-.H 4C.-.C C.-.N 5C.-.O 6H.-.H N.-.O H.-.N 4Hp Hb(AmtoM) SB
		1	6.342808385324	-2.15	3H.-.O 11C.-.H 3C.-.N 6H.-.H 4C.-.C 3N.-.O 4C.-.O 4H.-.N 4Hp Hb(AmtoM) SB
		4	7.187032271171	-1.31	6H.-.O 2C.-.O N.-.O 2O.-.O 6H.-.H 7C.-.H 2C.-.N H.-.N SB
	acril	9	0.000000000000	-5.30	6C.-.H 3C.-.O 3H.-.H 4H.-.O O.-.O H.-.N Hb(AmtoM)
		3	1.086937138181	-4.21	5H.-.O 9H.-.H 6C.-.H 3C.-.O 3H.-.N C.-.N N.-.O Hb(MtoAm)
		8	1.424975973744	-3.87	11C.-.H 2C.-.C 2C.-.O 10H.-.H 6H.-.O 3H.-.N 2C.-.N 2N.-.O 2Hp Hb(MtoAm)
		2	1.538244425040	-3.76	6C.-.H 2C.-.O 8H.-.H 3H.-.O 3H.-.N C.-.N N.-.O Hb(MtoAm)

Continue in the next page

Table SM1: Structures of various conformations are evaluated for their energetic properties and types of intermolecular interactions. In this context, Am stands for amino acid, FM for functional monomer, N° conf. for the spatial conformation number of the Amino acid-FM complex, E_{tot} represents the ground state electronic energy in kcal mol^{-1}, the ΔE represents the difference of the electronic energy in ascending order of energy between the complex and lastly, the type of interaction specifies the atoms that are in close proximity in the table. The symbols Hb denote a hydrogen bond, AmtoM indicates that an AM is complexing with an FM, and MtoAm is the reverse of AmtoM. The symbols SB denote a salt bridges. The symbols Hp denote a hydrophobics interactions. The symbols Cation-pi/pi-staquing/pi-T-sheped denote the type of π interactions interactions.

AA	FM	N° conf.	ΔE	E_{tot}	Type of interaction
		7	2.133178797134	-3.17	7C.-.H C.-.C 2C.-.O 5H.-.O 4H.-.H H.-.N N.-.O Hp Hb(AmtoM)
		1	3.912266031904	-1.39	2C.-.N 8C.-.H C.-.C 6H.-.N 9H.-.H 3H.-.O N.-.O Hp Hb(MtoAm)
		0	5.320931893673	0.02	
	alila	9	0.000000000000	-1.92	2C.-.O 5H.-.O 9H.-.H 8C.-.H C.-.C 2H.-.N 2C.-.N 2N.-.O Hp Hb(MtoAm) SB
		4	0.009229560312	-1.91	erro
		8	0.138856250209	-1.78	10C.-.H C.-.O 12H.-.H 2H.-.O C.-.C 2C.-.N 5H.-.N N.-.N Hp SB
		2	1.838002741041	-0.08	
		3	1.884795266950	-0.03	
		1	1.903839713086	-0.01	
		7	1.911975529250	-0.00	

Continue in the next page

Table SM1: Structures of various conformations are evaluated for their energetic properties and types of intermolecular interactions. In this context, Am stands for amino acid, FM for functional monomer, N° conf. for the spatial conformation number of the Amino acid-FM complex, E_{tot} represents the ground state electronic energy in kcal mol^{-1}, the ΔE represents the difference of the electronic energy in ascending order of energy between the complex and lastly, the type of interaction specifies the atoms that are in close proximity in the table. The symbols Hb denote a hydrogen bond, AmtoM indicates that an AM is complexing with an FM, and MtoAm is the reverse of AmtoM. The symbols SB denote a salt bridges. The symbols Hp denote a hydrophobics interactions. The symbols Cation-pi/pi-staquing/pi-T-sheped denote the type of π interactions interactions.

AA	FM	N° conf.	ΔE	E_{tot}	Type of interaction
		6	1.968456533180	0.05	
	estir	8	0.000000000000	-4.78	12C.-.O 19C.-.H 6C.-.C 13H.-.H 7H.-.O 6Hp
		5	0.997488668601	-3.78	6C.-.N 4C.-.C 17C.-.H 3C.-.O 10H.-.H 3H.-.O 4Hp Cation-pi(Amc)
		9	1.247399261342	-3.53	6C.-.C 7C.-.O 21C.-.H 4H.-.O 14H.-.H C.-.N H.-.N 6Hp
		0	1.336584487275	-3.45	6C.-.N 16C.-.H 3C.-.C 2C.-.O 10H.-.H 2H.-.O H.-.N 3Hp Cation-pi(Amc)
		3	2.397679437912	-2.38	8C.-.O 6C.-.H 6H.-.O 5H.-.H 2C.-.C 2Hp
		6	2.923404121834	-1.86	2C.-.O 9C.-.H 3H.-.O 9H.-.H C.-.C H.-.N Hp
		4	3.598016880055	-1.18	2C.-.C 10C.-.H 2H.-.N 5H.-.H 2C.-.O 2H.-.O 2Hp
		1	4.704660098530	-0.08	

Continue in the next page

Table SM1: Structures of various conformations are evaluated for their energetic properties and types of intermolecular interactions. In this context, Am stands for amino acid, FM for functional monomer, N° conf. for the spatial conformation number of the Amino acid-FM complex, E_{tot} represents the ground state electronic energy in kcal mol^{-1}, the ΔE represents the difference of the electronic energy in ascending order of energy between the complex and lastly, the type of interaction specifies the atoms that are in close proximity in the table. The symbols Hb denote a hydrogen bond, AmtoM indicates that an AM is complexing with an FM, and MtoAm is the reverse of AmtoM. The symbols SB denote a salt bridges. The symbols Hp denote a hydrophobics interactions. The symbols Cation-pi/pi-staquing/pi-T-sheped denote the type of π interactions interactions.

AA	FM	N° conf.	ΔE	E_{tot}	Type of interaction
		2	4.790054105555	0.01	
		7	5.319610943260	0.54	
	1viny	3	0.000000000000	-3.18	9C.-.O 13C.-.H 3N.-.O 2H.-.N 5C.-.C C.-.N 8H.-.H 6H.-.O 5Hp
		6	0.291251430725	-2.88	12C.-.H 10H.-.H 4H.-.N 3C.-.C 3C.-.O C.-.N N.-.O 3H.-.O 3Hp Cation-pi(Amc)
		4	0.402490824760	-2.77	12C.-.H 4C.-.O 2N.-.O H.-.N 8H.-.H 5H.-.O C.-.C Hp
		1	1.009768239949	-2.17	8C.-.H 2C.-.O H.-.N 5H.-.H 4H.-.O
		8	1.208099716373	-1.97	2C.-.N 5C.-.H C.-.O N.-.N 5H.-.N 5H.-.H H.-.O Cation-pi(Amc)
		5	1.348740314883	-1.83	9C.-.H C.-.N 3H.-.N 8H.-.H C.-.C H.-.O Hp
		2	1.824546875299	-1.35	8C.-.H 2C.-.C C.-.N 4H.-.N 4H.-.H 2H.-.O 2Hp

Continue in the next page

Table SM1: Structures of various conformations are evaluated for their energetic properties and types of intermolecular interactions. In this context, Am stands for amino acid, FM for functional monomer, N° conf. for the spatial conformation number of the Amino acid-FM complex, E_{tot} represents the ground state electronic energy in kcal mol^{-1}, the ΔE represents the difference of the electronic energy in ascending order of energy between the complex and lastly, the type of interaction specifies the atoms that are in close proximity in the table. The symbols Hb denote a hydrogen bond, AmtoM indicates that an AM is complexing with an FM, and MtoAm is the reverse of AmtoM. The symbols SB denote a salt bridges. The symbols Hp denote a hydrophobics interactions. The symbols Cation-pi/pi-staquing/pi-T-sheped denote the type of π interactions interactions.

AA	FM	N° conf.	ΔE	E_{tot}	Type of interaction
		7	2.876382099748	-0.30	H.-.H
		9	3.092397472718	-0.08	
		0	3.196066074584	0.02	
	2hydr	2	0.000000000000	-4.94	8C.-.H 5C.-.O 8H.-.H 7H.-.O N.-.O O.-.O H.-.N Hb(AmtoM) Hb(MtoAm)
		4	0.189875214384	-4.75	3H.-.H 4C.-.O 4C.-.H 5H.-.O 3O.-.O Hb(AmtoM)
		9	1.952375160535	-2.99	3C.-.C 8C.-.O 10C.-.H 9H.-.O 7H.-.H C.-.N 3O.-.O N.-.O H.-.N 3Hp Hb(AmtoM)
		6	2.153019024424	-2.78	9C.-.H C.-.C 4C.-.O 13H.-.H 7H.-.O O.-.O Hp Hb(AmtoM)
		8	2.865093442938	-2.07	16C.-.H 3C.-.C 3C.-.N 2C.-.O 7H.-.O N.-.O 18H.-.H 4H.-.N 3Hp
		5	2.886399553587	-2.05	6H.-.H C.-.H 3H.-.O C.-.O N.-.O 2H.-.N Hb(AmtoM)

Continue in the next page

Table SM1: Structures of various conformations are evaluated for their energetic properties and types of intermolecular interactions. In this context, Am stands for amino acid, FM for functional monomer, Nº conf. for the spatial conformation number of the Amino acid-FM complex, E_{tot} represents the ground state electronic energy in kcal mol^{-1}, the ΔE represents the difference of the electronic energy in ascending order of energy between the complex and lastly, the type of interaction specifies the atoms that are in close proximity in the table. The symbols Hb denote a hydrogen bond, AmtoM indicates that an AM is complexing with an FM, and MtoAm is the reverse of AmtoM. The symbols SB denote a salt bridges. The symbols Hp denote a hydrophobics interactions. The symbols Cation-pi/pi-staquing/pi-T-sheped denote the type of π interactions interactions.

AA	FM	Nº conf.	ΔE	E_{tot}	Type of interaction
		0	4.965064334458	0.03	
		7	5.286141645303	0.35	
	4viny	4	0.000000000000	-9.98	C.-.C 3C.-.O 5C.-.H 3H.-.O 2H.-.H C.-.N 2N.-.O H.-.N Hp Hb(AmtoM)
		6	7.055317210507	-2.93	5C.-.C 9C.-.O 11C.-.H 4H.-.O 8H.-.H N.-.O 3H.-.N 2C.-.N 5Hp
		3	7.416209123639	-2.56	16C.-.H 5C.-.C 2C.-.N 10H.-.H 4H.-.N 2C.-.O 3H.-.O 5Hp Cation-pi(Amc)
		5	8.095895129292	-1.88	2C.-.O 7C.-.H 6H.-.O 7H.-.H
		8	8.259038150383	-1.72	11C.-.H 4C.-.O 3C.-.C 4H.-.O 8H.-.H 3Hp
		9	9.843487608275	-0.14	
		0	9.949762099794	-0.03	H.-.O
		1	10.006673634280	0.03	
	acrol	2	0.000000000000	-5.56	3C.-.H C.-.C 3C.-.O 3H.-.O H.-.H 2O.-.O Hp Hb(AmtoM)

Continue in the next page

Table SM1: Structures of various conformations are evaluated for their energetic properties and types of intermolecular interactions. In this context, Am stands for amino acid, FM for functional monomer, N° conf. for the spatial conformation number of the Amino acid-FM complex, E_{tot} represents the ground state electronic energy in kcal mol^{-1}, the ΔE represents the difference of the electronic energy in ascending order of energy between the complex and lastly, the type of interaction specifies the atoms that are in close proximity in the table. The symbols Hb denote a hydrogen bond, AmtoM indicates that an AM is complexing with an FM, and MtoAm is the reverse of AmtoM. The symbols SB denote a salt bridges. The symbols Hp denote a hydrophobics interactions. The symbols Cation-pi/pi-staquing/pi-T-sheped denote the type of π interactions interactions.

AA	FM	N° conf.	ΔE	E_{tot}	Type of interaction
		7	3.580669493745	-1.98	C.-.N C.-.H 3H.-.O 2C.-.O N.-.O H.-.N 2H.-.H Hb(AmtoM)
		8	3.954621952584	-1.61	9C.-.H 7C.-.O 7H.-.H 3C.-.C 6H.-.O 2O.-.O 3Hp
		1	3.968873357098	-1.59	8C.-.H 3C.-.C 2C.-.N 7H.-.H 2H.-.N 2H.-.O C.-.O 3Hp
		9	4.167820904260	-1.39	3C.-.H H.-.N 3H.-.H C.-.N 3H.-.O C.-.O N.-.O Hb(AmtoM)
		4	4.679775525354	-0.88	10C.-.H 2C.-.O 5H.-.H 3H.-.O H.-.N
		6	5.047931067483	-0.51	5C.-.H 3H.-.O 3H.-.H C.-.O
		5	5.150620862143	-0.41	H.-.H C.-.H 2H.-.O C.-.O
		3	5.515130303141	-0.05	
		0	5.519292557330	-0.04	
	itaco	9	0.000000000000	-6.40	7C.-.O C.-.N 7C.-.H 7H.-.O 2H.-.N 8H.-.H N.-.O O.-.O Hb(AmtoM) Hb(MtoAm) SB

Continue in the next page

Table SM1: Structures of various conformations are evaluated for their energetic properties and types of intermolecular interactions. In this context, Am stands for amino acid, FM for functional monomer, N° conf. for the spatial conformation number of the Amino acid-FM complex, E_{tot} represents the ground state electronic energy in kcal mol^{-1}, the ΔE represents the difference of the electronic energy in ascending order of energy between the complex and lastly, the type of interaction specifies the atoms that are in close proximity in the table. The symbols Hb denote a hydrogen bond, AmtoM indicates that an AM is complexing with an FM, and MtoAm is the reverse of AmtoM. The symbols SB denote a salt bridges. The symbols Hp denote a hydrophobics interactions. The symbols Cation-pi/pi-staquing/pi-T-sheped denote the type of π interactions interactions.

AA	FM	N° conf.	ΔE	E_{tot}	Type of interaction
		7	0.300293738655	-6.10	4H.-.O 2C.-.O N.-.O 6C.-.H 6H.-.H H.-.N SB
		2	0.766620874113	-5.64	2N.-.O 9H.-.O 4C.-.N 12C.-.H 9H.-.H 3C.-.C 7C.-.O H.-.N 2O.-.O 3Hp Hb(AmtoM) 2SB
		4	2.208467189683	-4.19	
		5	2.467312840109	-3.94	2C.-.O O.-.O 4H.-.O 5C.-.H 4H.-.H Hb(AmtoM)
		0	2.647518181887	-3.76	8H.-.O 3C.-.O 2O.-.O 4C.-.H 3H.-.H H.-.N N.-.O Hb(MtoAm) Hb(AmtoM) SB
		1	2.918563469261	-3.48	5C.-.O 9C.-.H 6H.-.O 7H.-.H
		3	3.167982196263	-3.23	5H.-.O 3C.-.O 3O.-.O 3C.-.H 2H.-.H Hb(MtoAm) SB
THR	14dvb	6	0.000000000000	-5.27	26C.-.H 14H.-.H 10C.-.C 3C.-.N H.-.O 2H.-.N 10Hp Cation-pi(Amc)

Continue in the next page

Table SM1: Structures of various conformations are evaluated for their energetic properties and types of intermolecular interactions. In this context, Am stands for amino acid, FM for functional monomer, N° conf. for the spatial conformation number of the Amino acid-FM complex, E_{tot} represents the ground state electronic energy in kcal mol^{-1}, the ΔE represents the difference of the electronic energy in ascending order of energy between the complex and lastly, the type of interaction specifies the atoms that are in close proximity in the table. The symbols Hb denote a hydrogen bond, AmtoM indicates that an AM is complexing with an FM, and MtoAm is the reverse of AmtoM. The symbols SB denote a salt bridges. The symbols Hp denote a hydrophobics interactions. The symbols Cation-pi/pi-staquing/pi-T-sheped denote the type of π interactions interactions.

AA	FM	N° conf.	ΔE	E_{tot}	Type of interaction
		1	1.221663037348	-4.05	21C.-.H 9C.-.C 15H.-.H 3H.-.O 2C.-.O 9Hp Cation-pi(Amc)
		0	1.335983345056	-3.93	22C.-.H 2C.-.O 10C.-.C 12H.-.H 2H.-.O H.-.N 10Hp
		4	3.899232802681	-1.37	3C.-.O C.-.N 8C.-.H 4H.-.O 3H.-.N 7H.-.H
		2	3.922843454408	-1.34	13C.-.H 7H.-.H 8H.-.O 3C.-.C 5C.-.O 3Hp
		3	3.935488997018	-1.33	12C.-.H 9H.-.H C.-.N 2H.-.N
		7	4.631094354280	-0.64	
		8	4.648777796548	-0.62	
		9	5.300543861392	0.03	
	2viny	7	0.000000000000	-6.15	6C.-.C 22C.-.H 6C.-.N 7H.-.N N.-.N 10H.-.H 6Hp Hb(AmtoM)
		8	1.143831870905	-5.01	8C.-.C 19C.-.H 5C.-.N 5H.-.N N.-.N 4C.-.O 13H.-.H 2H.-.O 8Hp Cation-pi(Amc)

Continue in the next page

Table SM1: Structures of various conformations are evaluated for their energetic properties and types of intermolecular interactions. In this context, Am stands for amino acid, FM for functional monomer, N° conf. for the spatial conformation number of the Amino acid-FM complex, E_{tot} represents the ground state electronic energy in kcal mol^{-1}, the ΔE represents the difference of the electronic energy in ascending order of energy between the complex and lastly, the type of interaction specifies the atoms that are in close proximity in the table. The symbols Hb denote a hydrogen bond, AmtoM indicates that an AM is complexing with an FM, and MtoAm is the reverse of AmtoM. The symbols SB denote a salt bridges. The symbols Hp denote a hydrophobics interactions. The symbols Cation-pi/pi-staquing/pi-T-sheped denote the type of π interactions interactions.

AA	FM	N° conf.	ΔE	E_{tot}	Type of interaction
		3	2.070315897496	-4.08	12C.-.H 6H.-.N 2C.-.N N.-.N C.-.C 7H.-.H Hp Hb(AmtoM)
		9	3.599532899251	-2.55	3H.-.N 13C.-.H 4C.-.C 2C.-.N 10H.-.H 4Hp
		6	4.425683494085	-1.73	10C.-.H C.-.O C.-.N 8H.-.H H.-.O H.-.N
		0	5.604003334290	-0.55	
		1	6.251387188623	0.10	
		4	6.370816732356	0.22	H.-.H
	acidm	0	0.000000000000	-4.14	2C.-.O 3O.-.O 3H.-.O C.-.H H.-.H Hb(MtoAm) SB
		6	1.749516050762	-2.39	3C.-.C 11C.-.H 2C.-.O 11H.-.H 7H.-.O C.-.N N.-.O H.-.N 3Hp SB
		2	1.952059781883	-2.19	5C.-.C 16C.-.H 5C.-.O 13H.-.H H.-.N 6H.-.O N.-.O 2O.-.O 5Hp SB

Continue in the next page

Table SM1: Structures of various conformations are evaluated for their energetic properties and types of intermolecular interactions. In this context, Am stands for amino acid, FM for functional monomer, N° conf. for the spatial conformation number of the Amino acid-FM complex, E_{tot} represents the ground state electronic energy in kcal mol^{-1}, the ΔE represents the difference of the electronic energy in ascending order of energy between the complex and lastly, the type of interaction specifies the atoms that are in close proximity in the table. The symbols Hb denote a hydrogen bond, AmtoM indicates that an AM is complexing with an FM, and MtoAm is the reverse of AmtoM. The symbols SB denote a salt bridges. The symbols Hp denote a hydrophobics interactions. The symbols Cation-pi/pi-staquing/pi-T-sheped denote the type of π interactions interactions.

AA	FM	N° conf.	ΔE	E_{tot}	Type of interaction
		7	2.529360966493	-1.61	7C.-.O 6C.-.H 5H.-.H 6H.-.O C.-.C 3O.-.O Hp SB
		8	3.085410075553	-1.05	7C.-.O 8C.-.H 9H.-.O 2C.-.C 4O.-.O 6H.-.H H.-.N 2Hp SB
		4	3.688927518421	-0.45	
		3	4.208835001138	0.07	
		9	4.534329900753	0.40	
	acida	9	0.000000000000	-8.97	C.-.N 5C.-.H 2C.-.O 8H.-.O 2N.-.O 6H.-.H H.-.N Hb(MtoAm) SB
		5	2.557293869775	-6.41	4C.-.O 5C.-.H C.-.N 4O.-.O 8H.-.O N.-.O 4H.-.H H.-.N Hb(MtoAm) Hb(AmtoM) SB
		1	6.626490366416	-2.34	4C.-.C 12C.-.H 9H.-.H 6H.-.O 4C.-.O O.-.O 4Hp SB
		4	6.685108667820	-2.29	6C.-.H C.-.C 3C.-.O 5H.-.O 5H.-.H H.-.N Hp

Continue in the next page

Table SM1: Structures of various conformations are evaluated for their energetic properties and types of intermolecular interactions. In this context, Am stands for amino acid, FM for functional monomer, N° conf. for the spatial conformation number of the Amino acid-FM complex, E_{tot} represents the ground state electronic energy in kcal mol^{-1}, the ΔE represents the difference of the electronic energy in ascending order of energy between the complex and lastly, the type of interaction specifies the atoms that are in close proximity in the table. The symbols Hb denote a hydrogen bond, AmtoM indicates that an AM is complexing with an FM, and MtoAm is the reverse of AmtoM. The symbols SB denote a salt bridges. The symbols Hp denote a hydrophobics interactions. The symbols Cation-pi/pi-staquing/pi-T-sheped denote the type of π interactions interactions.

AA	FM	N° conf.	ΔE	E_{tot}	Type of interaction
		0	7.125066168672	-1.85	2C.-.H 2H.-.N 5H.-.H 4H.-.O N.-.O Hb(AmtoM) SB
		7	7.223654493750	-1.75	3C.-.C 10C.-.H 2C.-.O 7H.-.H 4H.-.O 3Hp
		3	7.629191043600	-1.34	2C.-.C 8C.-.H 11H.-.H 2H.-.N 2Hp
		6	7.986478928880	-0.98	3C.-.C 9C.-.H 2C.-.O 8H.-.H 2H.-.O 3Hp
		2	8.411850466642	-0.56	
	bisac	3	0.000000000000	-6.91	4C.-.O 4C.-.H 4H.-.H 2H.-.O H.-.N 2O.-.O Hb(AmtoM)
		6	1.229712119530	-5.68	5C.-.N 7C.-.O 23C.-.H 8H.-.O 21H.-.H 12C.-.C 6H.-.N 2O.-.O N.-.O 12Hp
		1	1.307905437959	-5.61	25C.-.H 16H.-.H 5C.-.C 4C.-.O 8H.-.O 6C.-.N 7H.-.N 2N.-.O 5Hp Hb(MtoAm) Hb(AmtoM)

Continue in the next page

Table SM1: Structures of various conformations are evaluated for their energetic properties and types of intermolecular interactions. In this context, Am stands for amino acid, FM for functional monomer, N° conf. for the spatial conformation number of the Amino acid-FM complex, E_{tot} represents the ground state electronic energy in kcal mol^{-1}, the ΔE represents the difference of the electronic energy in ascending order of energy between the complex and lastly, the type of interaction specifies the atoms that are in close proximity in the table. The symbols Hb denote a hydrogen bond, AmtoM indicates that an AM is complexing with an FM, and MtoAm is the reverse of AmtoM. The symbols SB denote a salt bridges. The symbols Hp denote a hydrophobics interactions. The symbols Cation-pi/pi-staquing/pi-T-sheped denote the type of π interactions interactions.

AA	FM	N° conf.	ΔE	E_{tot}	Type of interaction
		7	1.323873033360	-5.59	12C.-.H 3C.-.N 8H.-.H 2H.-.N 4C.-.C 7C.-.O 9H.-.O 3N.-.O O.-.O 4Hp Hb(AmtoM) Hb(MtoAm)
		5	1.708807872955	-5.21	3C.-.C 15C.-.H 3C.-.N 13H.-.H 4H.-.N 2C.-.O 5H.-.O N.-.O 3Hp Hb(AmtoM)
		8	1.812164677833	-5.10	8H.-.H 7C.-.H 4H.-.N 5C.-.O 7H.-.O 2O.-.O C.-.N N.-.O Hb(MtoAm)
		9	4.421335006115	-2.49	C.-.C 13C.-.H 2C.-.O 11H.-.H 5H.-.O 3C.-.N 3H.-.N 2N.-.O O.-.O Hp
		2	6.528296562644	-0.39	3H.-.O C.-.O C.-.H 2H.-.H
	1ally	0	0.000000000000	-6.36	9H.-.N 6C.-.N 25C.-.H 2C.-.O 6C.-.C 26H.-.H 4H.-.O N.-.N 6Hp Hb(AmtoM) SB

Continue in the next page

Table SM1: Structures of various conformations are evaluated for their energetic properties and types of intermolecular interactions. In this context, Am stands for amino acid, FM for functional monomer, N° conf. for the spatial conformation number of the Amino acid-FM complex, E_{tot} represents the ground state electronic energy in kcal mol^{-1}, the ΔE represents the difference of the electronic energy in ascending order of energy between the complex and lastly, the type of interaction specifies the atoms that are in close proximity in the table. The symbols Hb denote a hydrogen bond, AmtoM indicates that an AM is complexing with an FM, and MtoAm is the reverse of AmtoM. The symbols SB denote a salt bridges. The symbols Hp denote a hydrophobics interactions. The symbols Cation-pi/pi-staquing/pi-T-sheped denote the type of π interactions interactions.

AA	FM	N° conf.	ΔE	E_{tot}	Type of interaction
		9	4.349620594561	-2.01	9C.-.H 2C.-.N 6H.-.N 12H.-.H 5H.-.O 3C.-.O SB
		4	4.709767189944	-1.65	5C.-.H 2C.-.N 8H.-.H 3H.-.N H.-.O
		7	4.714342053327	-1.65	3H.-.N 7H.-.H 4C.-.H C.-.N C.-.O 2H.-.O
		8	5.683907729302	-0.68	
		5	5.710868714490	-0.65	
		6	5.810913479513	-0.55	
		2	6.307619535898	-0.05	
		3	6.374750310464	0.01	
	4imid	5	0.000000000000	-6.49	11C.-.H 4C.-.N 6H.-.H 6H.-.N N.-.O N.-.N C.-.O H.-.O 2Hb(AmtoM) SB
		8	0.462664919325	-6.03	3C.-.O 5C.-.H 2C.-.N 4H.-.N 3N.-.O 6H.-.H 5H.-.O Hb(MtoAm)

Continue in the next page

Table SM1: Structures of various conformations are evaluated for their energetic properties and types of intermolecular interactions. In this context, Am stands for amino acid, FM for functional monomer, Nº conf. for the spatial conformation number of the Amino acid-FM complex, E_{tot} represents the ground state electronic energy in kcal mol^{-1}, the ΔE represents the difference of the electronic energy in ascending order of energy between the complex and lastly, the type of interaction specifies the atoms that are in close proximity in the table. The symbols Hb denote a hydrogen bond, AmtoM indicates that an AM is complexing with an FM, and MtoAm is the reverse of AmtoM. The symbols SB denote a salt bridges. The symbols Hp denote a hydrophobics interactions. The symbols Cation-pi/pi-staquing/pi-T-sheped denote the type of π interactions interactions.

AA	FM	Nº conf.	ΔE	E_{tot}	Type of interaction
		9	2.395595765917	-4.10	3C.-.O 2O.-.O 6H.-.O 3C.-.H 4H.-.H Hb(AmtoM)
		0	3.645574126716	-2.85	12C.-.H 4C.-.C C.-.O 2C.-.N 6H.-.N 8H.-.H 2H.-.O 4Hp
		1	5.784088939425	-0.71	C.-.N 2H.-.N C.-.C 3C.-.H 4H.-.H Hp
		6	5.826358510814	-0.67	
	acril	9	0.000000000000	-6.44	C.-.C 11C.-.H 5C.-.O 9H.-.H 5H.-.O C.-.N O.-.O N.-.O 4H.-.N N.-.N Hp 2Hb(AmtoM)
		6	3.472038462113	-2.97	2C.-.O 4C.-.H 4H.-.O 6H.-.H N.-.N C.-.N 4H.-.N N.-.O Hb(MtoAm)
		8	3.593589433123	-2.85	8C.-.H 2C.-.O 9H.-.H 3H.-.O 3C.-.N 4H.-.N N.-.O Hb(MtoAm)
		2	4.436666156681	-2.00	8C.-.H 10H.-.H 3C.-.N 7H.-.N N.-.N N.-.O H.-.O 2Hb(AmtoM)

Continue in the next page

Table SM1: Structures of various conformations are evaluated for their energetic properties and types of intermolecular interactions. In this context, Am stands for amino acid, FM for functional monomer, N° conf. for the spatial conformation number of the Amino acid-FM complex, E_{tot} represents the ground state electronic energy in kcal mol^{-1}, the ΔE represents the difference of the electronic energy in ascending order of energy between the complex and lastly, the type of interaction specifies the atoms that are in close proximity in the table. The symbols Hb denote a hydrogen bond, AmtoM indicates that an AM is complexing with an FM, and MtoAm is the reverse of AmtoM. The symbols SB denote a salt bridges. The symbols Hp denote a hydrophobics interactions. The symbols Cation-pi/pi-staquing/pi-T-sheped denote the type of π interactions interactions.

AA	FM	N° conf.	ΔE	E_{tot}	Type of interaction
		5	5.153269636909	-1.29	C.-.H H.-.O 2H.-.N 3H.-.H
		7	5.600115163617	-0.84	C.-.N 3C.-.H H.-.N 5H.-.H
		3	5.890874241703	-0.55	
		4	6.043275483685	-0.40	
		0	6.491403342696	0.05	
	alila	6	0.000000000000	-12.16	erro
		9	7.067531232735	-5.09	2C.-.C 4C.-.N 12C.-.H 9H.-.N 15H.-.H H.-.O N.-.N 2Hp Hb(AmtoM) SB
		1	9.934469268404	-2.23	5C.-.O 5C.-.H 7H.-.O 5H.-.H C.-.C Hp SB
		3	10.895190678700	-1.27	3C.-.H 7H.-.H C.-.N 2H.-.N
		7	10.994489228813	-1.17	7C.-.H 6H.-.H 3H.-.O C.-.C Hp
		2	11.895149061944	-0.27	
		5	12.184288271909	0.02	
	estir	9	0.000000000000	-4.48	9C.-.C 21C.-.H 15H.-.H 3C.-.N H.-.N 9Hp

Continue in the next page

Table SM1: Structures of various conformations are evaluated for their energetic properties and types of intermolecular interactions. In this context, Am stands for amino acid, FM for functional monomer, N° conf. for the spatial conformation number of the Amino acid-FM complex, E_{tot} represents the ground state electronic energy in kcal mol^{-1}, the ΔE represents the difference of the electronic energy in ascending order of energy between the complex and lastly, the type of interaction specifies the atoms that are in close proximity in the table. The symbols Hb denote a hydrogen bond, AmtoM indicates that an AM is complexing with an FM, and MtoAm is the reverse of AmtoM. The symbols SB denote a salt bridges. The symbols Hp denote a hydrophobics interactions. The symbols Cation-pi/pi-staquing/pi-T-sheped denote the type of π interactions interactions.

AA	FM	N° conf.	ΔE	E_{tot}	Type of interaction
		5	0.009555005178	-4.47	7C.-.C 24C.-.H 3C.-.N 17H.-.H H.-.N 7Hp Cation-pi(Amc)
		0	1.851516468065	-2.63	16C.-.H 6C.-.O 10H.-.H 4H.-.O 4C.-.C 4Hp Cation-pi(Amc)
		6	2.371037923733	-2.11	9C.-.H 4H.-.O 9H.-.H 3C.-.O C.-.N 2H.-.N
		4	2.588606286199	-1.89	5C.-.O 5C.-.H 5H.-.O 4H.-.H C.-.C Hp
		7	4.017909467823	-0.46	
		2	4.808177237886	0.33	
	1viny	2	0.000000000000	-5.08	3C.-.O 8C.-.H 3C.-.N 5H.-.N N.-.O N.-.N 6H.-.H 3H.-.O Hb(AmtoM)
		3	1.441188285014	-3.63	17C.-.H 4C.-.N 6H.-.N 6C.-.C 2C.-.O 11H.-.H 2H.-.O 6Hp
		1	1.741569337786	-3.33	7C.-.O 10C.-.H 3C.-.C 6H.-.O 8H.-.H 2N.-.O 3H.-.N C.-.N 3Hp

Continue in the next page

Table SM1: Structures of various conformations are evaluated for their energetic properties and types of intermolecular interactions. In this context, Am stands for amino acid, FM for functional monomer, N° conf. for the spatial conformation number of the Amino acid-FM complex, E_{tot} represents the ground state electronic energy in kcal mol^{-1}, the ΔE represents the difference of the electronic energy in ascending order of energy between the complex and lastly, the type of interaction specifies the atoms that are in close proximity in the table. The symbols Hb denote a hydrogen bond, AmtoM indicates that an AM is complexing with an FM, and MtoAm is the reverse of AmtoM. The symbols SB denote a salt bridges. The symbols Hp denote a hydrophobics interactions. The symbols Cation-pi/pi-staquing/pi-T-sheped denote the type of π interactions interactions.

AA	FM	N° conf.	ΔE	E_{tot}	Type of interaction
		7	2.001258283236	-3.07	15C.-.H 4H.-.N C.-.N 2N.-.O 8C.-.C 3C.-.O 13H.-.H 2H.-.O 8Hp Cation-pi(Amc)
		5	2.326047142337	-2.75	3C.-.C 12C.-.H C.-.N 4H.-.N 9H.-.H 3Hp
		4	4.496942765112	-0.58	
		9	4.533969870799	-0.54	
	2hydr	6	0.000000000000	-9.60	6C.-.O 9H.-.O 5C.-.H 4O.-.O C.-.C 3H.-.H Hp Hb(AmtoM)
		4	2.678950534245	-6.92	3C.-.H 2C.-.O 5H.-.O 3H.-.H 2O.-.O Hb(AmtoM)
		2	2.791910129471	-6.81	5C.-.H C.-.C 3C.-.O 2O.-.O 7H.-.O 3H.-.H Hp Hb(AmtoM)
		3	3.638347468731	-5.96	3C.-.C 10C.-.O 5C.-.H 4H.-.H 7H.-.O 3O.-.O 3Hp Hb(AmtoM)
		0	6.261044886871	-3.34	9H.-.O 3C.-.C 9C.-.H 5C.-.O 10H.-.H 3H.-.N N.-.O O.-.O 3Hp Hb(AmtoM) Hb(MtoAm)

Continue in the next page

Table SM1: Structures of various conformations are evaluated for their energetic properties and types of intermolecular interactions. In this context, Am stands for amino acid, FM for functional monomer, N° conf. for the spatial conformation number of the Amino acid-FM complex, E_{tot} represents the ground state electronic energy in kcal mol^{-1}, the ΔE represents the difference of the electronic energy in ascending order of energy between the complex and lastly, the type of interaction specifies the atoms that are in close proximity in the table. The symbols Hb denote a hydrogen bond, AmtoM indicates that an AM is complexing with an FM, and MtoAm is the reverse of AmtoM. The symbols SB denote a salt bridges. The symbols Hp denote a hydrophobics interactions. The symbols Cation-pi/pi-staquing/pi-T-sheped denote the type of π interactions interactions.

AA	FM	N° conf.	ΔE	E_{tot}	Type of interaction
		1	7.182778249039	-2.41	6C.-.C 24C.-.H 14H.-.H 5C.-.O 8H.-.O 6Hp
		5	7.572238727729	-2.03	12C.-.H C.-.C C.-.O 4H.-.O 13H.-.H Hp
		7	8.390877006854	-1.21	14C.-.H 4C.-.O N.-.O 6H.-.O O.-.O 3H.-.N 15H.-.H 3C.-.C 2C.-.N 3Hp
		9	8.998085287340	-0.60	
		8	9.705426014705	0.11	
	4viny	8	0.000000000000	-11.13	3C.-.O 5C.-.H 3H.-.O 2H.-.H C.-.N 2N.-.O H.-.N C.-.C Hp Hb(AmtoM)
		5	6.332421729986	-4.80	18C.-.H 7C.-.C 5C.-.N 3H.-.N N.-.O 13H.-.H 3C.-.O 2H.-.O 7Hp Cation-pi(Amc)
		1	6.584539712894	-4.54	12C.-.C 21C.-.H C.-.N 3H.-.N 12H.-.H 2C.-.O 2H.-.O 12Hp

Continue in the next page

Table SM1: Structures of various conformations are evaluated for their energetic properties and types of intermolecular interactions. In this context, Am stands for amino acid, FM for functional monomer, N° conf. for the spatial conformation number of the Amino acid-FM complex, E_{tot} represents the ground state electronic energy in kcal mol^{-1}, the ΔE represents the difference of the electronic energy in ascending order of energy between the complex and lastly, the type of interaction specifies the atoms that are in close proximity in the table. The symbols Hb denote a hydrogen bond, AmtoM indicates that an AM is complexing with an FM, and MtoAm is the reverse of AmtoM. The symbols SB denote a salt bridges. The symbols Hp denote a hydrophobics interactions. The symbols Cation-pi/pi-staquing/pi-T-sheped denote the type of π interactions interactions.

AA	FM	N° conf.	ΔE	E_{tot}	Type of interaction
		6	6.752899830447	-4.38	9C.-.C 20C.-.H C.-.N H.-.N 14H.-.H 3C.-.O 2H.-.O 9Hp
		7	6.931425130403	-4.20	6C.-.C 14C.-.H C.-.O 10H.-.H H.-.O 4C.-.N 4H.-.N N.-.O 6Hp Cation-pi(Amc)
		0	7.042685571405	-4.09	9C.-.C 21C.-.H 15H.-.H 3C.-.N 3H.-.N 9Hp
		4	9.751117179996	-1.38	erro
		4	9.751521219148	-1.38	7C.-.H 2C.-.N 7H.-.H H.-.O 2H.-.N
		3	11.428715105875	0.30	
	acrol	9	0.000000000000	-6.00	6C.-.H 4C.-.O 4H.-.O 3H.-.H 2O.-.O Hb(AmtoM)
		6	1.316577702928	-4.68	C.-.C 10C.-.H 4C.-.O 6H.-.H 4H.-.O O.-.O N.-.O Hp Hb(AmtoM)
		7	4.124690372686	-1.88	9C.-.H 2C.-.N H.-.N 8H.-.H 2C.-.C H.-.O 2Hp

Continue in the next page

Table SM1: Structures of various conformations are evaluated for their energetic properties and types of intermolecular interactions. In this context, Am stands for amino acid, FM for functional monomer, N° conf. for the spatial conformation number of the Amino acid-FM complex, E_{tot} represents the ground state electronic energy in kcal mol^{-1}, the ΔE represents the difference of the electronic energy in ascending order of energy between the complex and lastly, the type of interaction specifies the atoms that are in close proximity in the table. The symbols Hb denote a hydrogen bond, AmtoM indicates that an AM is complexing with an FM, and MtoAm is the reverse of AmtoM. The symbols SB denote a salt bridges. The symbols Hp denote a hydrophobics interactions. The symbols Cation-pi/pi-staquing/pi-T-sheped denote the type of π interactions interactions.

AA	FM	N° conf.	ΔE	E_{tot}	Type of interaction
		0	4.435448361737	-1.56	8C.-.H 2C.-.O 6H.-.O 6H.-.H H.-.N C.-.N N.-.O
		8	4.820761172732	-1.18	4C.-.O 6C.-.H 5H.-.H 5H.-.O 2C.-.C O.-.O 2Hp
		5	5.423566253432	-0.58	
		4	5.601115294075	-0.40	4C.-.H C.-.O 4H.-.H H.-.O
	itaco	8	0.000000000000	-10.05	6C.-.O 9C.-.H 9H.-.O 4O.-.O 6H.-.H H.-.N Hb(AmtoM) Hb(MtoAm)
		4	0.960471888389	-9.09	3C.-.O 5H.-.O 2O.-.O 4C.-.H 5H.-.H Hb(MtoAm) SB
		2	2.868947675468	-7.18	9C.-.O 13H.-.O 3C.-.C 15C.-.H 9H.-.H 3O.-.O 3Hp
		0	3.676339921069	-6.37	11C.-.O 3O.-.O 9H.-.O 8H.-.H 5C.-.C 10C.-.H 5Hp Hb(AmtoM)
		5	4.795103227280	-5.25	9H.-.O 6C.-.O 9C.-.H 6H.-.H 2O.-.O C.-.C H.-.N Hp Hb(AmtoM)

Continue in the next page

Table SM1: Structures of various conformations are evaluated for their energetic properties and types of intermolecular interactions. In this context, Am stands for amino acid, FM for functional monomer, N° conf. for the spatial conformation number of the Amino acid-FM complex, E_{tot} represents the ground state electronic energy in $kcal\,mol^{-1}$, the ΔE represents the difference of the electronic energy in ascending order of energy between the complex and lastly, the type of interaction specifies the atoms that are in close proximity in the table. The symbols Hb denote a hydrogen bond, AmtoM indicates that an AM is complexing with an FM, and MtoAm is the reverse of AmtoM. The symbols SB denote a salt bridges. The symbols Hp denote a hydrophobics interactions. The symbols Cation-pi/pi-staquing/pi-T-sheped denote the type of π interactions interactions.

AA	FM	N° conf.	ΔE	E_{tot}	Type of interaction
		1	5.090094636576	-4.96	2N.-.O 8H.-.O 2H.-.N 8H.-.H 14C.-.H 2C.-.N 2C.-.O C.-.C Hp SB
		3	5.660967587620	-4.39	5H.-.O 11H.-.H 16C.-.H 2C.-.O 2C.-.C 2Hp
		7	7.540941692110	-2.51	
		6	10.562980476888	10.52	
TRP	14dvb	3	0.000000000000	-5.70	5C.-.N 21C.-.H 14H.-.H 10C.-.C 4H.-.N 3C.-.O H.-.O 10Hp Cation-pi(Amc)
		6	0.817139577366	-4.89	23C.-.H 10C.-.C 3C.-.O 11H.-.H 2H.-.O 10Hp pi-T-sheped
		8	1.127079797757	-4.58	14C.-.H 6C.-.C 11H.-.H 4C.-.N 4H.-.N 6Hp pi-T-sheped
		2	1.188952449388	-4.51	22C.-.H 12H.-.H 6C.-.C 2C.-.O 3H.-.O 6Hp pi-T-sheped
	2viny	0	0.000000000000	-6.21	21C.-.C 26C.-.H 6C.-.N 5H.-.N 12H.-.H 21Hp 2pi-staquing

Continue in the next page

Table SM1: Structures of various conformations are evaluated for their energetic properties and types of intermolecular interactions. In this context, Am stands for amino acid, FM for functional monomer, N° conf. for the spatial conformation number of the Amino acid-FM complex, E_{tot} represents the ground state electronic energy in kcal mol^{-1}, the ΔE represents the difference of the electronic energy in ascending order of energy between the complex and lastly, the type of interaction specifies the atoms that are in close proximity in the table. The symbols Hb denote a hydrogen bond, AmtoM indicates that an AM is complexing with an FM, and MtoAm is the reverse of AmtoM. The symbols SB denote a salt bridges. The symbols Hp denote a hydrophobics interactions. The symbols Cation-pi/pi-staquing/pi-T-sheped denote the type of π interactions interactions.

AA	FM	N° conf.	ΔE	E_{tot}	Type of interaction
		5	3.839742456277	-2.37	2H.-.N 14C.-.H 4C.-.C C.-.N 7H.-.H 2H.-.O 4Hp Cation-pi(Amc)
		2	6.181220941223	-0.03	
		3	6.294037305089	0.09	
		6	7.403490340223	1.20	2C.-.O 2C.-.H 2H.-.O 2H.-.H
	acidm	8	0.000000000000	-5.54	4C.-.N 14C.-.C 27C.-.H 6H.-.N 19H.-.H 8C.-.O 6H.-.O N.-.O 14Hp Hb(AmtoM) SB
		4	1.645119676009	-3.89	C.-.C 7C.-.H 9H.-.H H.-.N 5C.-.O 2O.-.O 7H.-.O Hp Hb(MtoAm) SB
		5	2.982723794467	-2.56	3H.-.N 8H.-.H 8C.-.H C.-.C C.-.N 3N.-.O 7C.-.O 5H.-.O Hp Hb(MtoAm) SB
		9	4.417799883691	-1.12	C.-.N 6C.-.H H.-.N 5H.-.H 5H.-.O 3C.-.C 4C.-.O 2O.-.O 3Hp SB

Continue in the next page

Table SM1: Structures of various conformations are evaluated for their energetic properties and types of intermolecular interactions. In this context, Am stands for amino acid, FM for functional monomer, N° conf. for the spatial conformation number of the Amino acid-FM complex, E_{tot} represents the ground state electronic energy in $\mathrm{kcal\,mol^{-1}}$, the ΔE represents the difference of the electronic energy in ascending order of energy between the complex and lastly, the type of interaction specifies the atoms that are in close proximity in the table. The symbols Hb denote a hydrogen bond, AmtoM indicates that an AM is complexing with an FM, and MtoAm is the reverse of AmtoM. The symbols SB denote a salt bridges. The symbols Hp denote a hydrophobics interactions. The symbols Cation-pi/pi-staquing/pi-T-sheped denote the type of π interactions interactions.

AA	FM	N° conf.	ΔE	E_{tot}	Type of interaction
		3	5.262980968445	-0.28	4C.-.O 5C.-.H 7H.-.O 5H.-.H C.-.C Hp
		1	5.450349905084	-0.09	
		6	5.742012635587	0.20	
		2	6.387977505822	0.85	
	acida	8	0.000000000000	-5.16	5C.-.N 25C.-.H 14C.-.C 3H.-.N 9H.-.H 4H.-.O 2C.-.O N.-.O 14Hp SB
		4	1.477292416693	-3.68	12C.-.O 10C.-.H 6H.-.H 8H.-.O 2O.-.O
		0	2.787883518377	-2.37	C.-.N 6C.-.C 8C.-.H 6C.-.O 2H.-.N 6H.-.O 4H.-.H 2O.-.O 6Hp SB
		6	3.026155816214	-2.13	C.-.N 7C.-.H C.-.C H.-.N 5H.-.H 4H.-.O N.-.O 2C.-.O 2O.-.O Hp SB
		1	3.461214031976	-1.70	13C.-.H 2C.-.C 2C.-.O 8H.-.H 2H.-.O 2Hp

Continue in the next page

Table SM1: Structures of various conformations are evaluated for their energetic properties and types of intermolecular interactions. In this context, Am stands for amino acid, FM for functional monomer, Nº conf. for the spatial conformation number of the Amino acid-FM complex, E_{tot} represents the ground state electronic energy in kcal mol^{-1}, the ΔE represents the difference of the electronic energy in ascending order of energy between the complex and lastly, the type of interaction specifies the atoms that are in close proximity in the table. The symbols Hb denote a hydrogen bond, AmtoM indicates that an AM is complexing with an FM, and MtoAm is the reverse of AmtoM. The symbols SB denote a salt bridges. The symbols Hp denote a hydrophobics interactions. The symbols Cation-pi/pi-staquing/pi-T-sheped denote the type of π interactions interactions.

AA	FM	Nº conf.	ΔE	E_{tot}	Type of interaction
		7	5.040321239890	-0.12	
		3	5.040664057179	-0.12	
		5	5.056311474229	-0.10	
	bisac	7	0.000000000000	-9.65	5C.-.O 5H.-.H 6C.-.H 6H.-.O C.-.N N.-.O 2H.-.N 2O.-.O Hb(MtoAm) Hb(AmtoM)
		0	3.521511109162	-6.13	15C.-.H 3C.-.C 8H.-.H 4C.-.N 4H.-.N 3H.-.O 3C.-.O N.-.O 3Hp Hb(AmtoM)
		4	5.329407951028	-4.33	2C.-.N 23C.-.H 3C.-.O 6C.-.C 4H.-.N 17H.-.H 4H.-.O 6Hp
		9	6.476370489744	-3.18	4H.-.N 2C.-.C 11C.-.H 8H.-.H 2C.-.N N.-.O 4C.-.O 5H.-.O O.-.O 2Hp Hb(AmtoM)
		2	6.803907223619	-2.85	12C.-.H 9H.-.H 4H.-.O 3H.-.N C.-.N N.-.O 3C.-.O O.-.O Hb(MtoAm)

Continue in the next page

Table SM1: Structures of various conformations are evaluated for their energetic properties and types of intermolecular interactions. In this context, Am stands for amino acid, FM for functional monomer, N° conf. for the spatial conformation number of the Amino acid-FM complex, E_{tot} represents the ground state electronic energy in kcal mol^{-1}, the ΔE represents the difference of the electronic energy in ascending order of energy between the complex and lastly, the type of interaction specifies the atoms that are in close proximity in the table. The symbols Hb denote a hydrogen bond, AmtoM indicates that an AM is complexing with an FM, and MtoAm is the reverse of AmtoM. The symbols SB denote a salt bridges. The symbols Hp denote a hydrophobics interactions. The symbols Cation-pi/pi-staquing/pi-T-sheped denote the type of π interactions interactions.

AA	FM	N° conf.	ΔE	E_{tot}	Type of interaction
	1ally	9	0.000000000000	-5.87	5C.-.N 34C.-.H 10H.-.N 24H.-.H 12C.-.C 12Hp Cation-pi(Mc)
		1	1.156543835439	-4.71	24C.-.H 7C.-.C C.-.O 23H.-.H 2H.-.O 4C.-.N 6H.-.N 7Hp SB
		4	3.339967096752	-2.53	6C.-.C 19C.-.H 14H.-.H C.-.N H.-.N 6Hp Cation-pi(Mc)
		3	4.394107394193	-1.47	2C.-.C 2C.-.N 12C.-.H 3H.-.N 10H.-.H 2Hp
		2	4.622724756064	-1.24	7H.-.H 2C.-.C 8C.-.H 2Hp
		8	5.693315672094	-0.17	
		7	5.731666559281	-0.13	
		6	5.879159604105	0.01	
		0	6.160111700589	0.29	
	4imid	2	0.000000000000	-6.39	H.-.O 23C.-.H 5C.-.N 7H.-.N 11H.-.H N.-.N 5C.-.C 5Hp Hb(MtoAm)

Continue in the next page

Table SM1: Structures of various conformations are evaluated for their energetic properties and types of intermolecular interactions. In this context, Am stands for amino acid, FM for functional monomer, N° conf. for the spatial conformation number of the Amino acid-FM complex, E_{tot} represents the ground state electronic energy in kcal mol^{-1}, the ΔE represents the difference of the electronic energy in ascending order of energy between the complex and lastly, the type of interaction specifies the atoms that are in close proximity in the table. The symbols Hb denote a hydrogen bond, AmtoM indicates that an AM is complexing with an FM, and MtoAm is the reverse of AmtoM. The symbols SB denote a salt bridges. The symbols Hp denote a hydrophobics interactions. The symbols Cation-pi/pi-staquing/pi-T-sheped denote the type of π interactions interactions.

AA	FM	N° conf.	ΔE	E_{tot}	Type of interaction
		3	0.945901763375	-5.45	9C.-.O 5H.-.O 17C.-.C 6C.-.N 32C.-.H 12H.-.H 2N.-.O 7H.-.N 17Hp Cation-pi(Amc)
		0	1.261740889408	-5.13	3H.-.O O.-.O 20C.-.H 11C.-.C C.-.O 6H.-.N 10H.-.H 6C.-.N N.-.N 11Hp 2pi-staquing
		4	3.311069020184	-3.08	9C.-.O 7H.-.O 5C.-.C 11C.-.H 8H.-.H N.-.O 2H.-.N 5Hp
		5	6.764970598025	0.37	
	acril	6	0.000000000000	-8.65	10C.-.H 2C.-.O 2O.-.O 3H.-.O N.-.O 5H.-.N 2C.-.N N.-.N 6H.-.H Hb(AmtoM)
		5	4.243542343420	-4.41	15C.-.H 4C.-.C 3C.-.O 12H.-.H 6H.-.O 2C.-.N N.-.N 6H.-.N N.-.O 4Hp Hb(AmtoM) Hb(MtoAm)

Continue in the next page

Table SM1: Structures of various conformations are evaluated for their energetic properties and types of intermolecular interactions. In this context, Am stands for amino acid, FM for functional monomer, N° conf. for the spatial conformation number of the Amino acid-FM complex, E_{tot} represents the ground state electronic energy in $kcal\,mol^{-1}$, the ΔE represents the difference of the electronic energy in ascending order of energy between the complex and lastly, the type of interaction specifies the atoms that are in close proximity in the table. The symbols Hb denote a hydrogen bond, AmtoM indicates that an AM is complexing with an FM, and MtoAm is the reverse of AmtoM. The symbols SB denote a salt bridges. The symbols Hp denote a hydrophobics interactions. The symbols Cation-pi/pi-staquing/pi-T-sheped denote the type of π interactions interactions.

AA	FM	N° conf.	ΔE	E_{tot}	Type of interaction
		2	4.281653074478	-4.37	8C.-.N 17C.-.H 3C.-.C 10H.-.H 6H.-.N N.-.N H.-.O 3Hp Hb(AmtoM)
		8	8.463346537269	-0.19	
		4	8.621379118261	-0.03	
		9	8.659841956459	0.01	
		3	8.877227541667	0.23	
		7	8.897379886033	0.25	
		0	9.684170349299	1.03	
	alila	4	0.000000000000	-3.91	5C.-.C 7C.-.N 18C.-.H 11H.-.H 2H.-.N 5Hp 2Cation-pi(Mc)
		3	2.002207374570	-1.90	3C.-.C 13C.-.H 8H.-.H 2H.-.N 3Hp
		5	2.053601492021	-1.85	5C.-.N N.-.N 4H.-.N 13C.-.H 6H.-.H 2Cation-pi(Mc)
		9	3.950569907079	0.05	
		8	4.012489523214	0.11	
		2	4.168397343843	0.26	

Continue in the next page

Table SM1: Structures of various conformations are evaluated for their energetic properties and types of intermolecular interactions. In this context, Am stands for amino acid, FM for functional monomer, N° conf. for the spatial conformation number of the Amino acid-FM complex, E_{tot} represents the ground state electronic energy in $kcal\,mol^{-1}$, the ΔE represents the difference of the electronic energy in ascending order of energy between the complex and lastly, the type of interaction specifies the atoms that are in close proximity in the table. The symbols Hb denote a hydrogen bond, AmtoM indicates that an AM is complexing with an FM, and MtoAm is the reverse of AmtoM. The symbols SB denote a salt bridges. The symbols Hp denote a hydrophobics interactions. The symbols Cation-pi/pi-staquing/pi-T-sheped denote the type of π interactions interactions.

AA	FM	N° conf.	ΔE	E_{tot}	Type of interaction
	estir	2	0.000000000000	-4.95	22C.-.C 24C.-.H 11H.-.H 2C.-.N 2H.-.N 22Hp 2pi-staquing
		4	0.398718573521	-4.55	11C.-.C 24C.-.H 14H.-.H 2C.-.N 4H.-.N 11Hp pi-T-sheped
		9	0.698785419235	-4.25	22C.-.H 12H.-.H 10C.-.C H.-.N 10Hp
		8	1.798254489949	-3.15	6C.-.C 14C.-.H 2C.-.N 7H.-.H 6Hp
	1viny	0	0.000000000000	-5.90	19C.-.H 15C.-.C 13C.-.N N.-.N 8H.-.N 13H.-.H 15Hp Hb(AmtoM) 2pi-staquing
		3	1.309620846929	-4.59	5C.-.N 19C.-.H 8C.-.C 6H.-.N 10H.-.H 8Hp 2pi-T-sheped
		4	1.489822462880	-4.41	5H.-.N 18C.-.H 3C.-.N 9C.-.C 15H.-.H 9Hp pi-T-sheped
		7	2.973004113164	-2.93	2C.-.N 6C.-.H C.-.O 2C.-.C N.-.N 5H.-.N N.-.O 5H.-.H H.-.O 2Hp Hb(AmtoM)

Continue in the next page

Table SM1: Structures of various conformations are evaluated for their energetic properties and types of intermolecular interactions. In this context, Am stands for amino acid, FM for functional monomer, N° conf. for the spatial conformation number of the Amino acid-FM complex, E_{tot} represents the ground state electronic energy in kcal mol^{-1}, the ΔE represents the difference of the electronic energy in ascending order of energy between the complex and lastly, the type of interaction specifies the atoms that are in close proximity in the table. The symbols Hb denote a hydrogen bond, AmtoM indicates that an AM is complexing with an FM, and MtoAm is the reverse of AmtoM. The symbols SB denote a salt bridges. The symbols Hp denote a hydrophobics interactions. The symbols Cation-pi/pi-staquing/pi-T-sheped denote the type of π interactions interactions.

AA	FM	N° conf.	ΔE	E_{tot}	Type of interaction
		1	4.809041036210	-1.09	C.-.C 6C.-.H 2H.-.H C.-.N 2H.-.N Hp
		5	5.989906553476	0.09	
		6	6.128036880819	0.23	
		2	6.144745415286	0.24	
		9	6.667475274280	0.77	
	2hydr	5	0.000000000000	-7.15	15C.-.C 29C.-.H 18H.-.H 2N.-.O 9H.-.O 8C.-.O 3C.-.N 5H.-.N 15Hp Hb(AmtoM)
		4	1.588168889502	-5.56	28C.-.H 13C.-.C 11H.-.H 2H.-.N 5C.-.O 4H.-.O N.-.O 13Hp
		1	4.023772397977	-3.12	8C.-.H 6C.-.O 7H.-.O 8H.-.H O.-.O Hb(MtoAm)
		8	4.913017353890	-2.23	6C.-.O 3C.-.C 19C.-.H 10H.-.H H.-.N 6H.-.O O.-.O 3Hp Hb(MtoAm)
		9	7.056325473822	-0.09	
		3	7.190872372840	0.04	

Continue in the next page

Table SM1: Structures of various conformations are evaluated for their energetic properties and types of intermolecular interactions. In this context, Am stands for amino acid, FM for functional monomer, Nº conf. for the spatial conformation number of the Amino acid-FM complex, E_{tot} represents the ground state electronic energy in kcal mol^{-1}, the ΔE represents the difference of the electronic energy in ascending order of energy between the complex and lastly, the type of interaction specifies the atoms that are in close proximity in the table. The symbols Hb denote a hydrogen bond, AmtoM indicates that an AM is complexing with an FM, and MtoAm is the reverse of AmtoM. The symbols SB denote a salt bridges. The symbols Hp denote a hydrophobics interactions. The symbols Cation-pi/pi-staquing/pi-T-sheped denote the type of π interactions interactions.

AA	FM	Nº conf.	ΔE	E_{tot}	Type of interaction
	4viny	4	0.000000000000	-9.56	3C.-.O 5C.-.H C.-.N 2N.-.O H.-.N 3H.-.O 2H.-.H C.-.C Hp Hb(AmtoM)
		6	0.564154653007	-9.00	3C.-.O 5C.-.H 3H.-.O 2H.-.H C.-.N 2N.-.O H.-.N C.-.C Hp Hb(AmtoM)
		1	4.561718916111	-5.00	16C.-.C 21C.-.H 12H.-.H 7C.-.N N.-.N 6H.-.N 16Hp 2pi-staquing
		8	5.029176340087	-4.53	24C.-.H 17C.-.C 11H.-.H 4C.-.N 2H.-.N 17Hp pi-staquing
		9	9.441503252629	-0.12	
		5	9.685513153027	0.12	
		0	9.877878806368	0.31	
	acrol	3	0.000000000000	-4.81	19C.-.C 23C.-.H 9H.-.H C.-.N 2H.-.N 4C.-.O 3H.-.O 19Hp
		1	2.555324873466	-2.25	7C.-.H 6C.-.O 6H.-.H 5H.-.O O.-.O
		0	4.879586664570	0.07	

Continue in the next page

Table SM1: Structures of various conformations are evaluated for their energetic properties and types of intermolecular interactions. In this context, Am stands for amino acid, FM for functional monomer, Nº conf. for the spatial conformation number of the Amino acid-FM complex, E_{tot} represents the ground state electronic energy in kcal mol^{-1}, the ΔE represents the difference of the electronic energy in ascending order of energy between the complex and lastly, the type of interaction specifies the atoms that are in close proximity in the table. The symbols Hb denote a hydrogen bond, AmtoM indicates that an AM is complexing with an FM, and MtoAm is the reverse of AmtoM. The symbols SB denote a salt bridges. The symbols Hp denote a hydrophobics interactions. The symbols Cation-pi/pi-staquing/pi-T-sheped denote the type of π interactions interactions.

AA	FM	Nº conf.	ΔE	E_{tot}	Type of interaction
	itaco	4	0.000000000000	-10.34	17C.-.C 13C.-.O 20C.-.H 8H.-.O 10H.-.H 2C.-.N 2H.-.N 17Hp
		9	2.702660857995	-7.63	C.-.N N.-.O H.-.N 12H.-.O 13C.-.H 8H.-.H 3C.-.C 9C.-.O 2O.-.O 3Hp Hb(MtoAm) Hb(AmtoM) SB
		8	3.361917588867	-6.97	20C.-.H 10C.-.C 12H.-.H 3C.-.O C.-.N 3H.-.N N.-.O 4H.-.O 10Hp
		3	4.254639516388	-6.08	N.-.O 2H.-.N 7H.-.O 11H.-.H 18C.-.H 7C.-.O 6C.-.C 6Hp Hb(MtoAm) SB
		1	8.068421945580	-2.27	
TYR	14dvb	2	0.000000000000	-6.16	22C.-.C 33C.-.H 16H.-.H 3H.-.O 22Hp pi-staquing
		1	1.473013616069	-4.68	14H.-.H 4C.-.O 17C.-.C 26C.-.H C.-.N 2H.-.N 17Hp pi-staquing
		7	1.577121464474	-4.58	2C.-.N 25C.-.H 2H.-.N 15H.-.H 7C.-.C 7Hp pi-T-sheped

Continue in the next page

Table SM1: Structures of various conformations are evaluated for their energetic properties and types of intermolecular interactions. In this context, Am stands for amino acid, FM for functional monomer, N° conf. for the spatial conformation number of the Amino acid-FM complex, E_{tot} represents the ground state electronic energy in kcal mol^{-1}, the ΔE represents the difference of the electronic energy in ascending order of energy between the complex and lastly, the type of interaction specifies the atoms that are in close proximity in the table. The symbols Hb denote a hydrogen bond, AmtoM indicates that an AM is complexing with an FM, and MtoAm is the reverse of AmtoM. The symbols SB denote a salt bridges. The symbols Hp denote a hydrophobics interactions. The symbols Cation-pi/pi-staquing/pi-T-sheped denote the type of π interactions interactions.

AA	FM	N° conf.	ΔE	E_{tot}	Type of interaction
		3	2.262436743515	-3.89	23C.-.C 30C.-.H 14H.-.H 6H.-.O 9C.-.O 23Hp pi-staquing
		5	2.732341814001	-3.42	14C.-.H 9H.-.H 5C.-.C 3C.-.O 3H.-.O 5Hp
		4	3.866273519248	-2.29	2H.-.O 12C.-.H 6C.-.C 3C.-.O 7H.-.H 6Hp pi-T-sheped
		6	4.038049629143	-2.12	C.-.C 8C.-.H 2C.-.O 10H.-.H 3H.-.O Hp
	2viny	0	0.000000000000	-8.08	11C.-.H 3C.-.O 2C.-.N 2H.-.N N.-.O 2C.-.C 2H.-.O 5H.-.H 2Hp Hb(AmtoM)
		2	3.063471725887	-5.01	5C.-.C 17C.-.H N.-.N 5H.-.N 4C.-.N 2N.-.O 11H.-.H 2C.-.O 2H.-.O 5Hp
		9	3.162175534781	-4.91	29C.-.H 16C.-.C N.-.N 6C.-.N 6H.-.N 12H.-.H C.-.O 3H.-.O 16Hp Hb(AmtoM) pi-staquing

Continue in the next page

Table SM1: Structures of various conformations are evaluated for their energetic properties and types of intermolecular interactions. In this context, Am stands for amino acid, FM for functional monomer, N° conf. for the spatial conformation number of the Amino acid-FM complex, E_{tot} represents the ground state electronic energy in kcal mol^{-1}, the ΔE represents the difference of the electronic energy in ascending order of energy between the complex and lastly, the type of interaction specifies the atoms that are in close proximity in the table. The symbols Hb denote a hydrogen bond, AmtoM indicates that an AM is complexing with an FM, and MtoAm is the reverse of AmtoM. The symbols SB denote a salt bridges. The symbols Hp denote a hydrophobics interactions. The symbols Cation-pi/pi-staquing/pi-T-sheped denote the type of π interactions interactions.

AA	FM	N° conf.	ΔE	E_{tot}	Type of interaction
		3	3.967758144965	-4.11	8C.-.C 19C.-.H 3C.-.N 3H.-.N 11H.-.H 2H.-.O 8Hp Cation-pi(Amc)
		1	5.430078785696	-2.65	2C.-.C 11C.-.H 3C.-.O C.-.N 2H.-.N 7H.-.H 2H.-.O 2Hp pi-T-sheped
		4	7.719783348080	-0.36	H.-.H
		7	7.775625567831	-0.30	
		8	7.852390658038	-0.22	
		5	8.055398701658	-0.02	
	acidm	9	0.000000000000	-5.51	8C.-.O 5C.-.H 6H.-.O 3C.-.C 3O.-.O 5H.-.H 3Hp Hb(AmtoM)
		1	3.460999844595	-2.05	3C.-.C 7C.-.O 8C.-.H 8H.-.O 6H.-.H 3Hp
		2	4.033630139496	-1.47	2C.-.C 10C.-.H 6H.-.H 2C.-.O 2H.-.O 2Hp
		0	5.279393403828	-0.23	
		3	5.330057508551	-0.18	

Continue in the next page

Table SM1: Structures of various conformations are evaluated for their energetic properties and types of intermolecular interactions. In this context, Am stands for amino acid, FM for functional monomer, Nº conf. for the spatial conformation number of the Amino acid-FM complex, E_{tot} represents the ground state electronic energy in kcal mol^{-1}, the ΔE represents the difference of the electronic energy in ascending order of energy between the complex and lastly, the type of interaction specifies the atoms that are in close proximity in the table. The symbols Hb denote a hydrogen bond, AmtoM indicates that an AM is complexing with an FM, and MtoAm is the reverse of AmtoM. The symbols SB denote a salt bridges. The symbols Hp denote a hydrophobics interactions. The symbols Cation-pi/pi-staquing/pi-T-sheped denote the type of π interactions interactions.

AA	FM	Nº conf.	ΔE	E_{tot}	Type of interaction
		6	5.526812607815	0.02	
	acida	4	0.000000000000	-5.14	C.-.C 4C.-.H 5C.-.O 4H.-.O 2O.-.O 2H.-.H Hp Hb(MtoAm)
		5	2.809294548760	-2.33	2C.-.N 13C.-.H 4C.-.O 2H.-.N 9H.-.H 6H.-.O C.-.C Hp SB
		0	3.230468364330	-1.91	8C.-.H 2H.-.N 5H.-.H 6H.-.O N.-.O 2C.-.O 2O.-.O Hb(AmtoM) SB
		2	5.050496374330	-0.09	
		3	5.206591893429	0.07	
		6	5.307196930397	0.17	
	bisac	6	0.000000000000	-7.39	33C.-.H 18C.-.C 4C.-.O 19H.-.H 7H.-.O 8H.-.N N.-.O 3C.-.N O.-.O 18Hp Hb(MtoAm)
		7	0.206762989531	-7.18	35C.-.H 17C.-.C 7C.-.O 17H.-.H 6H.-.O 8H.-.N 4C.-.N N.-.O O.-.O 17Hp Hb(MtoAm)

Continue in the next page

Table SM1: Structures of various conformations are evaluated for their energetic properties and types of intermolecular interactions. In this context, Am stands for amino acid, FM for functional monomer, Nº conf. for the spatial conformation number of the Amino acid-FM complex, E_{tot} represents the ground state electronic energy in kcal mol^{-1}, the ΔE represents the difference of the electronic energy in ascending order of energy between the complex and lastly, the type of interaction specifies the atoms that are in close proximity in the table. The symbols Hb denote a hydrogen bond, AmtoM indicates that an AM is complexing with an FM, and MtoAm is the reverse of AmtoM. The symbols SB denote a salt bridges. The symbols Hp denote a hydrophobics interactions. The symbols Cation-pi/pi-staquing/pi-T-sheped denote the type of π interactions interactions.

AA	FM	Nº conf.	ΔE	E_{tot}	Type of interaction
		8	1.488724206001	-5.90	3C.-.C 7C.-.O 4C.-.H 3H.-.H 6H.-.O 2O.-.O N.-.O H.-.N 3Hp Hb(AmtoM)
		0	2.728720195561	-4.66	21C.-.H 3C.-.C 3C.-.O 12H.-.H 4H.-.O 3C.-.N 6H.-.N N.-.O N.-.N 3Hp Hb(AmtoM)
		2	7.283012439832	-0.11	
		9	7.296335944141	-0.09	
	1ally	9	0.000000000000	-5.06	9H.-.N 25H.-.H 4C.-.N 31C.-.H 3C.-.C 2C.-.O 4H.-.O N.-.N 3Hp Hb(AmtoM) 2SB
		5	1.247685850630	-3.81	N.-.N 7H.-.N 16H.-.H C.-.N 13C.-.H H.-.O Hb(AmtoM) SB
		2	1.885107179181	-3.17	H.-.N 3C.-.O 8C.-.H 4H.-.O 6H.-.H 2C.-.C 2Hp
		6	2.192761340618	-2.86	8C.-.C 16C.-.H 12H.-.H 2H.-.O C.-.O 8Hp Cation-pi(Mc)

Continue in the next page

Table SM1: Structures of various conformations are evaluated for their energetic properties and types of intermolecular interactions. In this context, Am stands for amino acid, FM for functional monomer, N° conf. for the spatial conformation number of the Amino acid-FM complex, E_{tot} represents the ground state electronic energy in kcal mol^{-1}, the ΔE represents the difference of the electronic energy in ascending order of energy between the complex and lastly, the type of interaction specifies the atoms that are in close proximity in the table. The symbols Hb denote a hydrogen bond, AmtoM indicates that an AM is complexing with an FM, and MtoAm is the reverse of AmtoM. The symbols SB denote a salt bridges. The symbols Hp denote a hydrophobics interactions. The symbols Cation-pi/pi-staquing/pi-T-sheped denote the type of π interactions interactions.

AA	FM	N° conf.	ΔE	E_{tot}	Type of interaction
		4	2.394430128485	-2.66	23C.-.H 14H.-.H 4H.-.O 4C.-.C 2H.-.N C.-.O 4Hp
	4imid	4	0.000000000000	-9.20	O.-.O C.-.C 5C.-.O 5C.-.H 3H.-.O 2H.-.H 2H.-.N C.-.N 2N.-.O Hp Hb(AmtoM)
		0	0.414997791121	-8.79	5C.-.H 3H.-.O 2H.-.H 3C.-.O C.-.N 2N.-.O 2H.-.N C.-.C Hp Hb(AmtoM)
		1	4.383066090248	-4.82	4H.-.O 4C.-.O 11C.-.H 4H.-.H 2O.-.O C.-.C C.-.N 2H.-.N Hp Hb(AmtoM)
		6	5.208622659256	-3.99	3C.-.O 2O.-.O 5H.-.O 3C.-.H 4H.-.H Hb(AmtoM)
		9	9.026999717018	-0.17	
	acril	2	0.000000000000	-5.86	4C.-.O 4C.-.H 3H.-.O 2H.-.H H.-.N O.-.O Hb(AmtoM)

Continue in the next page

Table SM1: Structures of various conformations are evaluated for their energetic properties and types of intermolecular interactions. In this context, Am stands for amino acid, FM for functional monomer, N° conf. for the spatial conformation number of the Amino acid-FM complex, E_{tot} represents the ground state electronic energy in kcal mol^{-1}, the ΔE represents the difference of the electronic energy in ascending order of energy between the complex and lastly, the type of interaction specifies the atoms that are in close proximity in the table. The symbols Hb denote a hydrogen bond, AmtoM indicates that an AM is complexing with an FM, and MtoAm is the reverse of AmtoM. The symbols SB denote a salt bridges. The symbols Hp denote a hydrophobics interactions. The symbols Cation-pi/pi-staquing/pi-T-sheped denote the type of π interactions interactions.

AA	FM	N° conf.	ΔE	E_{tot}	Type of interaction
		4	0.349211881419	-5.51	2C.-.C 7C.-.H 5C.-.O 4H.-.H 3H.-.O H.-.N O.-.O 2Hp Hb(AmtoM)
		6	2.291399753242	-3.57	12C.-.H C.-.O 4H.-.N 10H.-.H 6H.-.O 2C.-.N C.-.C N.-.O Hp Hb(AmtoM)
		3	2.331834235429	-3.53	11C.-.H C.-.C 2C.-.O 12H.-.H 2H.-.N 5H.-.O C.-.N N.-.O Hp Hb(MtoAm)
		0	3.121718445067	-2.74	2C.-.N 6C.-.H 3C.-.O 2H.-.N 3H.-.H 4H.-.O N.-.O 2O.-.O Hb(AmtoM)
		5	4.048913239363	-1.82	5H.-.H 4C.-.H 3H.-.N N.-.O C.-.O 4H.-.O O.-.O Hb(AmtoM)
		8	5.779630026672	-0.08	
	alila	3	0.000000000000	-11.59	3C.-.O 4C.-.H 6H.-.O 5H.-.H C.-.N 2N.-.O H.-.N Hb(AmtoM) SB

Continue in the next page

Table SM1: Structures of various conformations are evaluated for their energetic properties and types of intermolecular interactions. In this context, Am stands for amino acid, FM for functional monomer, Nº conf. for the spatial conformation number of the Amino acid-FM complex, E_{tot} represents the ground state electronic energy in kcal mol^{-1}, the ΔE represents the difference of the electronic energy in ascending order of energy between the complex and lastly, the type of interaction specifies the atoms that are in close proximity in the table. The symbols Hb denote a hydrogen bond, AmtoM indicates that an AM is complexing with an FM, and MtoAm is the reverse of AmtoM. The symbols SB denote a salt bridges. The symbols Hp denote a hydrophobics interactions. The symbols Cation-pi/pi-staquing/pi-T-sheped denote the type of π interactions interactions.

AA	FM	Nº conf.	ΔE	E_{tot}	Type of interaction
		1	8.619993386115	-2.97	4C.-.C 5H.-.N 14H.-.H 19C.-.H N.-.N 4C.-.N H.-.O 4Hp Cation-pi(Mc)
		0	9.209530344131	-2.39	2C.-.N 3C.-.C 16C.-.H 2H.-.N 10H.-.H 3Hp
		5	10.630579224031	-0.96	4C.-.H C.-.O H.-.N 2H.-.H 2H.-.O
		4	11.470375712449	-0.12	
		6	11.593034894370	-0.00	
		2	11.627862277145	0.03	
		9	11.628845204341	0.03	
		8	11.841468078548	0.25	
	estir	5	0.000000000000	-5.08	4C.-.N 22C.-.H 13H.-.H 7C.-.C 5C.-.O 4H.-.O 7Hp Cation-pi(Amc)
		0	2.222191426552	-2.86	15C.-.H 10H.-.H 4C.-.N 2H.-.N
		1	2.321234023060	-2.76	8C.-.C 24C.-.H 13H.-.H 4C.-.N H.-.N H.-.O 8Hp pi-staquing

Continue in the next page

Table SM1: Structures of various conformations are evaluated for their energetic properties and types of intermolecular interactions. In this context, Am stands for amino acid, FM for functional monomer, Nº conf. for the spatial conformation number of the Amino acid-FM complex, E_{tot} represents the ground state electronic energy in kcal mol^{-1}, the ΔE represents the difference of the electronic energy in ascending order of energy between the complex and lastly, the type of interaction specifies the atoms that are in close proximity in the table. The symbols Hb denote a hydrogen bond, AmtoM indicates that an AM is complexing with an FM, and MtoAm is the reverse of AmtoM. The symbols SB denote a salt bridges. The symbols Hp denote a hydrophobics interactions. The symbols Cation-pi/pi-staquing/pi-T-sheped denote the type of π interactions interactions.

AA	FM	Nº conf.	ΔE	E_{tot}	Type of interaction
		2	2.684703340331	-2.39	13C.-.H C.-.N 3C.-.C 7H.-.H H.-.N 3Hp
		3	3.390361478408	-1.69	12C.-.H 11H.-.H 2C.-.N 2H.-.N
		7	5.095094848618	0.02	
		8	5.239307910155	0.16	
	1viny	3	0.000000000000	-9.43	2C.-.O 3C.-.H C.-.N 2N.-.O 2H.-.N 3H.-.O 2H.-.H Hb(AmtoM)
		9	5.005457651781	-4.42	18C.-.H 7H.-.N 4C.-.N 3C.-.C 2C.-.O 13H.-.H 2H.-.O 3Hp
		0	7.716263380247	-1.71	7H.-.H 3C.-.C 11C.-.H 3Hp
		7	8.615917127936	-0.81	4C.-.H C.-.O 2H.-.O H.-.N 2H.-.H
		5	8.890080719588	-0.54	C.-.O C.-.H 2H.-.O H.-.H
		4	9.108546830272	-0.32	
		8	9.344635412576	-0.08	
		6	9.511467710013	0.08	
		2	9.600156598716	0.17	

Continue in the next page

Table SM1: Structures of various conformations are evaluated for their energetic properties and types of intermolecular interactions. In this context, Am stands for amino acid, FM for functional monomer, N° conf. for the spatial conformation number of the Amino acid-FM complex, E_{tot} represents the ground state electronic energy in kcal mol^{-1}, the ΔE represents the difference of the electronic energy in ascending order of energy between the complex and lastly, the type of interaction specifies the atoms that are in close proximity in the table. The symbols Hb denote a hydrogen bond, AmtoM indicates that an AM is complexing with an FM, and MtoAm is the reverse of AmtoM. The symbols SB denote a salt bridges. The symbols Hp denote a hydrophobics interactions. The symbols Cation-pi/pi-staquing/pi-T-sheped denote the type of π interactions interactions.

AA	FM	N° conf.	ΔE	E_{tot}	Type of interaction
	2hydr	6	0.000000000000	-5.37	16C.-.C 7C.-.O 31C.-.H 9H.-.O 19H.-.H C.-.N H.-.N O.-.O 16Hp
		9	0.677357222414	-4.69	32C.-.H 10C.-.C 21H.-.H 2H.-.N C.-.N 5C.-.O N.-.O 10H.-.O O.-.O 10Hp Hb(MtoAm)
		4	3.343490091115	-2.03	C.-.N 22C.-.H 3C.-.C 4H.-.O 15H.-.H H.-.N C.-.O 3Hp
		0	5.585820686525	0.22	
		1	6.049386038573	0.68	
	4viny	0	0.000000000000	-4.50	5C.-.C 19C.-.H 15H.-.H 4H.-.N 3C.-.N 3C.-.O 3H.-.O 5Hp pi-T-sheped
		1	0.260562922666	-4.23	7C.-.H 7H.-.H H.-.O N.-.N 5H.-.N 4C.-.N C.-.C Hp Hb(AmtoM)
		8	1.054127657623	-3.44	19C.-.H 3C.-.C 15H.-.H 3H.-.N 5C.-.N N.-.O C.-.O H.-.O 3Hp Cation-pi(Amc)

Continue in the next page

Table SM1: Structures of various conformations are evaluated for their energetic properties and types of intermolecular interactions. In this context, Am stands for amino acid, FM for functional monomer, Nº conf. for the spatial conformation number of the Amino acid-FM complex, E_{tot} represents the ground state electronic energy in kcal mol^{-1}, the ΔE represents the difference of the electronic energy in ascending order of energy between the complex and lastly, the type of interaction specifies the atoms that are in close proximity in the table. The symbols Hb denote a hydrogen bond, AmtoM indicates that an AM is complexing with an FM, and MtoAm is the reverse of AmtoM. The symbols SB denote a salt bridges. The symbols Hp denote a hydrophobics interactions. The symbols Cation-pi/pi-staquing/pi-T-sheped denote the type of π interactions interactions.

AA	FM	Nº conf.	ΔE	E_{tot}	Type of interaction
		2	2.169556701000	-2.33	16C.-.H 6C.-.C C.-.N 13H.-.H 2H.-.N 6Hp pi-T-sheped
		6	2.371416489727	-2.12	12C.-.H C.-.N C.-.C 2C.-.O 2H.-.N 11H.-.H 2H.-.O Hp
	acrol	9	0.000000000000	-4.98	2C.-.H 3C.-.O 3H.-.O H.-.H O.-.O Hb(AmtoM)
		8	0.538342636326	-4.44	16C.-.C 18C.-.H 4C.-.O 11H.-.H 4H.-.O 16Hp
		5	2.144871668597	-2.83	13C.-.H 2C.-.C 8H.-.H 5H.-.O N.-.O 2C.-.O O.-.O 2Hp Hb(AmtoM)
		4	3.894791806162	-1.08	13C.-.H 11H.-.H C.-.N 3C.-.C 2C.-.O H.-.N 3H.-.O O.-.O 3Hp
		3	3.973030444417	-1.01	6C.-.H 4C.-.O 5H.-.O 6H.-.H C.-.C Hp
		2	4.408830148112	-0.57	C.-.N 7C.-.H 5H.-.H H.-.N N.-.O 3H.-.O C.-.O O.-.O Hb(AmtoM)
		7	4.929778877289	-0.05	

Continue in the next page

Table SM1: Structures of various conformations are evaluated for their energetic properties and types of intermolecular interactions. In this context, Am stands for amino acid, FM for functional monomer, N° conf. for the spatial conformation number of the Amino acid-FM complex, E_{tot} represents the ground state electronic energy in kcal mol^{-1}, the ΔE represents the difference of the electronic energy in ascending order of energy between the complex and lastly, the type of interaction specifies the atoms that are in close proximity in the table. The symbols Hb denote a hydrogen bond, AmtoM indicates that an AM is complexing with an FM, and MtoAm is the reverse of AmtoM. The symbols SB denote a salt bridges. The symbols Hp denote a hydrophobics interactions. The symbols Cation-pi/pi-staquing/pi-T-sheped denote the type of π interactions interactions.

AA	FM	N° conf.	ΔE	E_{tot}	Type of interaction
		0	5.088433750175	0.11	
	itaco	5	0.000000000000	-17.09	2C.-.N 3H.-.N N.-.O 25C.-.H 16H.-.H 7H.-.O 11C.-.C 5C.-.O 11Hp Hb(AmtoM) SB
		1	11.490334794249	-5.60	C.-.N 3H.-.N 26C.-.H 13H.-.H 7H.-.O 12C.-.C 5C.-.O O.-.O 12Hp Hb(MtoAm) SB
		7	11.963207462210	-5.12	9C.-.H 2C.-.C 6C.-.O 4H.-.H 5H.-.O O.-.O 2Hp Hb(MtoAm)
		4	12.237741251454	-4.85	H.-.N 9H.-.O 7H.-.H 14C.-.H 7C.-.O 2C.-.C O.-.O 2Hp Hb(AmtoM) SB
		8	14.712767694859	-2.37	
		6	15.147102969912	-1.94	9C.-.H 6C.-.O 5H.-.O 3H.-.H C.-.C Hp
VAL	14dvb	2	0.000000000000	-3.89	22C.-.H 5C.-.N 6C.-.C 12H.-.H 2H.-.N C.-.O H.-.O 6Hp Cation-pi(Amc)

Continue in the next page

Table SM1: Structures of various conformations are evaluated for their energetic properties and types of intermolecular interactions. In this context, Am stands for amino acid, FM for functional monomer, N° conf. for the spatial conformation number of the Amino acid-FM complex, E_{tot} represents the ground state electronic energy in kcal mol^{-1}, the ΔE represents the difference of the electronic energy in ascending order of energy between the complex and lastly, the type of interaction specifies the atoms that are in close proximity in the table. The symbols Hb denote a hydrogen bond, AmtoM indicates that an AM is complexing with an FM, and MtoAm is the reverse of AmtoM. The symbols SB denote a salt bridges. The symbols Hp denote a hydrophobics interactions. The symbols Cation-pi/pi-staquing/pi-T-sheped denote the type of π interactions interactions.

AA	FM	N° conf.	ΔE	E_{tot}	Type of interaction
		3	0.778357447221	-3.11	24C.-.H C.-.C 4C.-.N 17H.-.H 2H.-.N Hp Cation-pi(Amc)
		5	2.157058833605	-1.73	2C.-.N 17C.-.H C.-.O 12H.-.H 2H.-.N 2H.-.O C.-.C Hp
		1	2.189235358399	-1.70	15C.-.H 2C.-.C 12H.-.H 2Hp
		7	2.687329945538	-1.20	6C.-.H 6H.-.H
		9	3.139636834145	-0.75	2C.-.O 9C.-.H 11H.-.H 4H.-.O
		0	3.822751008205	-0.06	
		8	3.901651828917	0.02	
	2viny	0	0.000000000000	-8.88	5C.-.O 11C.-.H C.-.N 2N.-.O H.-.N C.-.C 4H.-.O 8H.-.H Hp Hb(AmtoM)
		5	4.970566778527	-3.91	4C.-.C 5C.-.N 16C.-.H 4C.-.O N.-.N 7H.-.N 11H.-.H 2H.-.O 4Hp
		7	5.501805789490	-3.37	5C.-.C 18C.-.H 2C.-.O C.-.N 4H.-.N N.-.N 11H.-.H 2H.-.O 5Hp

Continue in the next page

Table SM1: Structures of various conformations are evaluated for their energetic properties and types of intermolecular interactions. In this context, Am stands for amino acid, FM for functional monomer, Nº conf. for the spatial conformation number of the Amino acid-FM complex, E_{tot} represents the ground state electronic energy in kcal mol^{-1}, the ΔE represents the difference of the electronic energy in ascending order of energy between the complex and lastly, the type of interaction specifies the atoms that are in close proximity in the table. The symbols Hb denote a hydrogen bond, AmtoM indicates that an AM is complexing with an FM, and MtoAm is the reverse of AmtoM. The symbols SB denote a salt bridges. The symbols Hp denote a hydrophobics interactions. The symbols Cation-pi/pi-staquing/pi-T-sheped denote the type of π interactions interactions.

AA	FM	Nº conf.	ΔE	E_{tot}	Type of interaction
		8	5.877356975535	-3.00	8C.-.C 20C.-.H C.-.N N.-.O H.-.N C.-.O 11H.-.H H.-.O 8Hp
		1	6.588444269226	-2.29	16C.-.H 2H.-.N 9H.-.H 2C.-.C C.-.O 2H.-.O 2Hp
		9	8.602621931862	-0.27	C.-.N 3C.-.H 2H.-.N 4H.-.H
		9	8.602621931862	-0.27	erro
		3	8.757759245276	-0.12	
		2	8.864628585707	-0.01	
		6	8.991119888222	0.12	
	acidm	7	0.000000000000	-2.25	4C.-.N 13C.-.H 3H.-.N 11H.-.H 4C.-.O C.-.C 9H.-.O Hp SB
		0	0.750267623889	-1.50	2C.-.C 13C.-.H 14H.-.H 2Hp
		4	0.830812618201	-1.42	7C.-.O 10C.-.H 6H.-.O 9H.-.H 4C.-.C 2O.-.O 4Hp
		1	2.230440499939	-0.02	
		6	2.364310093961	0.11	
		9	2.481543299152	0.23	
		5	2.573547386650	0.32	

Continue in the next page

Table SM1: Structures of various conformations are evaluated for their energetic properties and types of intermolecular interactions. In this context, Am stands for amino acid, FM for functional monomer, N° conf. for the spatial conformation number of the Amino acid-FM complex, E_{tot} represents the ground state electronic energy in kcal mol^{-1}, the ΔE represents the difference of the electronic energy in ascending order of energy between the complex and lastly, the type of interaction specifies the atoms that are in close proximity in the table. The symbols Hb denote a hydrogen bond, AmtoM indicates that an AM is complexing with an FM, and MtoAm is the reverse of AmtoM. The symbols SB denote a salt bridges. The symbols Hp denote a hydrophobics interactions. The symbols Cation-pi/pi-staquing/pi-T-sheped denote the type of π interactions interactions.

AA	FM	N° conf.	ΔE	E_{tot}	Type of interaction
		8	4.890498755979	2.64	
	acida	2	0.000000000000	-3.13	3C.-.O 3C.-.H 5H.-.O 3H.-.H 2O.-.O Hb(AmtoM)
		0	1.269454893706	-1.86	3C.-.O 9C.-.H C.-.C 4H.-.O 6H.-.H Hp
		1	1.955762480678	-1.17	9C.-.H C.-.C 2C.-.N 7H.-.H 2H.-.N N.-.O 3H.-.O Hp SB
		6	2.883504660088	-0.25	2C.-.C C.-.O 7C.-.H 6H.-.O 6H.-.H 2Hp
		5	3.057426955736	-0.07	
		3	3.123164966165	-0.01	
		9	3.157408670670	0.03	
		7	3.216728023214	0.09	
		8	3.294857006567	0.17	
		4	6.739579206871	3.61	2C.-.N 5C.-.H C.-.O 2H.-.N 4H.-.H H.-.O SB

Continue in the next page

Table SM1: Structures of various conformations are evaluated for their energetic properties and types of intermolecular interactions. In this context, Am stands for amino acid, FM for functional monomer, N° conf. for the spatial conformation number of the Amino acid-FM complex, E_{tot} represents the ground state electronic energy in $kcal\,mol^{-1}$, the ΔE represents the difference of the electronic energy in ascending order of energy between the complex and lastly, the type of interaction specifies the atoms that are in close proximity in the table. The symbols Hb denote a hydrogen bond, AmtoM indicates that an AM is complexing with an FM, and MtoAm is the reverse of AmtoM. The symbols SB denote a salt bridges. The symbols Hp denote a hydrophobics interactions. The symbols Cation-pi/pi-staquing/pi-T-sheped denote the type of π interactions interactions.

AA	FM	N° conf.	ΔE	E_{tot}	Type of interaction
	bisac	2	0.000000000000	-6.68	5C.-.H 5H.-.H 3C.-.O 2N.-.O 2H.-.N 2O.-.O 4H.-.O Hb(AmtoM) Hb(MtoAm)
		5	0.526492797874	-6.16	5C.-.O 8C.-.H 5H.-.H 5H.-.O N.-.O 2H.-.N 2O.-.O Hb(AmtoM)
		9	3.168654597712	-3.52	18C.-.H 4H.-.N 16H.-.H 3C.-.C 3C.-.O 8H.-.O N.-.O 3Hp Hb(AmtoM)
		7	4.090144905498	-2.59	9H.-.H 7C.-.H 3H.-.N 6C.-.O 2N.-.O 7H.-.O Hb(MtoAm)
		8	4.872656761020	-1.81	11C.-.H 13H.-.H H.-.O N.-.O 3H.-.N C.-.N 2C.-.O O.-.O 2C.-.C 2Hp
		3	5.114034150089	-1.57	10C.-.H N.-.N 4H.-.N 10H.-.H 3C.-.N N.-.O 2H.-.O C.-.C Hp
		6	6.819301623493	0.13	

Continue in the next page

Table SM1: Structures of various conformations are evaluated for their energetic properties and types of intermolecular interactions. In this context, Am stands for amino acid, FM for functional monomer, N° conf. for the spatial conformation number of the Amino acid-FM complex, E_{tot} represents the ground state electronic energy in kcal mol^{-1}, the ΔE represents the difference of the electronic energy in ascending order of energy between the complex and lastly, the type of interaction specifies the atoms that are in close proximity in the table. The symbols Hb denote a hydrogen bond, AmtoM indicates that an AM is complexing with an FM, and MtoAm is the reverse of AmtoM. The symbols SB denote a salt bridges. The symbols Hp denote a hydrophobics interactions. The symbols Cation-pi/pi-staquing/pi-T-sheped denote the type of π interactions interactions.

AA	FM	N° conf.	ΔE	E_{tot}	Type of interaction
		1	6.900351780066	0.22	5C.-.H C.-.O 3H.-.O 6H.-.H H.-.N
	1ally	3	0.000000000000	-3.71	29C.-.H 26H.-.H 2H.-.O 3C.-.N 5H.-.N C.-.C Hp SB
		7	2.426492295901	-1.28	10C.-.H 5H.-.O 15H.-.H C.-.O C.-.C Hp SB
		9	2.834304019821	-0.87	7H.-.H 3C.-.H H.-.N
		5	2.886450689934	-0.82	C.-.C 4C.-.H 6H.-.H Hp
		4	3.406891721423	-0.30	C.-.O C.-.H 2H.-.O 3H.-.H SB
		2	3.621763820825	-0.08	
		0	3.644943089350	-0.06	
		8	3.677075740346	-0.03	
		6	3.717983624384	0.01	
		1	3.762523348347	0.06	
	4imid	6	0.000000000000	-6.31	2N.-.O 4C.-.O 6H.-.O 2O.-.O C.-.N 4C.-.H H.-.N 2H.-.H Hb(MtoAm) Hb(AmtoM) SB

Continue in the next page

Table SM1: Structures of various conformations are evaluated for their energetic properties and types of intermolecular interactions. In this context, Am stands for amino acid, FM for functional monomer, N° conf. for the spatial conformation number of the Amino acid-FM complex, E_{tot} represents the ground state electronic energy in kcal mol^{-1}, the ΔE represents the difference of the electronic energy in ascending order of energy between the complex and lastly, the type of interaction specifies the atoms that are in close proximity in the table. The symbols Hb denote a hydrogen bond, AmtoM indicates that an AM is complexing with an FM, and MtoAm is the reverse of AmtoM. The symbols SB denote a salt bridges. The symbols Hp denote a hydrophobics interactions. The symbols Cation-pi/pi-staquing/pi-T-sheped denote the type of π interactions interactions.

AA	FM	N° conf.	ΔE	E_{tot}	Type of interaction
		8	3.930129325685	-2.38	2H.-.O 17C.-.H 3C.-.C 9H.-.H 3H.-.N 3Hp Cation-pi(Amc)
		2	4.303918942752	-2.01	4H.-.H N.-.O C.-.O 4C.-.H 3H.-.O
		0	5.609959686394	-0.71	3C.-.O 5H.-.O 7C.-.H 8H.-.H SB
		5	6.113433681063	-0.20	
		4	6.253590639854	-0.06	
		7	6.282728160720	-0.03	
	acril	7	0.000000000000	-4.44	C.-.C 3C.-.N C.-.O 2N.-.O 5H.-.O O.-.O N.-.N 4H.-.N 2C.-.H 4H.-.H Hp Hb(MtoAm)
		0	0.022728660885	-4.42	C.-.N C.-.O 4C.-.H 3H.-.N 2N.-.O 5H.-.O O.-.O 2H.-.H Hb(MtoAm) Hb(AmtoM)
		6	0.564231463014	-3.88	3C.-.C 2C.-.N 12C.-.H 4H.-.N 8H.-.H 2C.-.O N.-.O 5H.-.O 3Hp Hb(AmtoM)

Continue in the next page

Table SM1: Structures of various conformations are evaluated for their energetic properties and types of intermolecular interactions. In this context, Am stands for amino acid, FM for functional monomer, N° conf. for the spatial conformation number of the Amino acid-FM complex, E_{tot} represents the ground state electronic energy in kcal mol^{-1}, the ΔE represents the difference of the electronic energy in ascending order of energy between the complex and lastly, the type of interaction specifies the atoms that are in close proximity in the table. The symbols Hb denote a hydrogen bond, AmtoM indicates that an AM is complexing with an FM, and MtoAm is the reverse of AmtoM. The symbols SB denote a salt bridges. The symbols Hp denote a hydrophobics interactions. The symbols Cation-pi/pi-staquing/pi-T-sheped denote the type of π interactions interactions.

AA	FM	N° conf.	ΔE	E_{tot}	Type of interaction
		9	1.574064162684	-2.87	4C.-.C 4C.-.N 11C.-.H 10H.-.H 5H.-.N 2C.-.O N.-.O 5H.-.O 4Hp Hb(AmtoM)
		8	2.589056253590	-1.85	4C.-.C 11C.-.H C.-.N 10H.-.H 2H.-.N C.-.O 2H.-.O N.-.O 4Hp
		2	3.078631786722	-1.36	11C.-.H 13H.-.H 3C.-.C 2C.-.O 2H.-.O O.-.O 2C.-.N 3H.-.N N.-.O 3Hp
		3	3.285735234683	-1.16	3C.-.C 12C.-.H 14H.-.H 2H.-.O O.-.O C.-.N 2H.-.N 3Hp
		1	3.495109799402	-0.95	4C.-.O 7C.-.H 8H.-.H 6H.-.O C.-.C N.-.O C.-.N 2H.-.N Hp
		4	4.051689540819	-0.39	C.-.C 5C.-.H 7H.-.H Hp
		5	4.420839284776	-0.02	
	alila	8	0.000000000000	-3.28	3C.-.N 4C.-.H 8H.-.N 12H.-.H N.-.N Hb(AmtoM)

Continue in the next page

Table SM1: Structures of various conformations are evaluated for their energetic properties and types of intermolecular interactions. In this context, Am stands for amino acid, FM for functional monomer, N° conf. for the spatial conformation number of the Amino acid-FM complex, E_{tot} represents the ground state electronic energy in kcal mol^{-1}, the ΔE represents the difference of the electronic energy in ascending order of energy between the complex and lastly, the type of interaction specifies the atoms that are in close proximity in the table. The symbols Hb denote a hydrogen bond, AmtoM indicates that an AM is complexing with an FM, and MtoAm is the reverse of AmtoM. The symbols SB denote a salt bridges. The symbols Hp denote a hydrophobics interactions. The symbols Cation-pi/pi-staquing/pi-T-sheped denote the type of π interactions interactions.

AA	FM	N° conf.	ΔE	E_{tot}	Type of interaction
		9	0.300225888938	-2.98	10C.-.H 2C.-.O 7H.-.N 9H.-.H 2H.-.O N.-.N N.-.O Hb(AmtoM) SB
		6	1.356638938912	-1.92	2C.-.N 8C.-.H 13H.-.H 5H.-.N C.-.C Hp
		1	2.148166757044	-1.13	13C.-.H 11H.-.H H.-.O H.-.N
		0	2.684477825682	-0.59	4C.-.H 5H.-.H
		2	3.278432905594	0.00	
		5	3.349057166631	0.07	
	estir	8	0.000000000000	-3.98	26C.-.H 4C.-.C 12H.-.H 2H.-.O C.-.N H.-.N 4Hp
		7	0.254922994169	-3.73	3C.-.C 25C.-.H 14H.-.H 4C.-.N H.-.O 3Hp Cation-pi(Amc)
		1	0.364969768338	-3.62	23C.-.H 3C.-.N 14H.-.H 3C.-.C 2H.-.N 3Hp
		9	0.920091696503	-3.06	29C.-.H 4C.-.N 12H.-.H 3C.-.C 2C.-.O 3H.-.O 3Hp Cation-pi(Amc)

Continue in the next page

Table SM1: Structures of various conformations are evaluated for their energetic properties and types of intermolecular interactions. In this context, Am stands for amino acid, FM for functional monomer, N° conf. for the spatial conformation number of the Amino acid-FM complex, E_{tot} represents the ground state electronic energy in kcal mol^{-1}, the ΔE represents the difference of the electronic energy in ascending order of energy between the complex and lastly, the type of interaction specifies the atoms that are in close proximity in the table. The symbols Hb denote a hydrogen bond, AmtoM indicates that an AM is complexing with an FM, and MtoAm is the reverse of AmtoM. The symbols SB denote a salt bridges. The symbols Hp denote a hydrophobics interactions. The symbols Cation-pi/pi-staquing/pi-T-sheped denote the type of π interactions interactions.

AA	FM	N° conf.	ΔE	E_{tot}	Type of interaction
		2	0.929323548580	-3.05	7C.-.C 19C.-.H 14H.-.H C.-.O H.-.O 7Hp
		3	3.906797514581	-0.08	
		5	3.954511193823	-0.03	
		6	3.988557375527	0.00	
	1viny	5	0.000000000000	-2.55	3C.-.C 2C.-.N 8C.-.O 4N.-.O 4H.-.O 6C.-.H 3H.-.N 4H.-.H 3Hp
	2hydr	0	0.000000000000	-7.80	10C.-.O 15C.-.H 13H.-.O 2O.-.O 11H.-.H 4C.-.C 2C.-.N 3H.-.N N.-.O 4Hp Hb(MtoAm)
		8	3.374077323928	-4.43	3C.-.H 3H.-.O 3H.-.H 2C.-.O 2O.-.O Hb(AmtoM)
		4	5.116947577635	-2.69	2C.-.C 14C.-.H 3C.-.N 14H.-.H 4H.-.N 4H.-.O 2Hp
		2	6.807627706452	-1.00	C.-.N 7C.-.H C.-.C N.-.O 4H.-.O C.-.O 2H.-.N 11H.-.H Hp
		9	7.814013498111	0.01	

Continue in the next page

Table SM1: Structures of various conformations are evaluated for their energetic properties and types of intermolecular interactions. In this context, Am stands for amino acid, FM for functional monomer, N° conf. for the spatial conformation number of the Amino acid-FM complex, E_{tot} represents the ground state electronic energy in kcal mol^{-1}, the ΔE represents the difference of the electronic energy in ascending order of energy between the complex and lastly, the type of interaction specifies the atoms that are in close proximity in the table. The symbols Hb denote a hydrogen bond, AmtoM indicates that an AM is complexing with an FM, and MtoAm is the reverse of AmtoM. The symbols SB denote a salt bridges. The symbols Hp denote a hydrophobics interactions. The symbols Cation-pi/pi-staquing/pi-T-sheped denote the type of π interactions interactions.

AA	FM	N° conf.	ΔE	E_{tot}	Type of interaction
	4viny	5	0.000000000000	-10.11	C.-.C 3C.-.O 5C.-.H C.-.N 2N.-.O H.-.N 3H.-.O 2H.-.H Hp Hb(AmtoM)
		7	6.244672633934	-3.87	8C.-.C 18C.-.H 8C.-.O 13H.-.H C.-.N 2H.-.N 6H.-.O 8Hp
		1	7.879927642987	-2.23	2C.-.C 6C.-.O 11C.-.H 2N.-.O 2H.-.N 4H.-.O 4H.-.H 2Hp
		3	9.543993394335	-0.57	3H.-.N 6C.-.H 9H.-.H
		6	10.087088607214	-0.03	
		2	10.164467597836	0.05	
		9	10.302353893435	0.19	
		8	10.548116361068	0.44	
	acrol	3	0.000000000000	-2.99	C.-.C 2C.-.N 9C.-.H H.-.N 4H.-.H 2C.-.O N.-.O 5H.-.O Hp Hb(AmtoM)
		0	0.823996178002	-2.17	2C.-.N 13C.-.H 3C.-.O 3H.-.N 11H.-.H 5H.-.O 3C.-.C 3Hp

Continue in the next page

Table SM1: Structures of various conformations are evaluated for their energetic properties and types of intermolecular interactions. In this context, Am stands for amino acid, FM for functional monomer, N° conf. for the spatial conformation number of the Amino acid-FM complex, E_{tot} represents the ground state electronic energy in kcal mol^{-1}, the ΔE represents the difference of the electronic energy in ascending order of energy between the complex and lastly, the type of interaction specifies the atoms that are in close proximity in the table. The symbols Hb denote a hydrogen bond, AmtoM indicates that an AM is complexing with an FM, and MtoAm is the reverse of AmtoM. The symbols SB denote a salt bridges. The symbols Hp denote a hydrophobics interactions. The symbols Cation-pi/pi-staquing/pi-T-sheped denote the type of π interactions interactions.

AA	FM	N° conf.	ΔE	E_{tot}	Type of interaction
		5	1.290208434209	-1.70	2C.-.C 6C.-.H 7H.-.H 2C.-.O 3H.-.O O.-.O 2Hp
		2	1.305733879261	-1.69	5C.-.O 10C.-.H 8H.-.H 3C.-.C 2H.-.O O.-.O 3Hp
		1	1.774201922777	-1.22	2C.-.C 2C.-.N 11C.-.H 2H.-.N 9H.-.H 2Hp
		8	2.872255343050	-0.12	C.-.H H.-.O H.-.H
		4	3.007163001445	0.01	
		6	3.120353027544	0.13	
	itaco	0	0.000000000000	-4.54	7H.-.O 5C.-.O 6C.-.H 3H.-.H 4O.-.O C.-.C Hp Hb(AmtoM) SB
		2	0.233828532838	-4.31	
		1	1.303760644585	-3.24	6C.-.O 2O.-.O 8H.-.O 6H.-.H 4C.-.C 10C.-.H 3C.-.N 3H.-.N 4Hp 2SB
		9	1.601964582754	-2.94	3C.-.O 10H.-.O 5C.-.H 3H.-.H N.-.O O.-.O 2SB

Continue in the next page

Table SM1: Structures of various conformations are evaluated for their energetic properties and types of intermolecular interactions. In this context, Am stands for amino acid, FM for functional monomer, N° conf. for the spatial conformation number of the Amino acid-FM complex, E_{tot} represents the ground state electronic energy in kcal mol^{-1}, the ΔE represents the difference of the electronic energy in ascending order of energy between the complex and lastly, the type of interaction specifies the atoms that are in close proximity in the table. The symbols Hb denote a hydrogen bond, AmtoM indicates that an AM is complexing with an FM, and MtoAm is the reverse of AmtoM. The symbols SB denote a salt bridges. The symbols Hp denote a hydrophobics interactions. The symbols Cation-pi/pi-staquing/pi-T-sheped denote the type of π interactions interactions.

AA	FM	N° conf.	ΔE	E_{tot}	Type of interaction
		6	1.935223331689	-2.61	8H.-.O 15C.-.H 13H.-.H C.-.C 3C.-.O N.-.O Hp SB
		3	2.298463054563	-2.24	
		4	2.565469073431	-1.98	

6 Advanced insights for relevant amino acid-monomer complexes

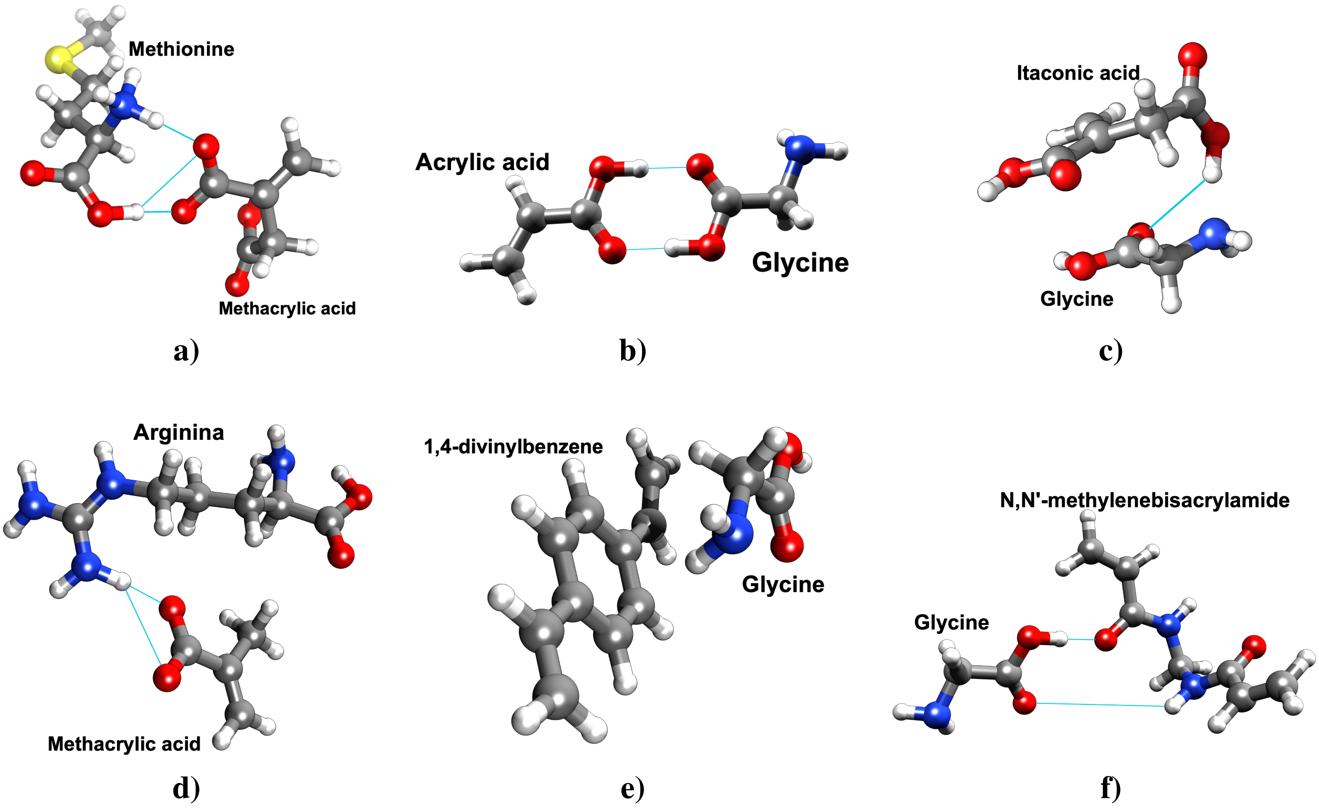

Figure SM38: Optimized structures of amino acid-monomer complexes, highlighting key interaction types, including van der Waals interactions, hydrogen bonds (Hb), salt bridges (SB), hydrophobic interactions (Hp), and π-type interactions (cation-π, π-stacking, π-T-shaped). The binding energies (ΔE) are provided in $\mathrm{kcal\,mol^{-1}}$. a) MET-itaco: $\Delta E = -14.08$, 2Hb (AmtoM), SB. b) GLY-acida: $\Delta E = -10.51$, Hb (MtoAm), Hb (AmtoM). c) GLY-itaco: $\Delta E = -13.64$, Hb (MtoAm), 2SB. d) ARG-acidm: $\Delta E = -18.38$, Hp, Hb (AmtoM), SB. e) GLY-14dvb: $\Delta E = -4.40$, cation-π. f) GLY-bisac: $\Delta E = -6.60$, Hb (AmtoM).

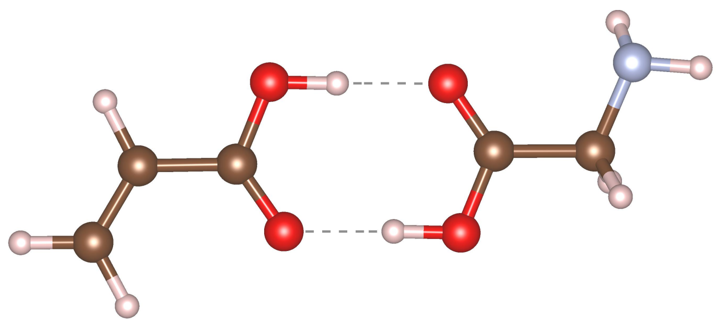

Figure SM39: Hydrogen bond of the Hb type in the AMtoM configuration between the MF acida and the amino acid GLY. Two hydrogen bonds can be observed: two very close, at n 1.7 Å each. The energy of this complex is $-10.51\ \mathrm{kcal\,mol^{-1}}$ (complex number 9), indicating it is a very stable complex.

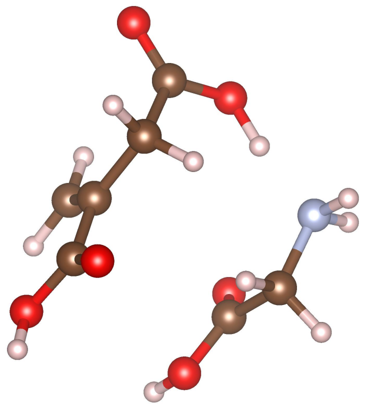

Figure SM40: Hydrogen bond of the Hb type in the AMtoM configuration between the MF itaco and the amino acid GLY. One hydrogen bonds can be observed, at 1.6 Å. The energy of this complex is $-13.64\ \mathrm{kcal\,mol^{-1}}$ (complex number 8), indicating it is a very stable complex.

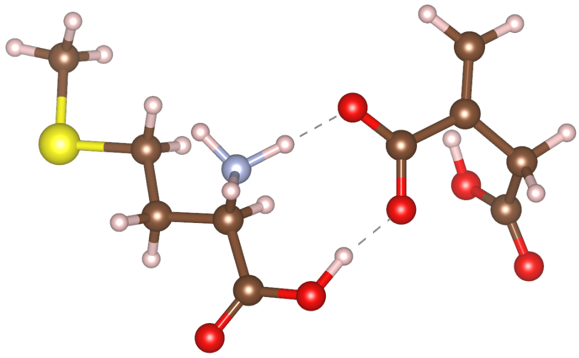

Figure SM41: Hydrogen bond of the Hb type in the AMtoM configuration between the MF itaco and the amino acid MET. Three hydrogen bonds can be observed: two very close, at less than 1.7 Å, and one more distant, at 2.5 Å. The energy of this complex is $-14.08\ \text{kcal}\,\text{mol}^{-1}$ (complex number 8), indicating it is a very stable complex.

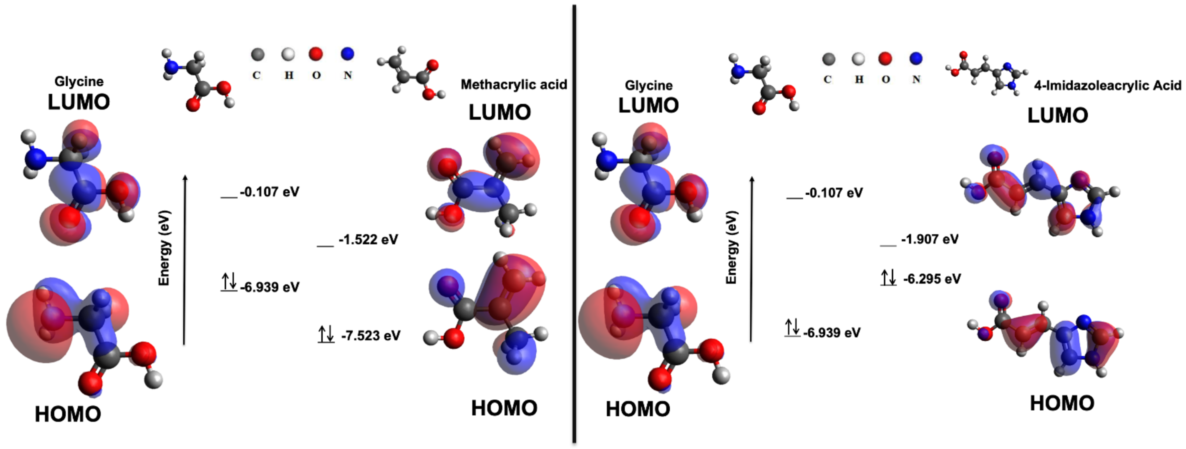

Figure SM42: Frontier molecular orbital (HOMO and LUMO) analysis for Glycine, Methacrylic Acid, and 4-Imidazoleacrylic Acid. The energy levels are shown in eV, with the positive phase in blue and the negative phase in red. The HOMO-LUMO gaps illustrate the electronic properties and interaction tendencies between the molecules, highlighting their electron-donating and accepting capabilities. The colors of the atoms are, respectively, hydrogen (H) in white, carbon (C) in gray, nitrogen (N) in blue, and oxygen (O) in red.

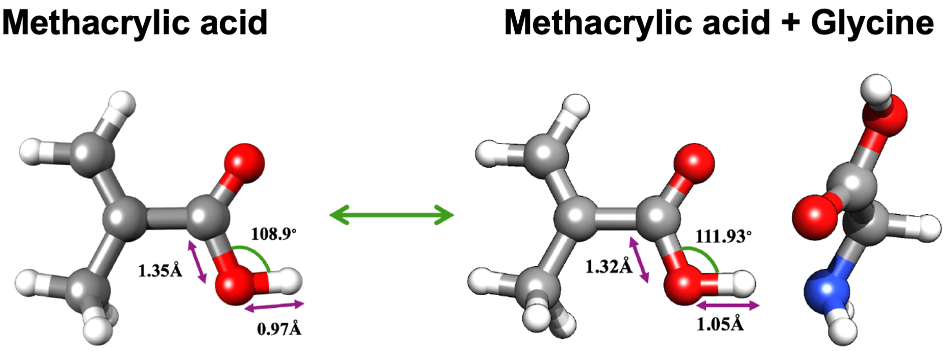

Figure SM43: Structural comparison highlighting the changes in methacrylic acid when unbound (left) versus when complexed with glycine (right).

7 Molecular Docking

7.1 Preparation and Validation of the Receptor Model

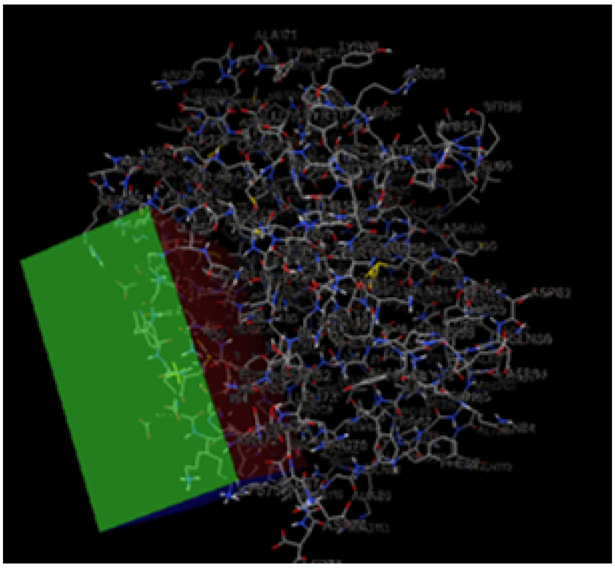

Figure SM44: Gridbox selected according to the region identical to PSA to carry out docking.

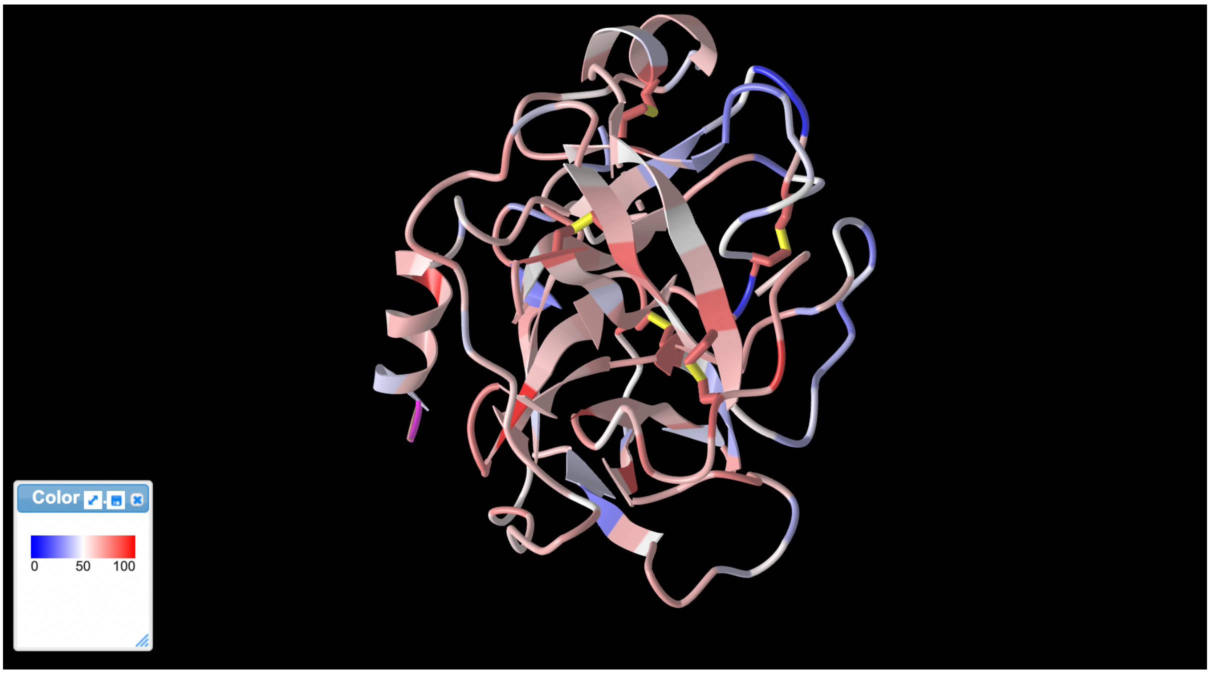

Figure SM45: Comparison of HPK homologation with a human PSA performed using BLAST analysis based on FASTA sequence information. The results highlight sequence similarities and differences, providing insights into the structural and functional relationships between HPK and human PSA.

7.2 Molecular docking results for the complexes formed between 1GVZ aminoacids and monomers

Table SM2: Docking results, all complexes formed with 1GVZ achieved RMSD < 2. Each monomer (e.g., 1viny, 2viny) forms a complex with amino acids from 1GVZ. "H-bond" means hydrogen bond and "Hydr." means hydrophobic bond. ΔG in (kcal/mol).

**Complex**	ΔG	**H-bond**	**Hydr.**
1ally	-2.60	Gly19	Ile16, Gly18, Gln156
1viny	-2.40	Gly19	Ile16, Gly18, Gln156
2hydr	-3.00	Ile16, Gly19, Gln156	Ile17, Gly18
2viny	-2.90	None	Ile16, Ile17, Gly18, Gln156
4imid	-3.10	Gly18, Gly19, Gln156	Ile16, Ile17, Glu21
4viny	-2.90	None	Trp20
acidm	-3.00	Ile16, Gly19	Ile17, Gly18, Gln156
acril	-2.50	Ile16, Ile17, Gly18	Gly19, Gln156
acrol	-2.10	Trp20, Glu159	HiSM184, Arg184
acida	-2.50	Ile16, Gly18, Gly19	Ile16, Gln156
alila	-2.00	Ile16	Gln156
estir	-3.40	None	Trp20, Glu21
itaco	-3.40	Ile16, Gly19	Gly18, Gln156

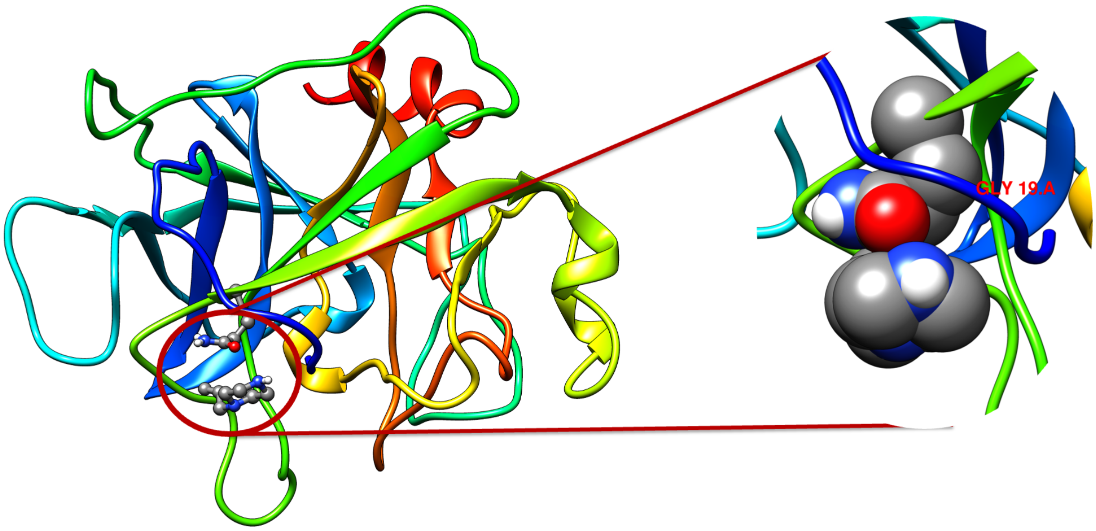

Figure SM46: 3D molecular affinity of 1-allylpiperazine with the amino acids present in the strategic region of the PSA protein is depicted in this figure, which includes a ribbon representation specifying the conformation of each protease peptide. The ΔG for 1-allylpiperazine found in Table SM1 is $-2.60\ \mathrm{kcal\,mol^{-1}}$.

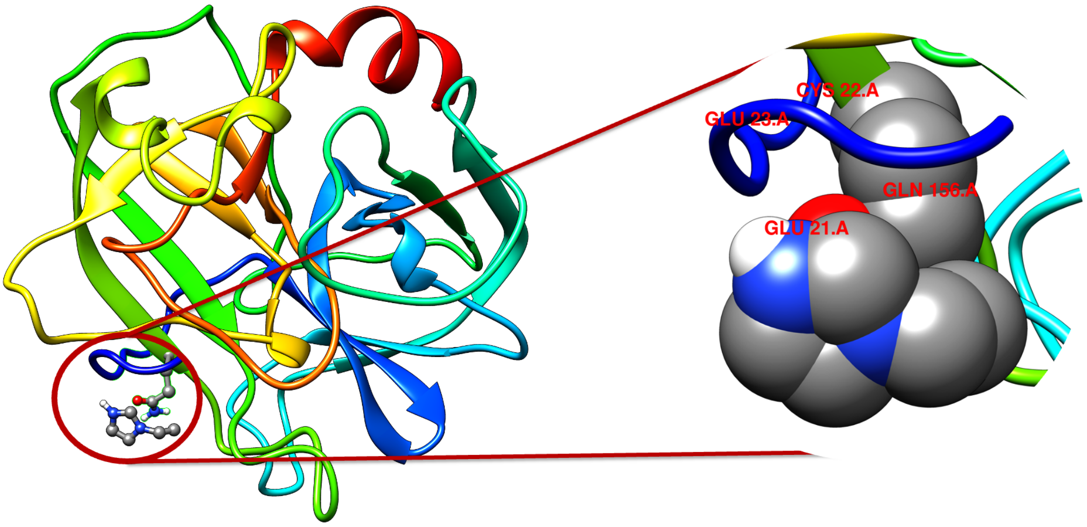

Figure SM47: 3D molecular affinity of 1-vinylimidazole with the amino acids present in the strategic region of the PSA protein is depicted in this figure, which includes a ribbon representation specifying the conformation of each protease peptide. The ΔG for 1-vinylimidazole found in Table SM1 is $-2.40\ \mathrm{kcal\,mol^{-1}}$.

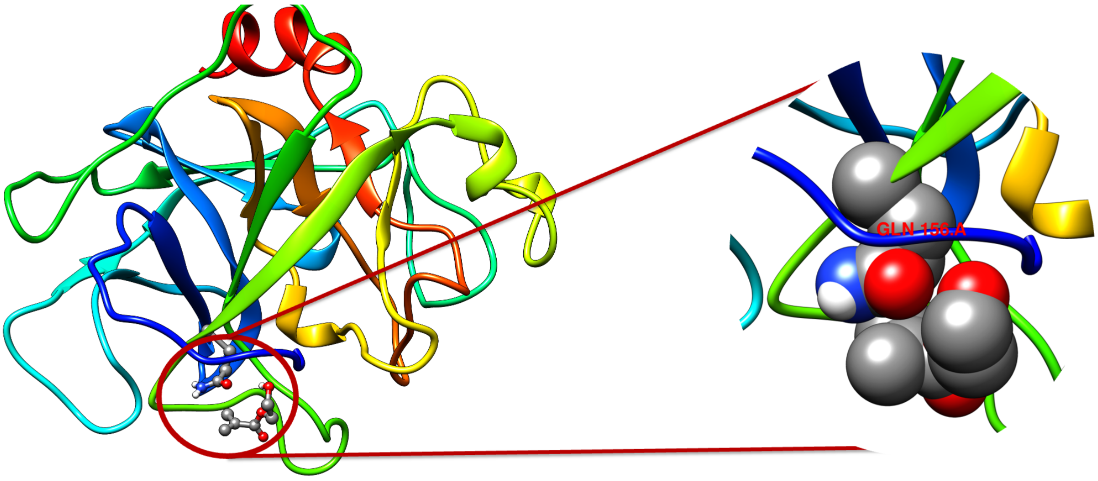

Figure SM48: 3D molecular affinity of 2-hydroxyethyl methacrylate with the amino acids present in the strategic region of the PSA protein is depicted in this figure, which includes a ribbon representation specifying the conformation of each protease peptide. The ΔG for 2-hydroxyethyl methacrylate found in Table SM1 is $-3.00\,\mathrm{kcal\,mol^{-1}}$.

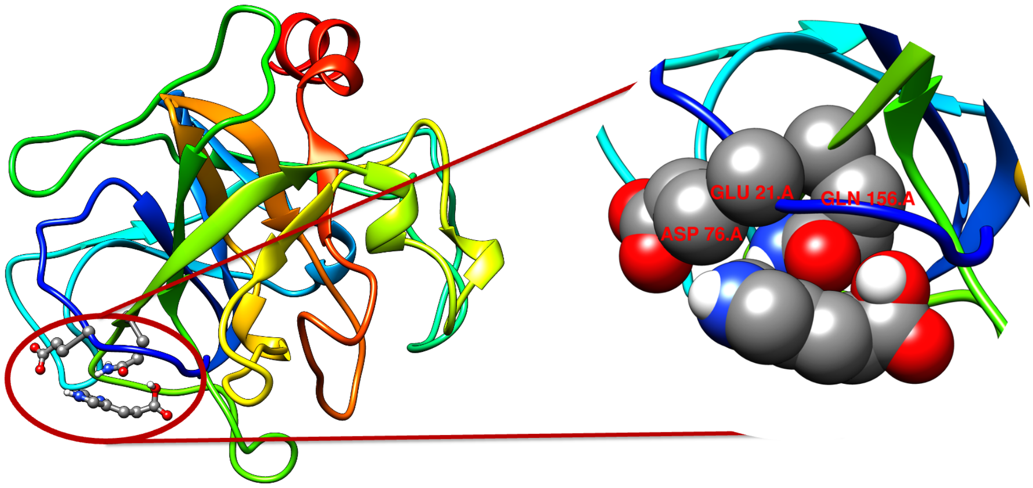

Figure SM49: 3D molecular affinity of 4-imidazoleacrylic acid with the amino acids present in the strategic region of the PSA protein is depicted in this figure, which includes a ribbon representation specifying the conformation of each protease peptide. The ΔG for 4-imidazoleacrylic acid found in Table SM1 is $-3.10\,\mathrm{kcal\,mol^{-1}}$.

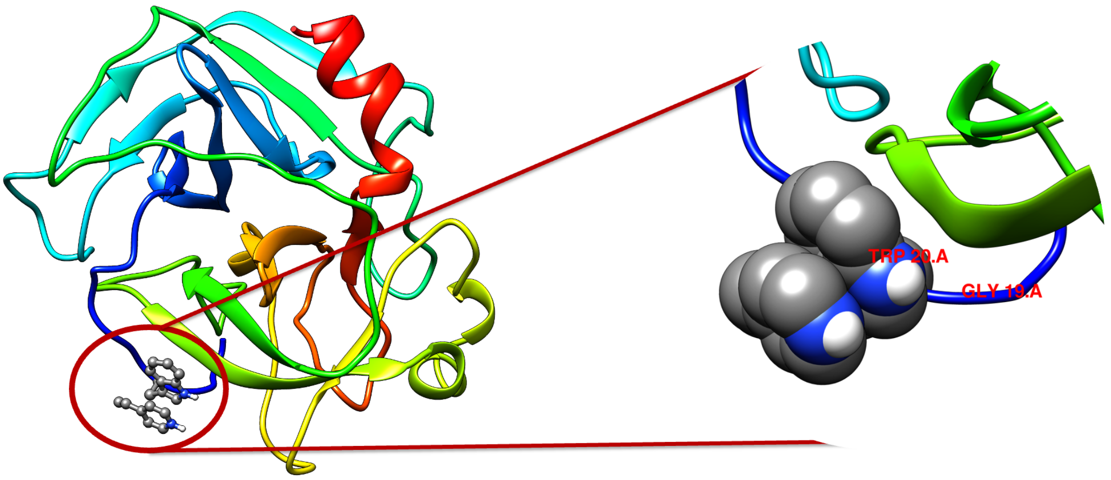

Figure SM50: 3D molecular affinity of 4-vinylpyridine with the amino acids present in the strategic region of the PSA protein is depicted in this figure, which includes a ribbon representation specifying the conformation of each protease peptide. The ΔG for 4-vinylpyridine found in Table SM1 is $-2.90\ \mathrm{kcal\,mol^{-1}}$.

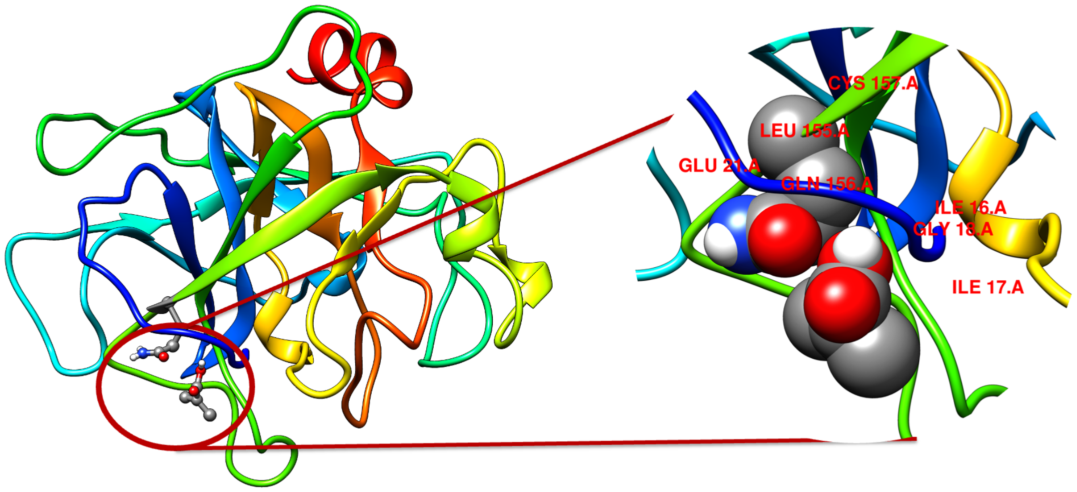

Figure SM51: 3D molecular affinity of methacrylic acid with the amino acids present in the strategic region of the PSA protein is depicted in this figure, which includes a ribbon representation specifying the conformation of each protease peptide. The ΔG for methacrylic acid found in Table SM1 is $-3.00\ \mathrm{kcal\,mol^{-1}}$.

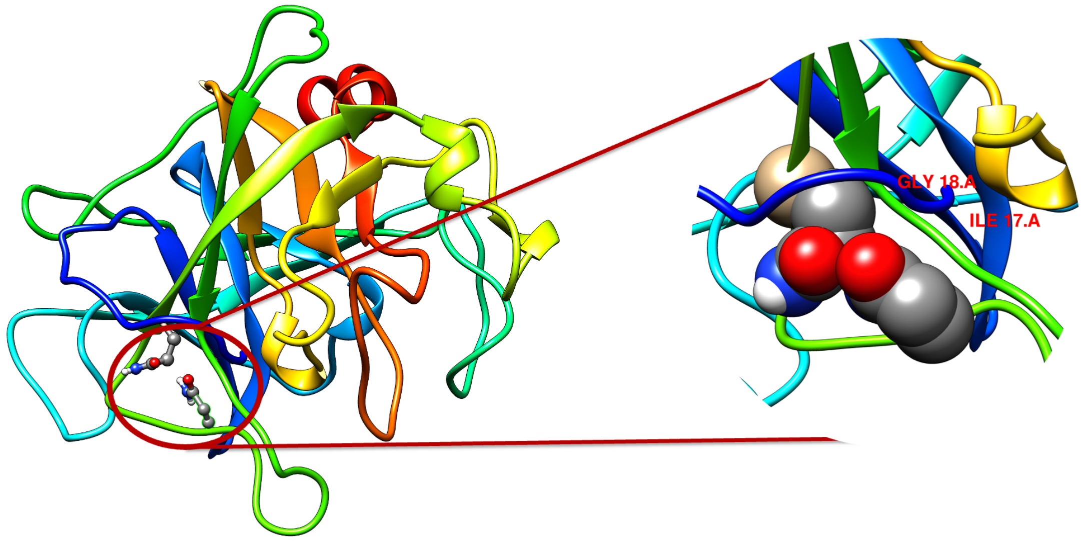

Figure SM52: 3D molecular affinity of acrylamide with the amino acids present in the strategic region of the PSA protein is depicted in this figure, which includes a ribbon representation specifying the conformation of each protease peptide. The ΔG for acrylamide found in Table SM1 is $-2.50\ \mathrm{kcal\,mol^{-1}}$.

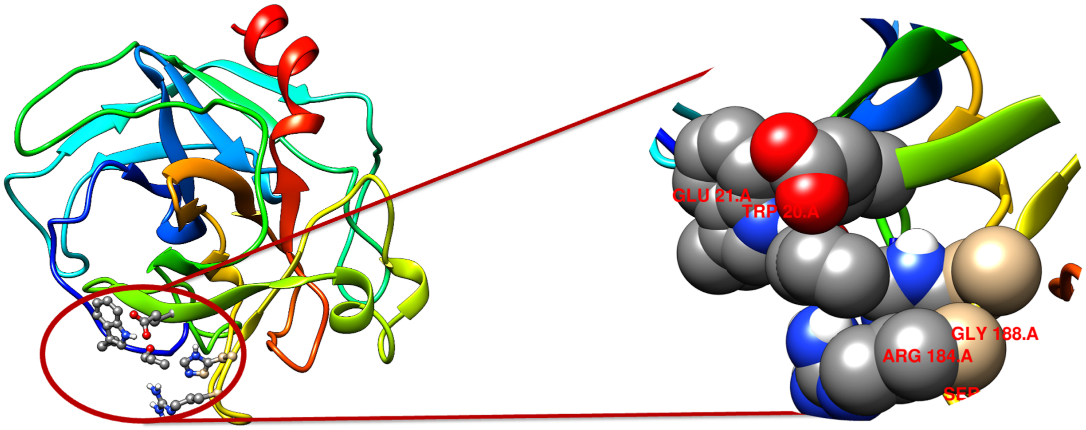

Figure SM53: 3D molecular affinity of acrolein with the amino acids present in the strategic region of the PSA protein is depicted in this figure, which includes a ribbon representation specifying the conformation of each protease peptide. The ΔG for acrolein found in Table SM1 is $-2.10\ \mathrm{kcal\,mol^{-1}}$.

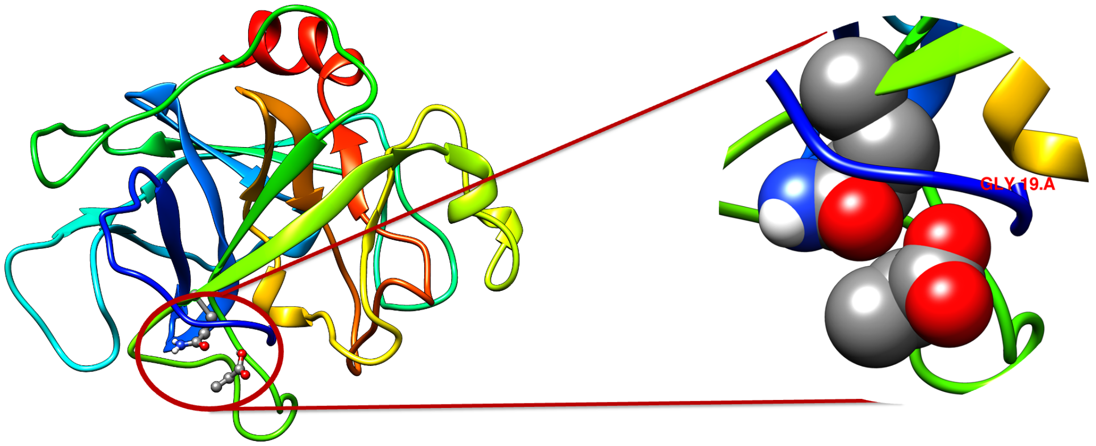

Figure SM54: 3D molecular affinity of acrylic acid with the amino acids present in the strategic region of the PSA protein is depicted in this figure, which includes a ribbon representation specifying the conformation of each protease peptide. The ΔG for acrylic acid found in Table SM1 is $-2.50\ \mathrm{kcal\,mol^{-1}}$.

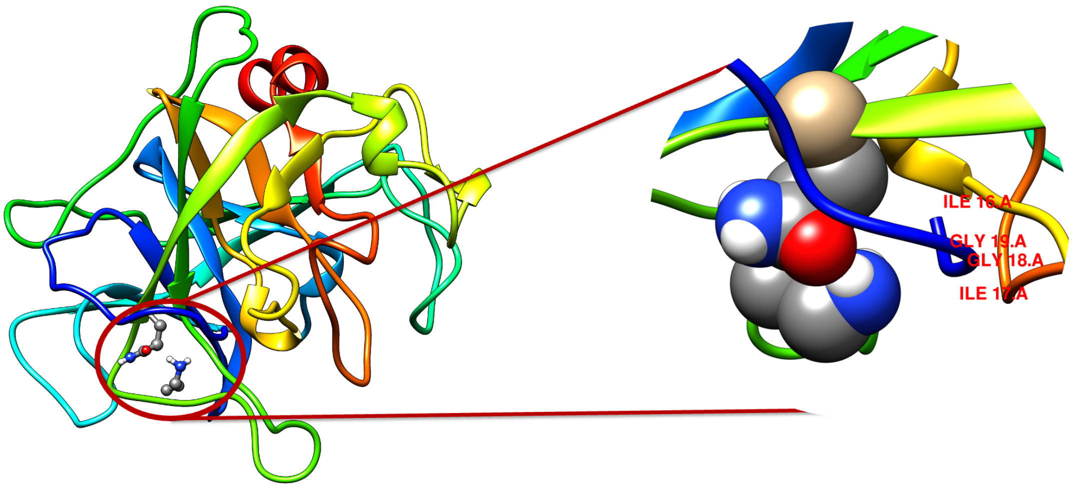

Figure SM55: 3D molecular affinity of allylamine with the amino acids present in the strategic region of the PSA protein is depicted in this figure, which includes a ribbon representation specifying the conformation of each protease peptide. The ΔG for allylamine found in Table SM1 is $-2.00\ \mathrm{kcal\,mol^{-1}}$.

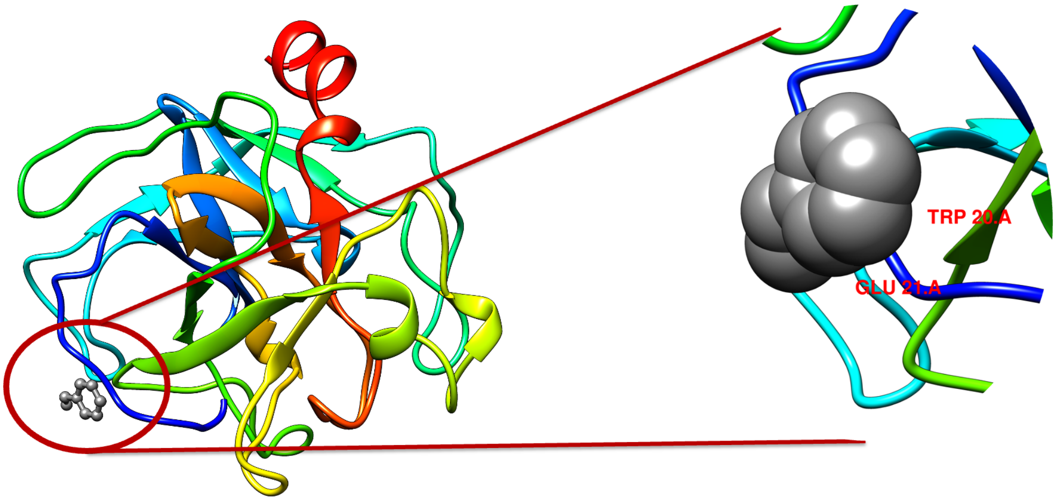

Figure SM56: 3D molecular affinity of styrene with the amino acids present in the strategic region of the PSA protein is depicted in this figure, which includes a ribbon representation specifying the conformation of each protease peptide. The ΔG for styrene found in Table SM1 is $-3.40\ \mathrm{kcal\,mol^{-1}}$.

8 Data and MBASM code availability

Additional raw data, e.g., XYZ optimized coordinates, and MBASM can be obtained directly from the authors upon request.
